# Supplementary material for: High-throughput chiral copper foils by curved-surface confinement recrystallization
Source: Nat Commun. 2026 Feb 20;17:2796. doi: 10.1038/s41467-026-69862-7 (PMC13022495; doi:10.1038/s41467-026-69862-7)
Supplement: Supplementary file 1 — Supplementary Information.pdf [file 41467_2026_69862_MOESM1_ESM.pdf]

Supplementary Information

High-throughput chiral copper foils by curved-surface confinement recrystallization

Deping Huang[1,2,#], Zhancheng Li[1,2,#] , Yinwu Duan[1,3], Xin li[1,2], Yongna Zhang[1,2], Jiaxing Dong[4], Guilin Wu[5], Xiaoxu Huang[5], Leining Zhang[6,*], Feng Ding[7,*], Haofei Shi[1,2,*]

[1] Chongqing Institute of Green and Intelligent Technology, Chinese Academy of Sciences, Chongqing, 400714, P. R. China.

[2] University of Chinese Academy of Sciences (UCAS), Beijing, 100190, P. R. China.

[3] National Engineering Research Center for Instrument Functional Materials, Chongqing, 400707, P.R. China

[4] School of Chemistry and Chemical Engineering, Southwest University, Beibei, Chongqing, 400715, P. R. China.

[5] International Joint Laboratory for Light Alloys (MOE), College of Materials Science and Engineering, Chongqing University, Chongqing, 400044, P.R. China

[6] Beijing Key Laboratory of Intelligent Molecular Materials and High-throughput Manufacturing, School of Chemistry and Chemical Engineering, Beijing Institute of Technology, Beijing, 100081, P.R. China

[7] Research Division of Advanced Materials, Suzhou Laboratory, Suzhou, 215133, P.R. China

[#] These authors contributed equally: Deping Huang, Zhancheng Li.

*Corresponding author. Email: leiningzhang@bit.edu.cn (L.Z.); dingf@szlab.ac.cn (F. D.); shi@cigit.ac.cn (H.S.)

This PDF file includes:

Supplementary Figures 1 to 18.

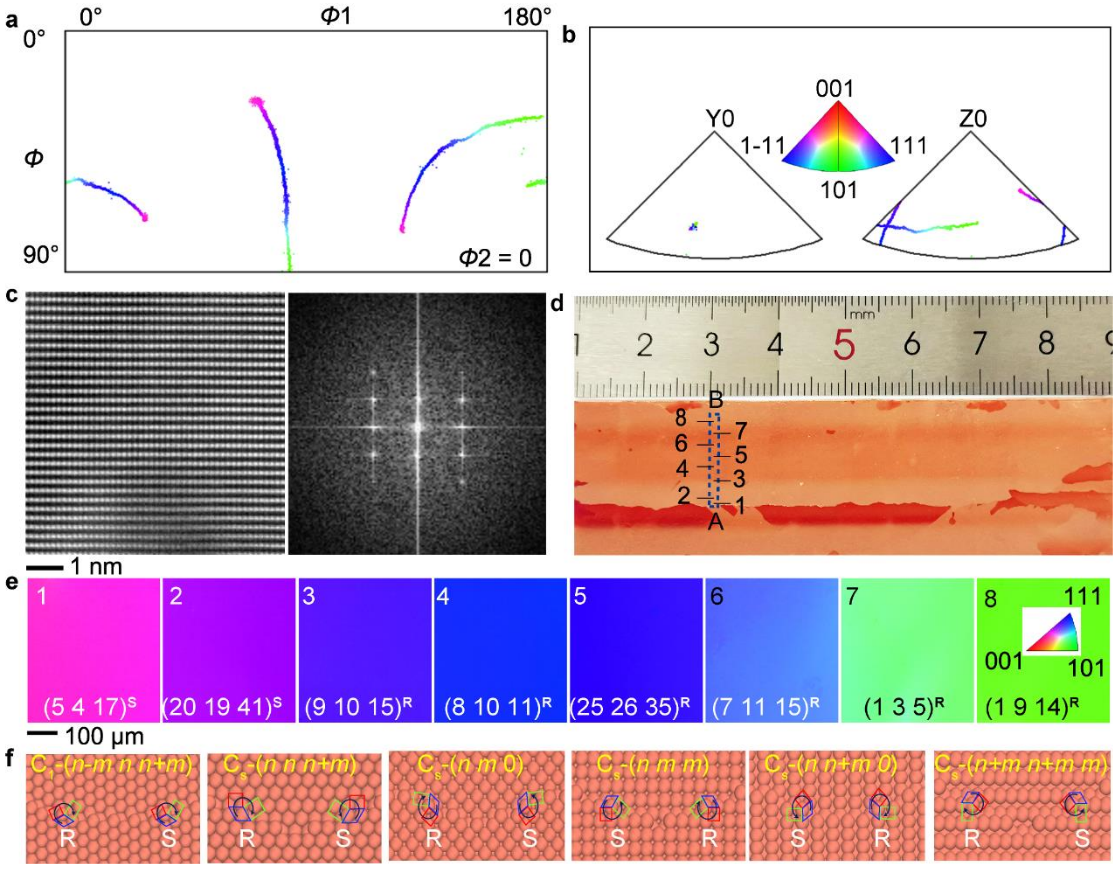

Supplementary Figure 1. Characterization of gradient surface orientation in copper foil. a-b, Corresponding orientation distribution function and unfolded IPF images of EBSD measurement in Figure 1. Y0 represent the transverse direction and Z0 represent the normal direction of the sample. **c**, HRTEM atomic image and Fast Fourier Transform of copper foil tested near Cu(1 1 2) of the gradient substrate. **d**, Optical micrographs of copper foil after confinement annealing marked with position 1-8. **e**, EBSD images of eight regions selected at equal intervals marked in (**d**). **f**, Chirality change of surface orientation with different symmetries. (The (*n*-*m* *n* *n*+*m*), (*n* *n* *n*+*m*), (*n* *m* 0), (*n* *m* *m*), (*n* *n*+*m* 0) and (*n*+*m* *n*+*m* *m*) are Miller indices of surfaces where $m \ll n$. Among them, only (*n* *n* *n*+*m*) is chiral due to its C_1 symmetry, the rest are Cs symmetric and have no chirality. Curvature induction during annealing generates kinks, rendering Cs-symmetric surfaces capable of exhibiting two distinct chirality.)

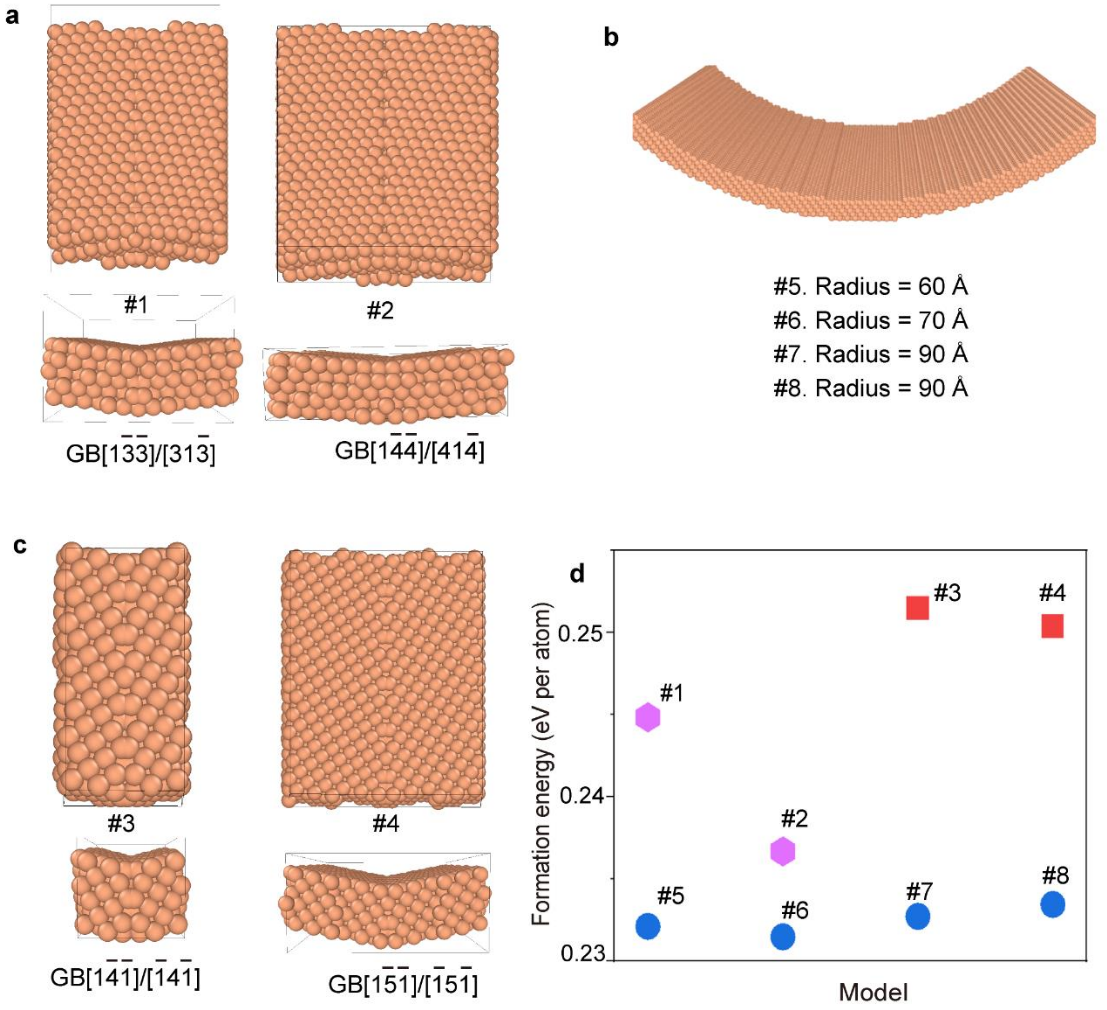

Supplementary Figure 2. Formation energies of arc-shape copper foils with polycrystalline or single-crystal structures. a-b, Atomic structures of arc-shape polycrystals with twin boundaries and low-index surfaces, GB is abbreviation of grain boundary. **c**, Atomic structures of arc-shape single crystals with a graded surface under different curvatures. **d**, Formation energies of different arc-shape copper foils shown in (**a-c**).

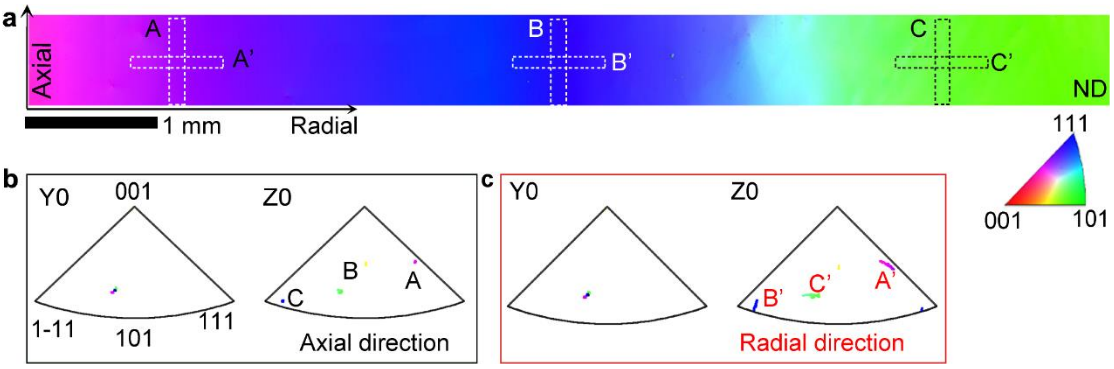

Supplementary Figure 3. Surface orientation distribution along axial and radial direction. **a**, The analyzed areas of surface orientation, ND denotes the normal direction of the copper foil. **b**, Evolution of the surface orientation in unfolded IPF along the axis direction. Y0 represent the transverse direction and Z0 represent the normal direction of the sample. **c**, Evolution of the surface orientation in unfolded IPF along radial direction.

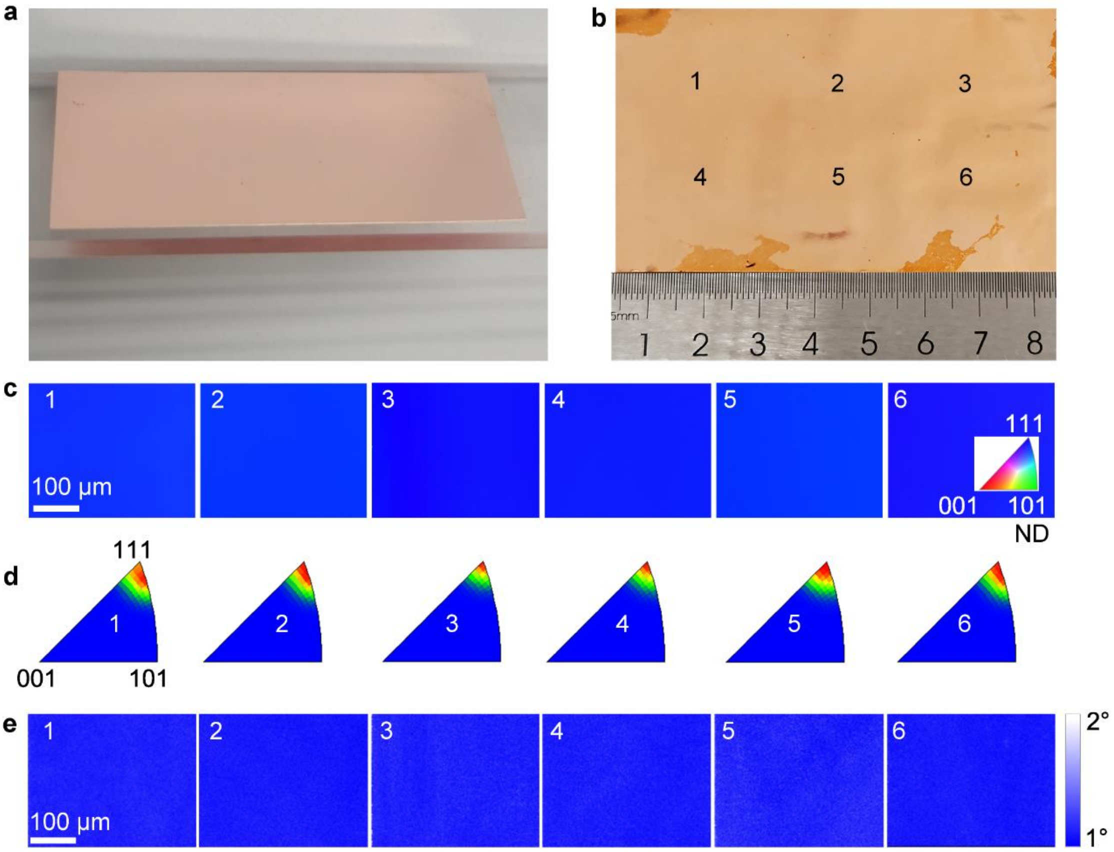

Supplementary Figure 4. The single crystal copper foil obtained by flat-surface annealing. a-b, Photographs of copper foil before and after flat-surface annealing. **c**, EBSD IPF maps, ND denotes the normal direction of the copper foil. **d**, The corresponding IPFs for the normal direction. **e**, The kernel average misorientation maps.

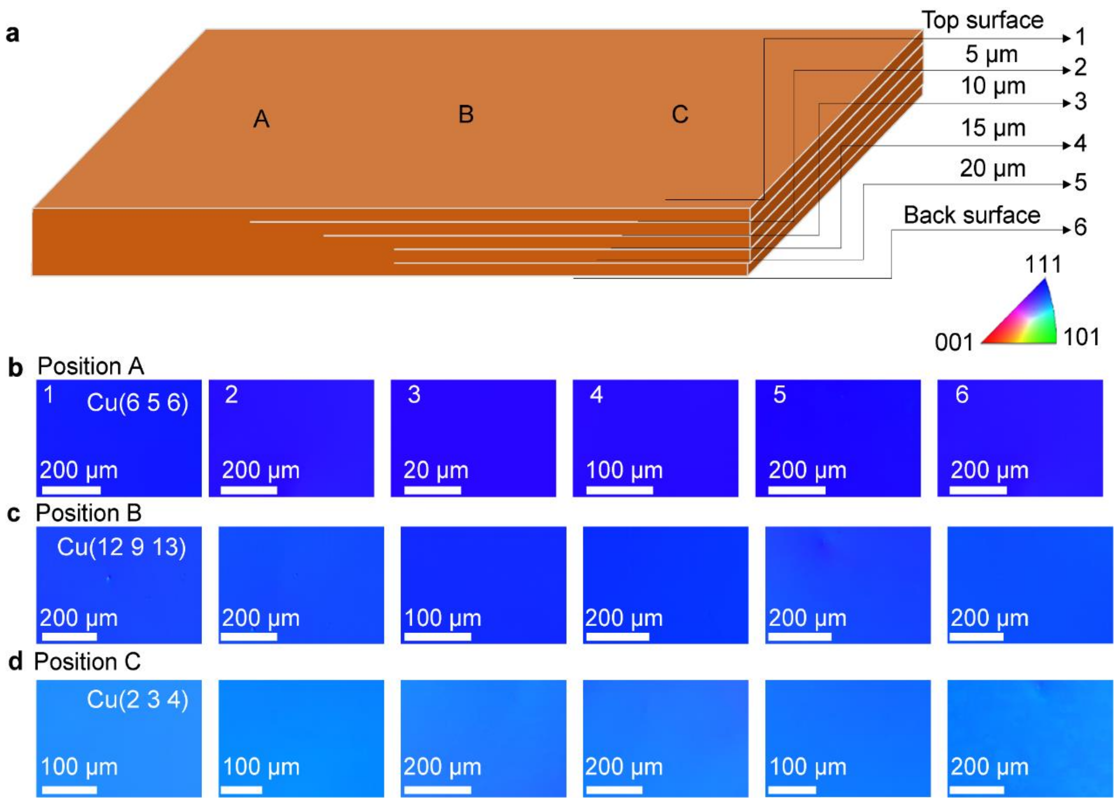

Supplementary Figure 5. The surface orientation of copper foil (25 μm) across thickness direction. **a**, Schematic diagram of surface orientation testing at 5-micrometer intervals across thickness direction. **b-d**, EBSD IPF maps in normal direction at the position A, B and C marked in (**a**). The scale bars are 200 μm.

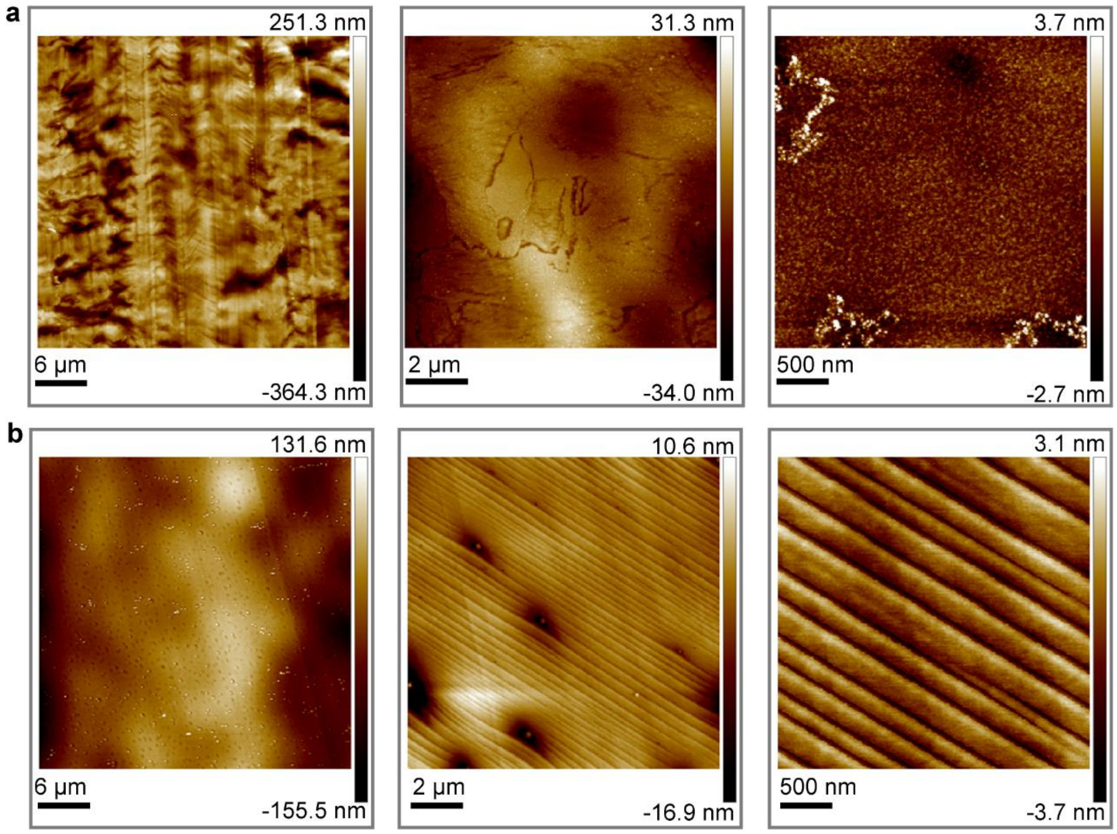

Supplementary Figure 6. AFM characterization. a, AFM images of copper foils before annealing. **b**, AFM images of copper foils after annealing. The raw data are available in Supplementary Data 1.

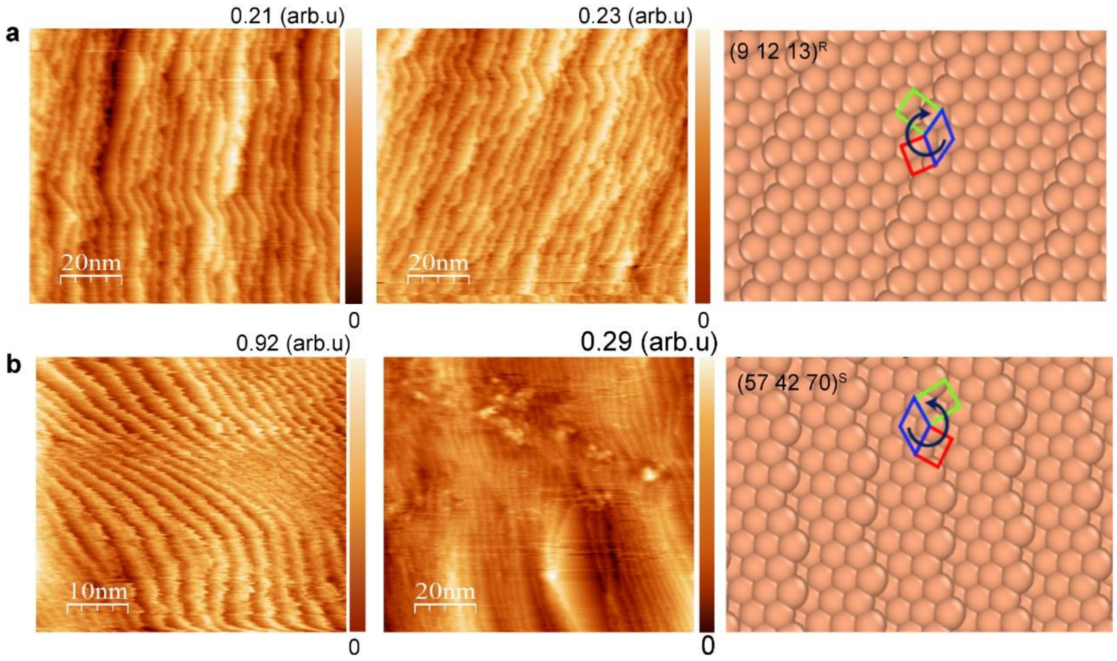

Supplementary Figure 7. STM characterization of chiral copper surfaces. a-b, STM topography images of the copper surface, exhibiting atomic-scale chiral reconstructions. The corresponding atomic structural models identifying the *R*- (**a**) and *S*-chirality (**b**). The color intensity in STM images is given in arbitrary units (arb. u.). The raw data are available in Supplementary Data 1.

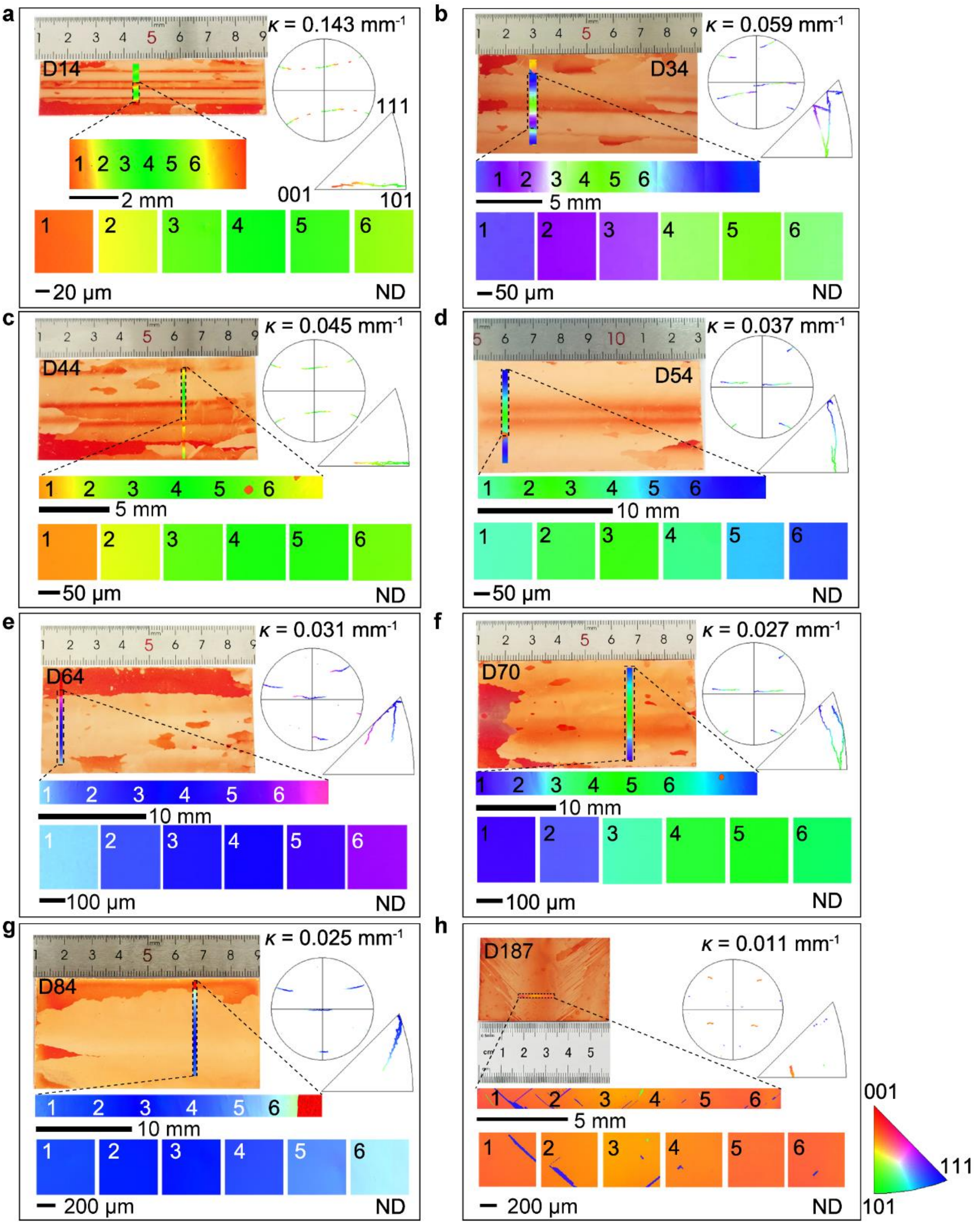

Supplementary Figure 8. Photographs, EBSD results of the copper foil annealed in quartz tubes with different diameters. a, 14 mm. **b**, 34mm. **c**, 44 mm. **d**, 54 mm. **e**, 64mm. **f**, 70 mm. **g**, 84 mm. **h**, 187 mm. The pole figures are presented for the {111} plane and IPFs presented for ND. ND denotes the normal direction of the copper foil.

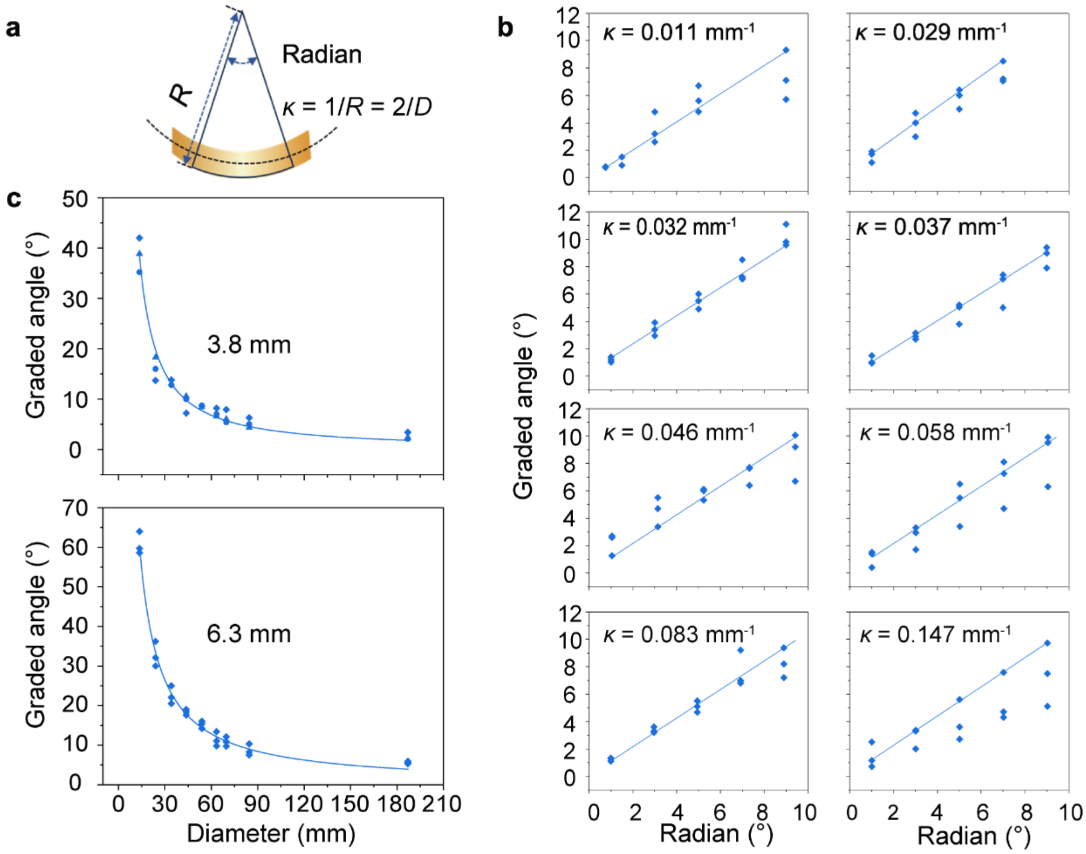

Supplementary Figure 9. The relationship between curvature and graded angle of copper foils. **a**, Schematic illustration of the experimental geometry, defining the substrate curvature (κ), the radius of curvature (r), and the diameter (D) of the quartz tube confinement. **b**, Relationship between the graded angle and radian for copper foils annealed under different curvatures (κ). Individual data points represent results from three independent annealing experiments (n = 3), compared to flat-surface annealing controls. All datasets are excellently described by linear regressions. The overall relationship is highly significant (ANOVA, $p < 0.0001$), with slopes around 1.0, confirming a direct proportional relationship. **c**, Relationship between the graded angle and substrate curvature for arc lengths of 3.8 and 6.3 mm. The data are accurately described by an allometric model, as evidenced by the high goodness-of-fit (adjusted $R^2 = 0.97$, ANOVA $p = 1.98 \times 10^{-23}$ for 3.8 mm and $R^2 = 0.99$, $p = 2.12 \times 10^{-30}$ for 6.3 mm, respectively). Source data are provided in Source Data file.

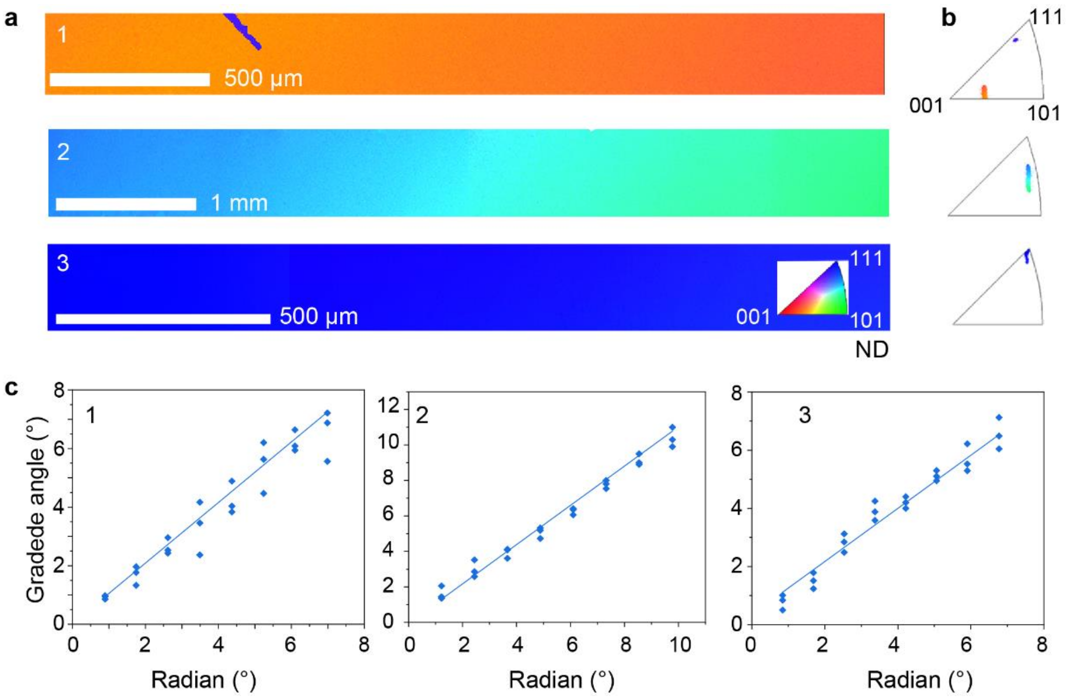

Supplementary Figure 10. Reproducibility of annealing on multiple copper foils under identical curvature (*κ* = 0.029 cm^{-1}). a-b, The EBSD results of the annealed copper foils. ND denotes the sample normal direction. **c**, Linear relationship between graded angle and radian, confirmed across three independent annealing experiments (*n* = 3). Each replicate is presented in a separate plot. Individual data points represent the mean values from 3 analyzed regions for each sample. Regression parameters are: Replicate 1: slope = 1.03 ± 0.07 (R^2 = 0.97); Replicate 2: slope = 1.11 ± 0.05 (R^2 = 0.99); Replicate 3: slope = 0.91 ± 0.05 (R^2 = 0.99). Source data are provided as a Source Data file.

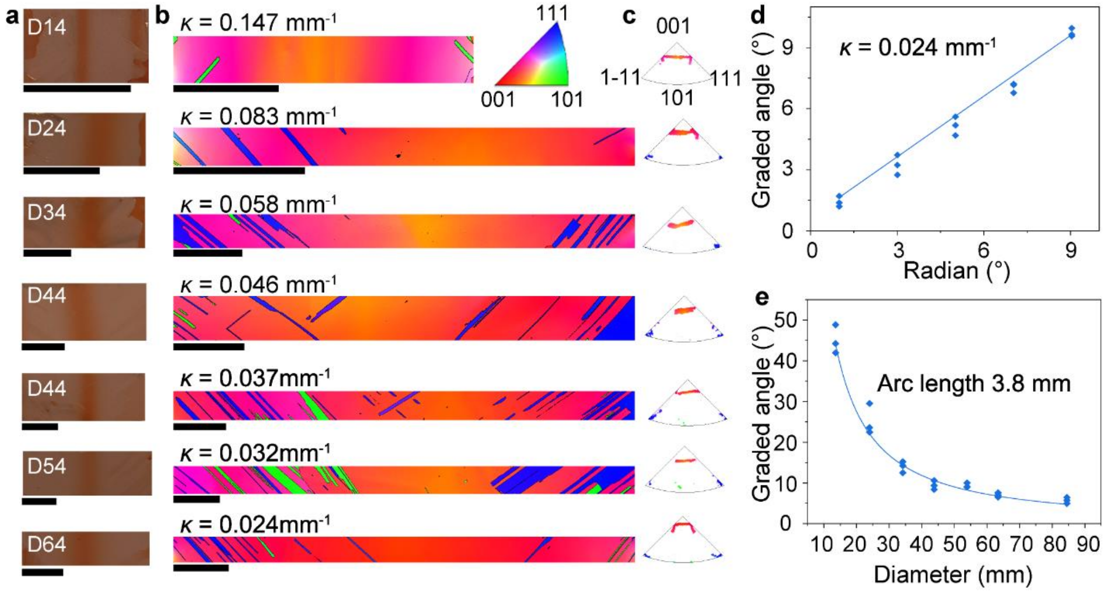

Supplementary Figure 11. Copper foils with a thickness of 46 μm annealed under different curvatures (14-84 mm). a, Photographs of annealed copper foils. Scale bar: 5 mm. **b**, EBSD IPF maps for normal direction. Scale bar: 2 mm in **(b)**. **c**, Unfolded IPFs for normal direction. **d**, Linear relationship between graded angle and radian, derived from one experiment. Individual data points are mean values from three analyzed regions ($n = 3$), fitted by a linear model (slope = 0.99 ± 0.05; adjusted $R^2 = 0.99$). **e**, Relationship between the graded angle and quartz tube diameter. Data are from seven independent annealing experiments (one biological replicate per tube diameter condition). For each condition, the data point represents the mean value from three analyzed regions ($n = 3$). The data are described by an allometric model (adjusted R^2 of 0.99). Source data are provided in the Source Data file.

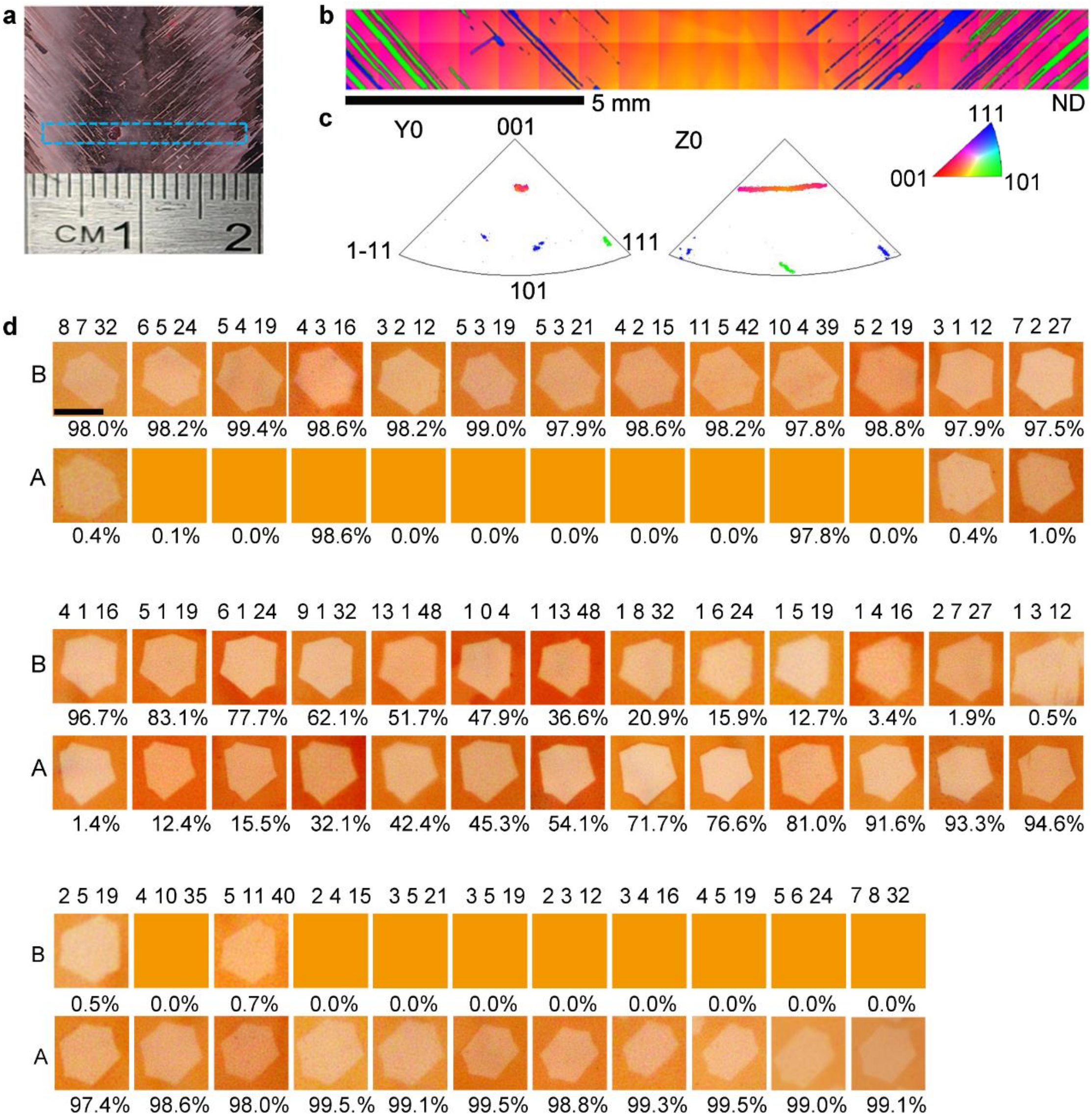

Supplementary Figure 12. Graphene epitaxy on gradient copper substrate. a, Photograph of the gradient copper foil for graphene epitaxy, parallel line patterns are twin grain boundaries. **b-c,** EBSD measurement results of the gradient copper foil. Y0 represent the transverse direction and Z0 represent the normal direction of the sample. **d,** Representative graphene grains and percentage of grains A and B from (8 7 32) to (7 8 32) with interval of 325 μm, scale bar:10 μm. Unnucleated areas are marked by blank boxes. Source data are provided in Source Data file.

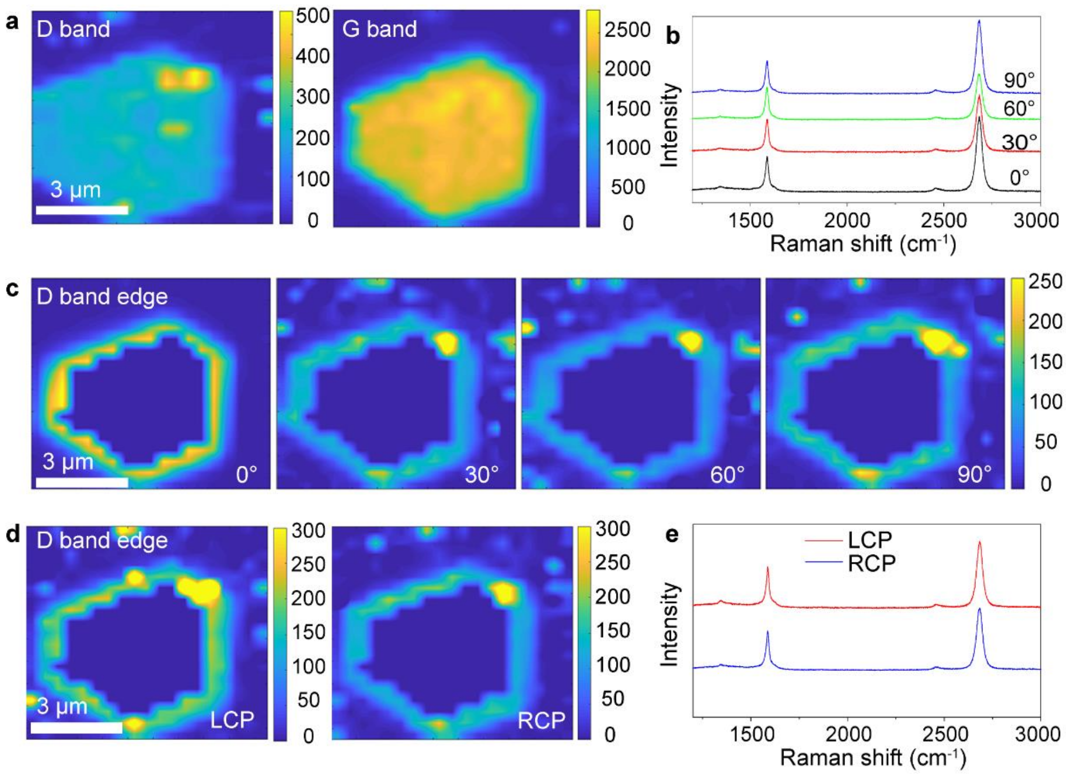

Supplementary Figure 13. Angle-Resolved Raman Spectroscopy of graphene grains with polarized light. a, D band and G band Raman mapping of the graphene grain without polarization. **b**, Typical Raman spectrum under 0-90 ° polarized light of edge area of graphene grain. **c**, D band mapping of edge area under 0-90° polarized light. **d**, D band mapping of edge area under LCP and RCP light. **e**, Typical Raman spectrum under LCP and RCP light. The color intensity corresponds to the relative Raman scattering intensity (in arbitrary units, arb. u.).

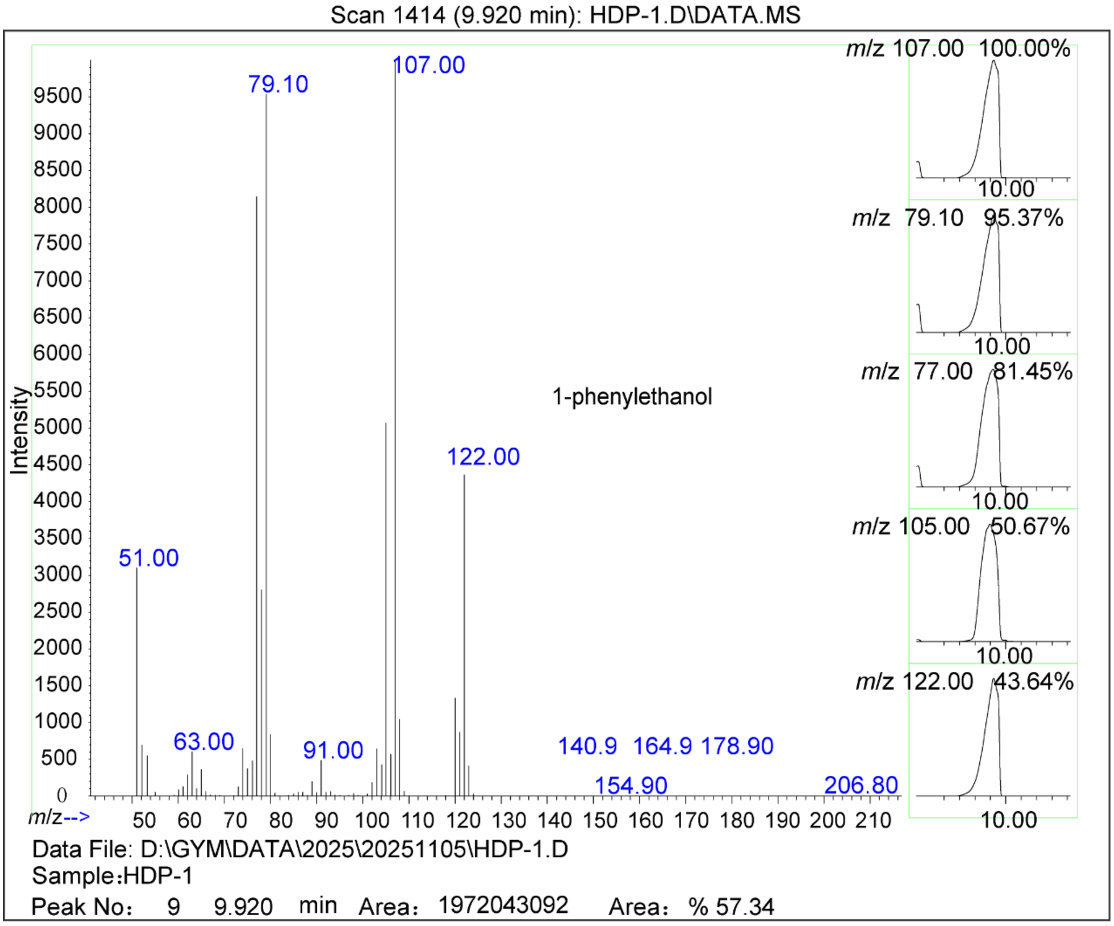

Supplementary Figure 14. GC-MS spectrum of the residual 1-phenylethanol from the catalytic reaction. The results confirm that unreacted starting material remains, which can be used to analyze the enantioselectivity of the reaction process. The raw data are available in Supplementary Data 1.

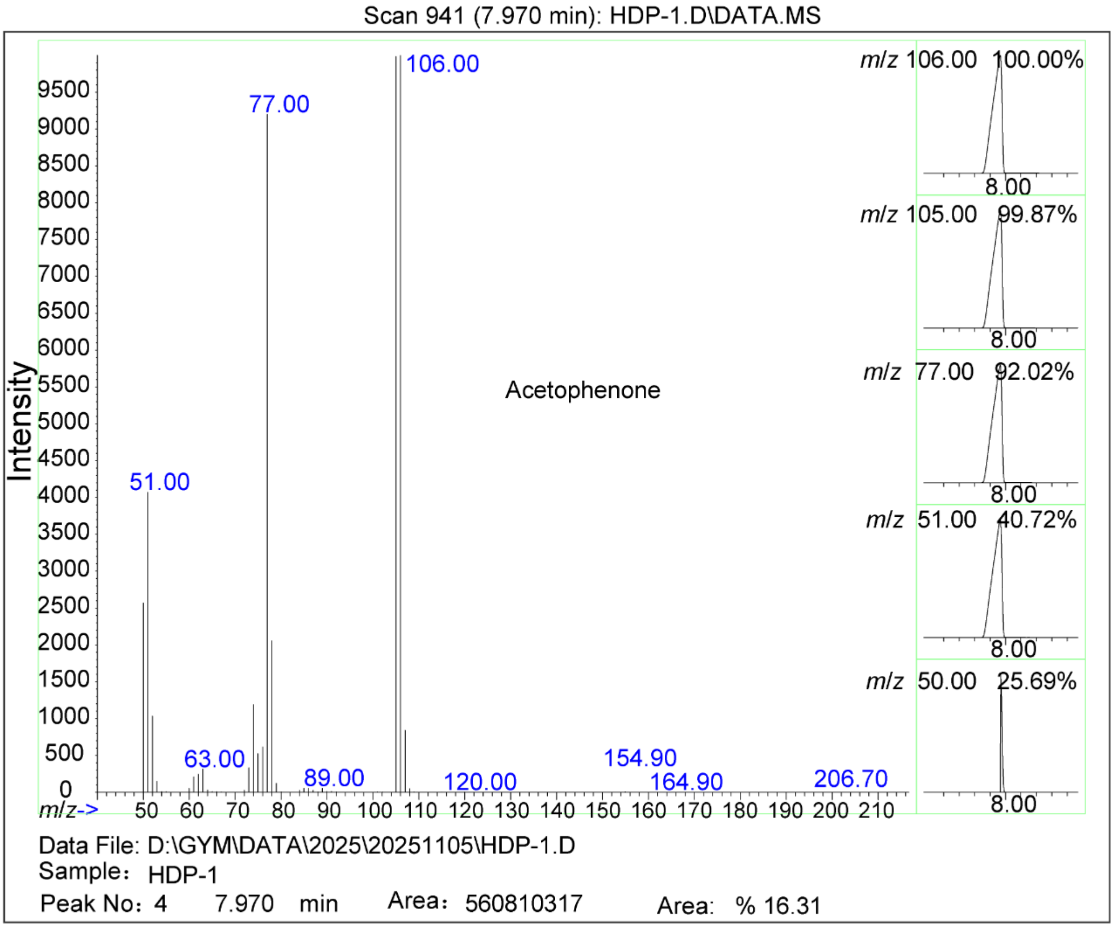

Supplementary Figure 15. GC-MS spectrum of reaction product acetophenone. The results confirm the successful progress of the asymmetric catalytic reaction on the chiral copper surface. The raw data are available in Supplementary Data 1.

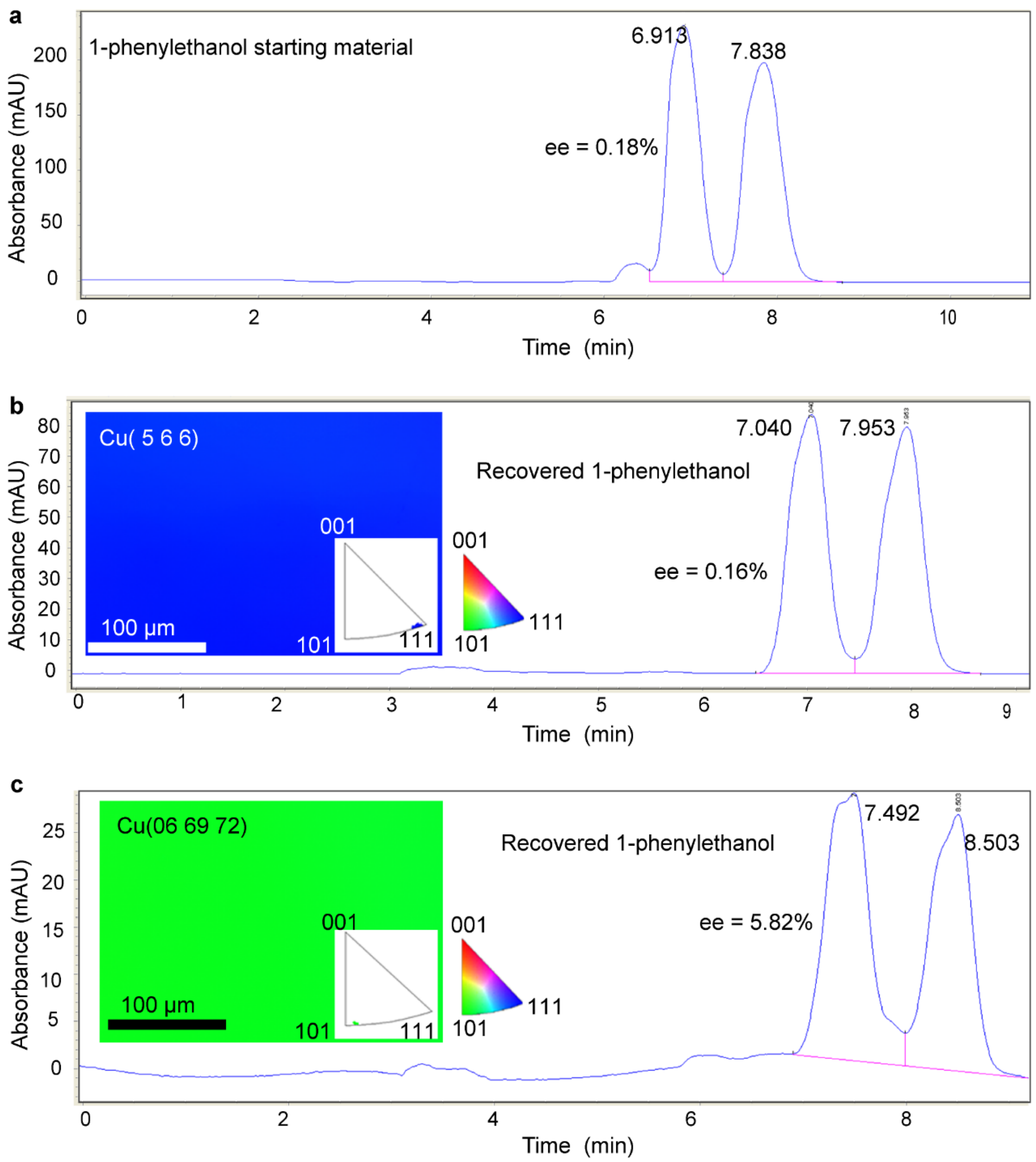

Supplementary Figure 16. Validation of enantioselective catalysis through chiral HPLC analysis. **a**, Chiral HPLC chromatogram of the 1-phenylethanol starting material (racemic mixture). **b**, Chiral HPLC chromatogram of recovered 1-phenylethanol from the control reaction using an achiral Cu(5 6 6) as catalyst. **c**, Chiral HPLC chromatogram of recovered 1-phenylethanol from the reaction catalyzed by the chiral Cu(06 69 72). The shows the retention time in minutes (min), and the y-axis shows the absorbance in milli-absorbance units (mAU). The raw data are available in Supplementary Data 1.

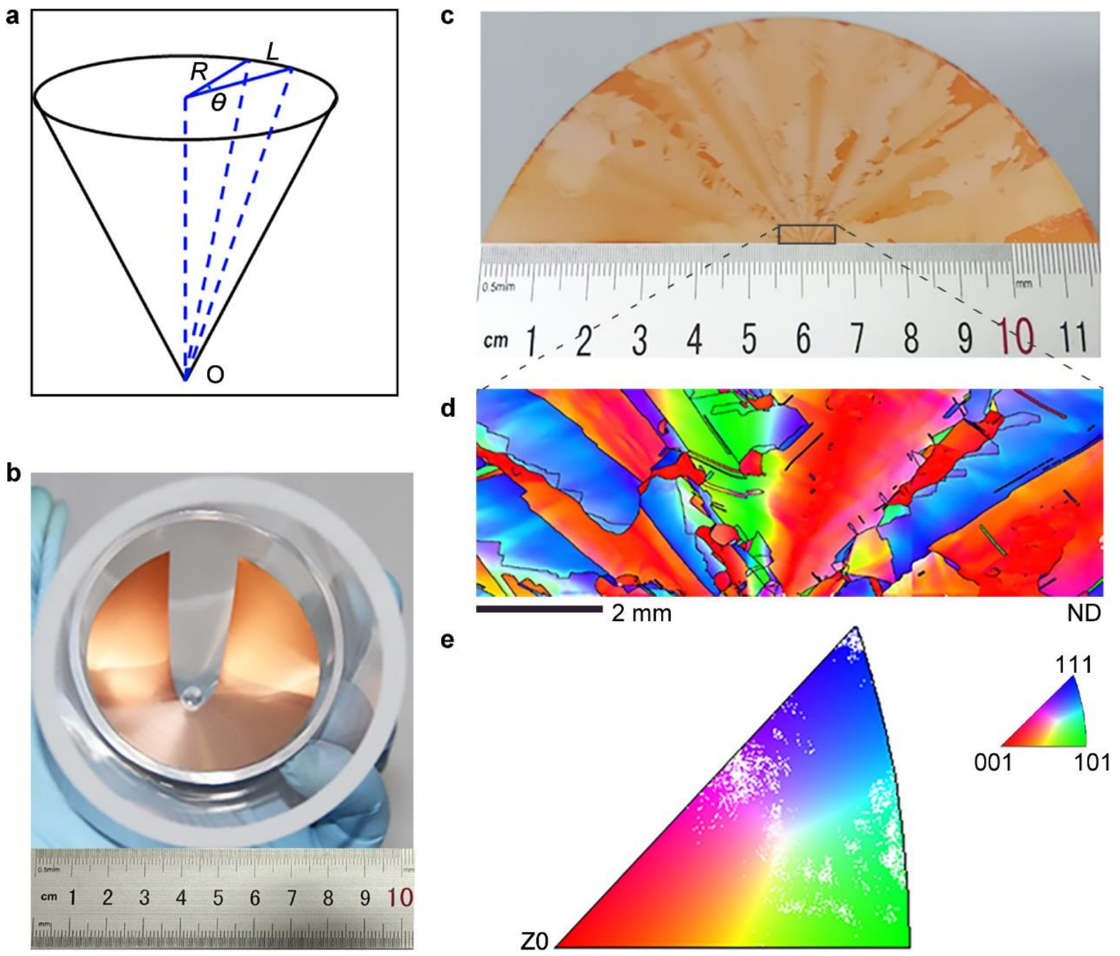

Supplementary Figure 17. Copper foils annealed with quartz cone. a-b, Schematic diagram and photograph of quartz cone confinement annealing. **c**, Photograph of annealed copper foil. **d**, The EBSD IPF map of the annealed copper foil at the conic node, ND denotes the normal direction of the copper foil. **e**, IPF corresponding to the EBSD map in (**d**).

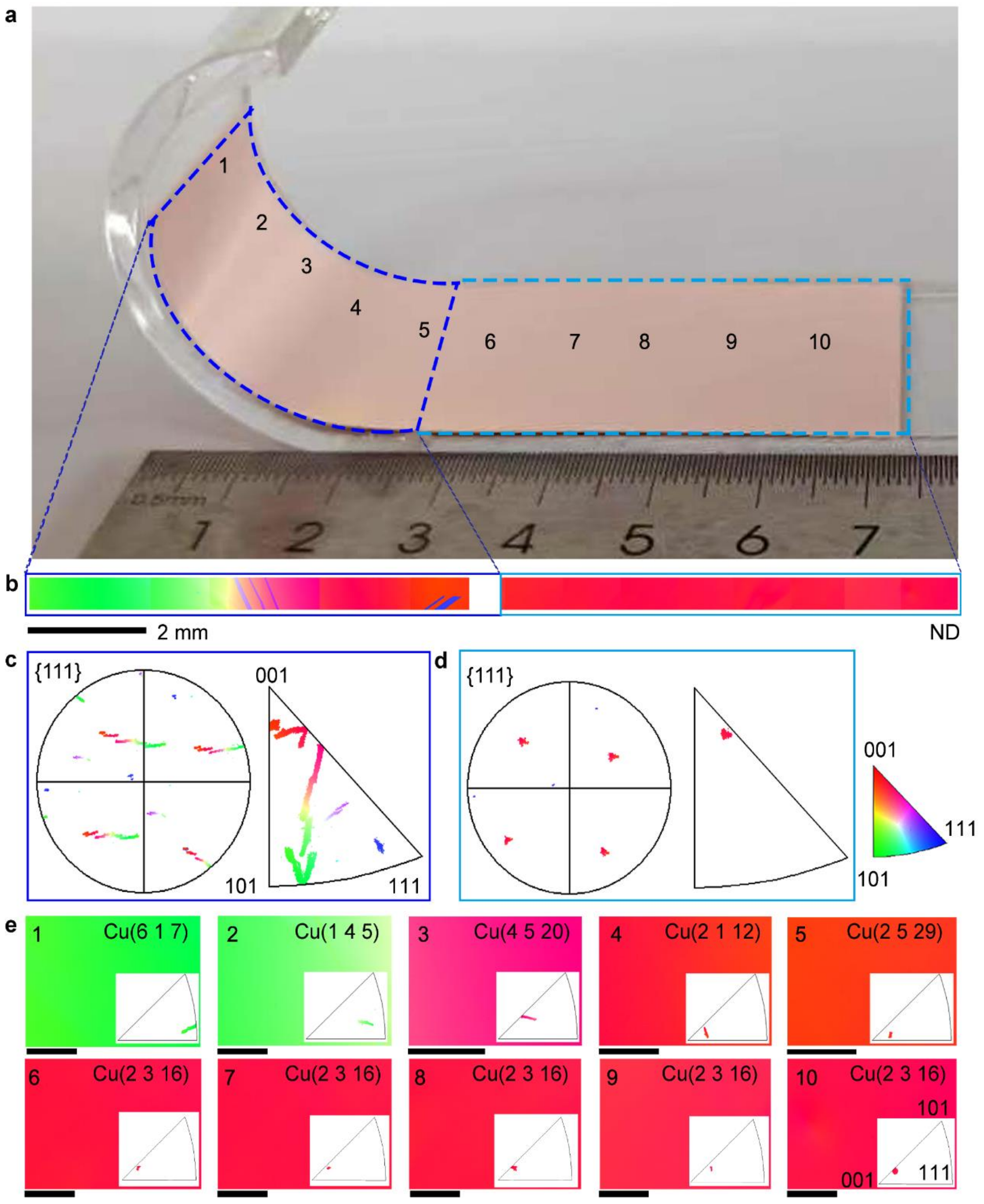

Supplementary Figure 18. Controllable synthesis of graded copper surface by seeded growth. **a**, Photograph of the copper foil by surface orientation tailoring annealing. **b-e**, The EBSD characterization of the obtained copper foil, indicating the surface indexes varies across the curved region (**c**) and keep constant in the flat region (**d**). ND denotes the normal direction of the copper foil. The scale bars: 500 µm.
