# Supplementary material for: High-throughput chiral copper foils by curved-surface confinement recrystallization
Source: Nat Commun. 2026 Feb 20;17:2796. doi: 10.1038/s41467-026-69862-7 (PMC13022495; doi:10.1038/s41467-026-69862-7)
Supplement: Supplementary file 2 — Description of Additional Supplementary Files [file 41467_2026_69862_MOESM2_ESM.pdf]

Description of Additional Supplementary Files

File Name: Supplementary Data 1

Description: These data are the raw data for Figure 3, 4 and Supplementary Figs.1, 6, 7,14, 15, 16.
