# Supplementary material for: High-throughput chiral copper foils by curved-surface confinement recrystallization
Source: Nat Commun. 2026 Feb 20;17:2796. doi: 10.1038/s41467-026-69862-7 (PMC13022495; doi:10.1038/s41467-026-69862-7)
Supplement: Supplementary file 3 — Supplementary Dataset 1 [file 41467_2026_69862_MOESM3_ESM.zip › Supplementary Data 1/Supplementary Fig.14-16/GC-MS Cu(06 69 72).pdf]

谱库检索报告

数据路径 : D:\GYM\DATA\2025\20251105\
数据文件 : HDP-2.D
采集 : 05 Nov 2025 17:09
操作者 : zky-HP\zky
样品 : HDP-2
其他 :
ALS 样品瓶: 4 样品乘积因子: 1

检索库： C:\database\DEMO.L 最小匹配度： 0

未知谱图： 顶点
积分事件： 化学工作站积分器 - events14.e

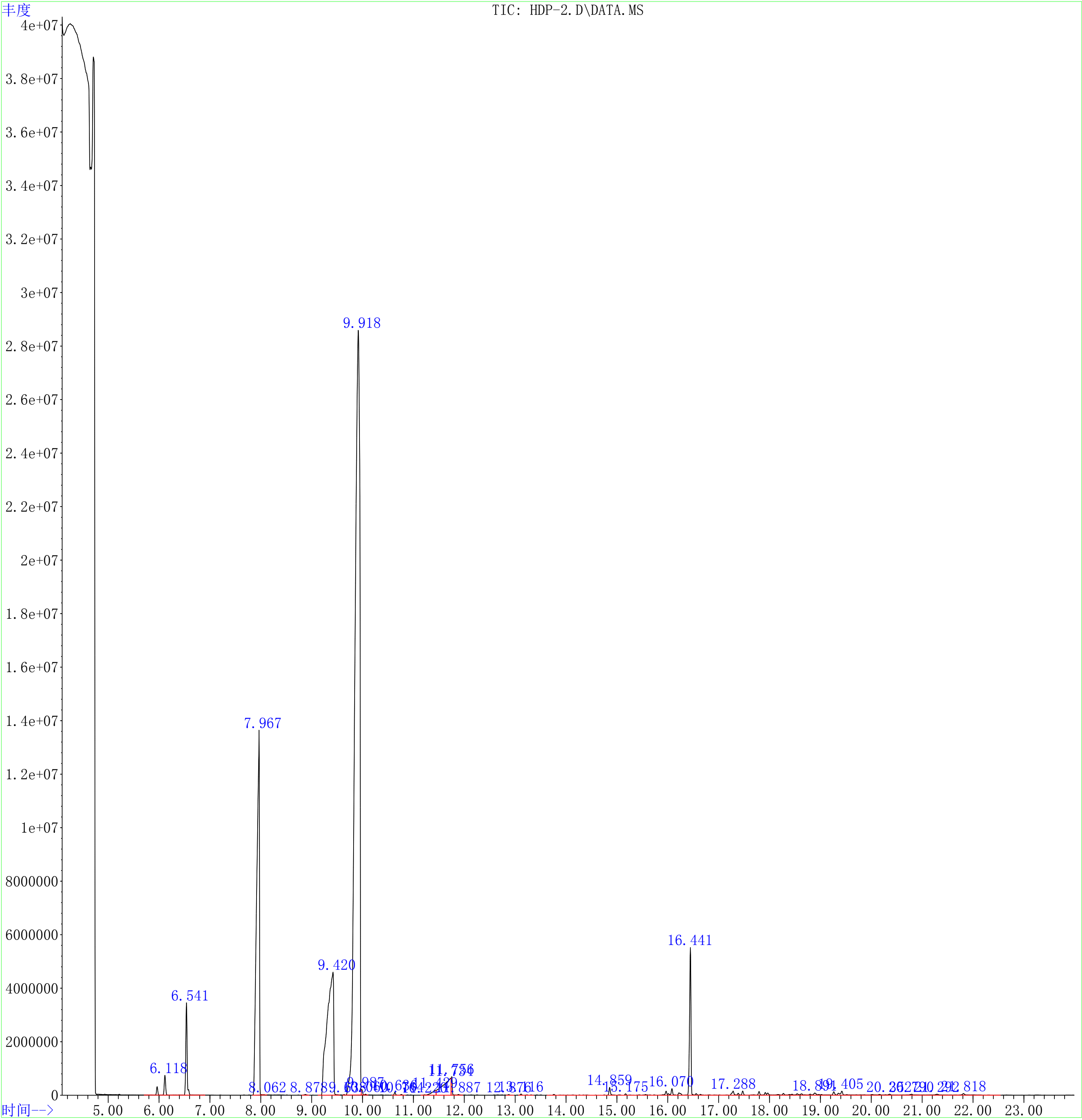

未知谱图基于顶点

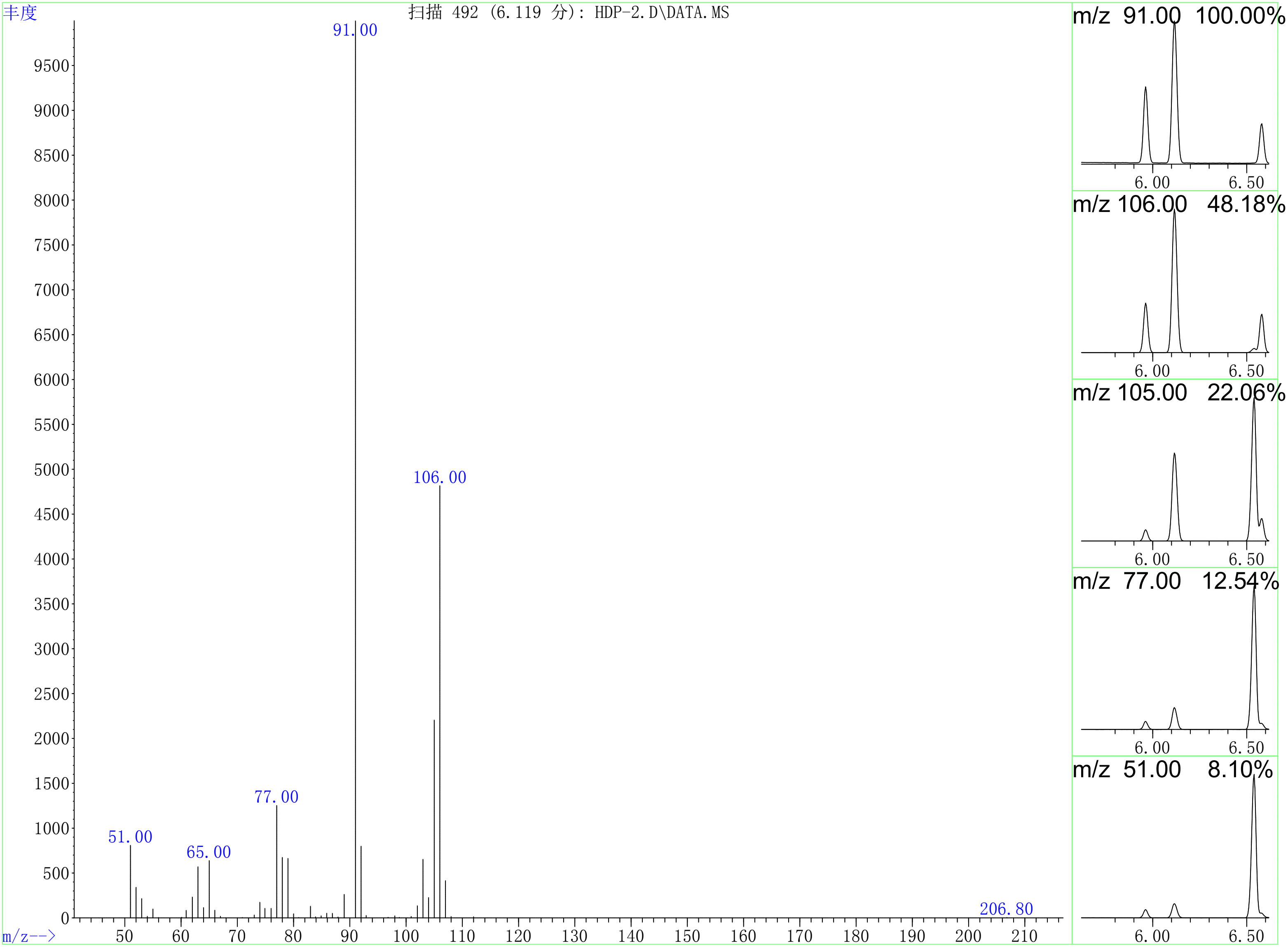

Data File: D:\GYM\DATA\2025\20251105\HDP-2.D
样品：HDP-2

峰编号： 1 6.119 分钟处 面积： 19153220 面积 % 0.60

每个谱库中 3 个最匹配的记录。 Ref\# CAS\# 匹配度

C:\database\DEMO.L 未检索到匹配。

未知谱图基于顶点

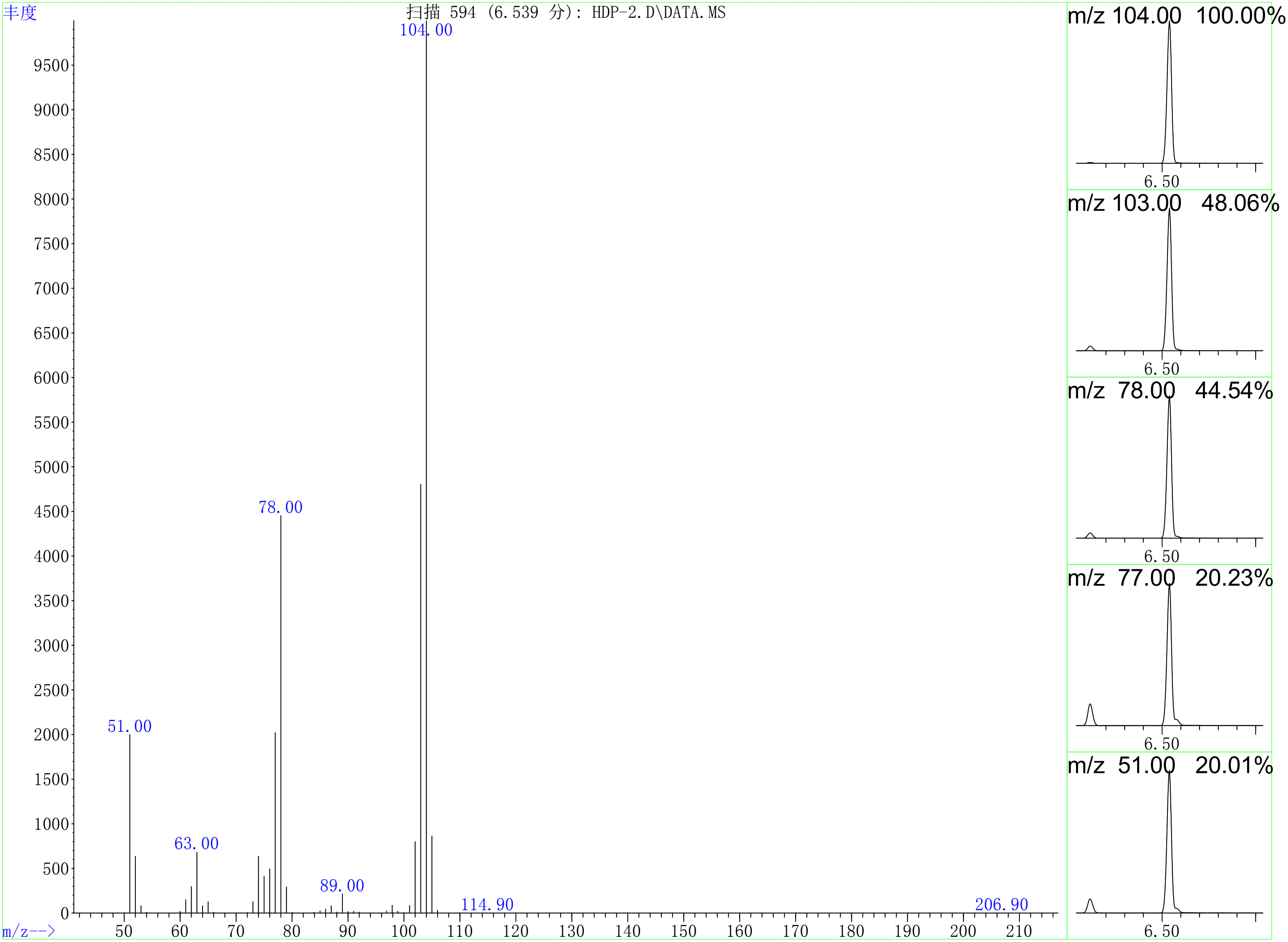

Data File: D:\GYM\DATA\2025\20251105\HDP-2.D
样品：HDP-2

峰编号： 2 6.539 分钟处 面积： 62701960 面积 % 1.96

每个谱库中 3 个最匹配的记录。 Ref\# CAS\# 匹配度

C:\database\DEMO.L 未检索到匹配。

未知谱图基于顶点

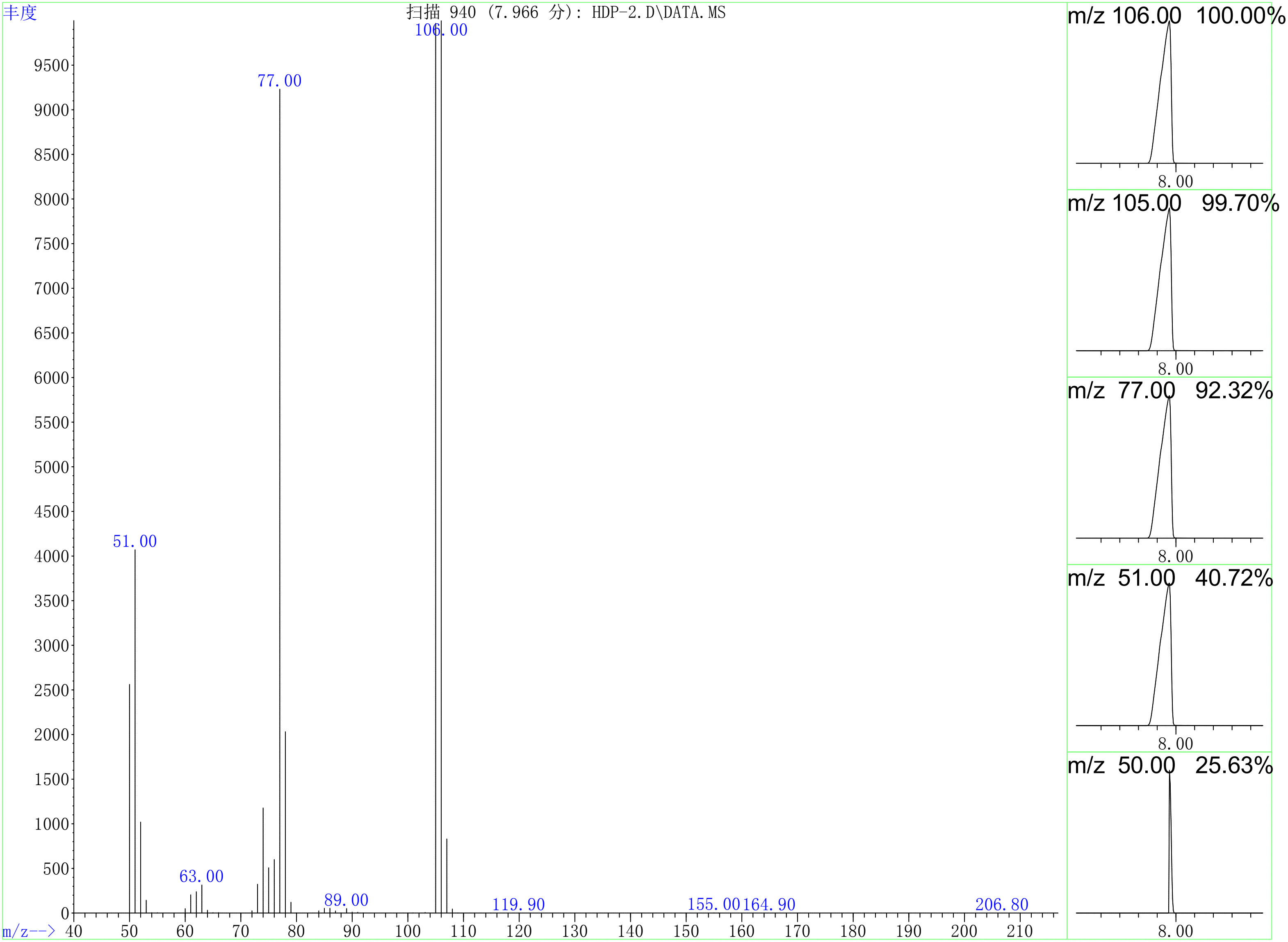

Data File: D:\GYM\DATA\2025\20251105\HDP-2.D
样品：HDP-2

峰编号： 3 7.966 分钟处 面积：511887916 面积 % 15.99

每个谱库中 3 个最匹配的记录。 Ref\# CAS\# 匹配度

C:\database\DEMO.L 未检索到匹配。

未知谱图基于顶点

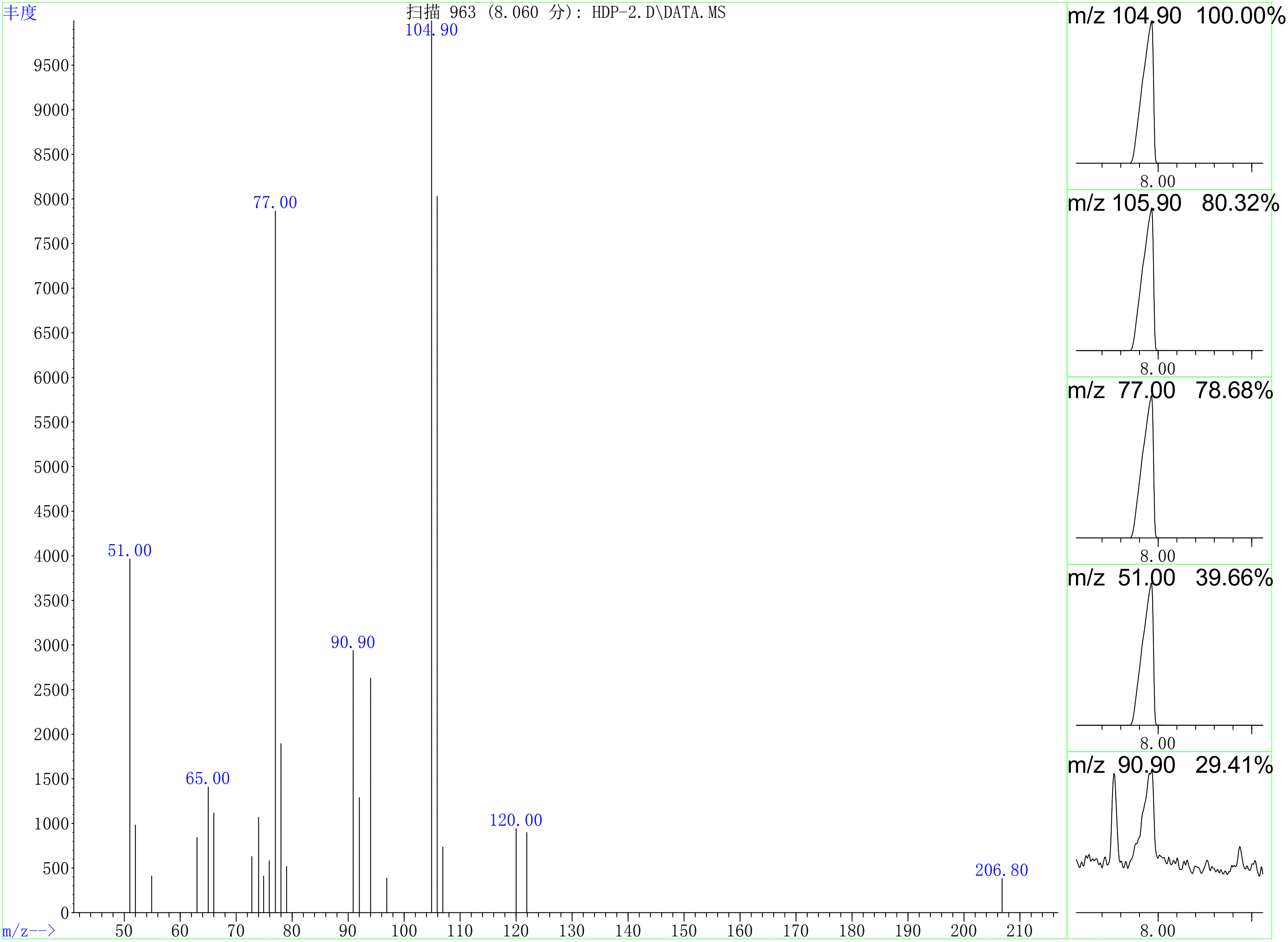

Data File: D:\GYM\DATA\2025\20251105\HDP-2.D
样品：HDP-2

峰编号：4　　8.060 分钟处　面积：　255083　面积 % 0.01

每个谱库中 3 个最匹配的记录。　　Ref\#　CAS\#　匹配度

C:\database\DEMO.L　未检索到匹配。

未知谱图基于顶点

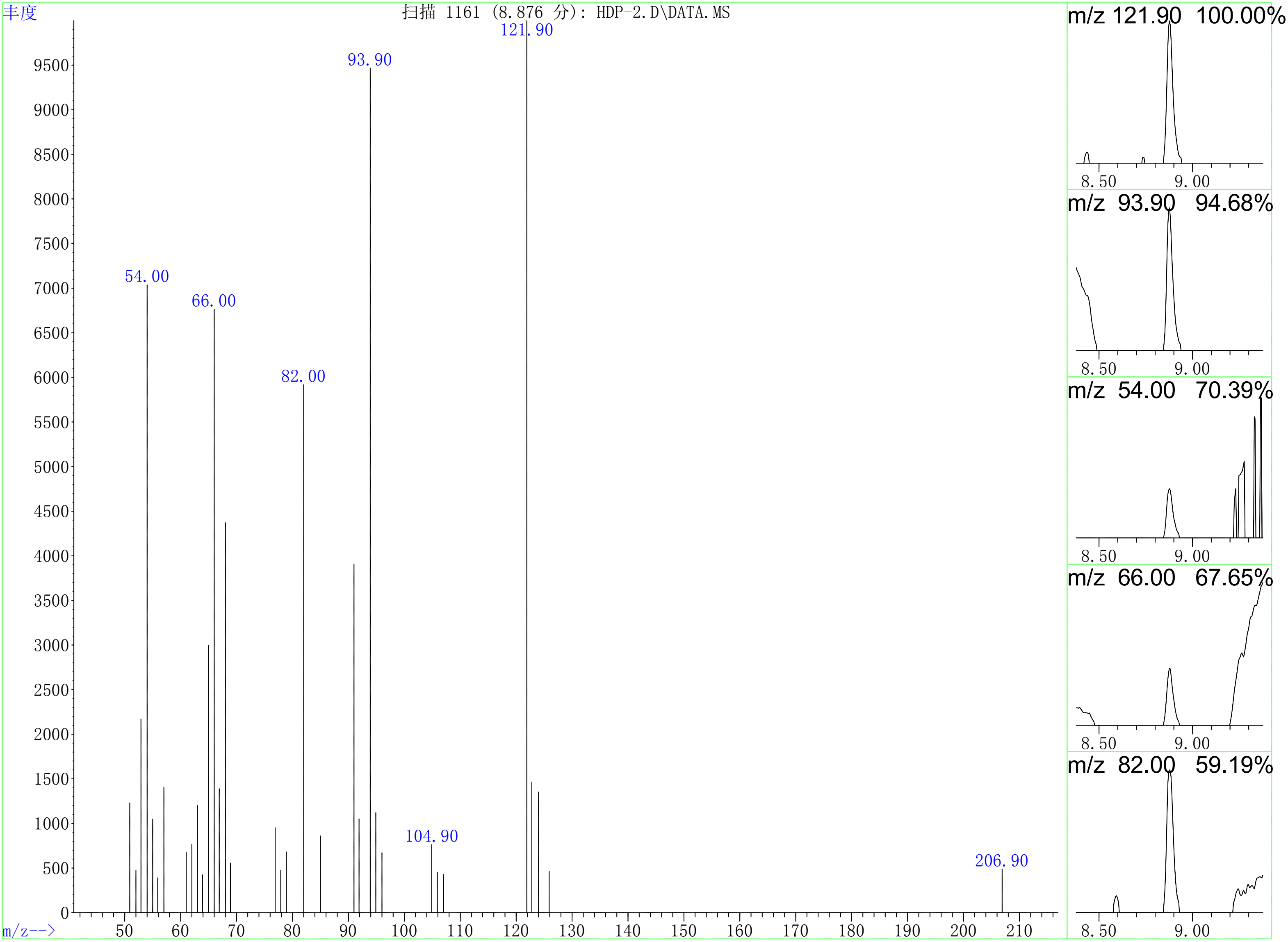

Data File: D:\GYM\DATA\2025\20251105\HDP-2.D
样品：HDP-2

峰编号： 5 8.876 分钟处 面积： 674775 面积 % 0.02

每个谱库中 3 个最匹配的记录。 Ref\# CAS\# 匹配度

C:\database\DEMO.L 未检索到匹配。

未知谱图基于顶点

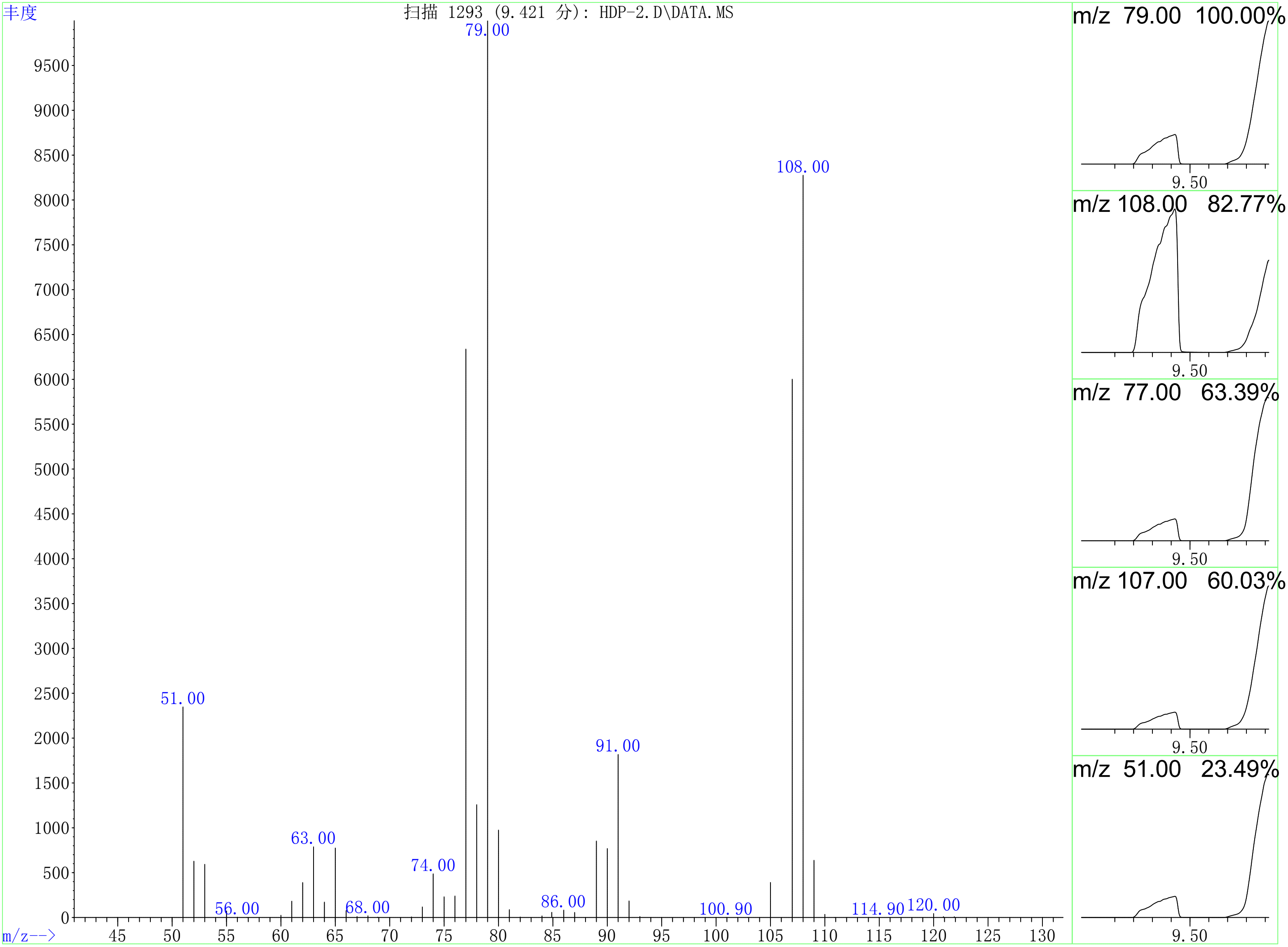

Data File: D:\GYM\DATA\2025\20251105\HDP-2.D
样品：HDP-2

峰编号： 6 9.421 分钟处 面积：412819308 面积 % 12.89

每个谱库中 3 个最匹配的记录。 Ref\# CAS\# 匹配度

C:\database\DEMO.L 未检索到匹配。

未知谱图基于顶点

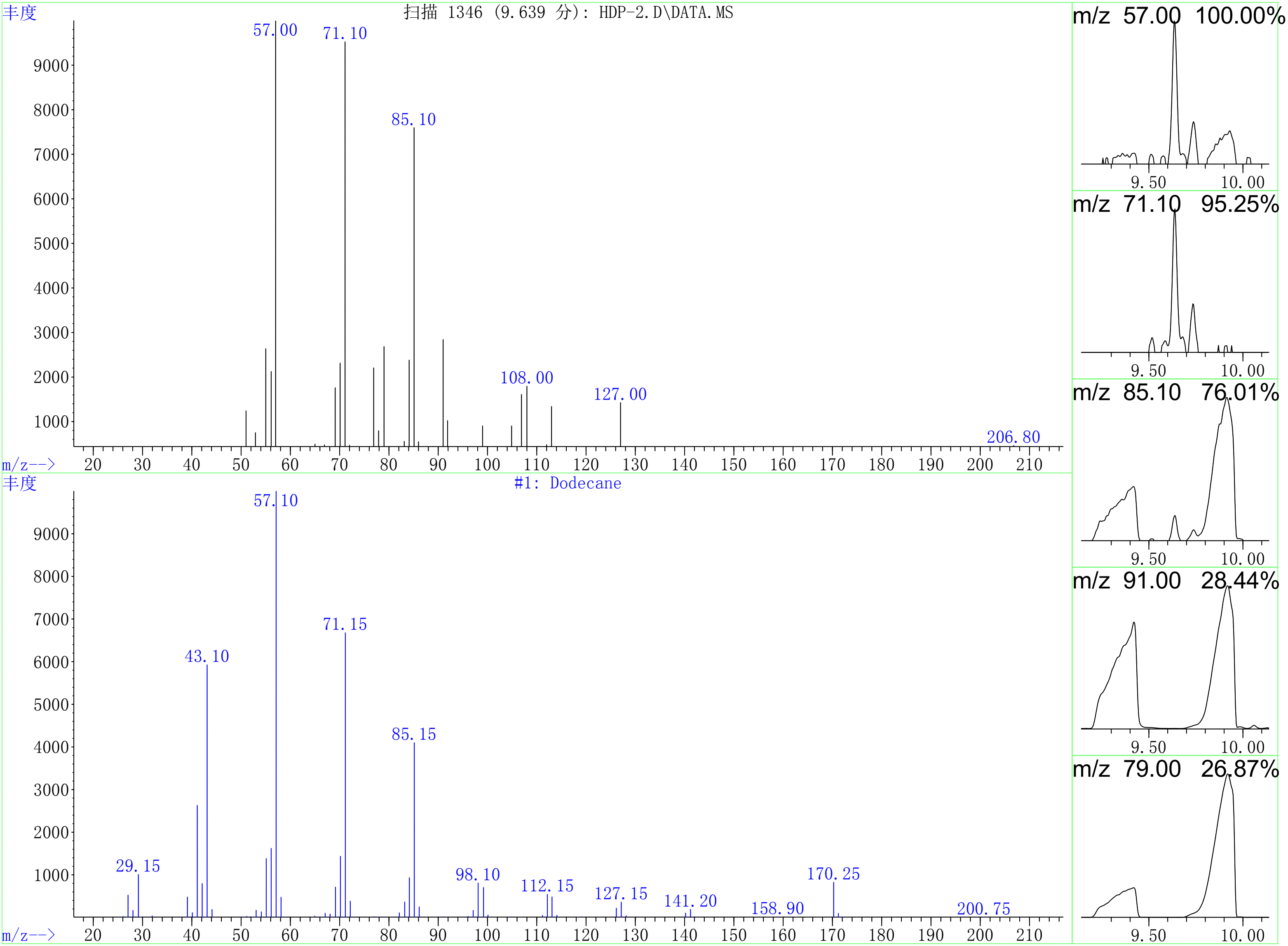

Data File: D:\GYM\DATA\2025\20251105\HDP-2.D
样品：HDP-2

峰编号： 7 9.639 分钟处 面积： 286188 面积 % 0.01

每个谱库中 3 个最匹配的记录。	Ref\#	CAS\#	匹配度
C:\database\DEMO.L			
1 Dodecane	1	000112-40-3	9

未知谱图基于顶点

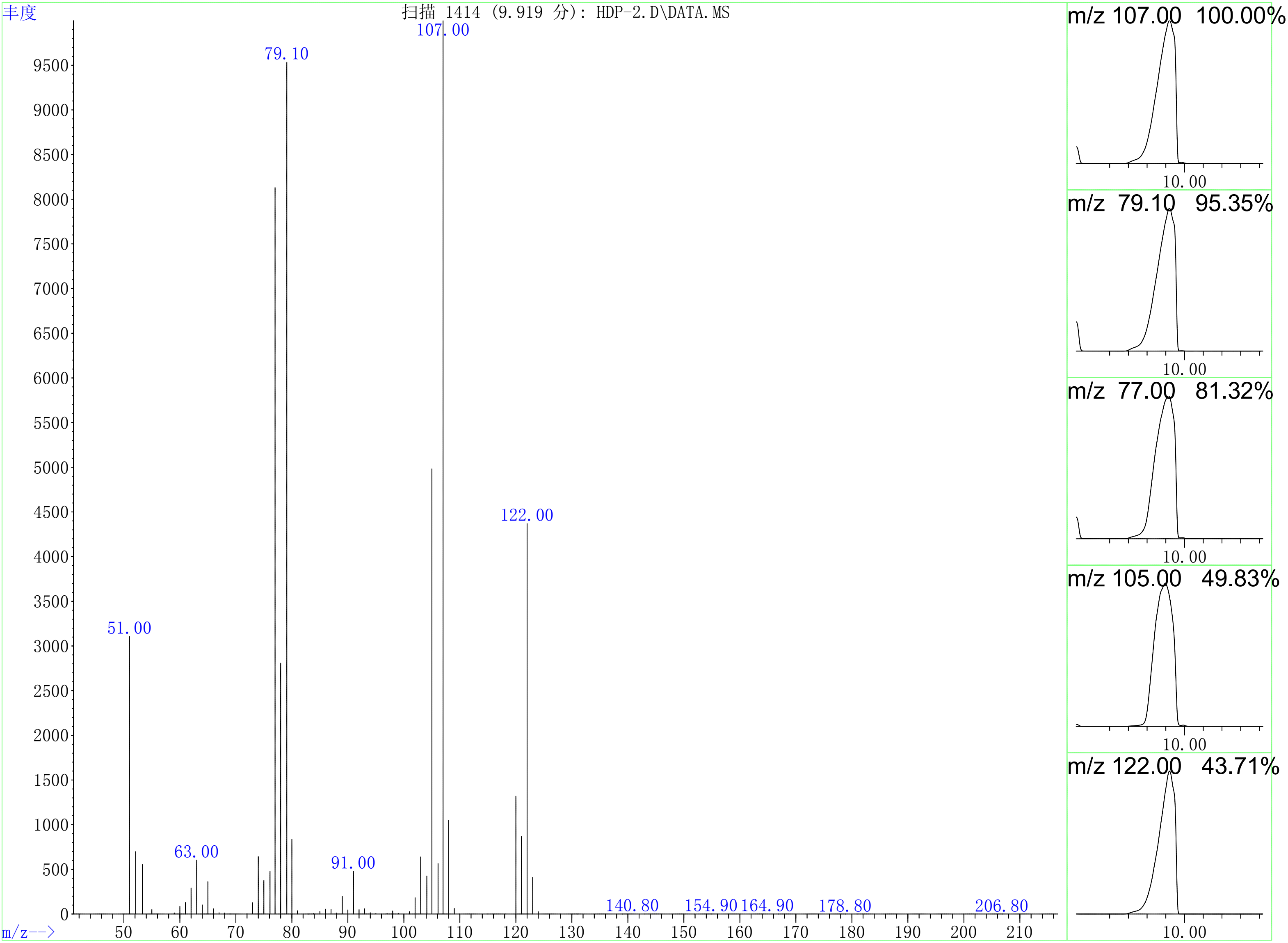

Data File: D:\GYM\DATA\2025\20251105\HDP-2.D
样品：HDP-2

峰编号： 8 9.919 分钟处 面积：1910806958 面积 % 59.68

每个谱库中 3 个最匹配的记录。 Ref\# CAS\# 匹配度

C:\database\DEMO.L 未检索到匹配。

未知谱图基于顶点

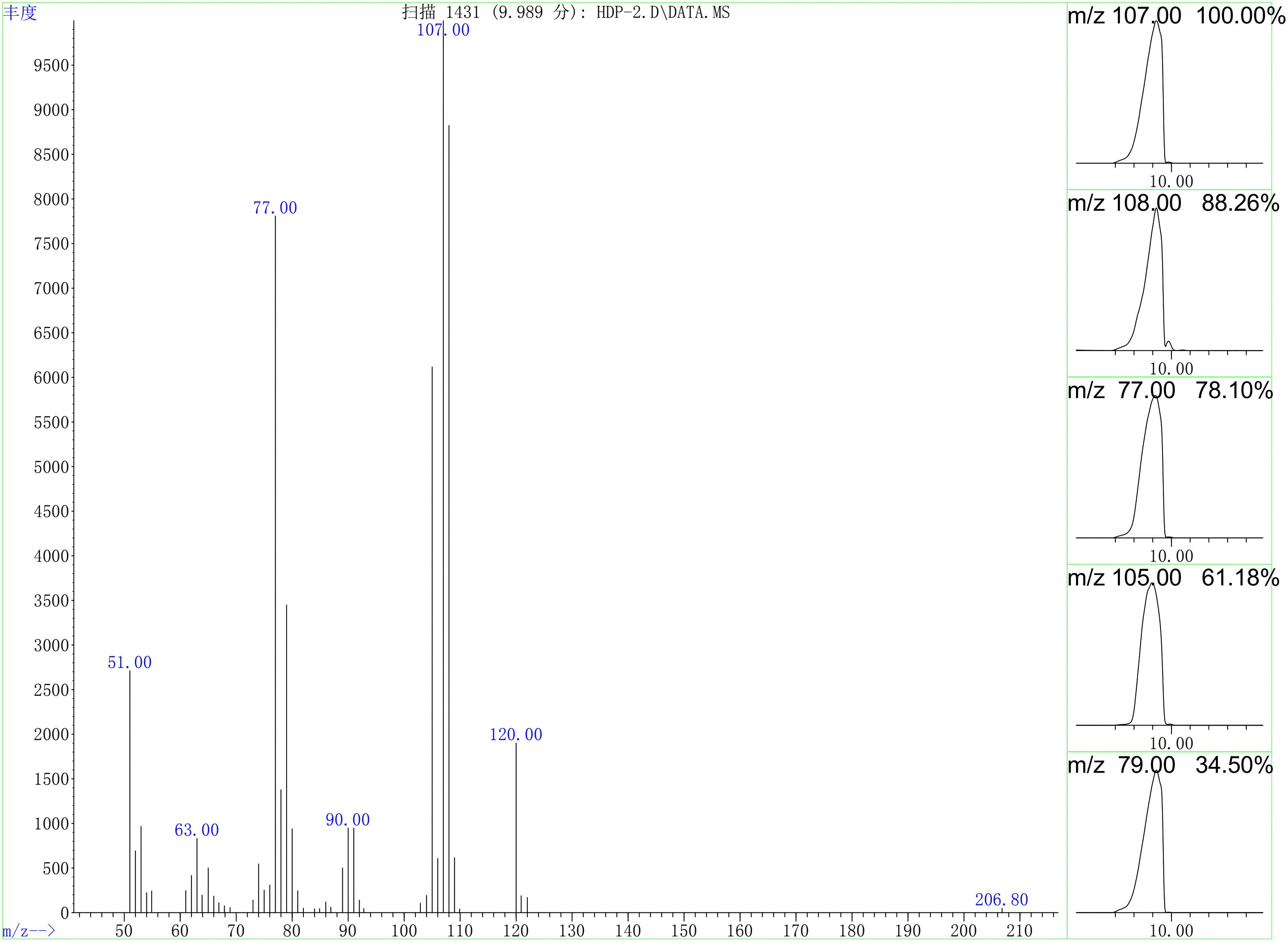

Data File: D:\GYM\DATA\2025\20251105\HDP-2.D
样品：HDP-2

峰编号： 9 9.989 分钟处 面积： 3435712 面积 % 0.11

每个谱库中 3 个最匹配的记录。 Ref\# CAS\# 匹配度

C:\database\DEMO.L 未检索到匹配。

未知谱图基于顶点

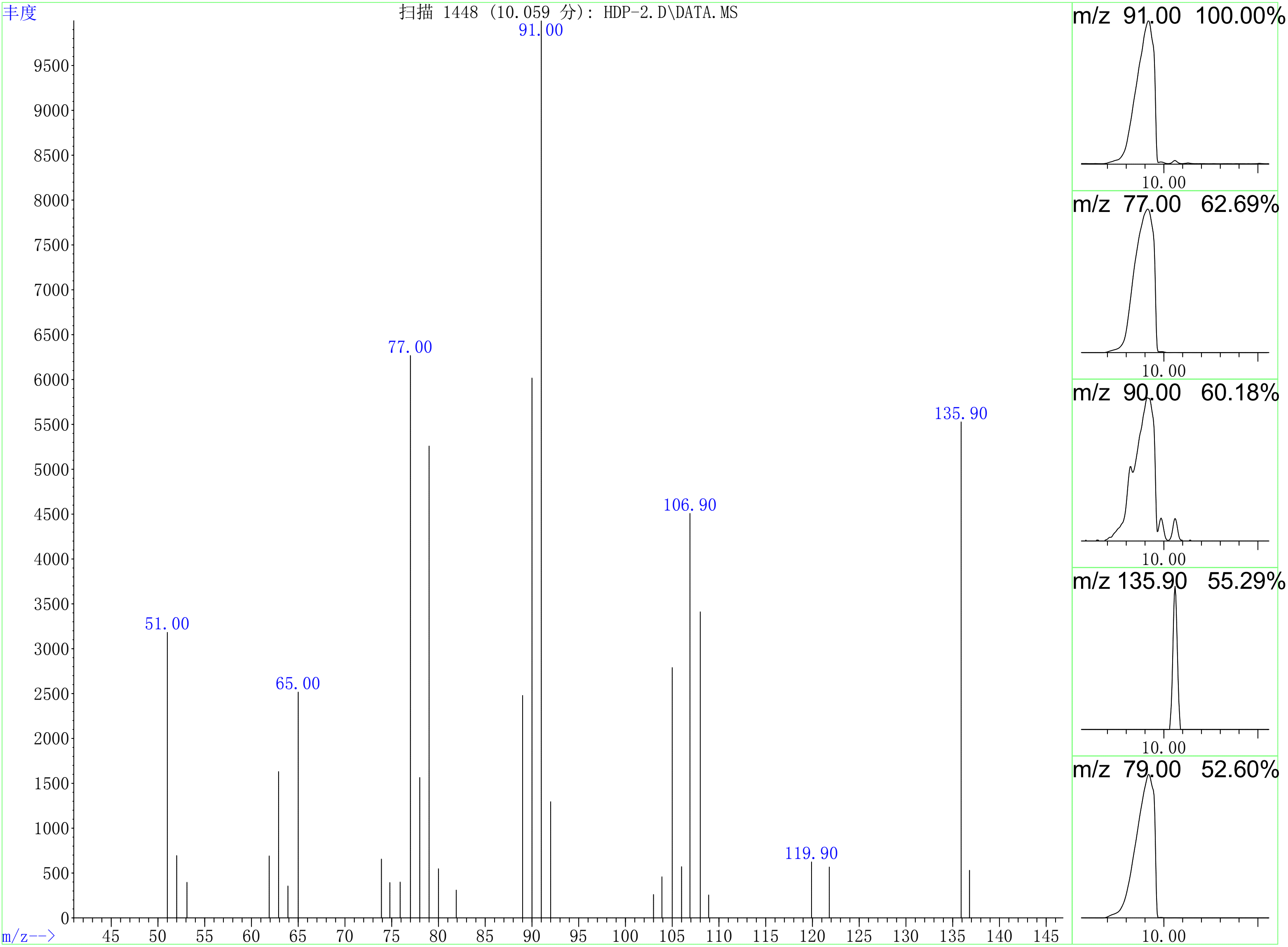

Data File: D:\GYM\DATA\2025\20251105\HDP-2.D
样品：HDP-2

峰编号： 10　　10.059 分钟处　面积：　598730　面积 % 0.02

每个谱库中 3 个最匹配的记录。　　Ref\#　CAS\#　匹配度

C:\database\DEMO.L　未检索到匹配。

未知谱图基于顶点

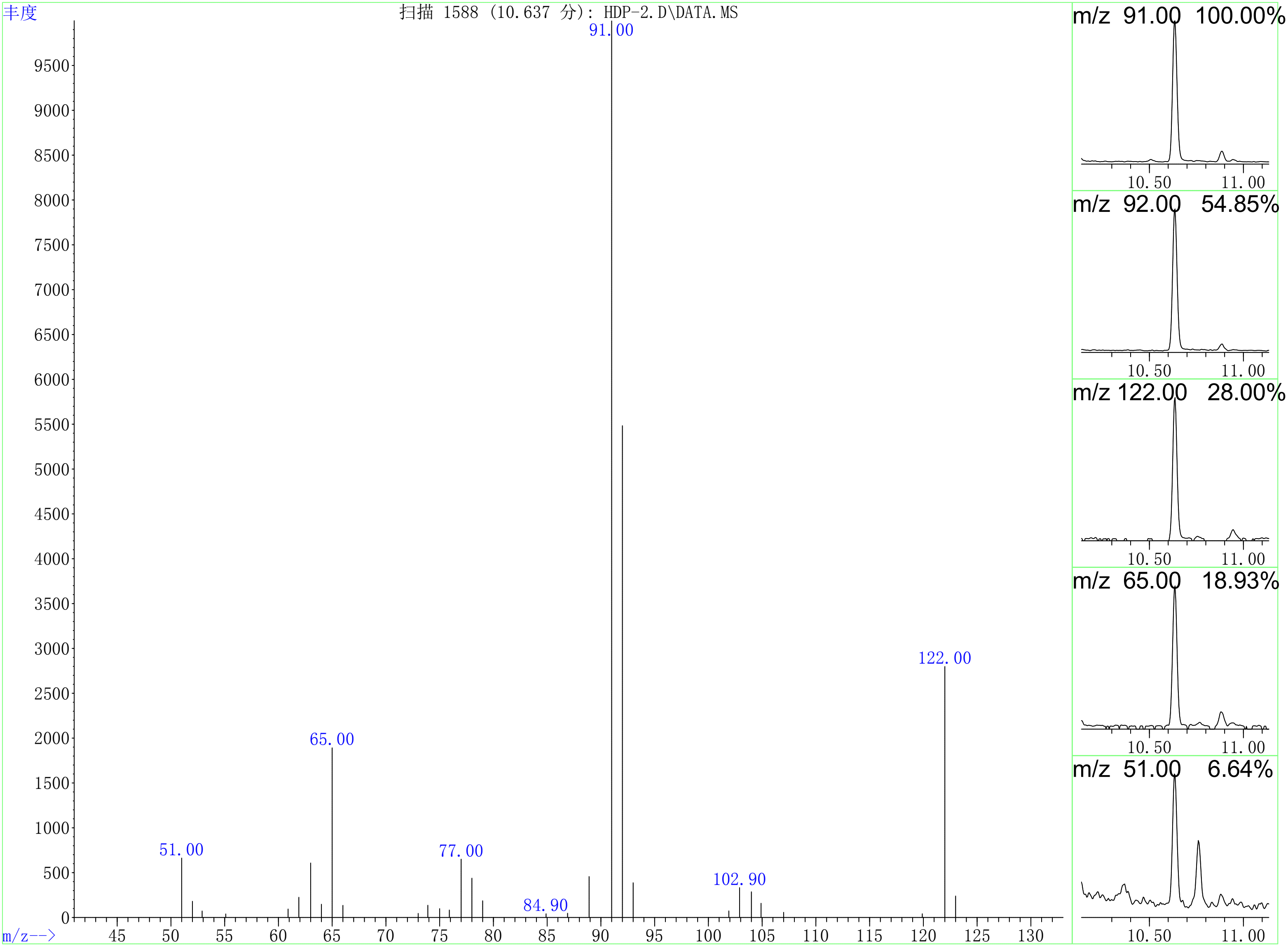

Data File: D:\GYM\DATA\2025\20251105\HDP-2.D
样品：HDP-2

峰编号： 11 10.637 分钟处 面积： 1787499 面积 % 0.06

每个谱库中 3 个最匹配的记录。 Ref\# CAS\# 匹配度

C:\database\DEMO.L 未检索到匹配。

未知谱图基于顶点

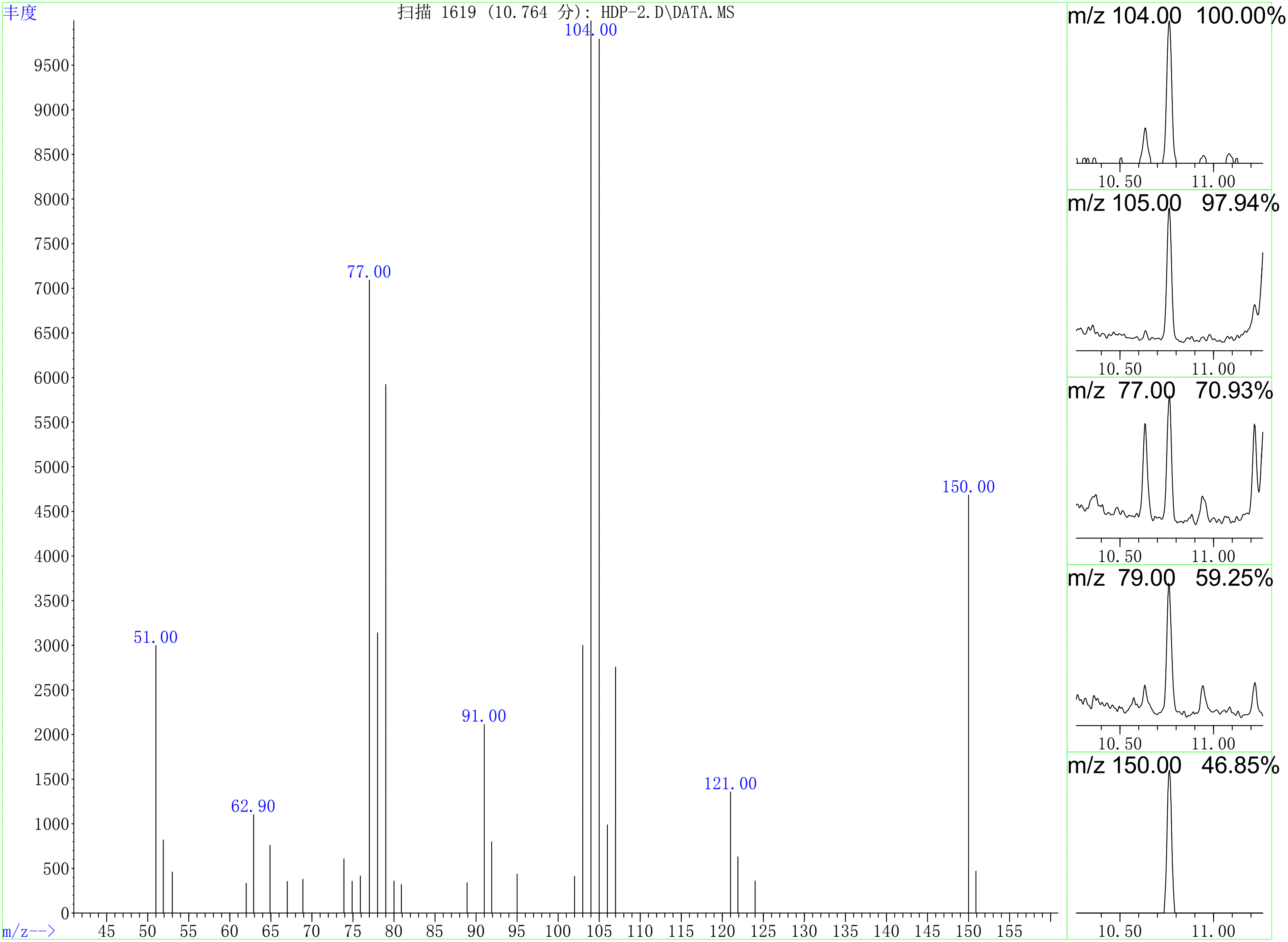

Data File: D:\GYM\DATA\2025\20251105\HDP-2.D
样品：HDP-2

峰编号： 12 10.764 分钟处 面积： 489886 面积 % 0.02

每个谱库中 3 个最匹配的记录。 Ref\# CAS\# 匹配度

C:\database\DEMO.L 未检索到匹配。

未知谱图基于顶点

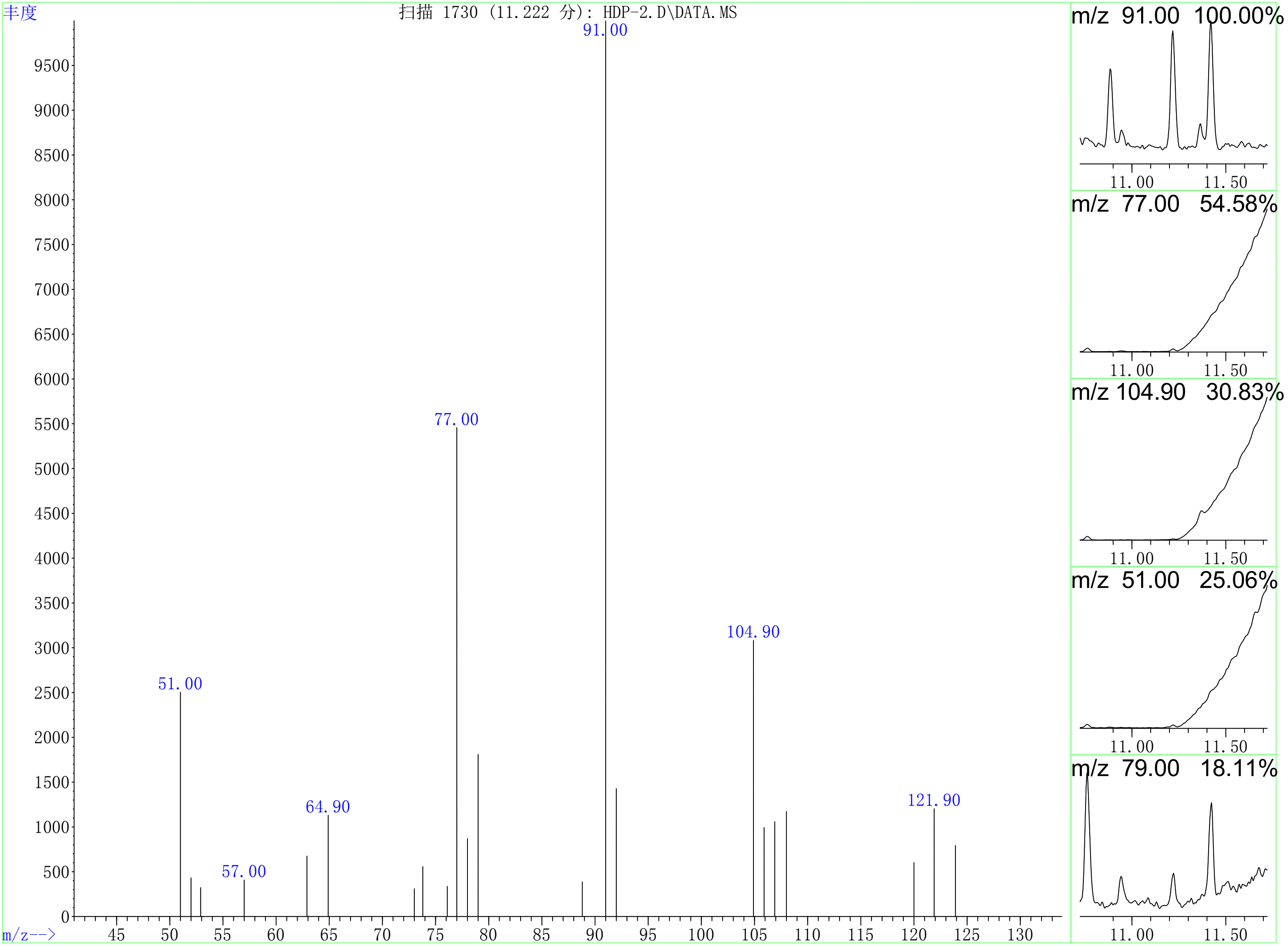

Data File: D:\GYM\DATA\2025\20251105\HDP-2.D
样品：HDP-2

峰编号： 13 11.222 分钟处 面积： 283475 面积 % 0.01

每个谱库中 3 个最匹配的记录。 Ref\# CAS\# 匹配度

C:\database\DEMO.L 未检索到匹配。

未知谱图基于顶点

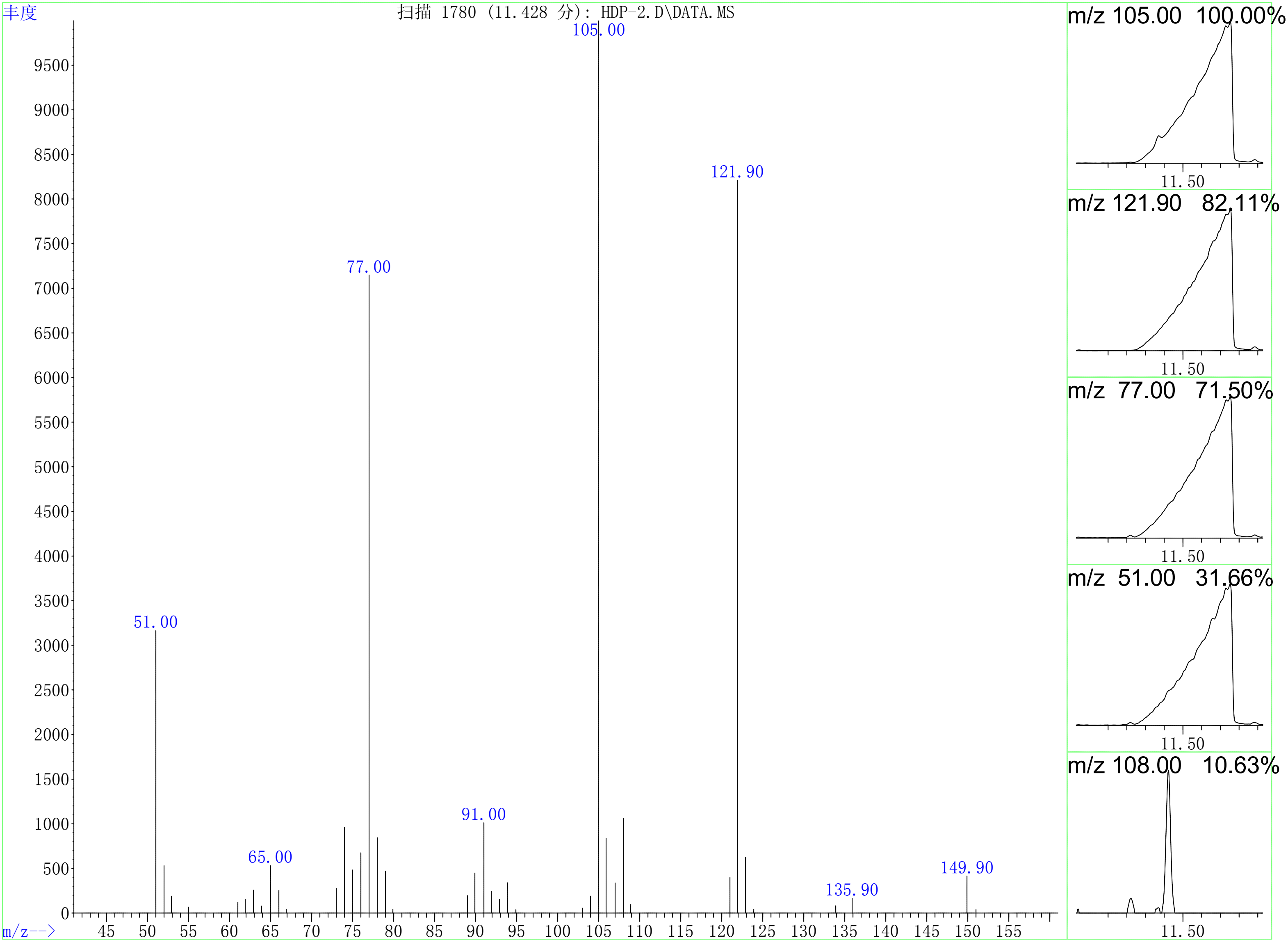

Data File: D:\GYM\DATA\2025\20251105\HDP-2.D
样品：HDP-2

峰编号： 14 11.428 分钟处 面积： 9916054 面积 % 0.31

每个谱库中 3 个最匹配的记录。 Ref\# CAS\# 匹配度

C:\database\DEMO.L 未检索到匹配。

未知谱图基于顶点

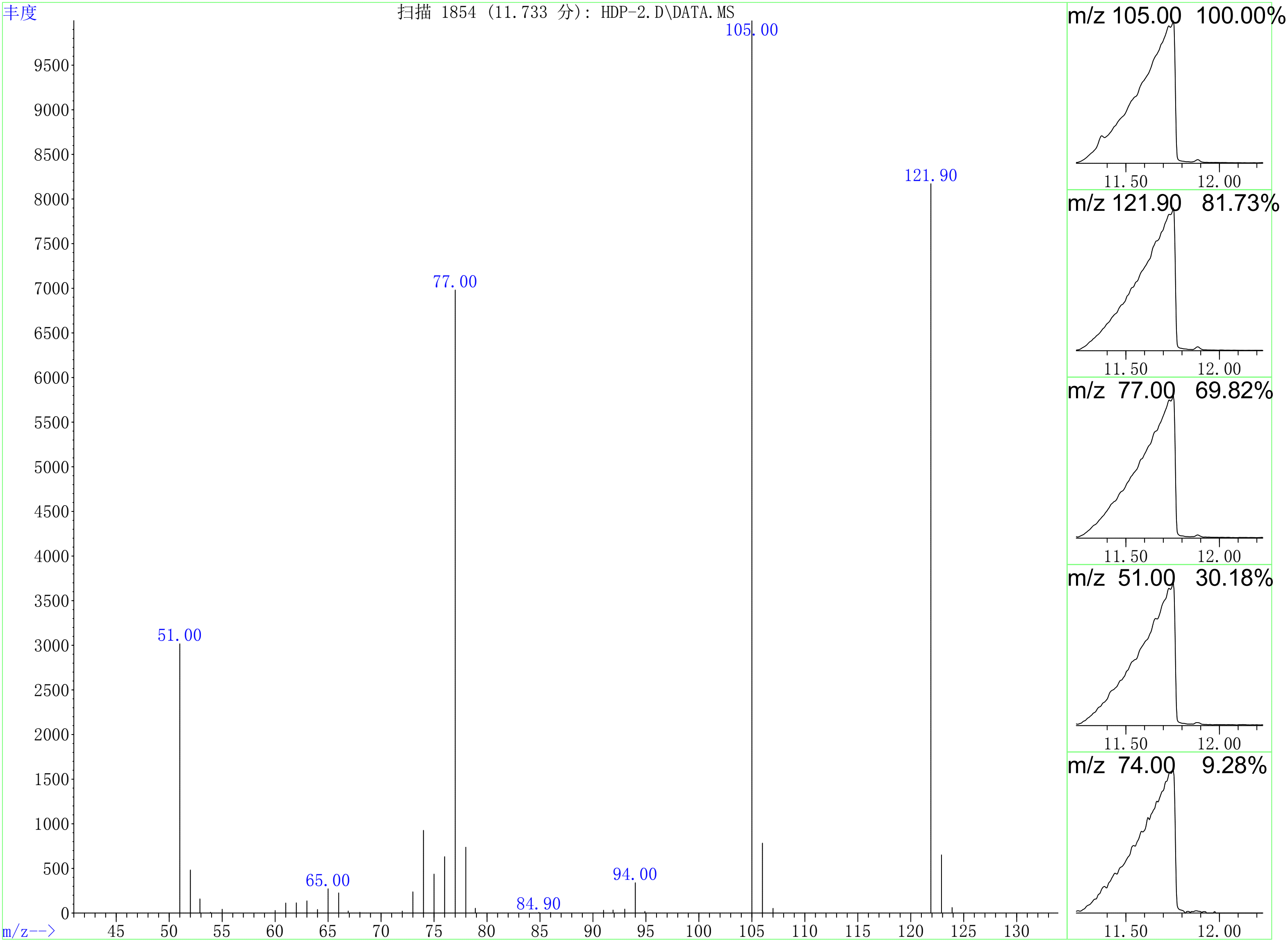

Data File: D:\GYM\DATA\2025\20251105\HDP-2.D
样品：HDP-2

峰编号： 15 11.733 分钟处 面积： 68054434 面积 % 2.13

每个谱库中 3 个最匹配的记录。 Ref\# CAS\# 匹配度

C:\database\DEMO.L 未检索到匹配。

未知谱图基于顶点

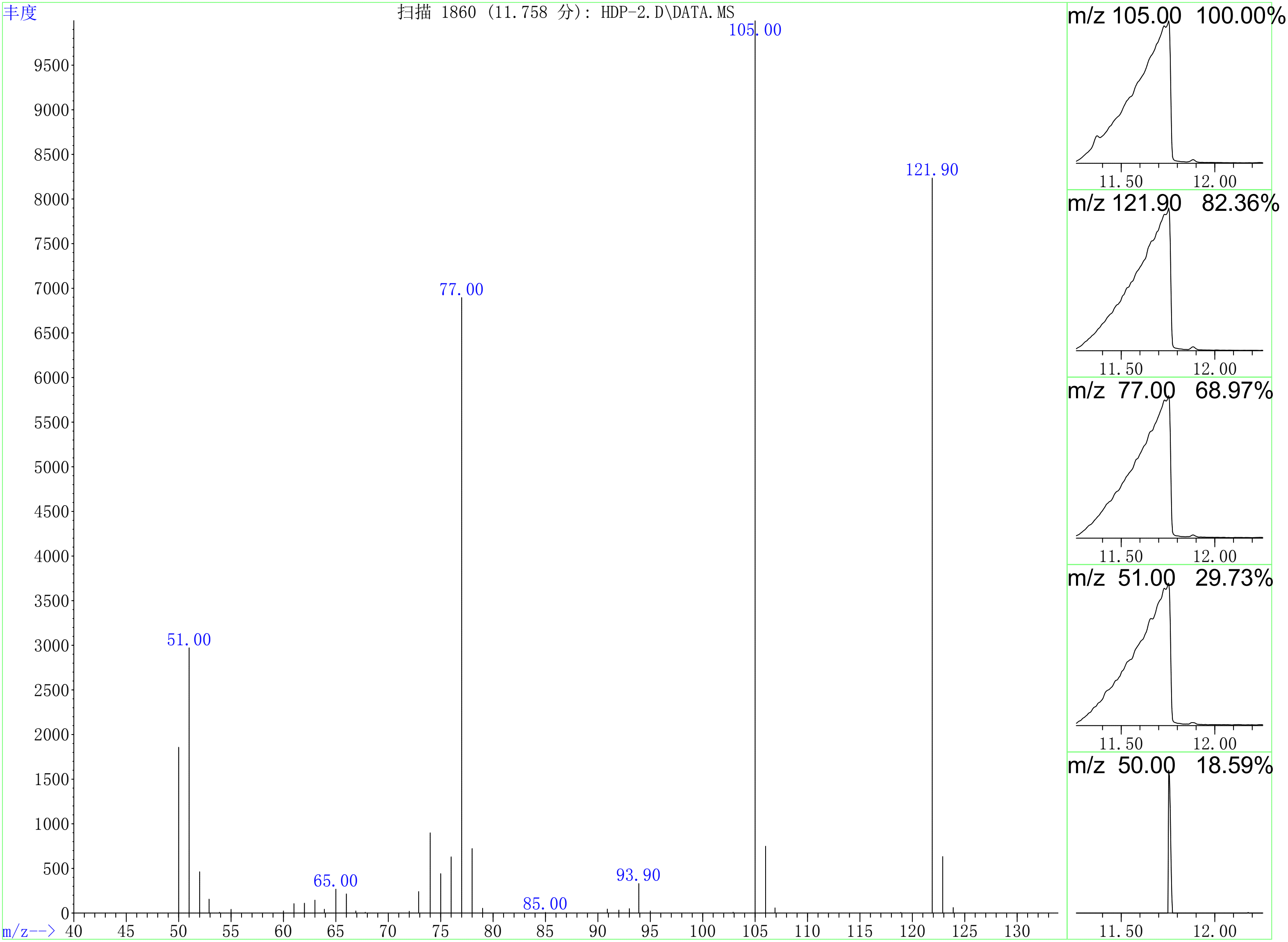

Data File: D:\GYM\DATA\2025\20251105\HDP-2.D
样品：HDP-2

峰编号： 16 11.758 分钟处 面积： 11072120 面积 % 0.35

每个谱库中 3 个最匹配的记录。 Ref\# CAS\# 匹配度

C:\database\DEMO.L 未检索到匹配。

未知谱图基于顶点

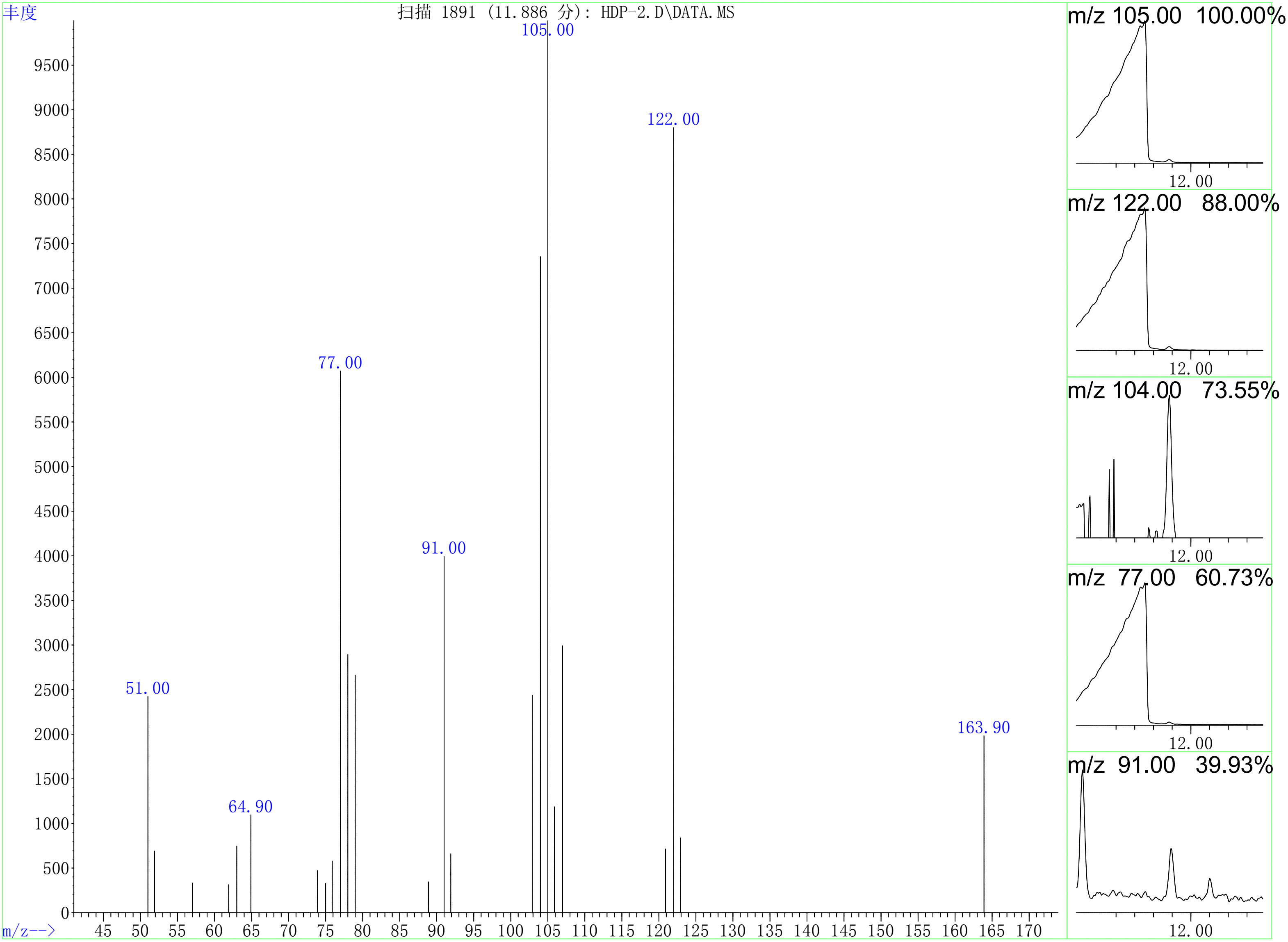

Data File: D:\GYM\DATA\2025\20251105\HDP-2.D
样品：HDP-2

峰编号： 17 11.886 分钟处 面积： 571995 面积 % 0.02

每个谱库中 3 个最匹配的记录。 Ref\# CAS\# 匹配度

C:\database\DEMO.L 未检索到匹配。

未知谱图基于顶点

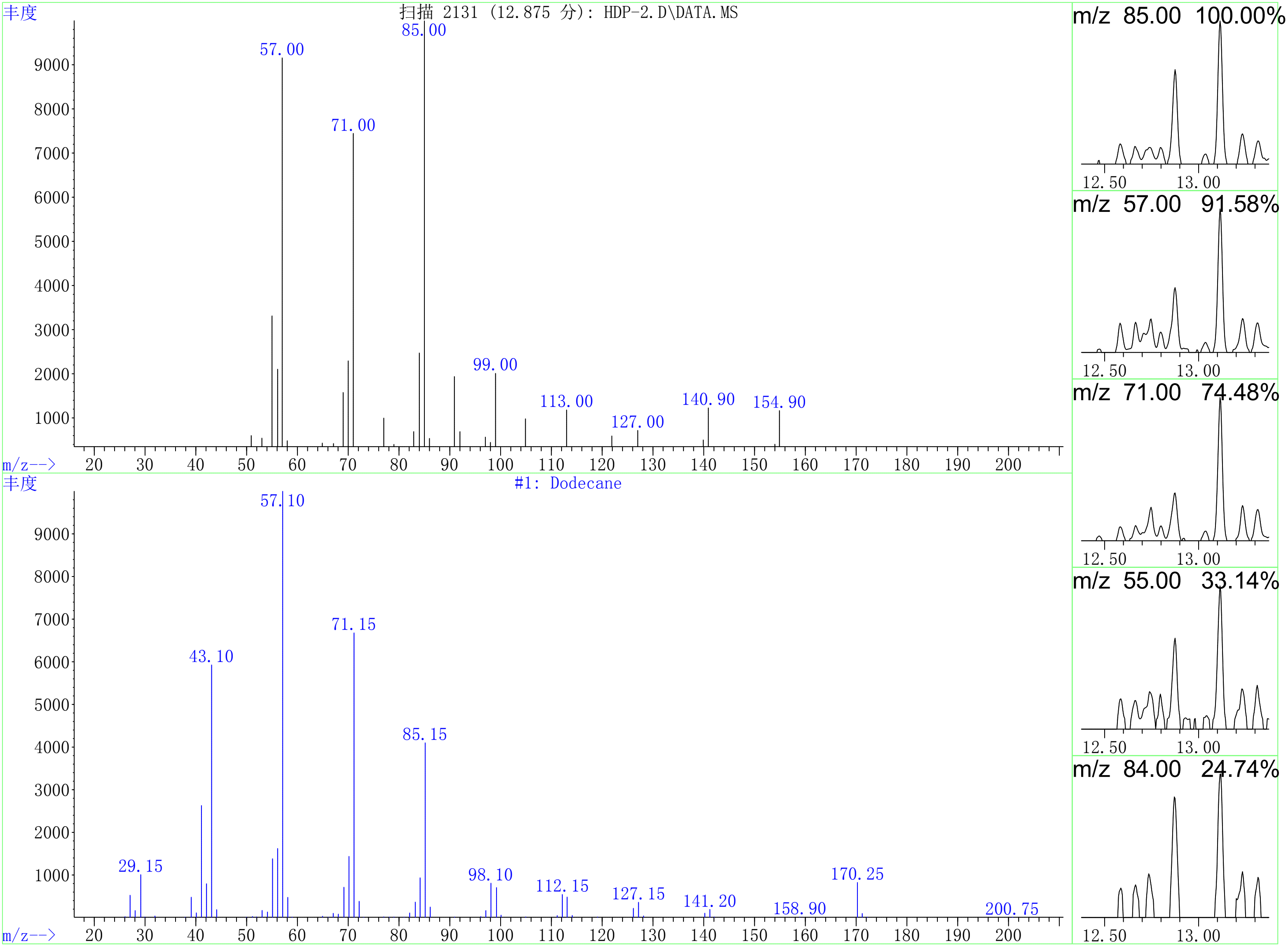

Data File: D:\GYM\DATA\2025\20251105\HDP-2.D
样品：HDP-2

峰编号： 18 12.875 分钟处 面积： 493133 面积 % 0.02

每个谱库中 3 个最匹配的记录。

	Ref\#	CAS\#	匹配度
C:\database\DEMO.L			
1 Dodecane	1	000112-40-3	4

未知谱图基于顶点

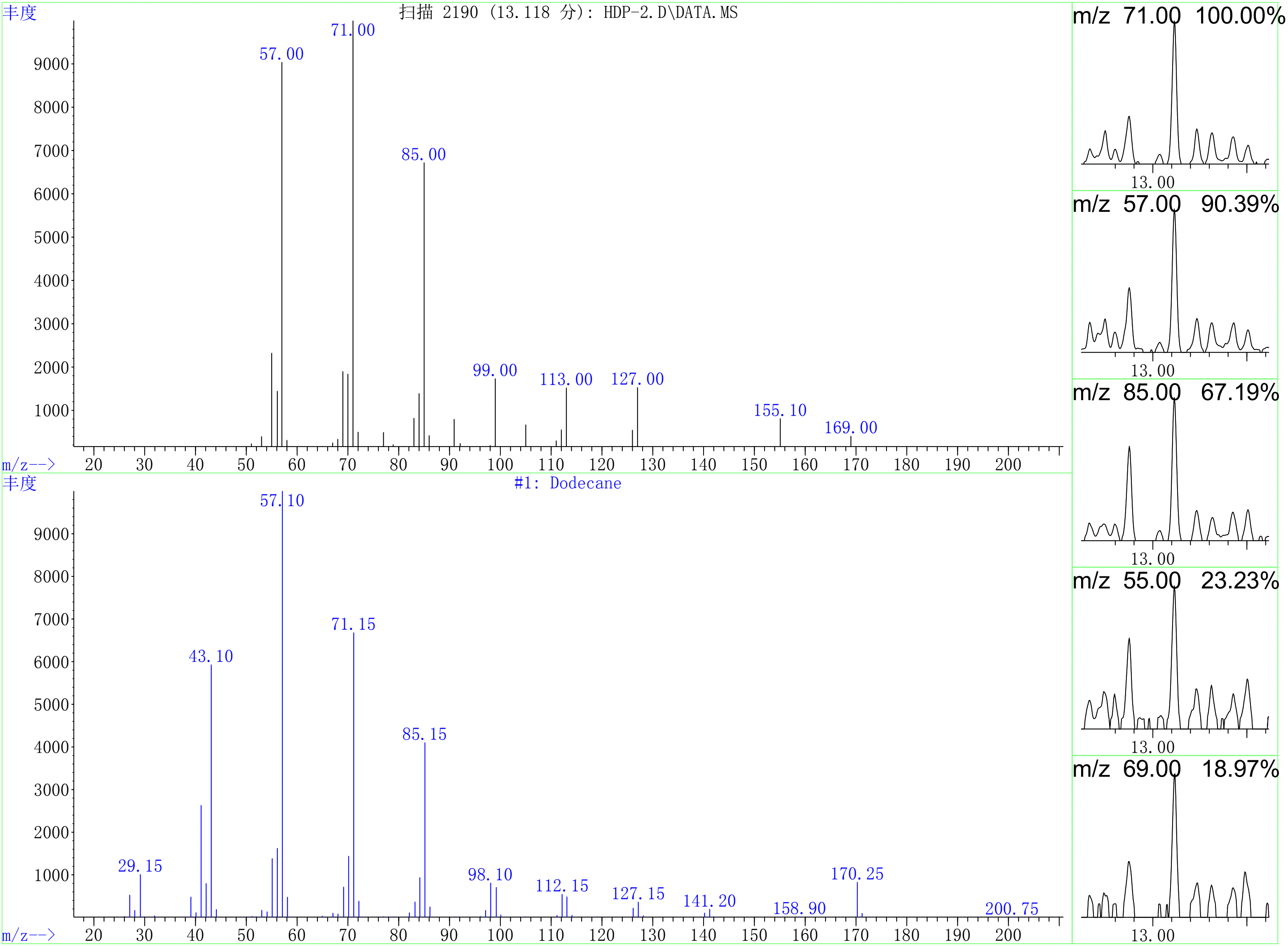

Data File: D:\GYM\DATA\2025\20251105\HDP-2.D
样品：HDP-2

峰编号： 19 13.118 分钟处 面积： 1126062 面积 % 0.04

每个谱库中 3 个最匹配的记录。	Ref\#	CAS\#	匹配度
C:\database\DEMO.L			
1 Dodecane	1	000112-40-3	64

未知谱图基于顶点

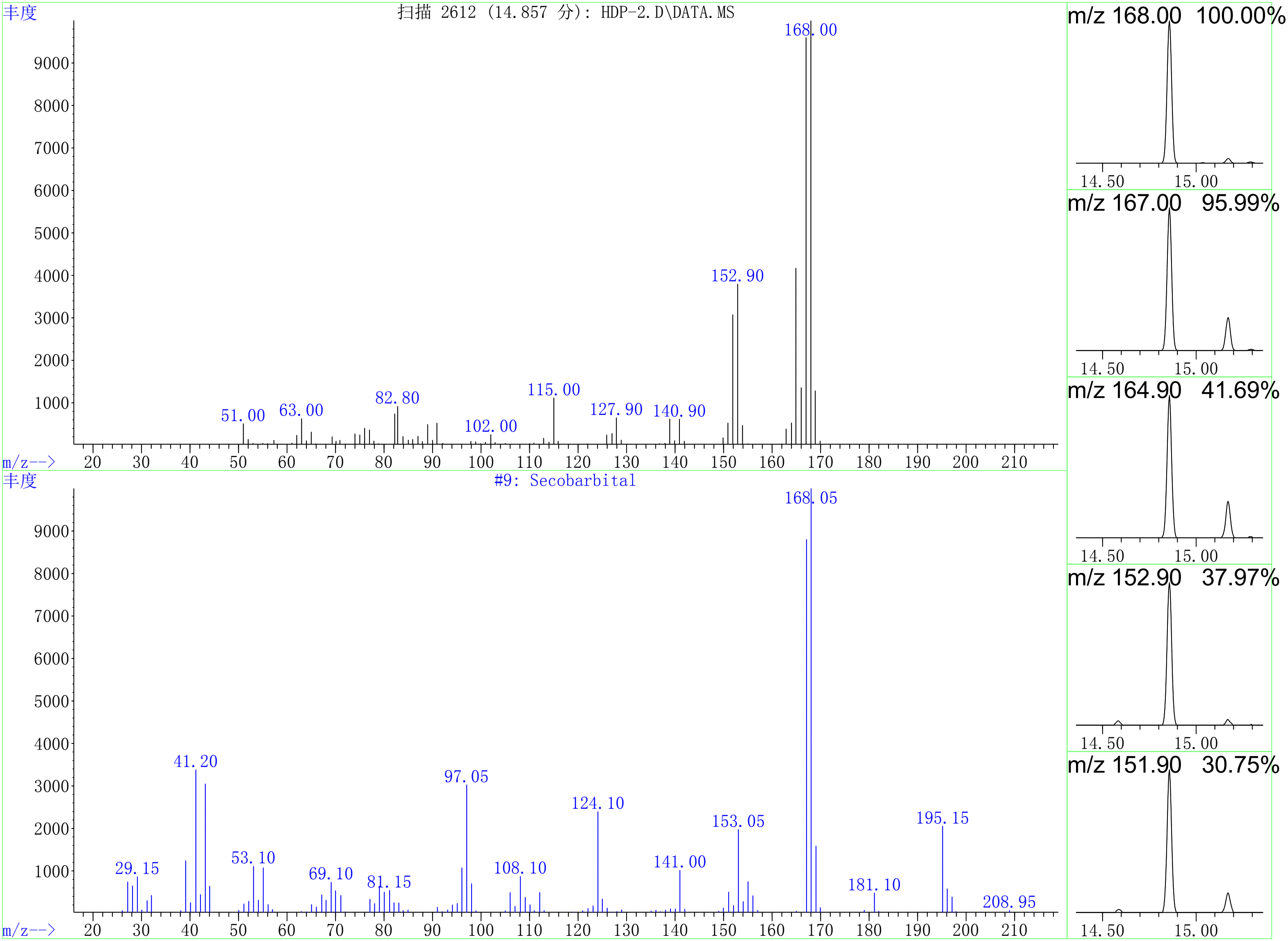

Data File: D:\GYM\DATA\2025\20251105\HDP-2.D
样品：HDP-2

峰编号： 20 14.857 分钟处 面积： 7127722 面积 % 0.22

每个谱库中 3 个最匹配的记录。	Ref\#	CAS\#	匹配度
C:\database\DEMO.L			
1 Secobarbital	9	000309-43-3	4

未知谱图基于顶点

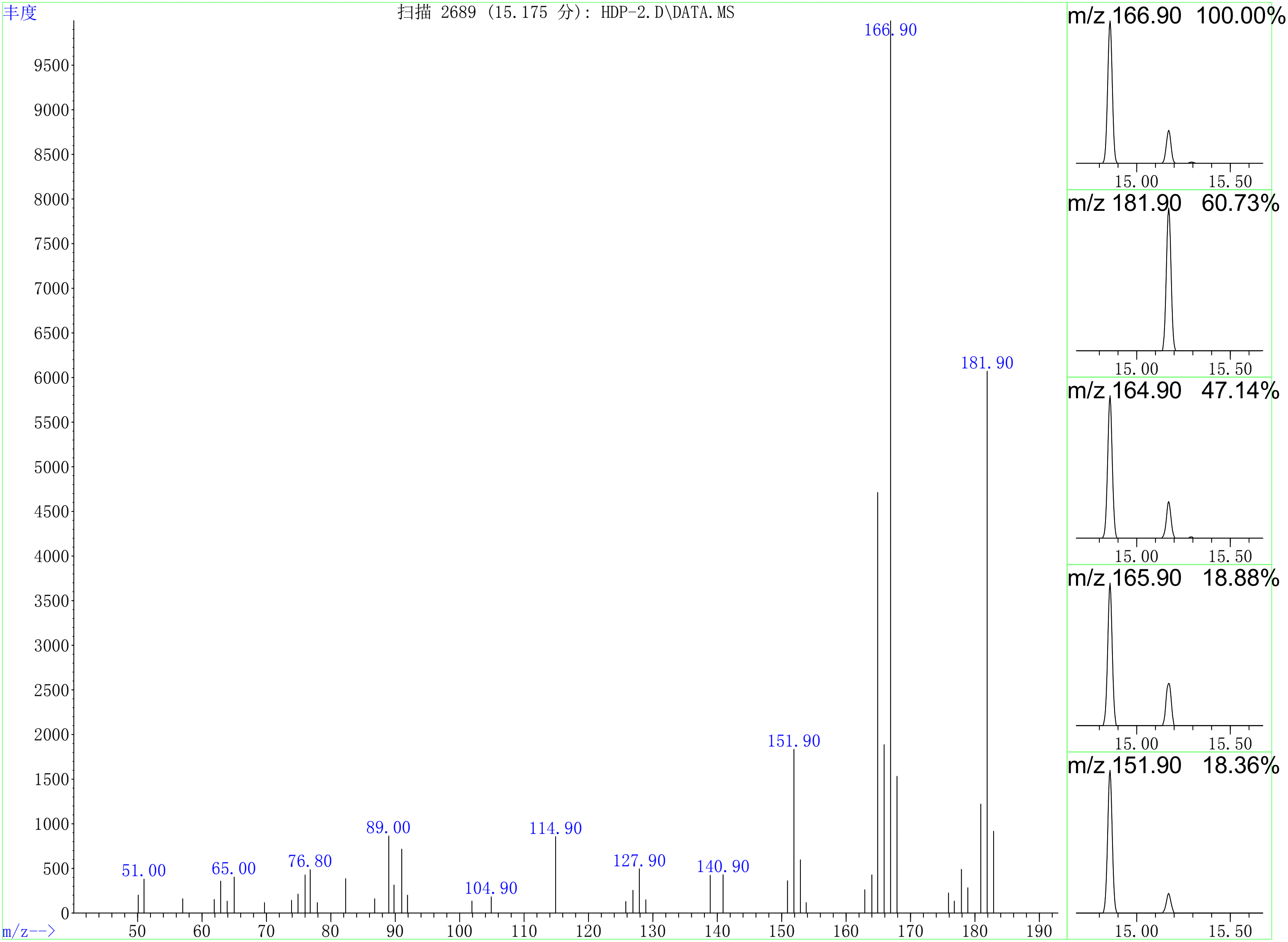

Data File: D:\GYM\DATA\2025\20251105\HDP-2.D
样品：HDP-2

峰编号： 21 15.175 分钟处 面积： 1030523 面积 % 0.03

每个谱库中 3 个最匹配的记录。 Ref\# CAS\# 匹配度

C:\database\DEMO.L 未检索到匹配。

未知谱图基于顶点

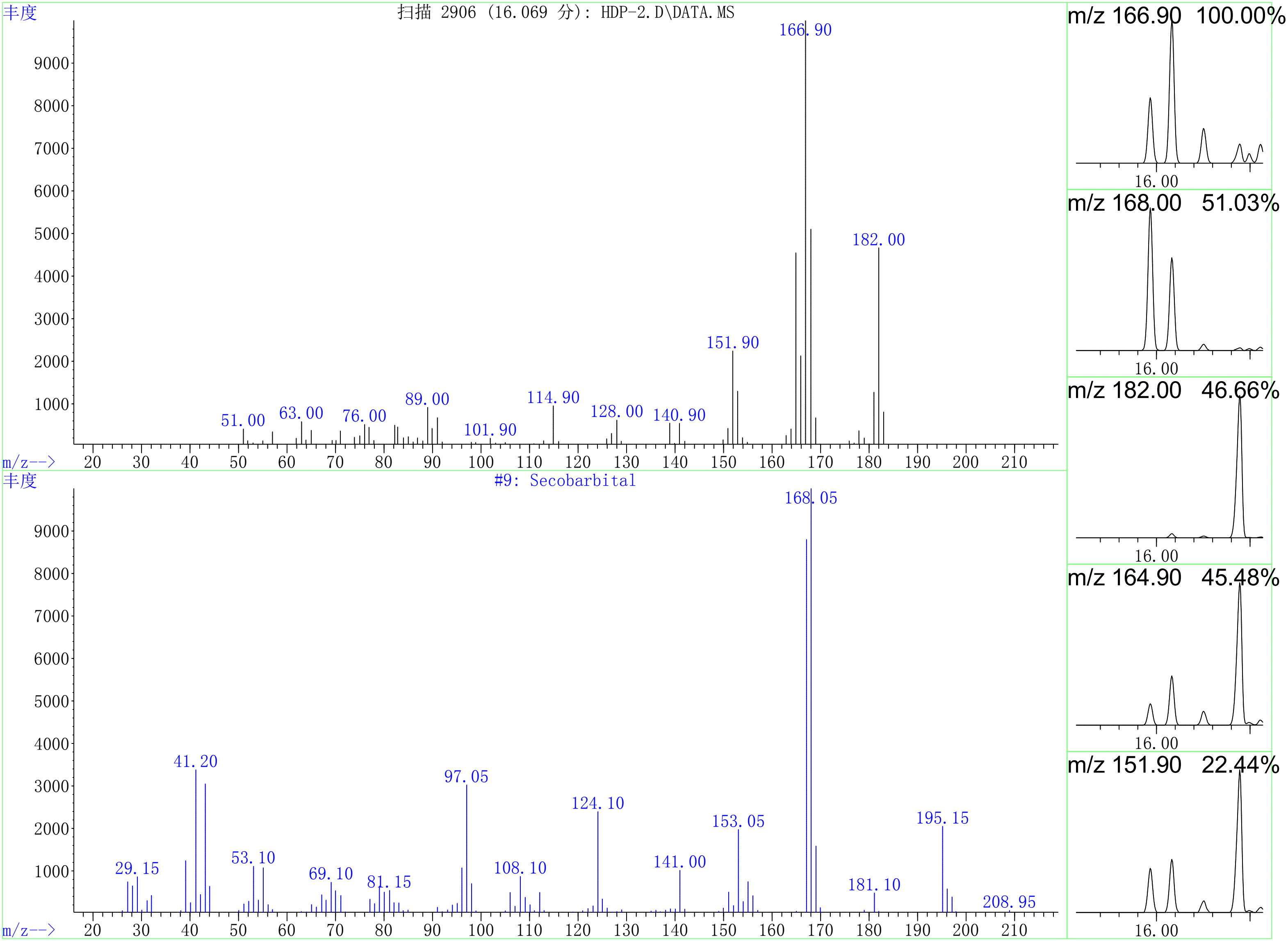

Data File: D:\GYM\DATA\2025\20251105\HDP-2.D
样品：HDP-2

峰编号： 22　　16.069 分钟处　面积： 13398920　面积 % 0.42

每个谱库中 3 个最匹配的记录。	Ref\#	CAS\#	匹配度
C:\database\DEMO.L			
1 Secobarbital	9	000309-43-3	8

未知谱图基于顶点

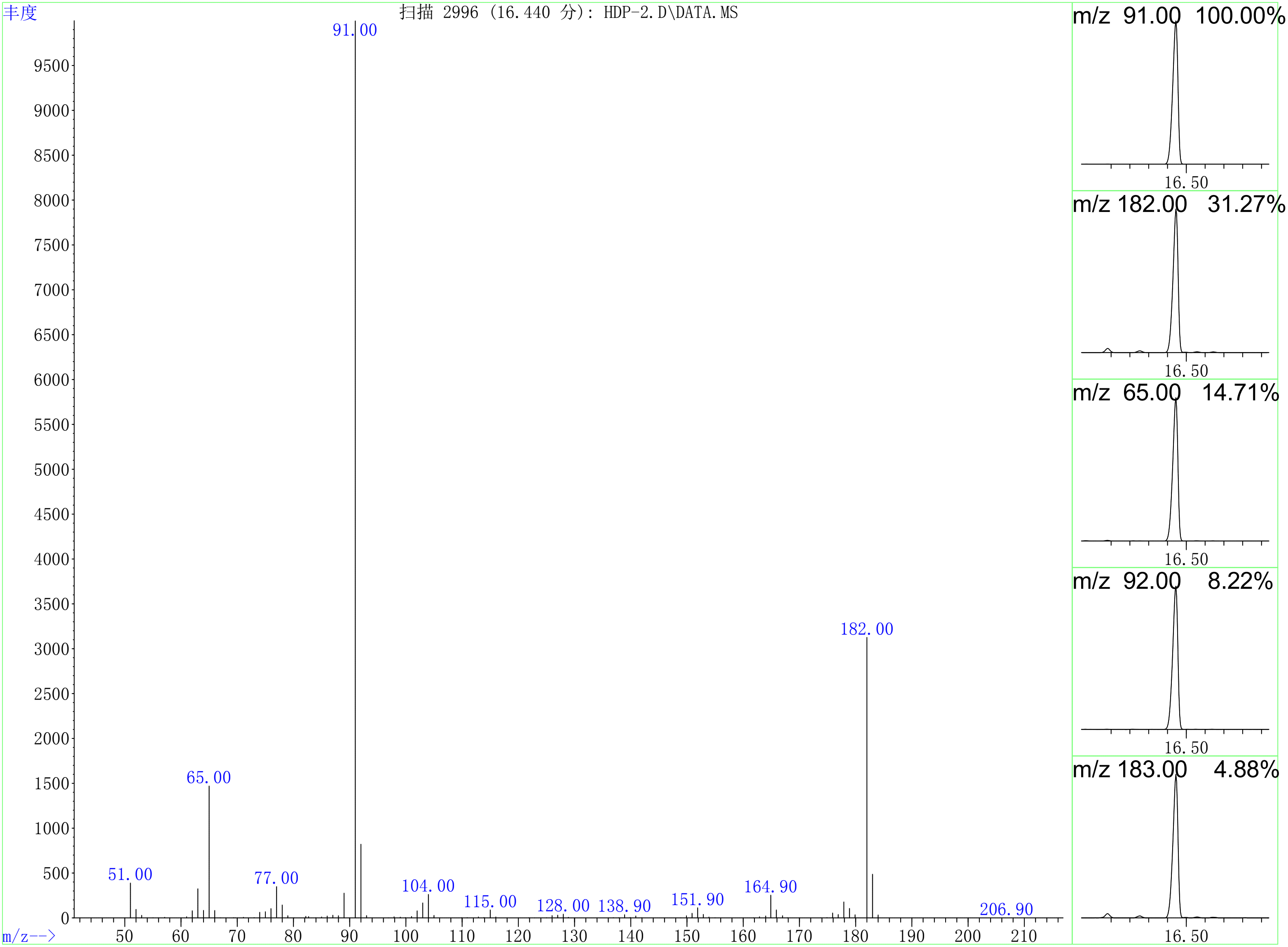

Data File: D:\GYM\DATA\2025\20251105\HDP-2.D
样品：HDP-2

峰编号： 23   16.440 分钟处 面积：108959534 面积 % 3.40

每个谱库中 3 个最匹配的记录。   Ref\# CAS\# 匹配度

C:\database\DEMO.L 未检索到匹配。

未知谱图基于顶点

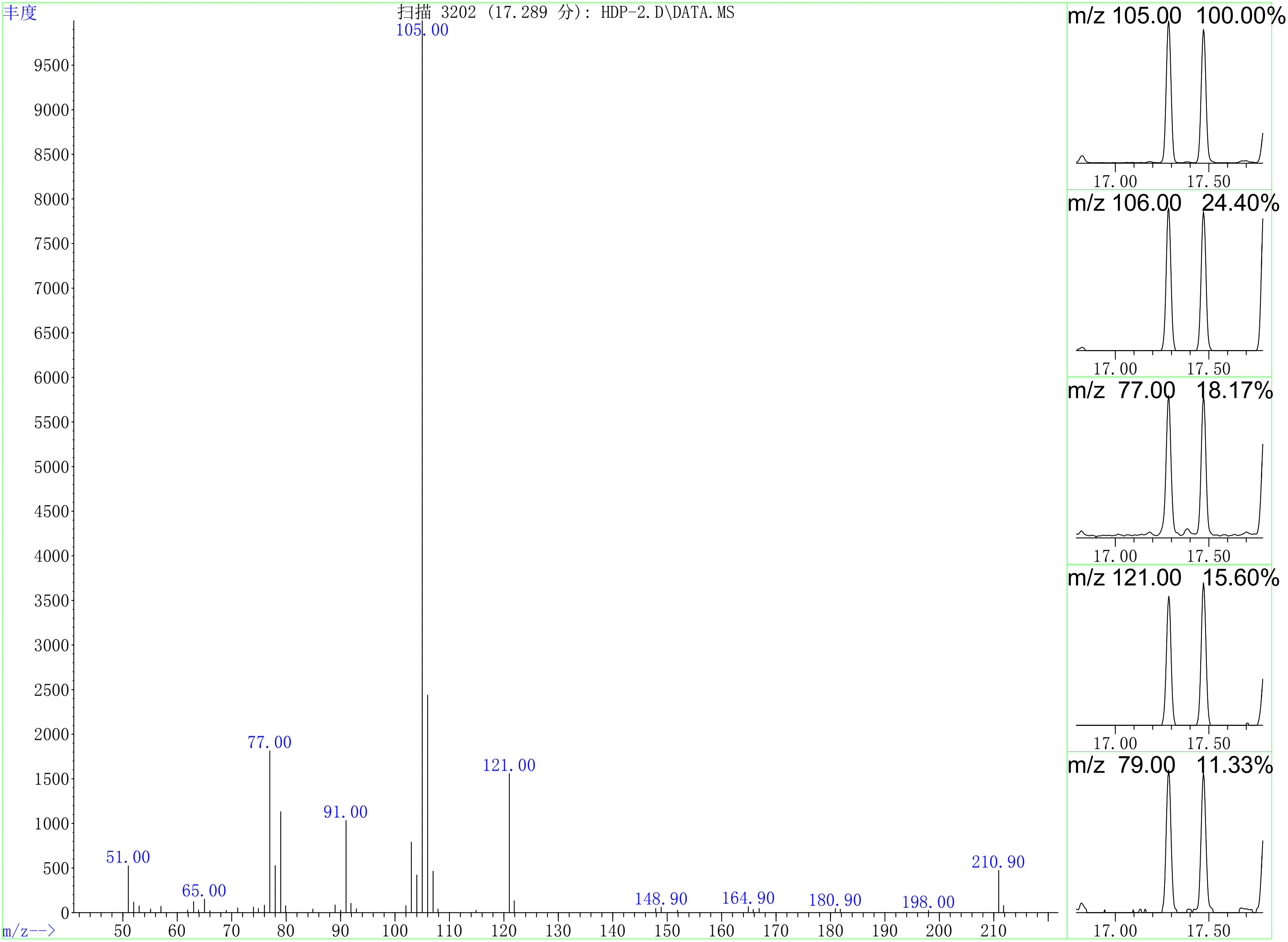

Data File: D:\GYM\DATA\2025\20251105\HDP-2.D
样品：HDP-2

峰编号： 24 17.289 分钟处 面积： 24321331 面积 % 0.76

每个谱库中 3 个最匹配的记录。 Ref\# CAS\# 匹配度

C:\database\DEMO.L 未检索到匹配。

未知谱图基于顶点

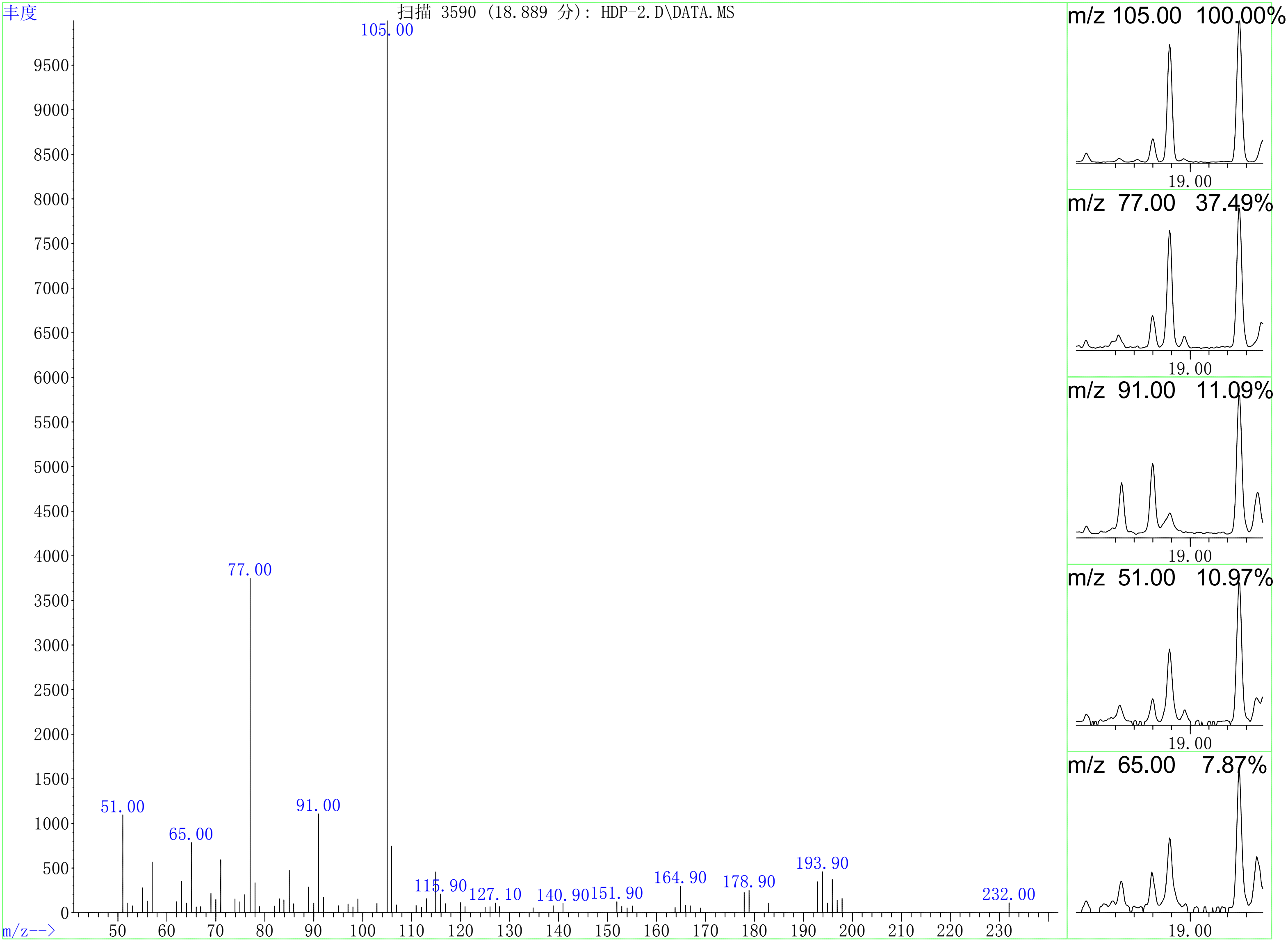

Data File: D:\GYM\DATA\2025\20251105\HDP-2.D
样品：HDP-2

峰编号： 25 18.889 分钟处 面积： 5726436 面积 % 0.18

每个谱库中 3 个最匹配的记录。 Ref\# CAS\# 匹配度

C:\database\DEMO.L 未检索到匹配。

未知谱图基于顶点

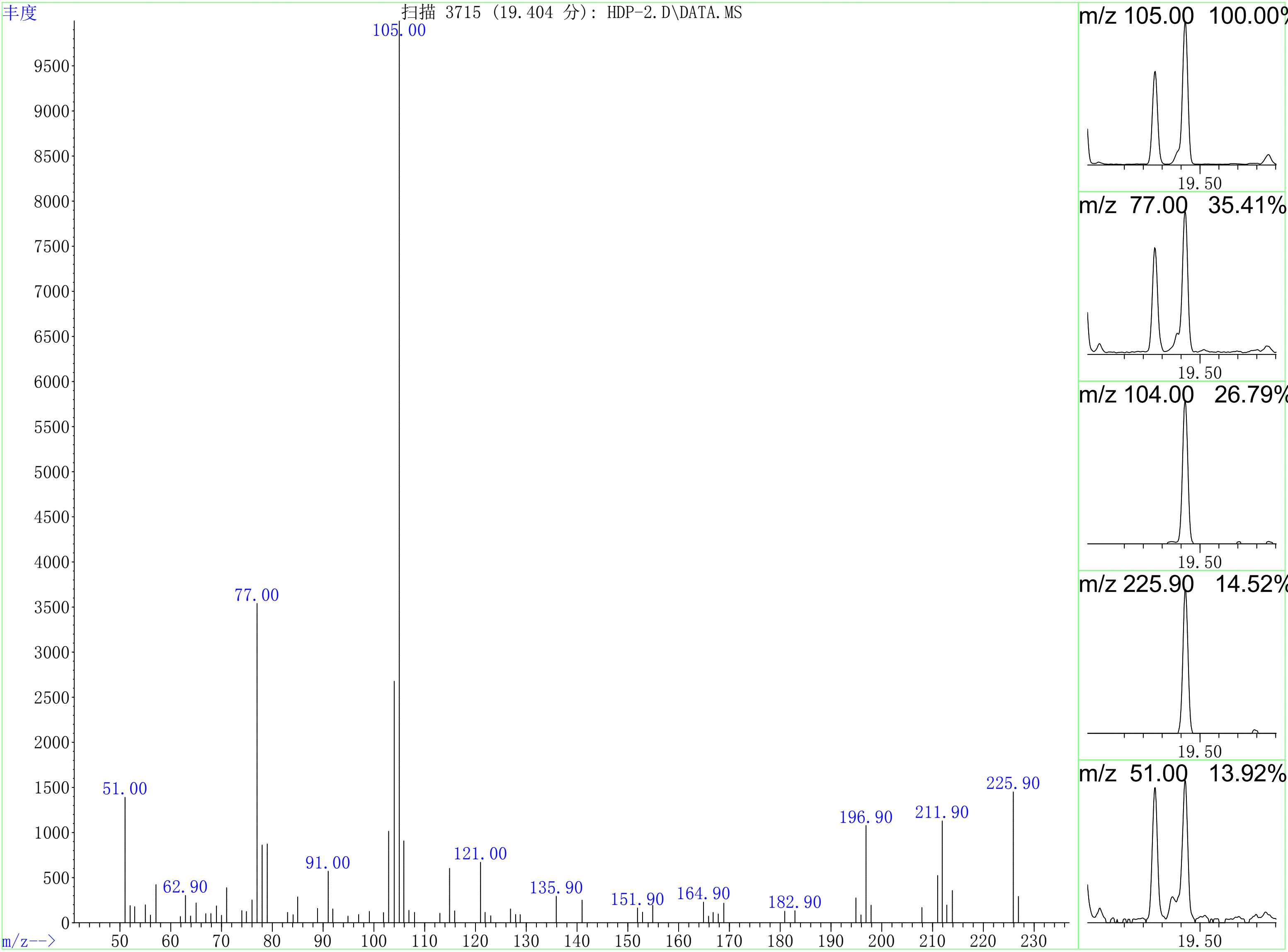

Data File: D:\GYM\DATA\2025\20251105\HDP-2.D
样品：HDP-2

峰编号： 26   19.404 分钟处 面积： 10830859 面积 % 0.34

每个谱库中 3 个最匹配的记录。   Ref\# CAS\# 匹配度

C:\database\DEMO.L 未检索到匹配。

未知谱图基于顶点

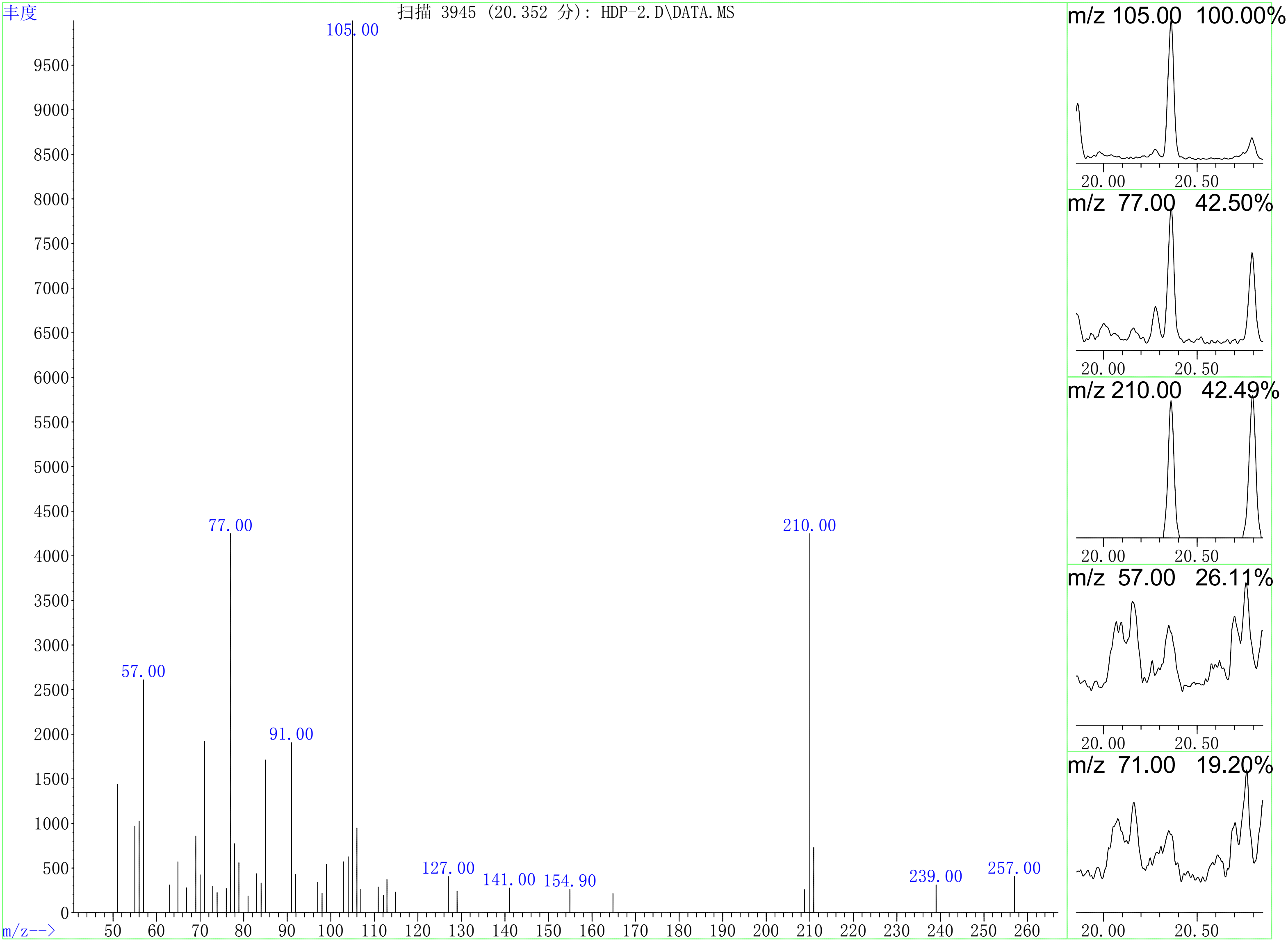

Data File: D:\GYM\DATA\2025\20251105\HDP-2.D
样品：HDP-2

峰编号： 27 20.352 分钟处 面积： 4338801 面积 % 0.14

每个谱库中 3 个最匹配的记录。 Ref\# CAS\# 匹配度

C:\database\DEMO.L 未检索到匹配。

未知谱图基于顶点

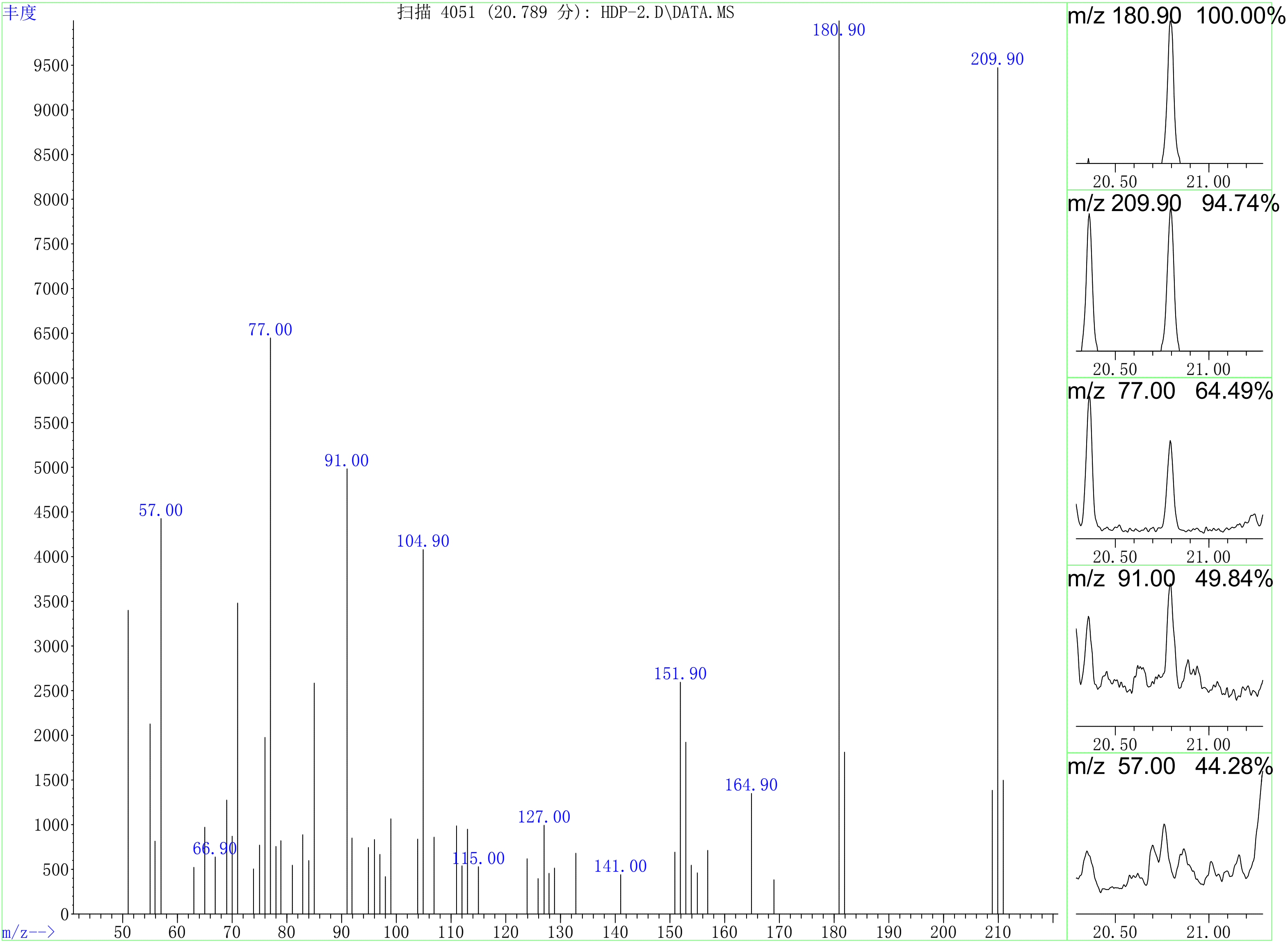

Data File: D:\GYM\DATA\2025\20251105\HDP-2.D
样品：HDP-2

峰编号： 28　　20.789 分钟处　面积： 2554636　面积 % 0.08

每个谱库中 3 个最匹配的记录。　　Ref\#　CAS\#　匹配度

C:\database\DEMO.L　未检索到匹配。

未知谱图基于顶点

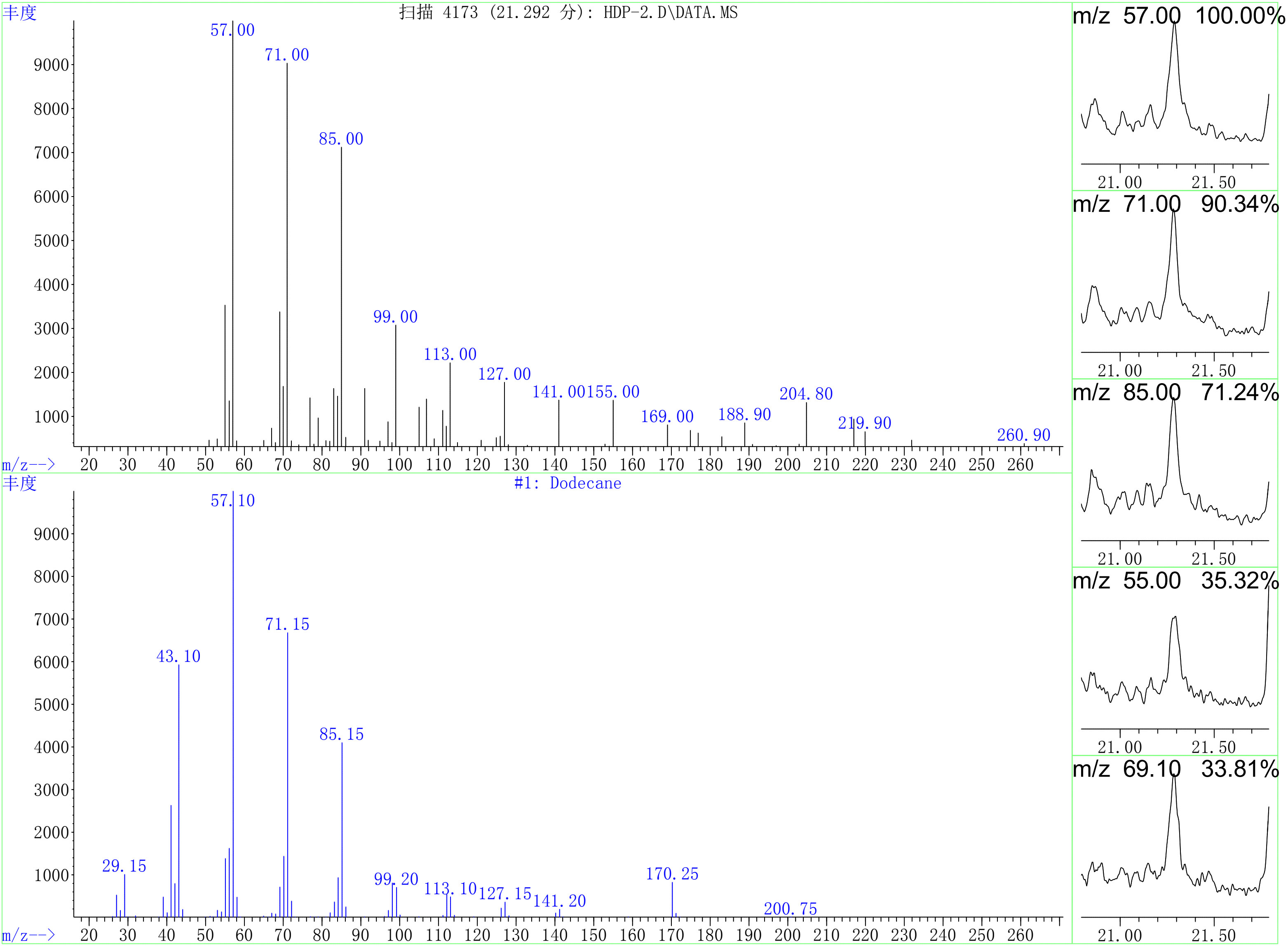

Data File: D:\GYM\DATA\2025\20251105\HDP-2.D
样品：HDP-2

峰编号： 29 21.292 分钟处 面积： 3480463 面积 % 0.11

每个谱库中 3 个最匹配的记录。 Ref\# CAS\# 匹配度

C:\database\DEMO.L

1 Dodecane	1	000112-40-3	38

未知谱图基于顶点

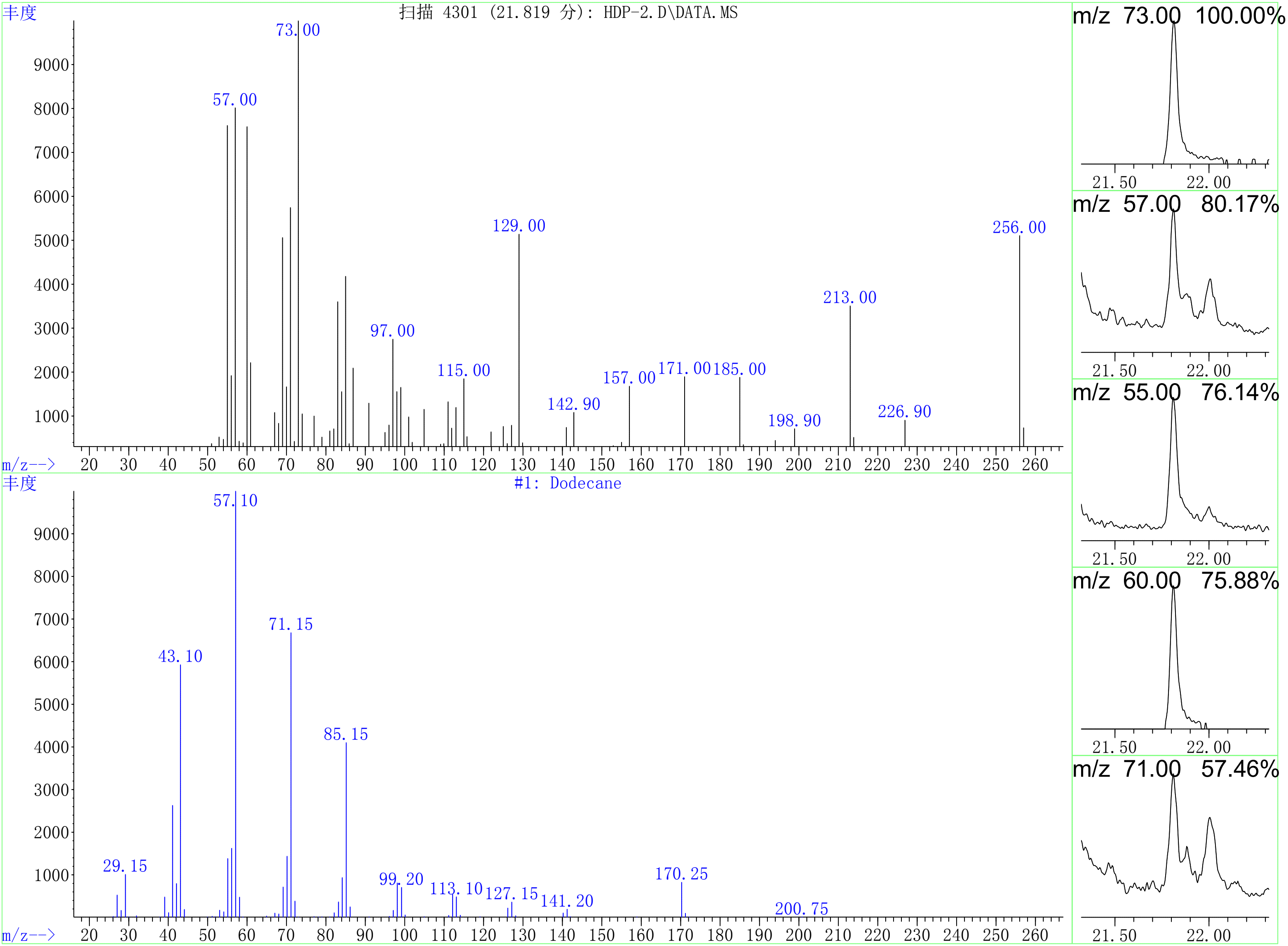

Data File: D:\GYM\DATA\2025\20251105\HDP-2.D
样品：HDP-2

峰编号： 30 21.819 分钟处 面积： 3421039 面积 % 0.11

每个谱库中 3 个最匹配的记录。	Ref\#	CAS\#	匹配度
C:\database\DEMO.L			
1 Dodecane	1	000112-40-3	10
