# Supplementary material for: High-throughput chiral copper foils by curved-surface confinement recrystallization
Source: Nat Commun. 2026 Feb 20;17:2796. doi: 10.1038/s41467-026-69862-7 (PMC13022495; doi:10.1038/s41467-026-69862-7)
Supplement: Supplementary file 3 — Supplementary Dataset 1 [file 41467_2026_69862_MOESM3_ESM.zip › Supplementary Data 1/Supplementary Fig.14-16/GC-MS Cu(5 6 6)catalyst.pdf]

谱库检索报告

数据路径 : D:\GYM\DATA\2025\20251105\
数据文件 : HDP-1.D
采集 : 05 Nov 2025 16:40
操作者 : zky-HP\zky
样品 : HDP-1
其他 :
ALS 样品瓶: 3 样品乘积因子: 1

检索库： C:\database\DEMO.L 最小匹配度： 0

未知谱图： 顶点
积分事件： 化学工作站积分器 - events14.e

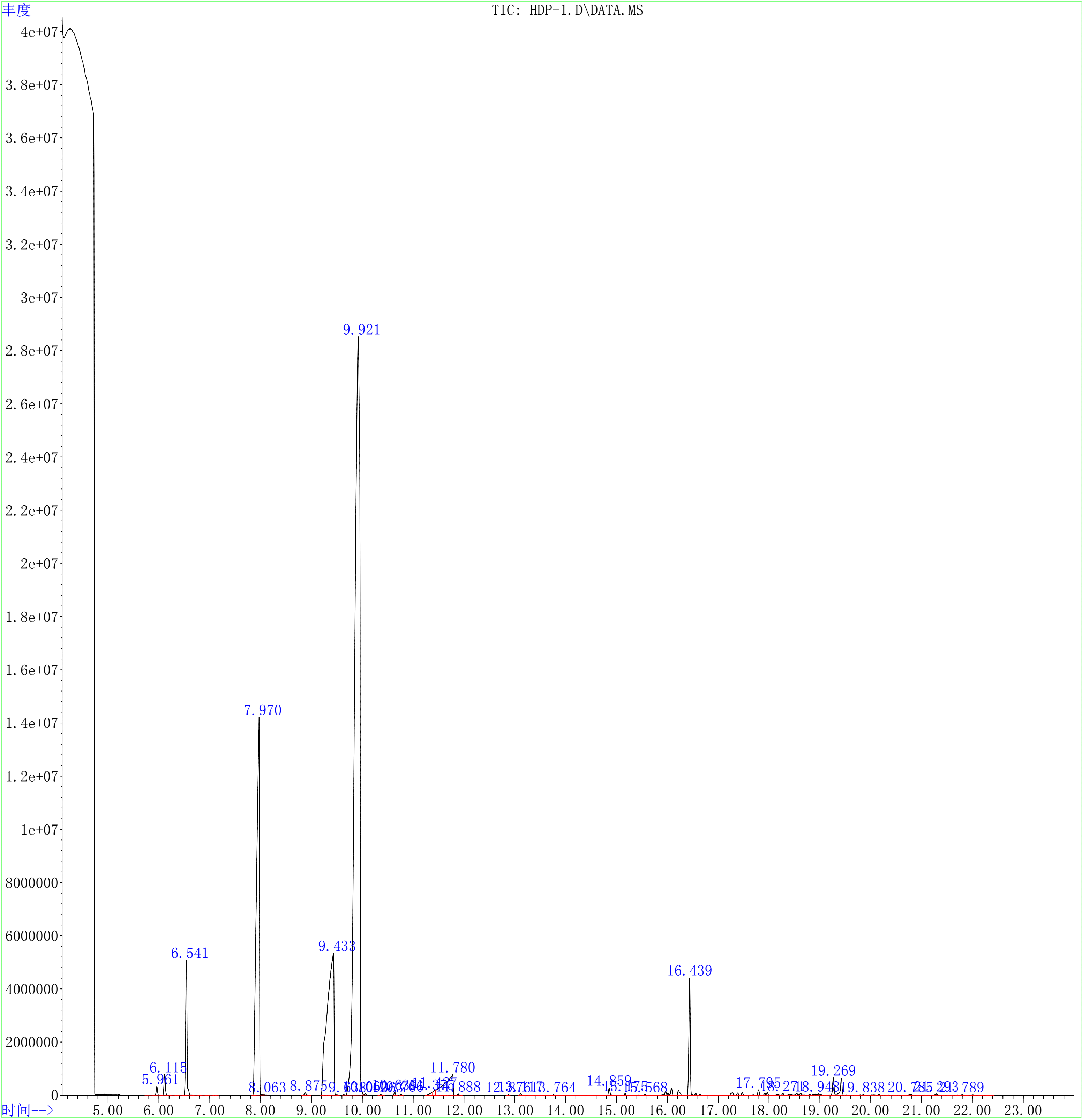

未知谱图基于顶点

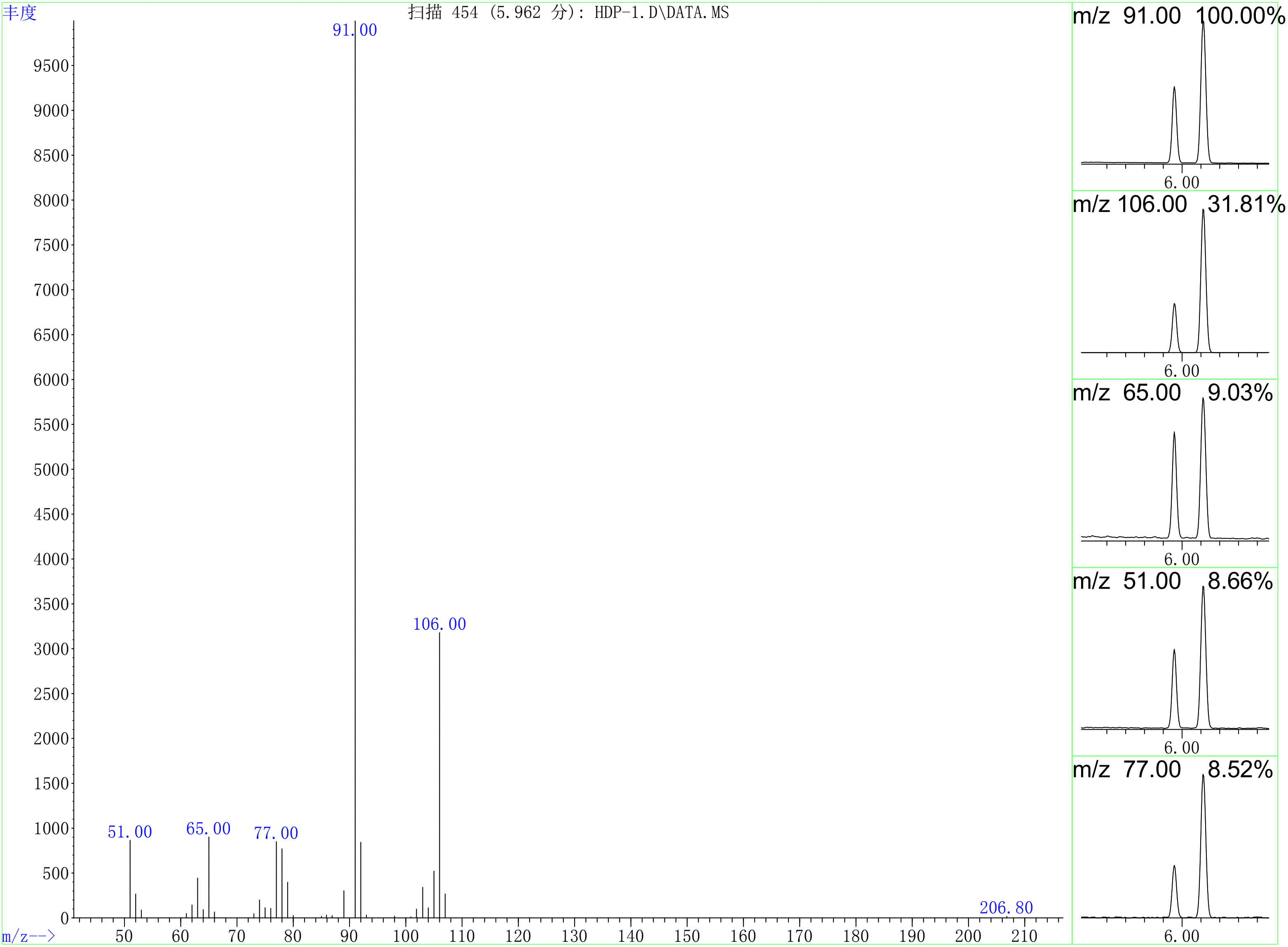

Data File: D:\GYM\DATA\2025\20251105\HDP-1.D
样品：HDP-1

峰编号： 1 5.962 分钟处 面积： 5595585 面积 % 0.16

每个谱库中 3 个最匹配的记录。 Ref\# CAS\# 匹配度

C:\database\DEMO.L 未检索到匹配。

未知谱图基于顶点

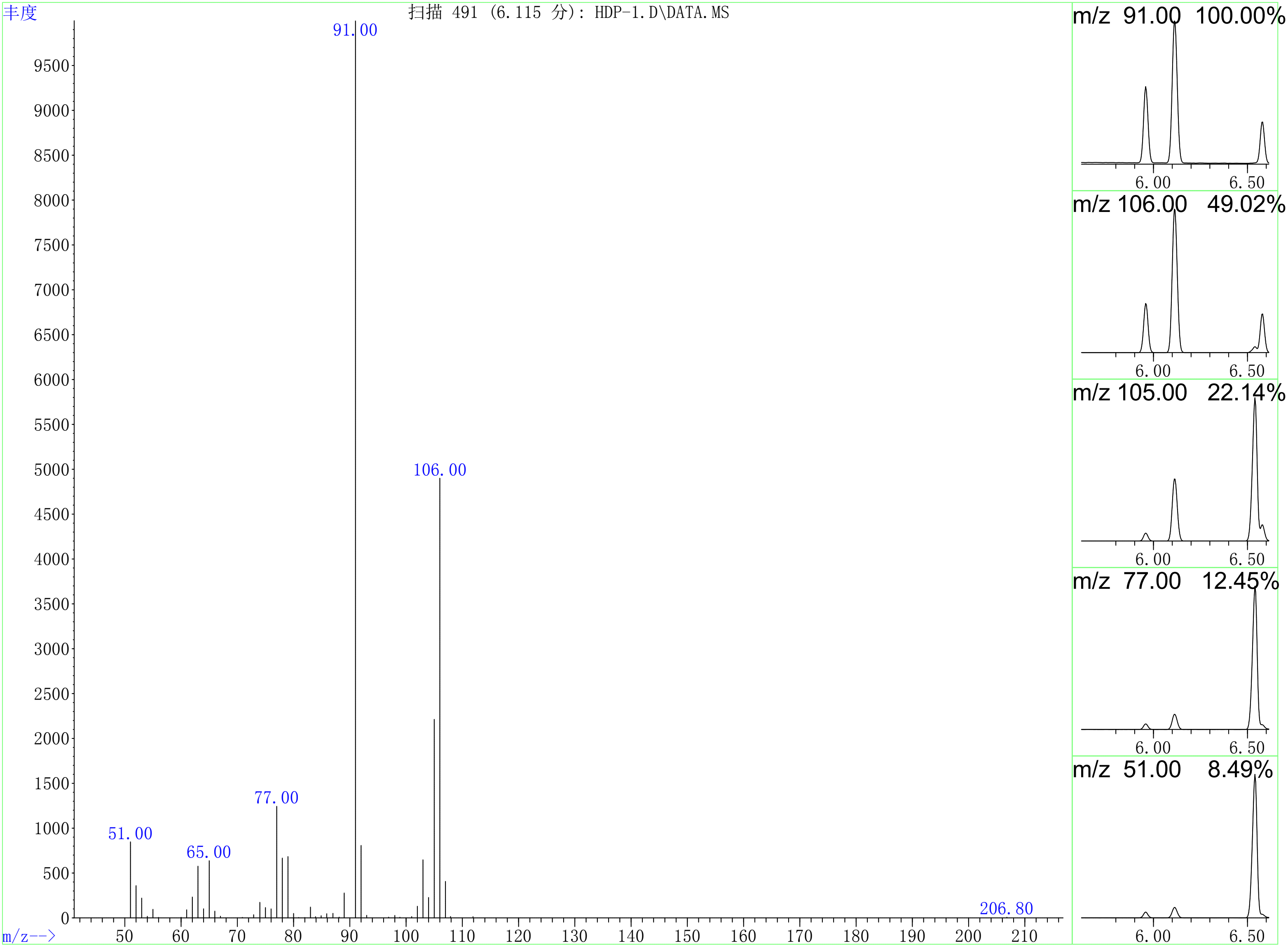

Data File: D:\GYM\DATA\2025\20251105\HDP-1.D
样品：HDP-1

峰编号： 2 6.115 分钟处 面积： 14723998 面积 % 0.43

每个谱库中 3 个最匹配的记录。 Ref\# CAS\# 匹配度

C:\database\DEMO.L 未检索到匹配。

未知谱图基于顶点

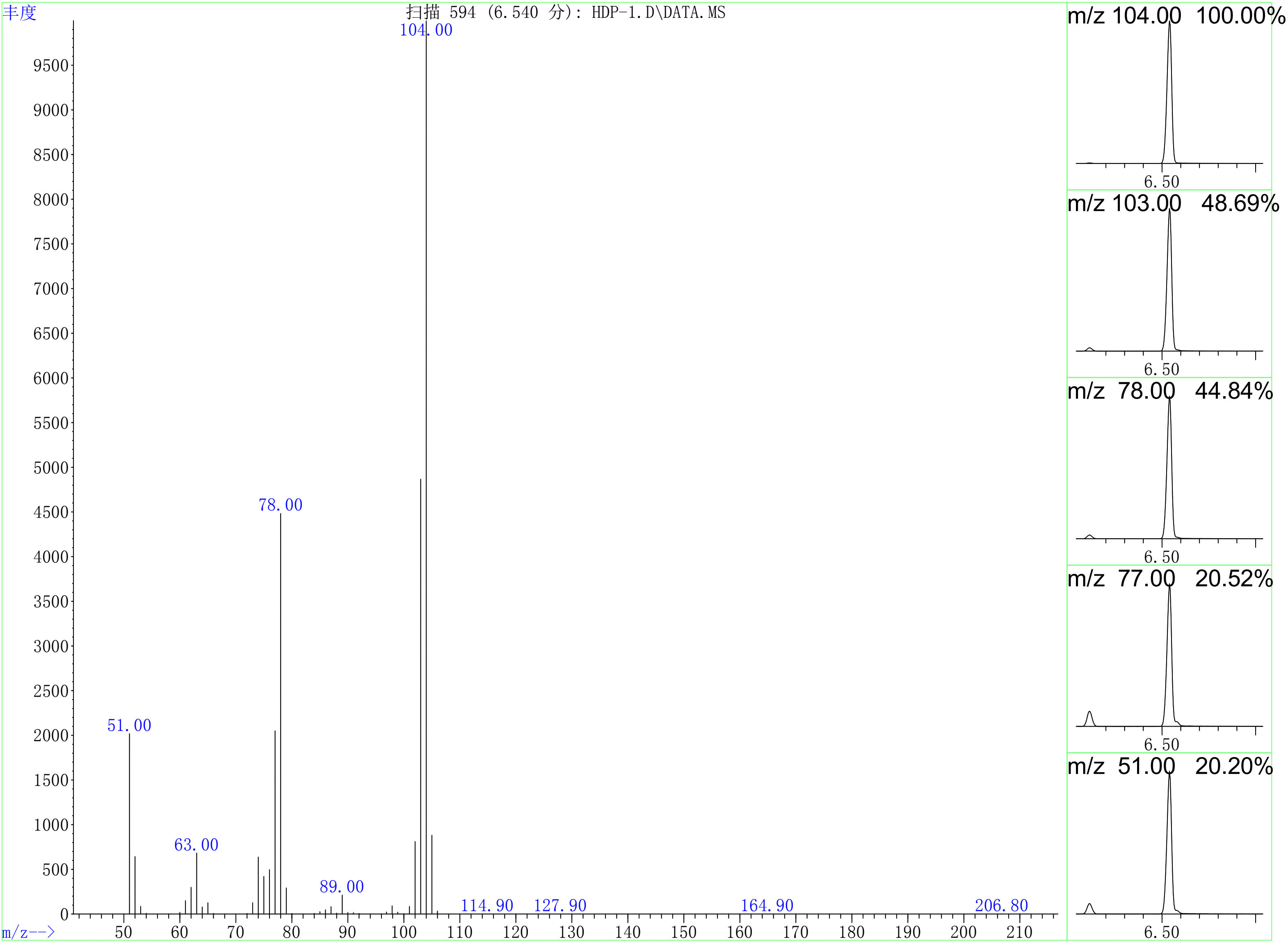

Data File: D:\GYM\DATA\2025\20251105\HDP-1.D
样品：HDP-1

峰编号： 3 6.540 分钟处 面积： 95065576 面积 % 2.76

每个谱库中 3 个最匹配的记录。 Ref\# CAS\# 匹配度

C:\database\DEMO.L 未检索到匹配。

未知谱图基于顶点

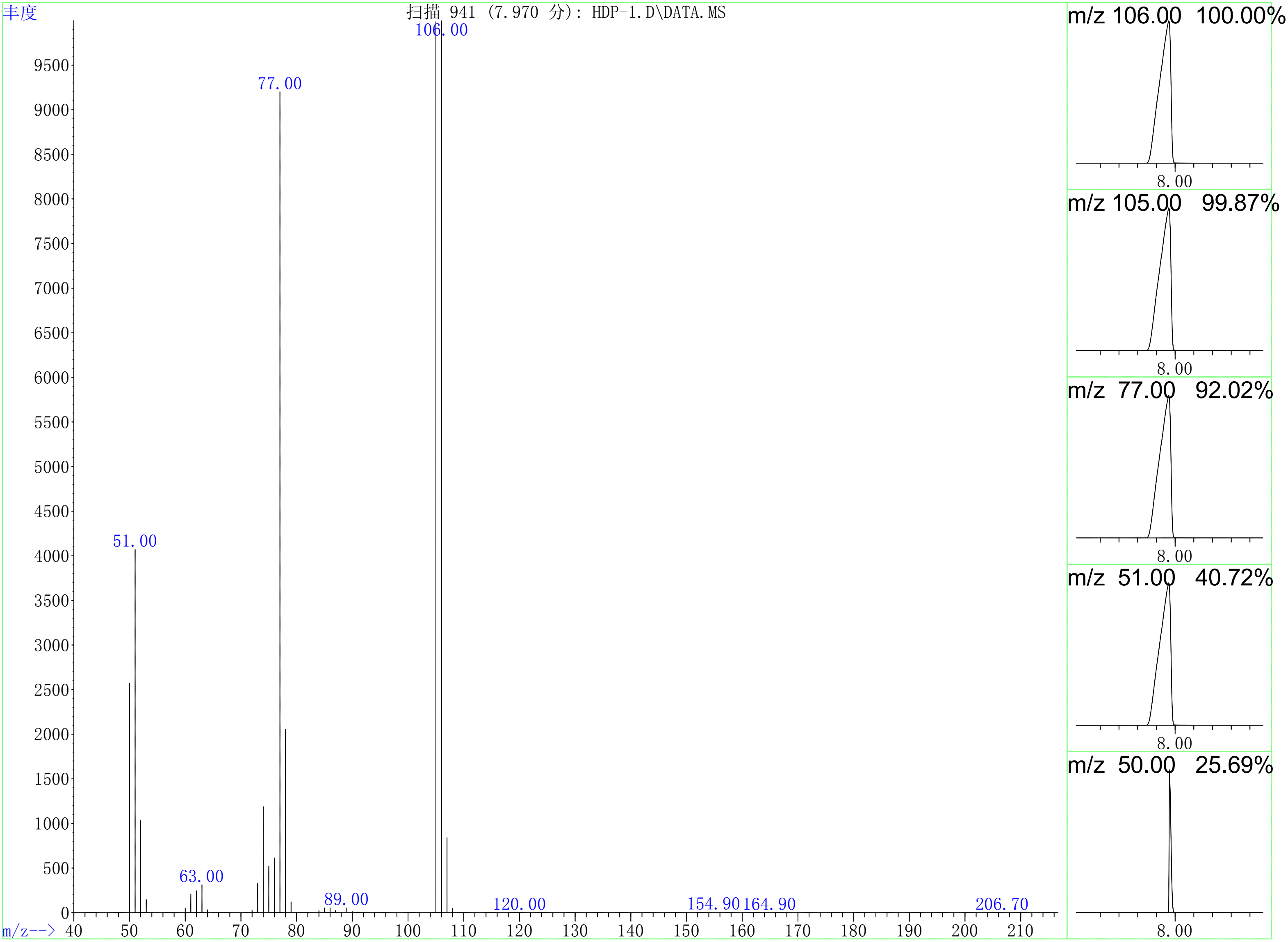

Data File: D:\GYM\DATA\2025\20251105\HDP-1.D
样品：HDP-1

峰编号： 4 7.970 分钟处 面积：560810317 面积 % 16.31

每个谱库中 3 个最匹配的记录。 Ref\# CAS\# 匹配度

C:\database\DEMO.L 未检索到匹配。

未知谱图基于顶点

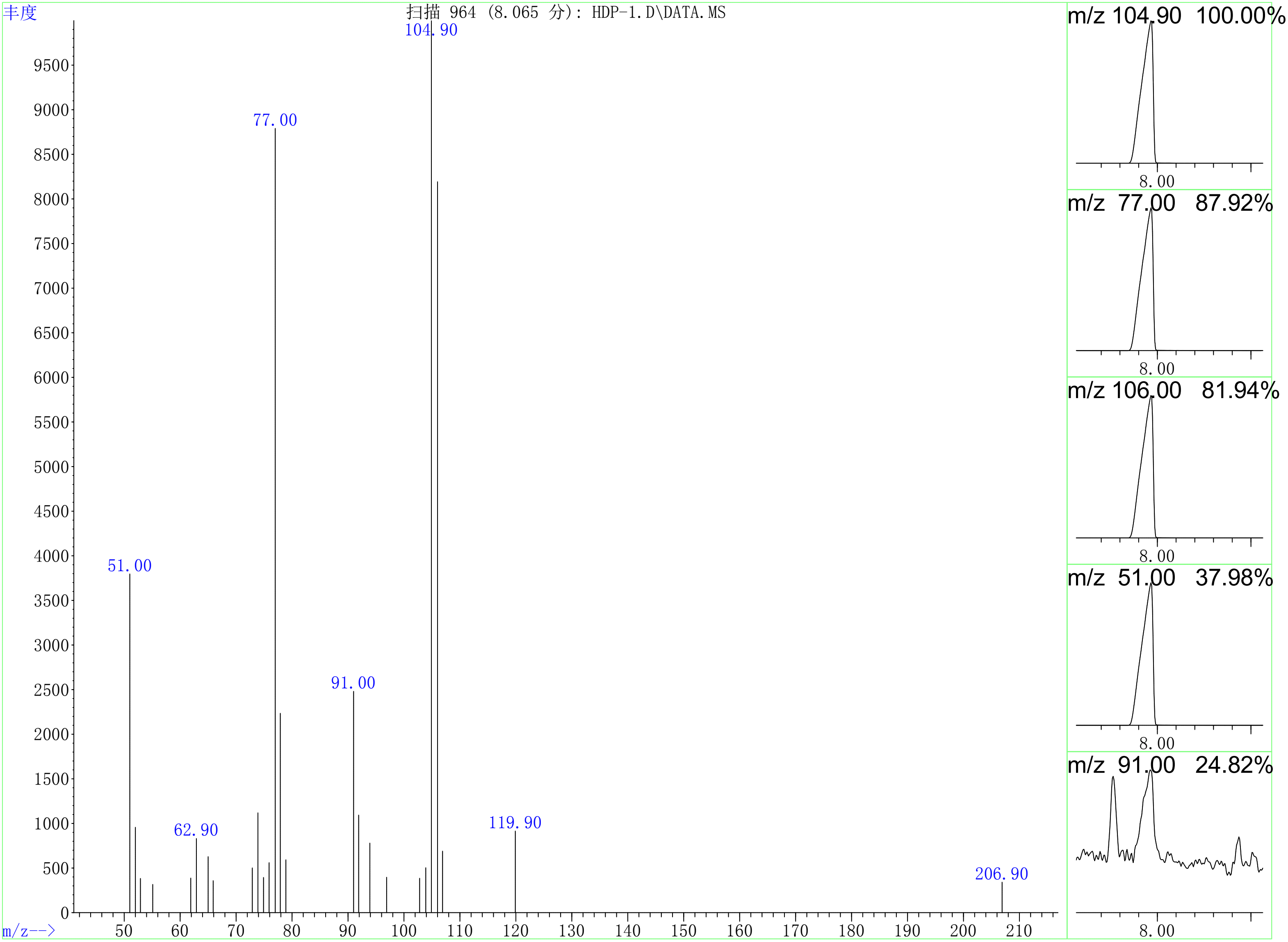

Data File: D:\GYM\DATA\2025\20251105\HDP-1.D
样品：HDP-1

峰编号： 5 8.065 分钟处 面积： 366327 面积 % 0.01

每个谱库中 3 个最匹配的记录。 Ref\# CAS\# 匹配度

C:\database\DEMO.L 未检索到匹配。

未知谱图基于顶点

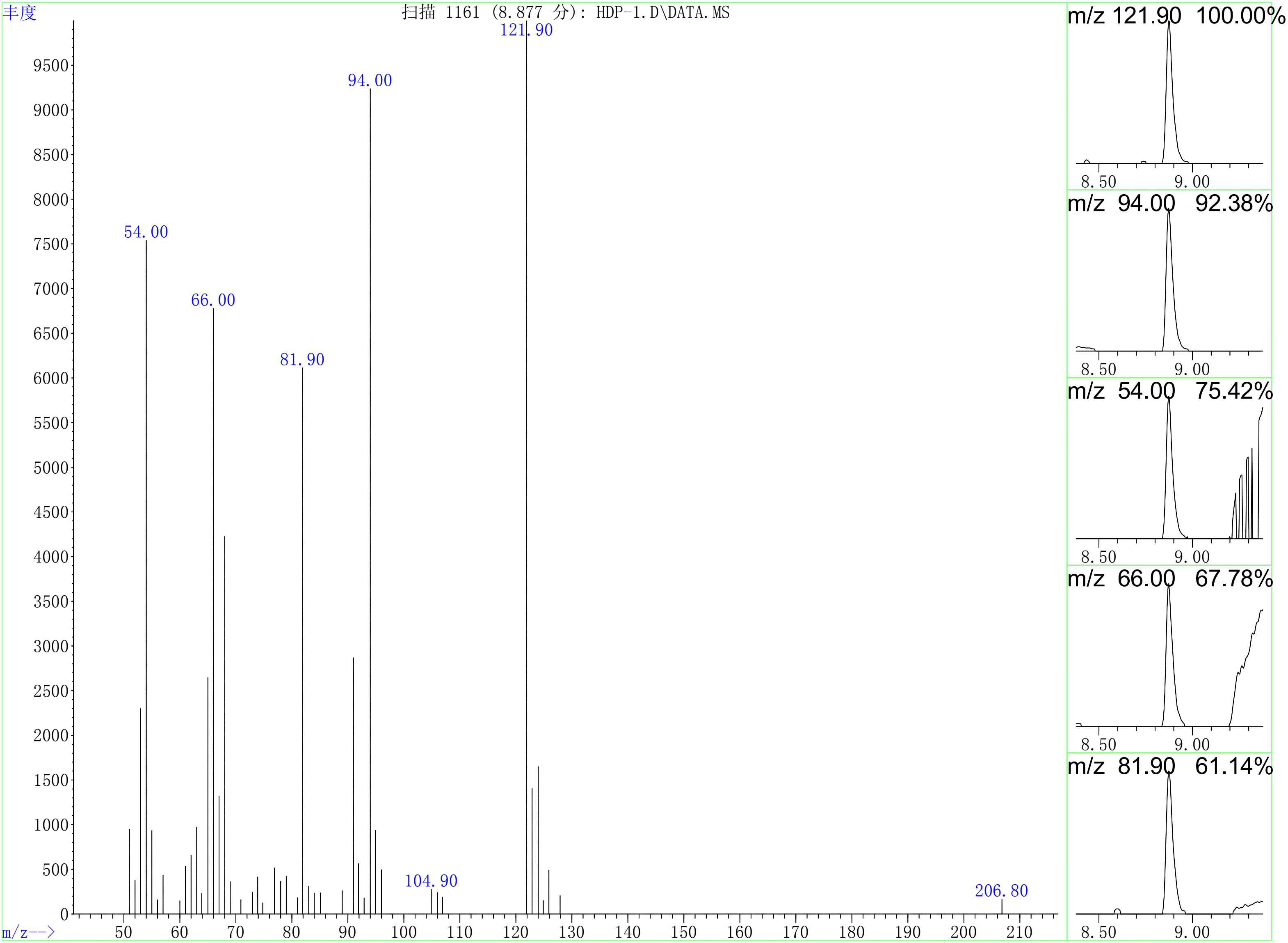

Data File: D:\GYM\DATA\2025\20251105\HDP-1.D
样品：HDP-1

峰编号： 6 8.877 分钟处 面积： 2331626 面积 % 0.07

每个谱库中 3 个最匹配的记录。 Ref\# CAS\# 匹配度

C:\database\DEMO.L 未检索到匹配。

未知谱图基于顶点

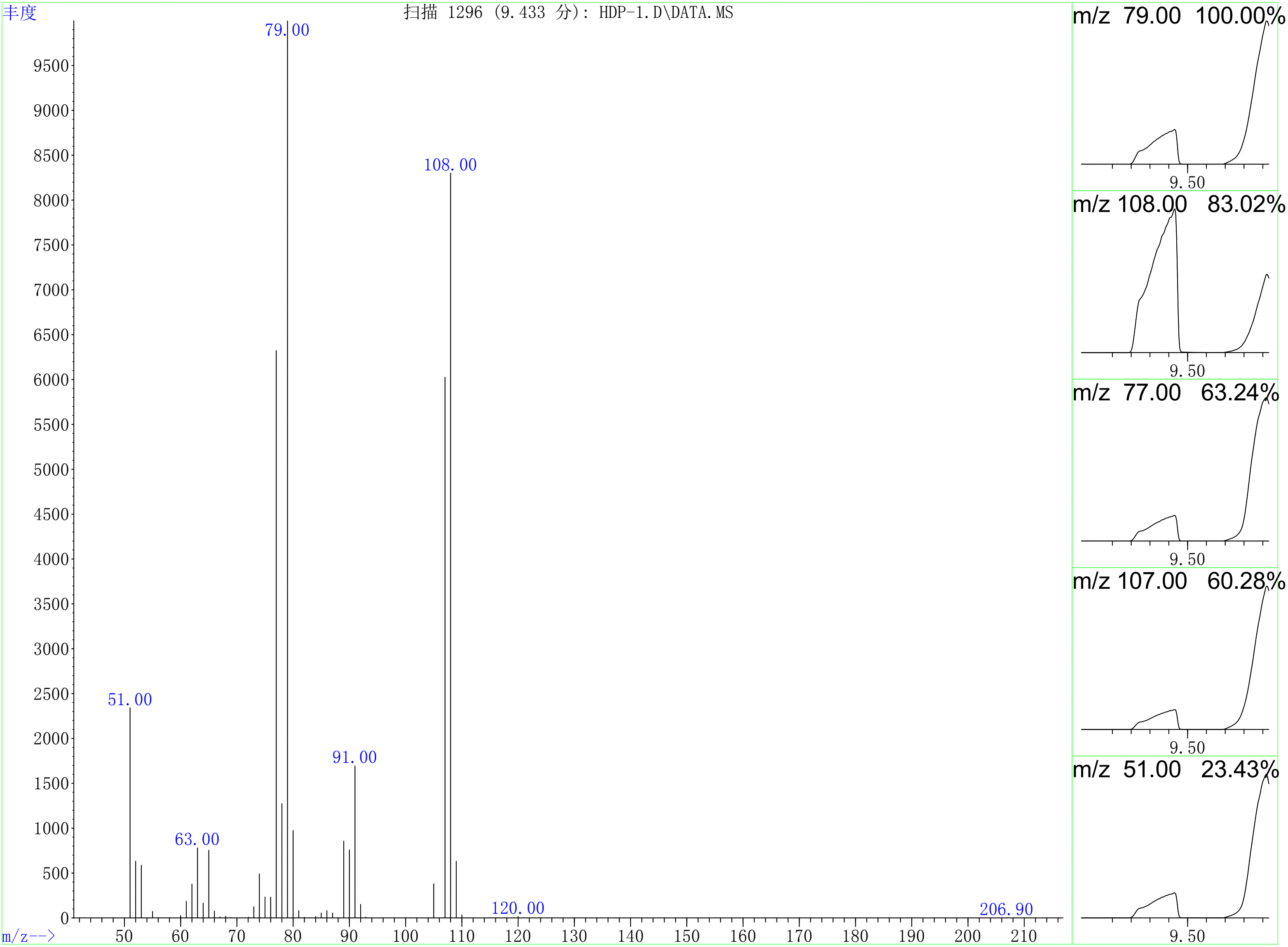

Data File: D:\GYM\DATA\2025\20251105\HDP-1.D
样品：HDP-1

峰编号： 7 9.433 分钟处 面积：495364402 面积 % 14.40

每个谱库中 3 个最匹配的记录。 Ref\# CAS\# 匹配度

C:\database\DEMO.L 未检索到匹配。

未知谱图基于顶点

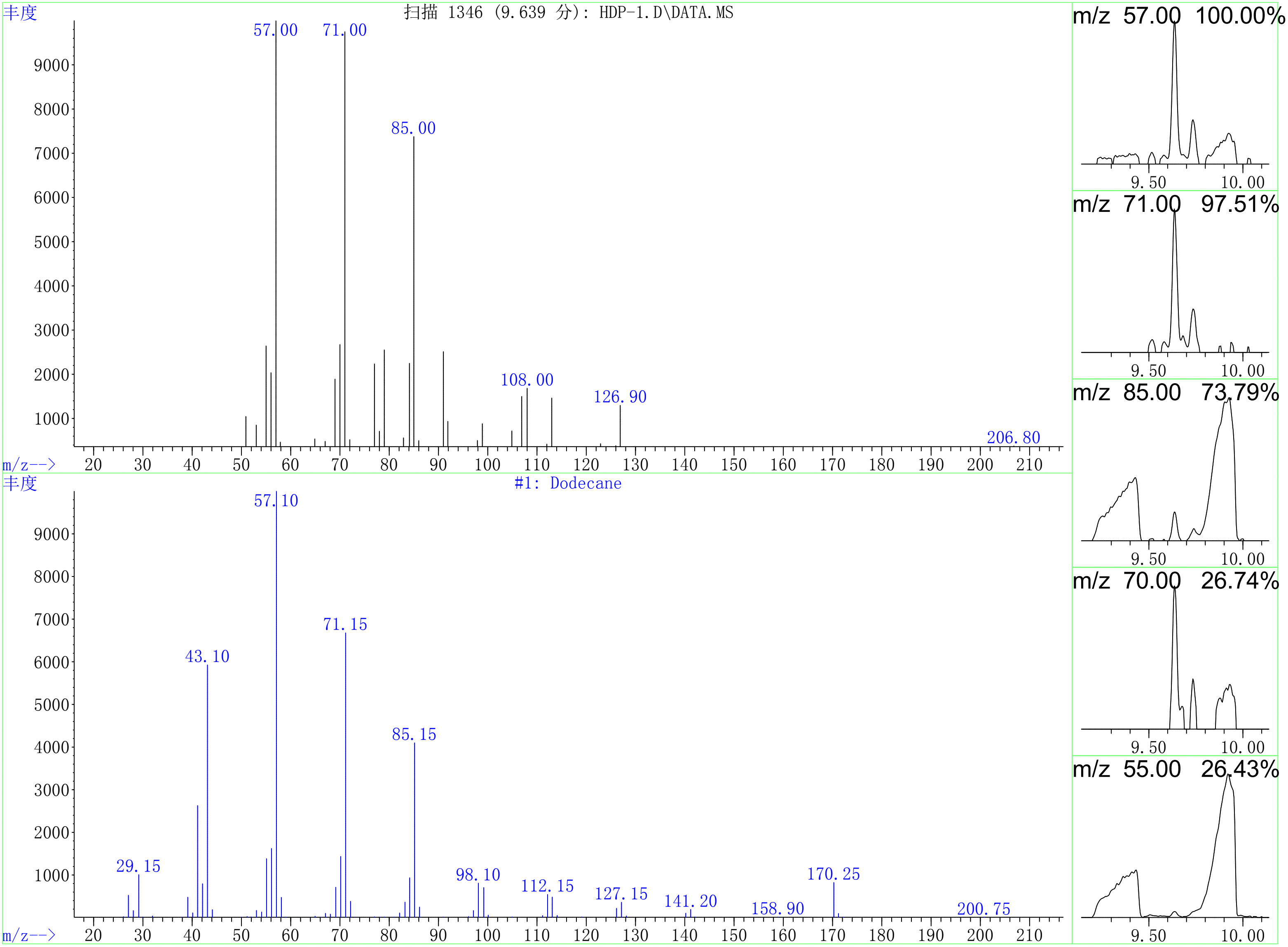

Data File: D:\GYM\DATA\2025\20251105\HDP-1.D
样品：HDP-1

峰编号： 8　9.639 分钟处　面积：　354801　面积 % 0.01

每个谱库中 3 个最匹配的记录。	Ref\#	CAS\#	匹配度
C:\database\DEMO.L			
1 Dodecane	1	000112-40-3	40

未知谱图基于顶点

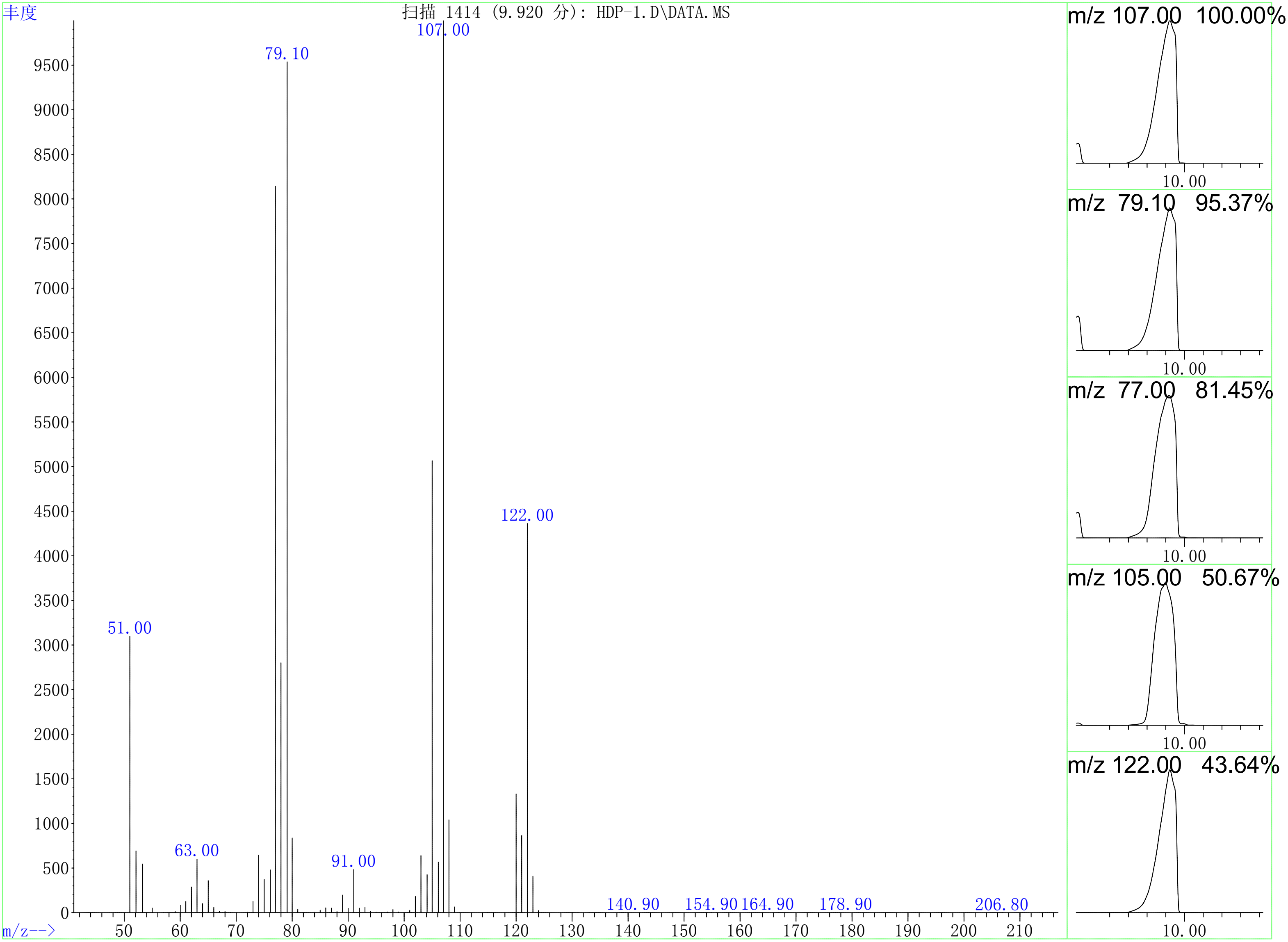

Data File: D:\GYM\DATA\2025\20251105\HDP-1.D
样品：HDP-1

峰编号： 9 9.920 分钟处 面积：1972043092 面积 % 57.34

每个谱库中 3 个最匹配的记录。 Ref\# CAS\# 匹配度

C:\database\DEMO.L 未检索到匹配。

未知谱图基于顶点

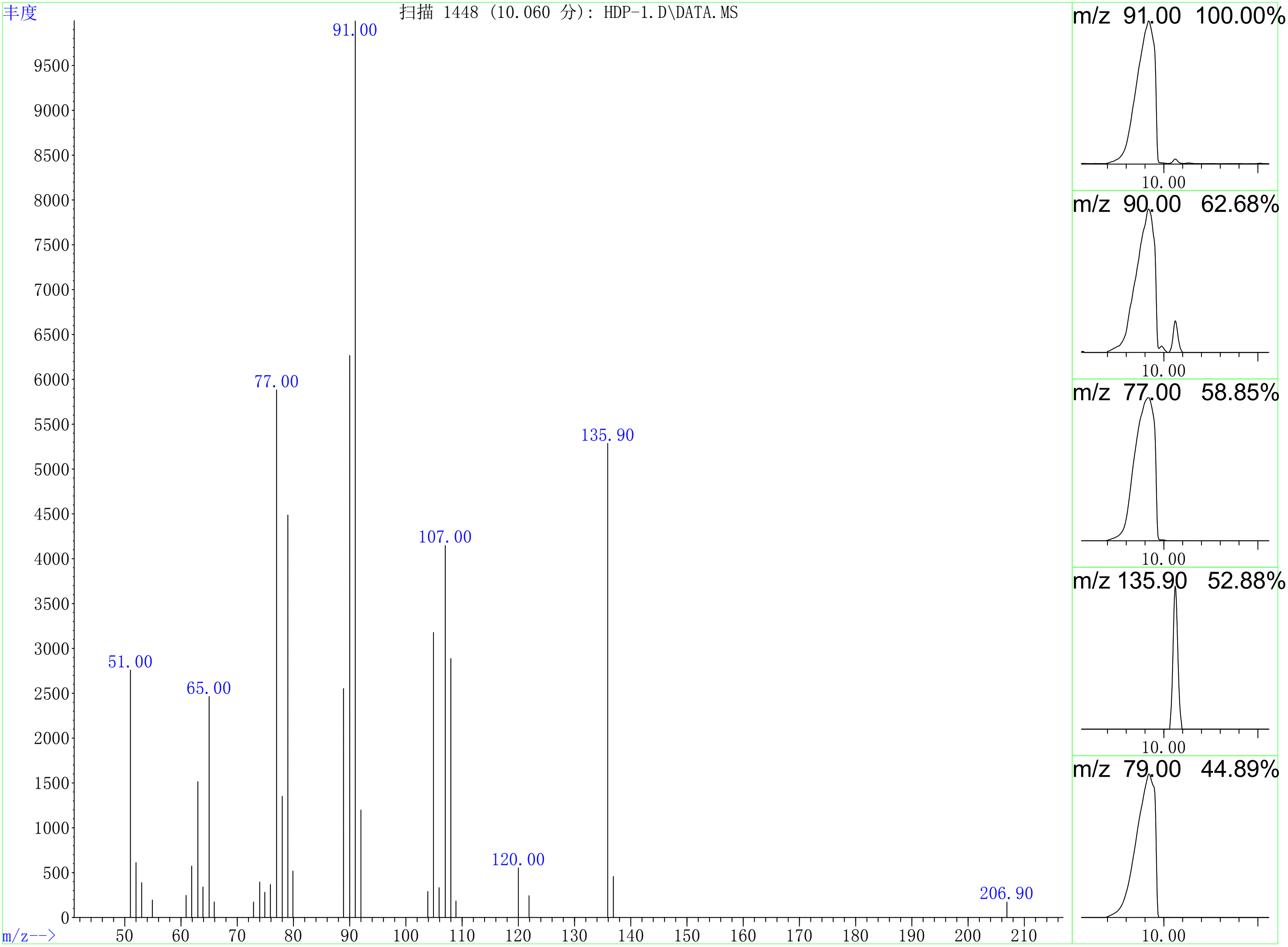

Data File: D:\GYM\DATA\2025\20251105\HDP-1.D
样品：HDP-1

峰编号：10　　10.060 分钟处　面积：　916000　面积 % 0.03

每个谱库中 3 个最匹配的记录。　　Ref\#　CAS\#　匹配度

C:\database\DEMO.L　未检索到匹配。

未知谱图基于顶点

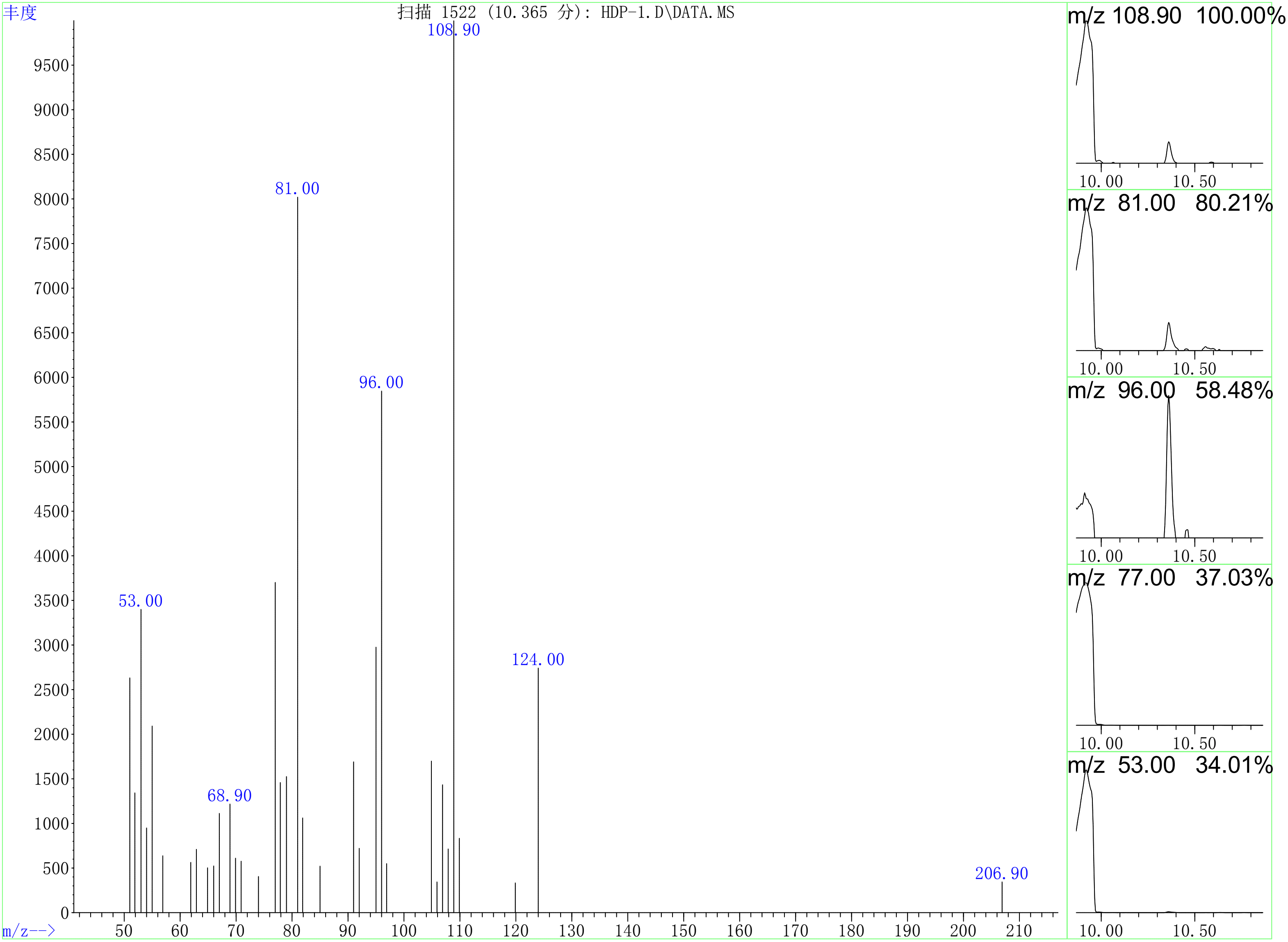

Data File: D:\GYM\DATA\2025\20251105\HDP-1.D
样品：HDP-1

峰编号： 11 10.365 分钟处 面积： 623766 面积 % 0.02

每个谱库中 3 个最匹配的记录。 Ref\# CAS\# 匹配度

C:\database\DEMO.L 未检索到匹配。

未知谱图基于顶点

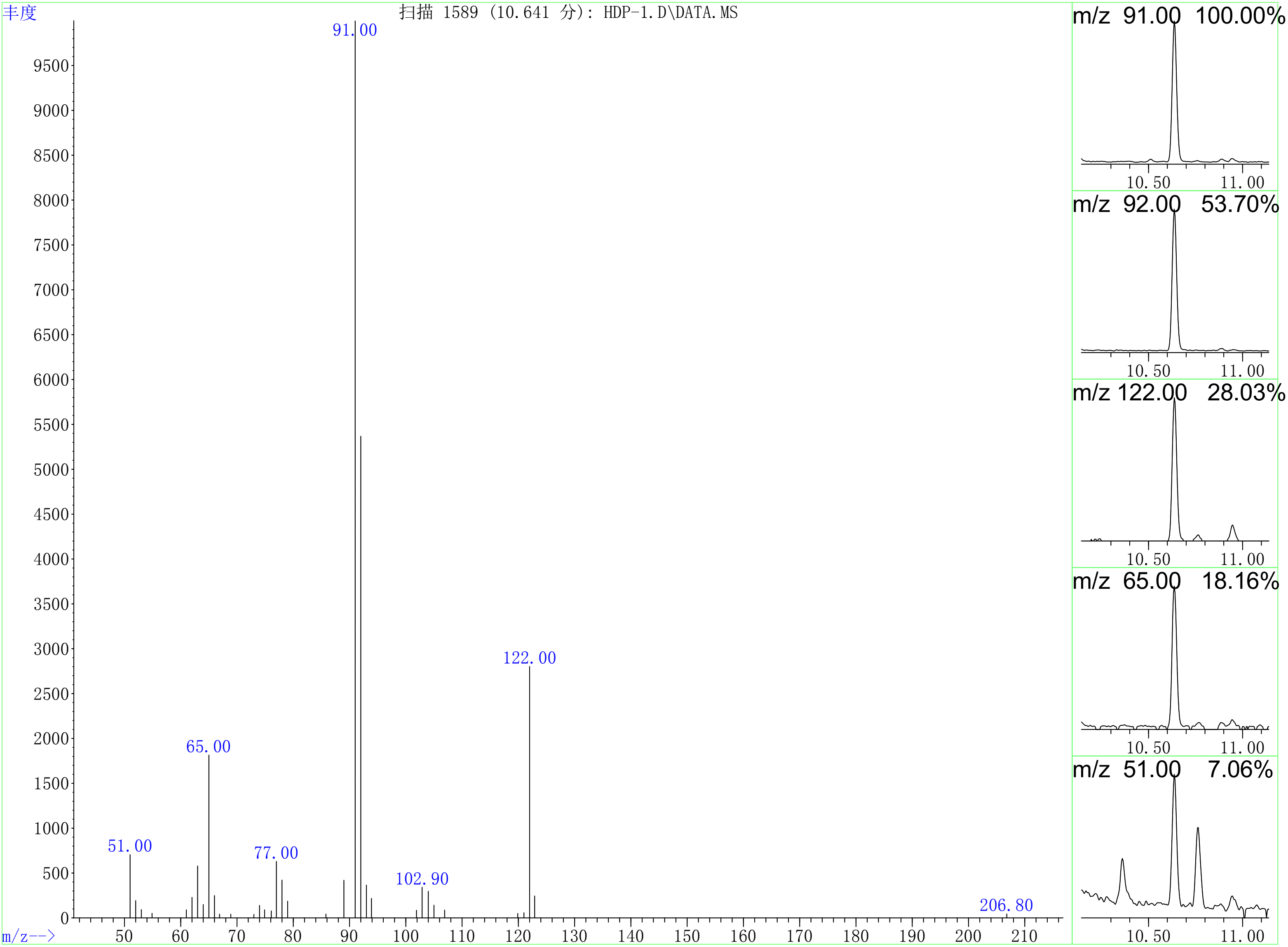

Data File: D:\GYM\DATA\2025\20251105\HDP-1.D
样品：HDP-1

峰编号： 12 10.641 分钟处 面积： 1950190 面积 % 0.06

每个谱库中 3 个最匹配的记录。 Ref\# CAS\# 匹配度

C:\database\DEMO.L 未检索到匹配。

未知谱图基于顶点

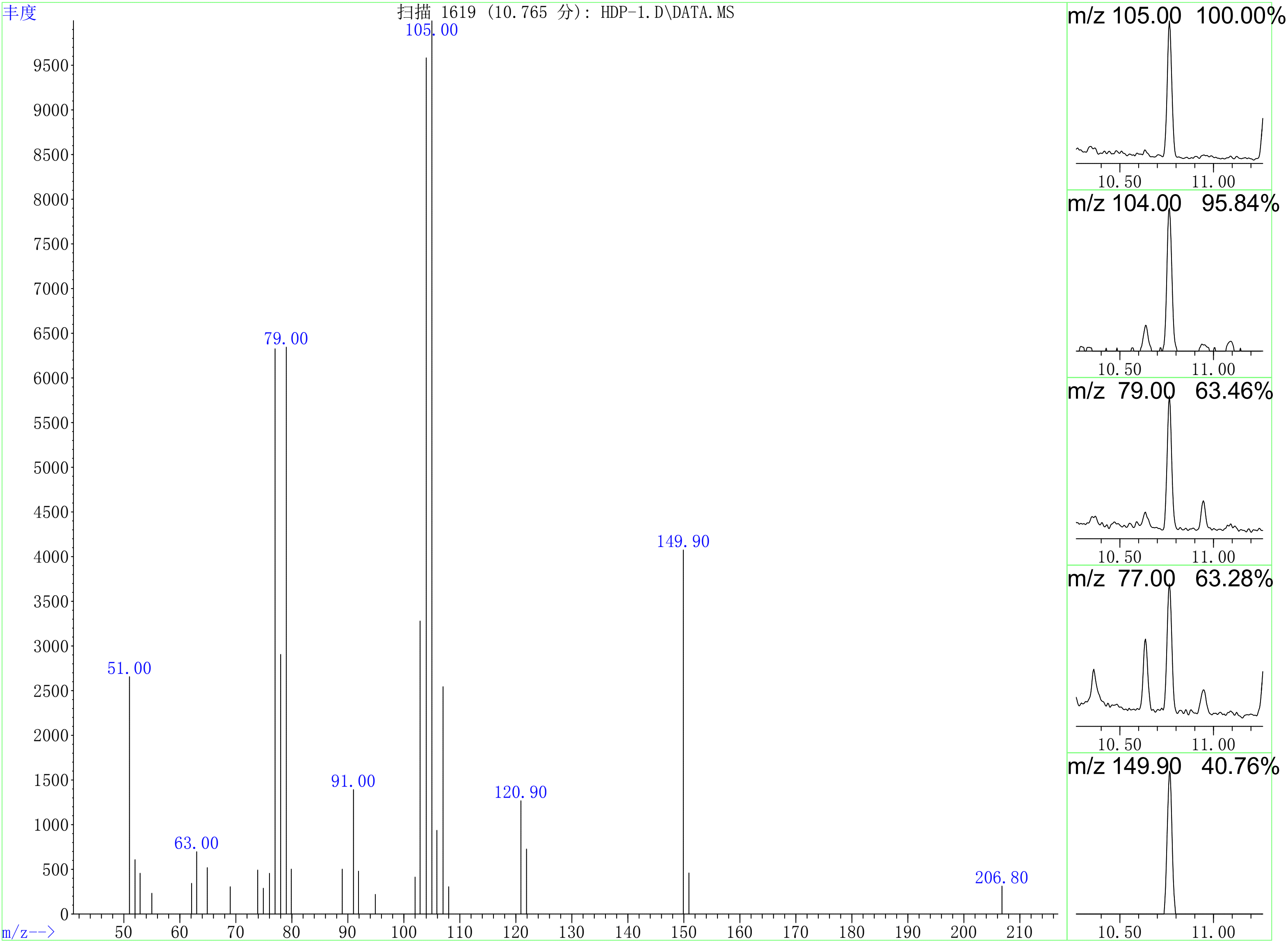

Data File: D:\GYM\DATA\2025\20251105\HDP-1.D
样品：HDP-1

峰编号： 13 10.765 分钟处 面积： 736853 面积 % 0.02

每个谱库中 3 个最匹配的记录。 Ref\# CAS\# 匹配度

C:\database\DEMO.L 未检索到匹配。

未知谱图基于顶点

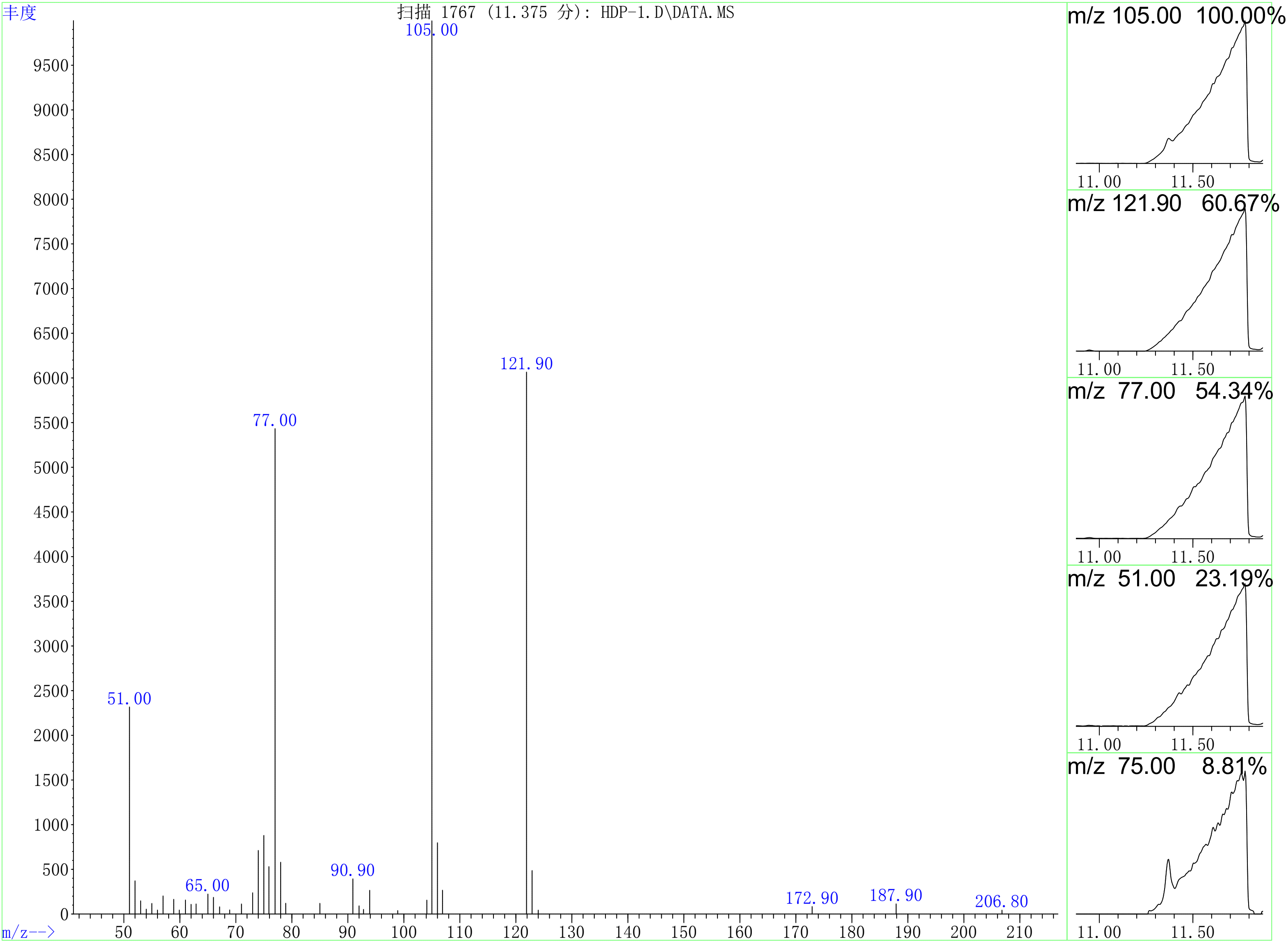

Data File: D:\GYM\DATA\2025\20251105\HDP-1.D
样品：HDP-1

峰编号： 14 11.375 分钟处 面积： 4167005 面积 % 0.12

每个谱库中 3 个最匹配的记录。	Ref\#	CAS\#	匹配度
C:\database\DEMO.L 未检索到匹配。			

未知谱图基于顶点

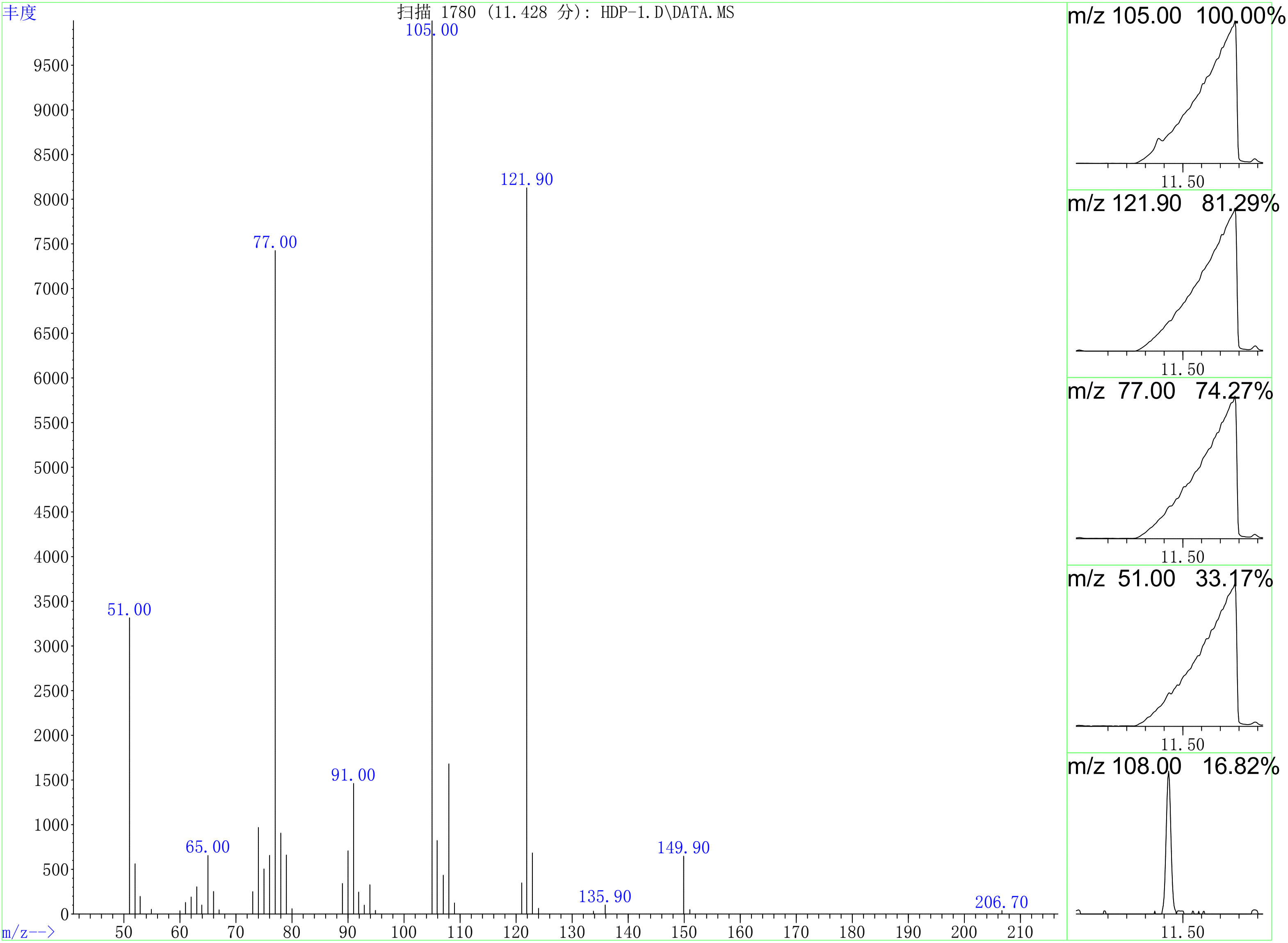

Data File: D:\GYM\DATA\2025\20251105\HDP-1.D
样品：HDP-1

峰编号： 15   11.428 分钟处 面积： 5622574 面积 % 0.16

每个谱库中 3 个最匹配的记录。   Ref\# CAS\# 匹配度

C:\database\DEMO.L 未检索到匹配。

未知谱图基于顶点

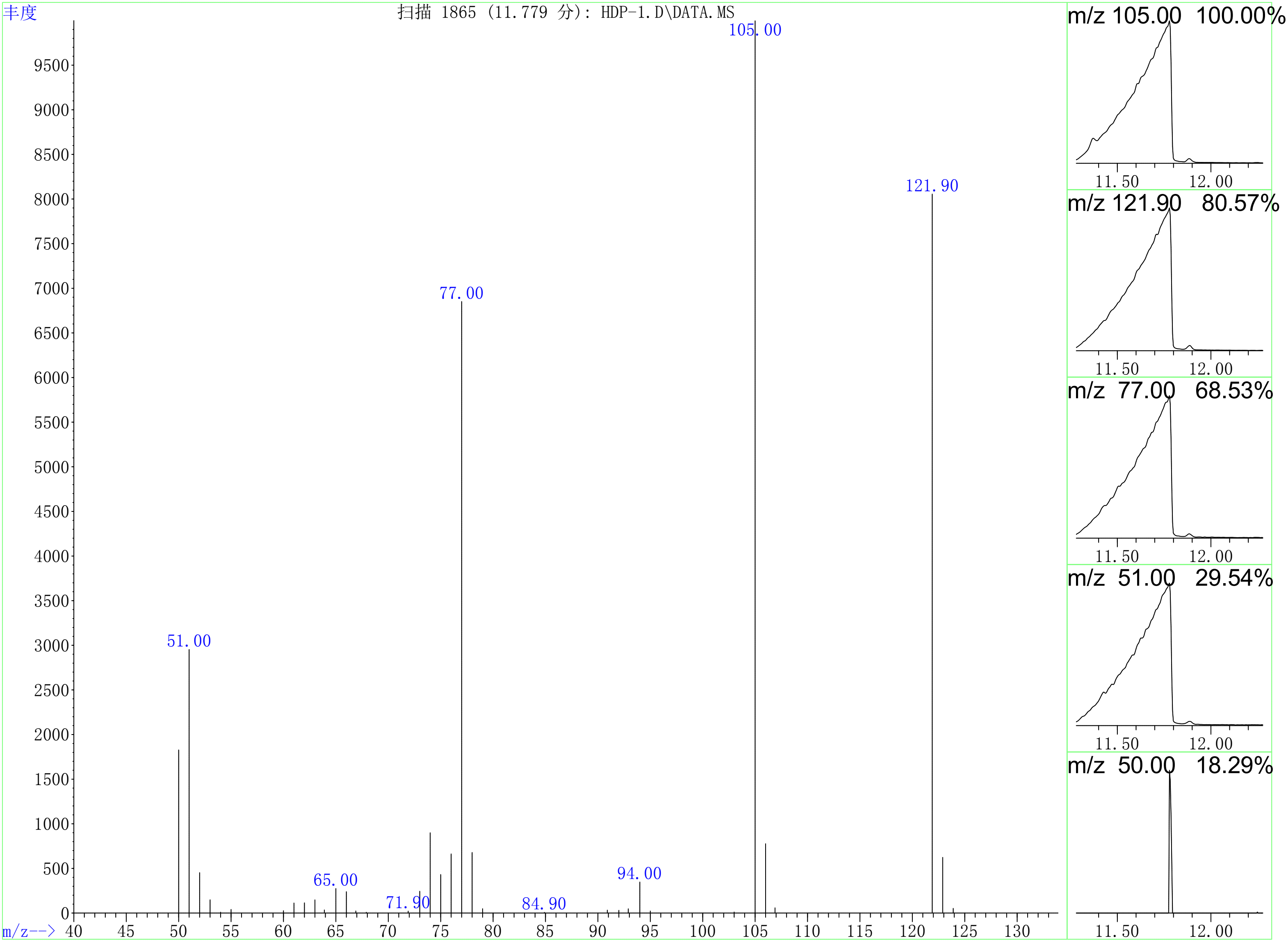

Data File: D:\GYM\DATA\2025\20251105\HDP-1.D
样品：HDP-1

峰编号： 16 11.779 分钟处 面积： 91089110 面积 % 2.65

每个谱库中 3 个最匹配的记录。	Ref\#	CAS\#	匹配度
C:\database\DEMO.L 未检索到匹配。			

未知谱图基于顶点

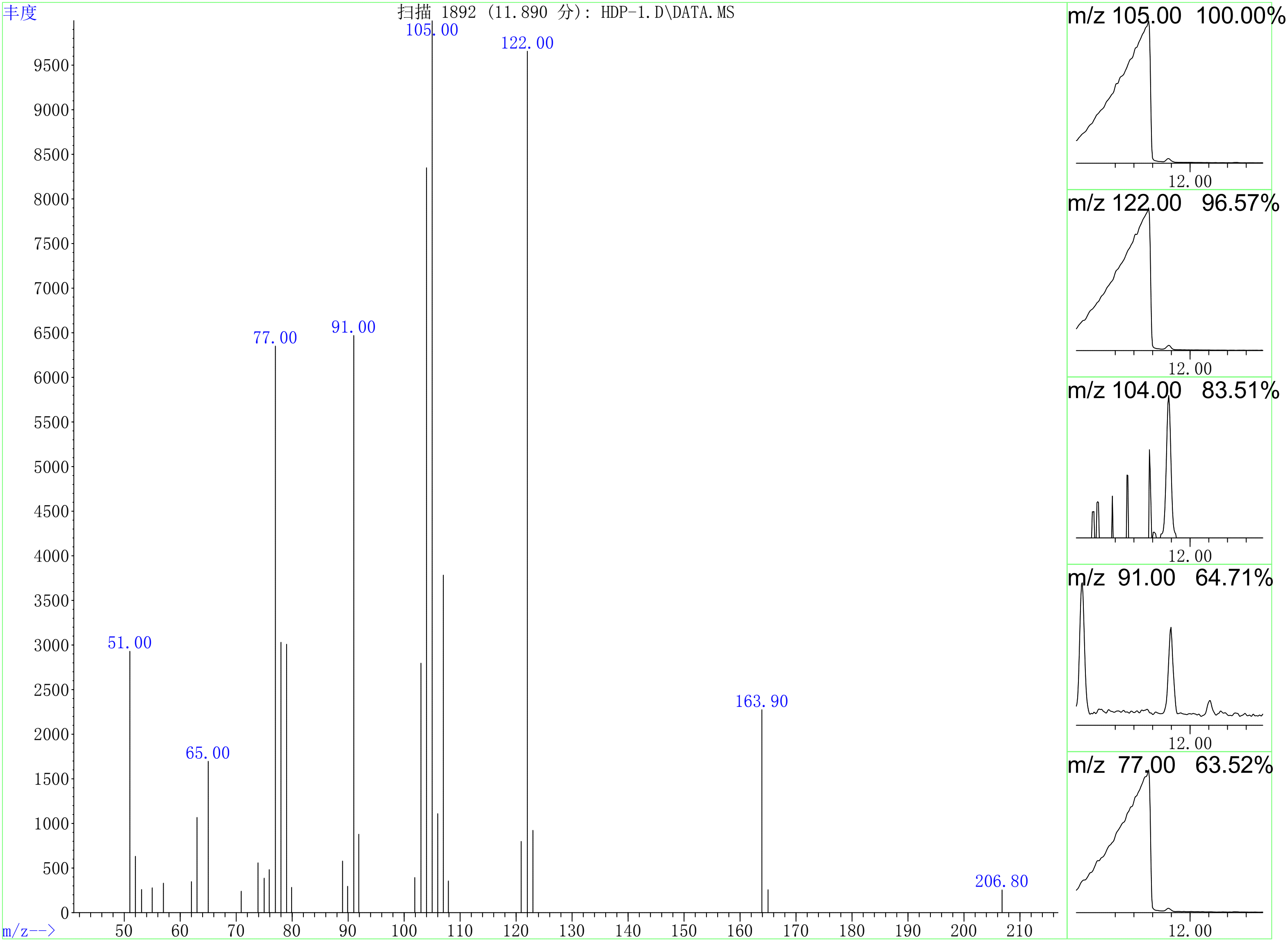

Data File: D:\GYM\DATA\2025\20251105\HDP-1.D
样品：HDP-1

峰编号： 17 11.890 分钟处 面积： 907487 面积 % 0.03

每个谱库中 3 个最匹配的记录。 Ref\# CAS\# 匹配度

C:\database\DEMO.L 未检索到匹配。

未知谱图基于顶点

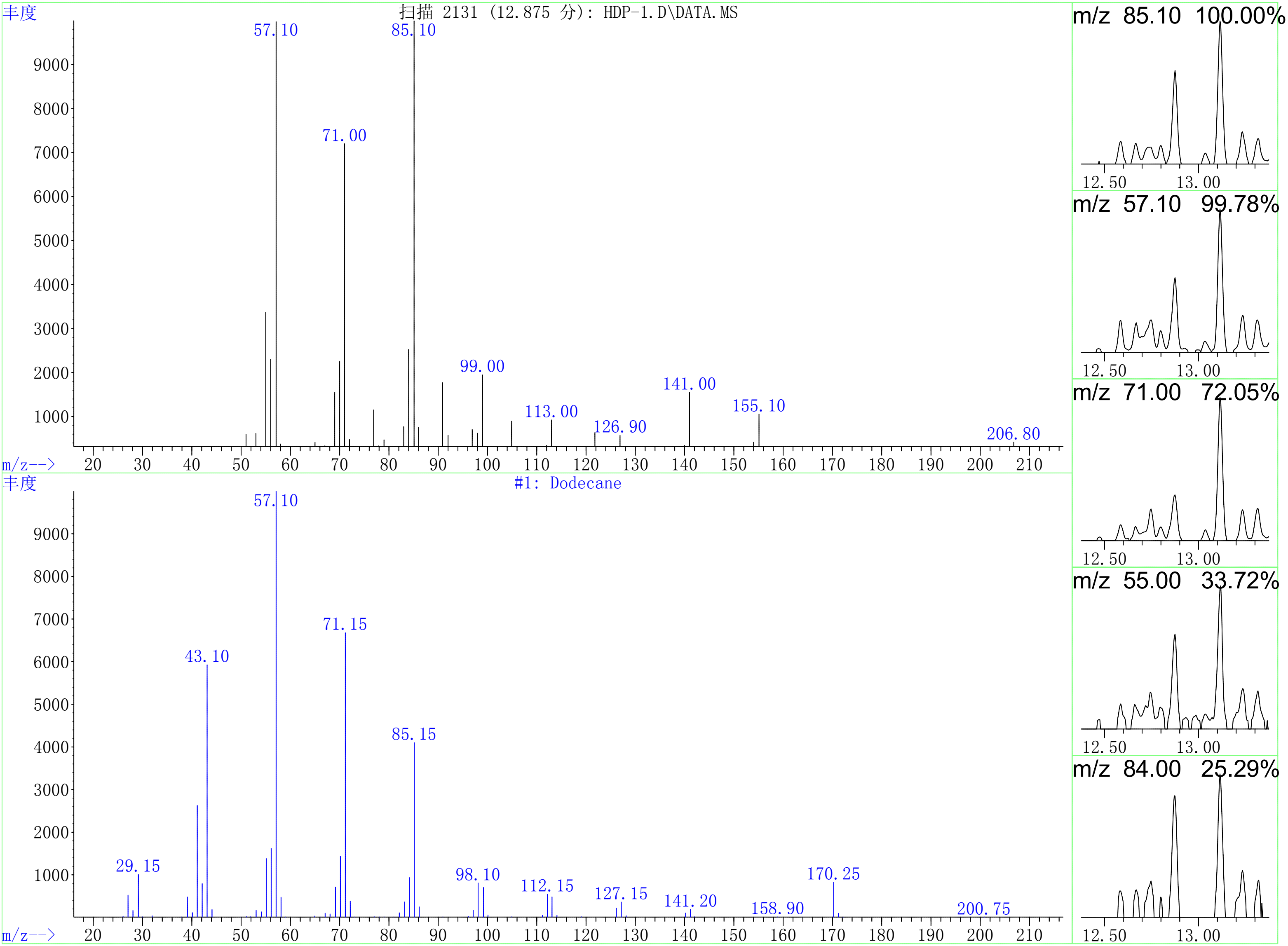

Data File: D:\GYM\DATA\2025\20251105\HDP-1.D
样品：HDP-1

峰编号： 18 12.875 分钟处 面积： 484390 面积 % 0.01

每个谱库中 3 个最匹配的记录。	Ref\#	CAS\#	匹配度
C:\database\DEMO.L			
1 Dodecane	1	000112-40-3	40

未知谱图基于顶点

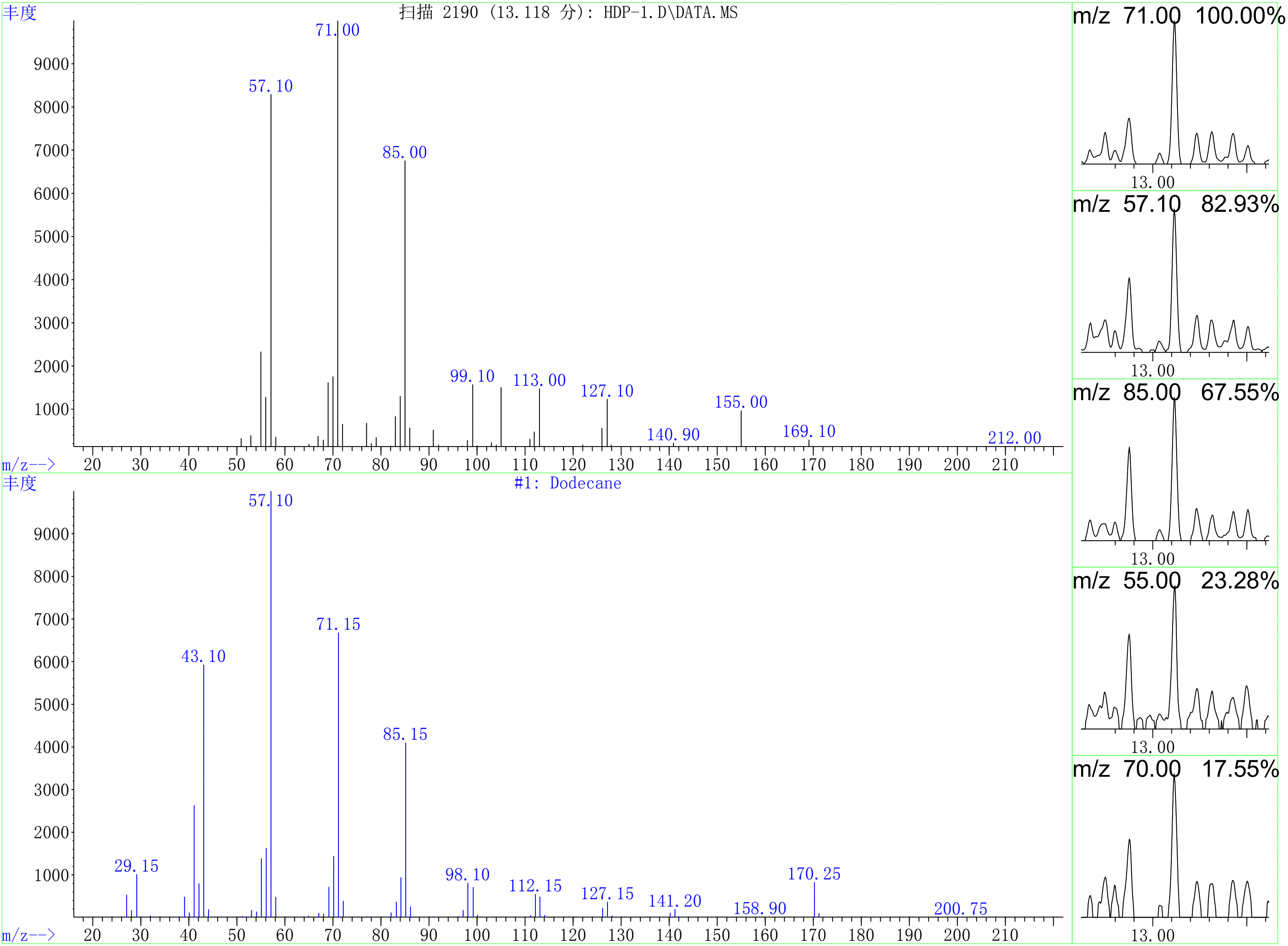

Data File: D:\GYM\DATA\2025\20251105\HDP-1.D
样品：HDP-1

峰编号： 19 13.118 分钟处 面积： 1914357 面积 % 0.06

每个谱库中 3 个最匹配的记录。	Ref\#	CAS\#	匹配度
C:\database\DEMO.L			
1 Dodecane	1	000112-40-3	72

未知谱图基于顶点

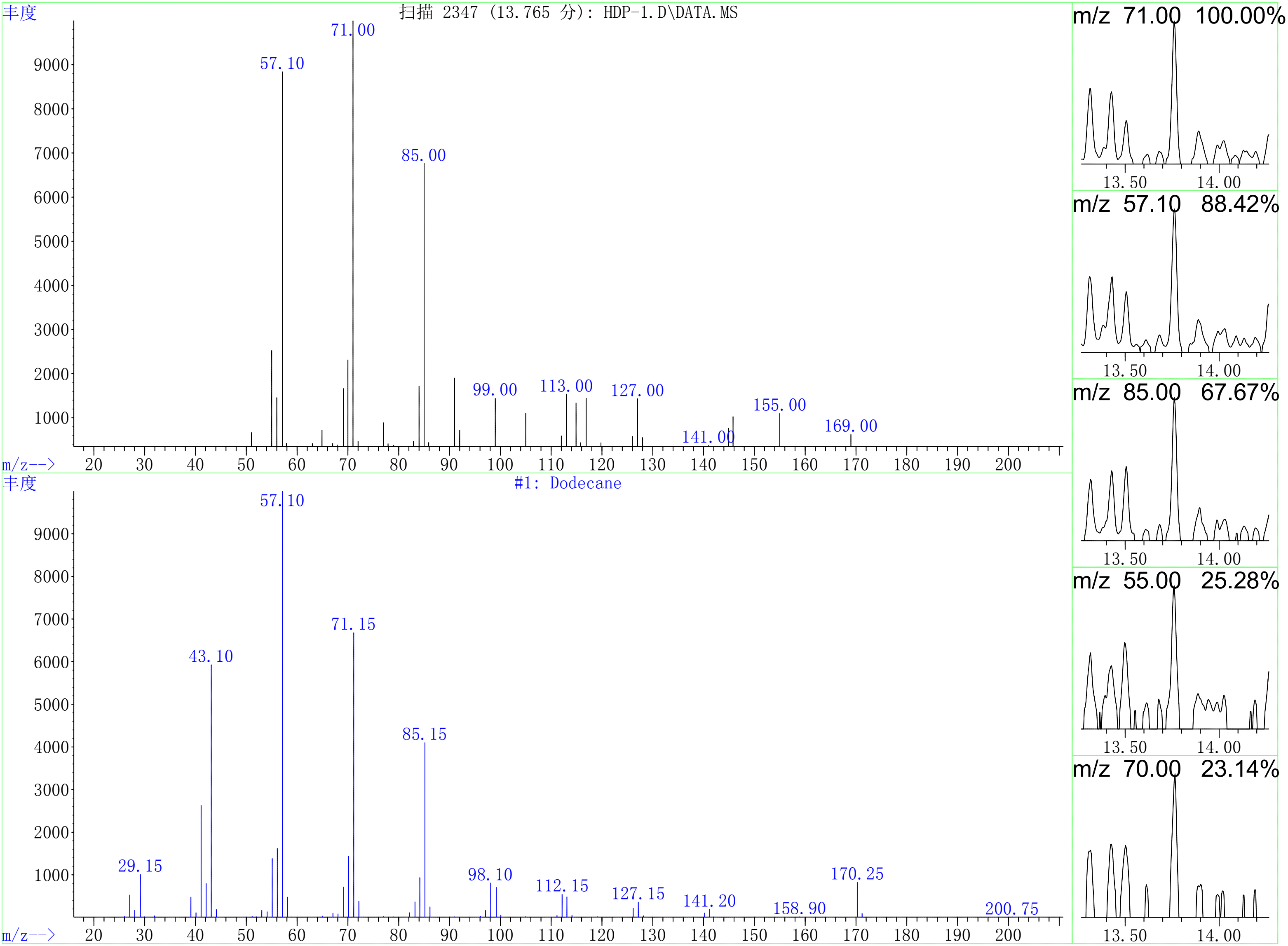

Data File: D:\GYM\DATA\2025\20251105\HDP-1.D
样品：HDP-1

峰编号： 20 13.765 分钟处 面积： 2116352 面积 % 0.06

每个谱库中 3 个最匹配的记录。	Ref\#	CAS\#	匹配度
C:\database\DEMO.L			
1 Dodecane	1	000112-40-3	50

谱库检索报告 - 化学工作站积分器

未知谱图基于顶点

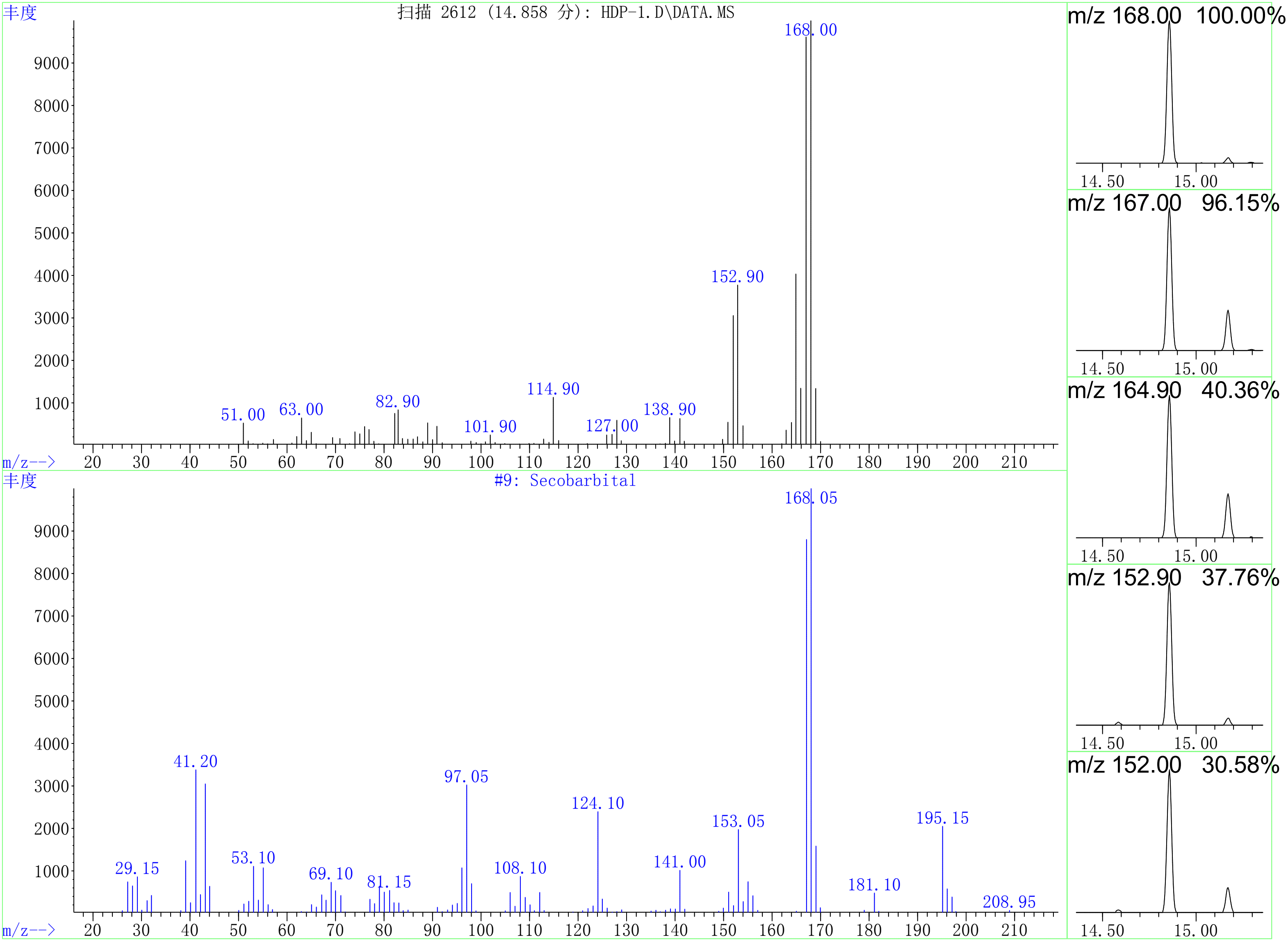

Data File: D:\GYM\DATA\2025\20251105\HDP-1.D
样品：HDP-1

峰编号： 21 14.858 分钟处 面积： 6179666 面积 % 0.18

每个谱库中 3 个最匹配的记录。	Ref\#	CAS\#	匹配度
C:\database\DEMO.L			
1 Secobarbital	9	000309-43-3	4

未知谱图基于顶点

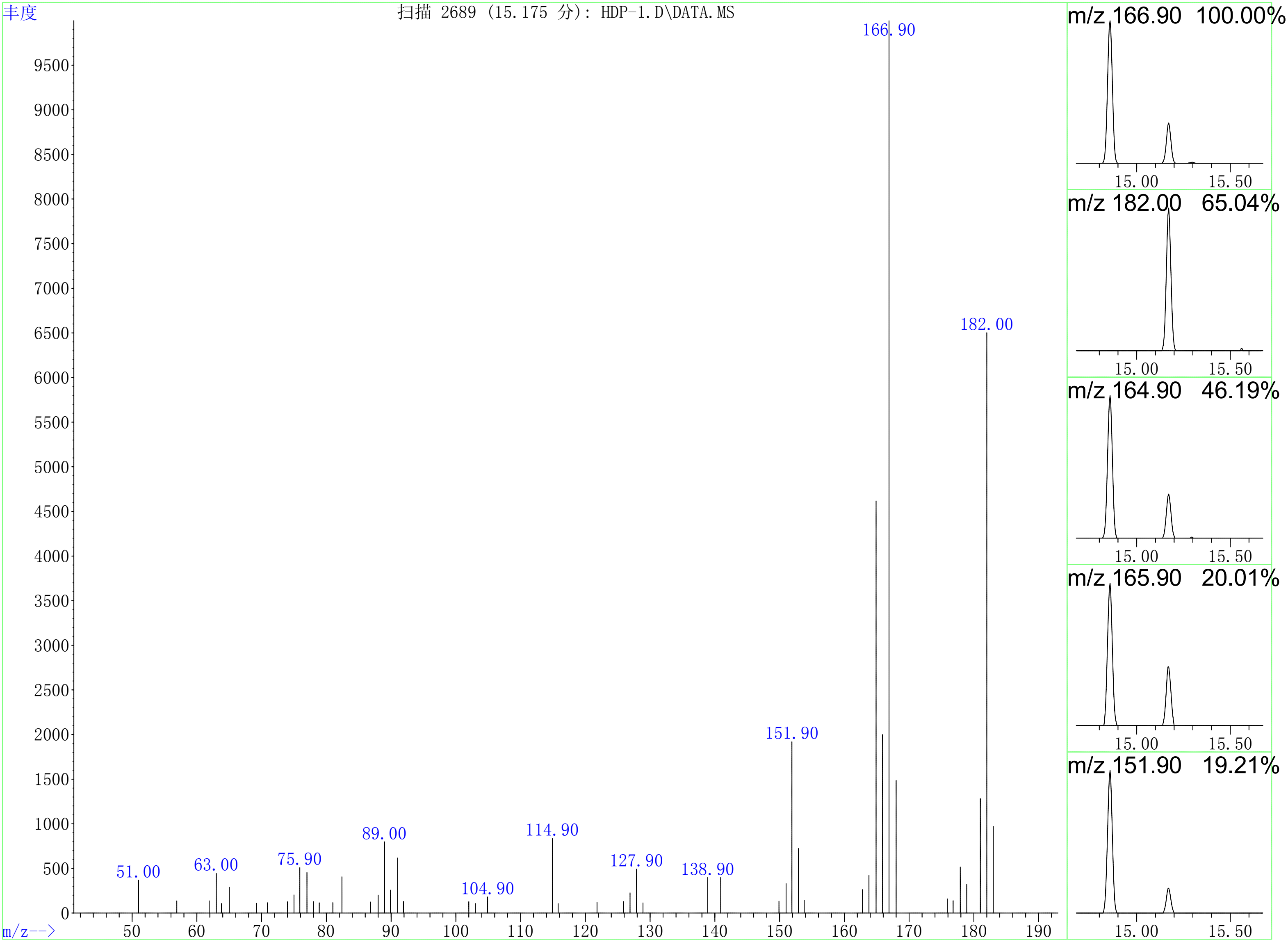

Data File: D:\GYM\DATA\2025\20251105\HDP-1.D
样品：HDP-1

峰编号： 22 15.175 分钟处 面积： 1247133 面积 % 0.04

每个谱库中 3 个最匹配的记录。 Ref\# CAS\# 匹配度

C:\database\DEMO.L 未检索到匹配。

未知谱图基于顶点

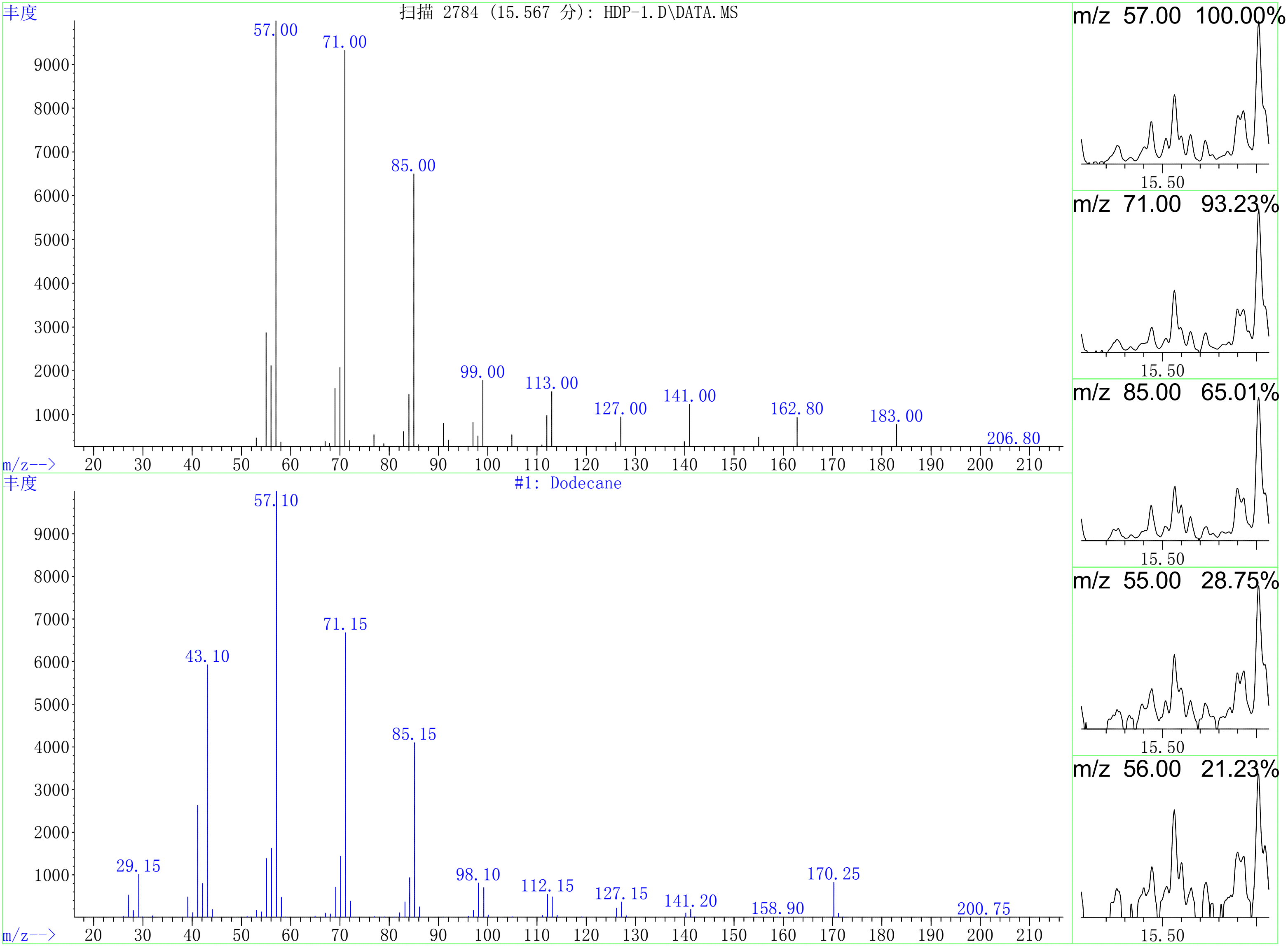

Data File: D:\GYM\DATA\2025\20251105\HDP-1.D
样品：HDP-1

峰编号： 23 15.567 分钟处 面积： 1764322 面积 % 0.05

每个谱库中 3 个最匹配的记录。	Ref\#	CAS\#	匹配度
C:\database\DEMO.L			
1 Dodecane	1	000112-40-3	78

未知谱图基于顶点

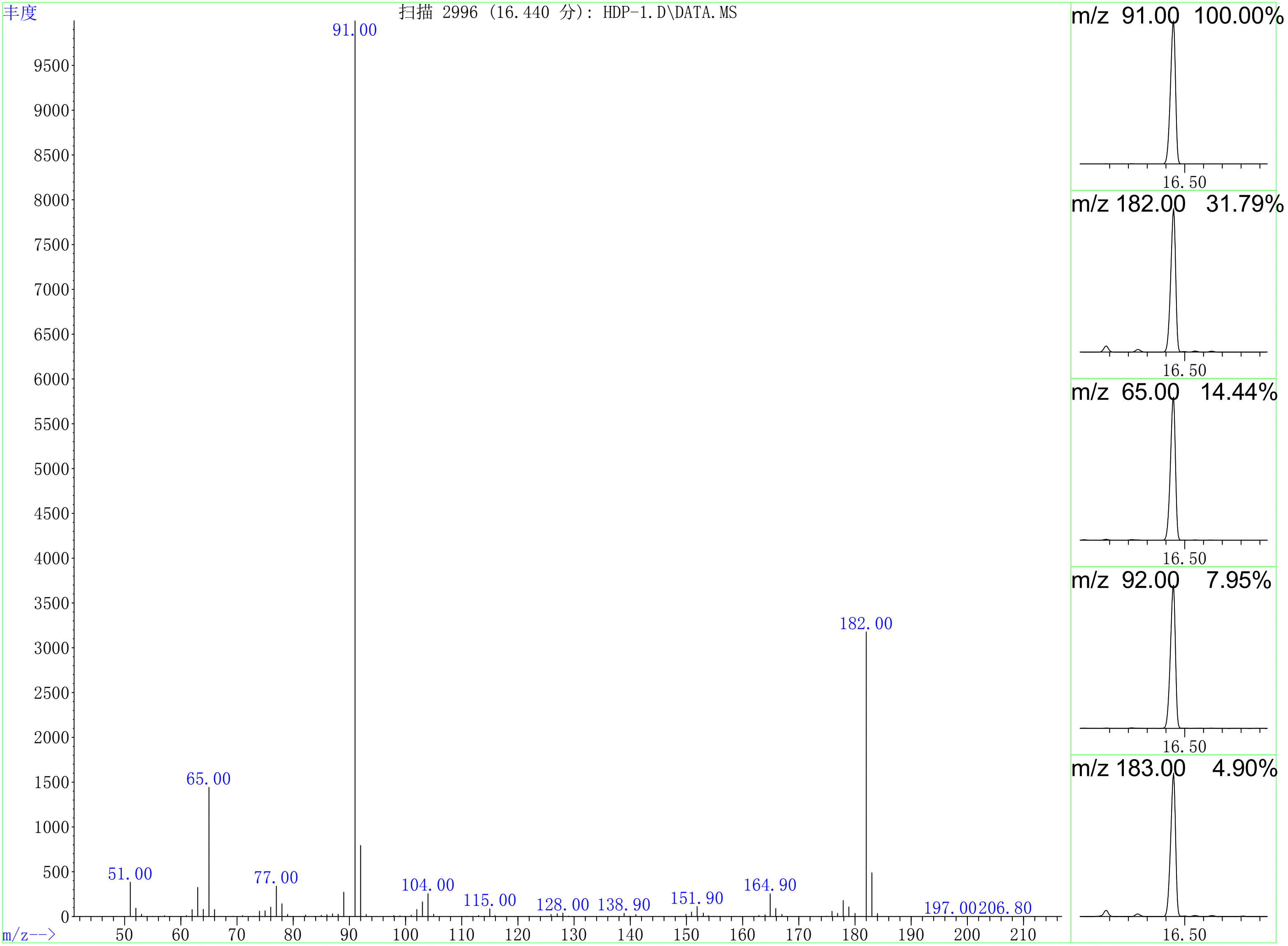

Data File: D:\GYM\DATA\2025\20251105\HDP-1.D
样品：HDP-1

峰编号： 24 16.440 分钟处 面积：100706087 面积 % 2.93

每个谱库中 3 个最匹配的记录。 Ref\# CAS\# 匹配度

C:\database\DEMO.L 未检索到匹配。

未知谱图基于顶点

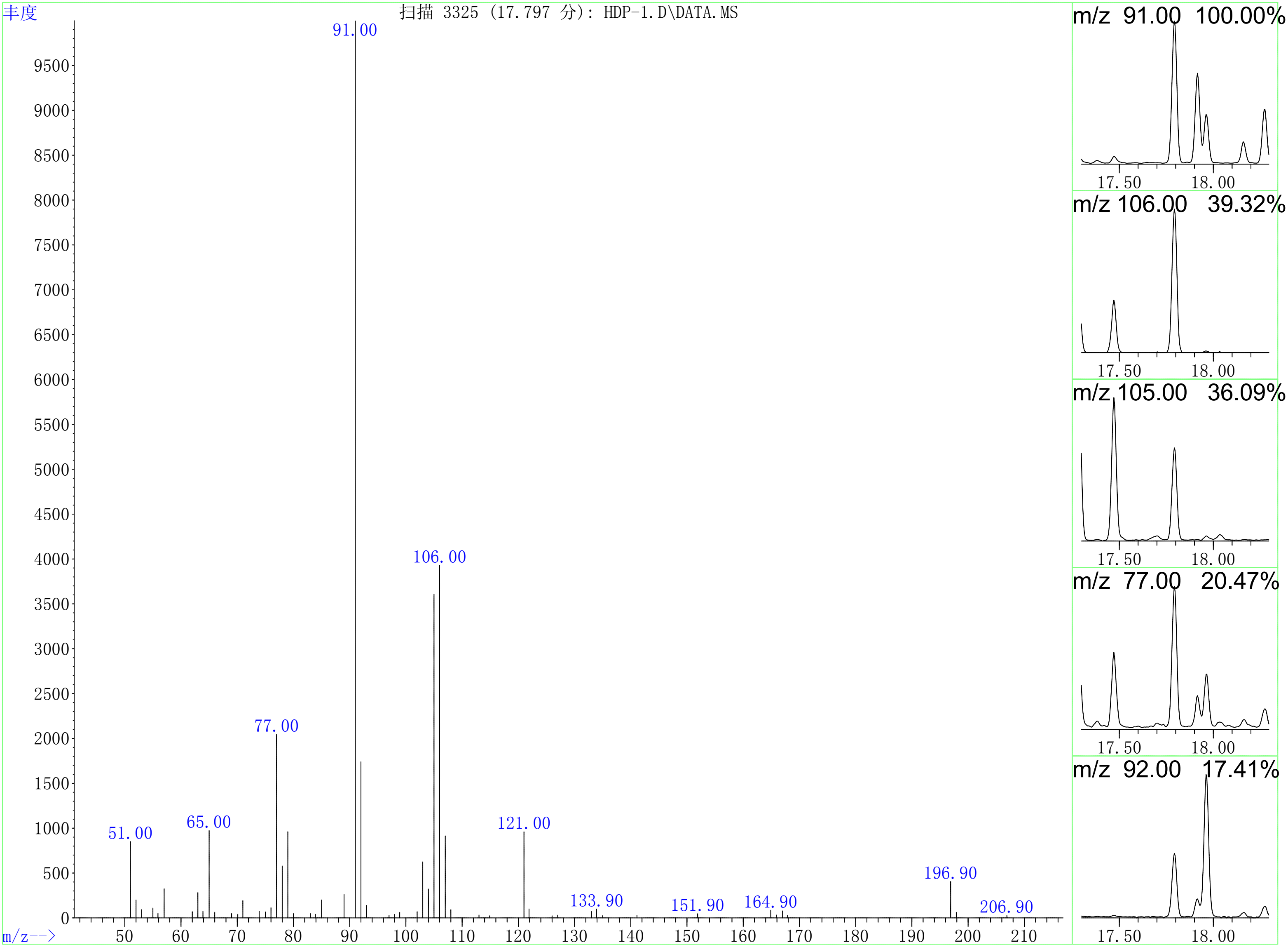

Data File: D:\GYM\DATA\2025\20251105\HDP-1.D
样品：HDP-1

峰编号： 25　　17.797 分钟处　面积： 16824473　面积 % 0.49

每个谱库中 3 个最匹配的记录。　　Ref\#　CAS\#　匹配度

C:\database\DEMO.L　未检索到匹配。

未知谱图基于顶点

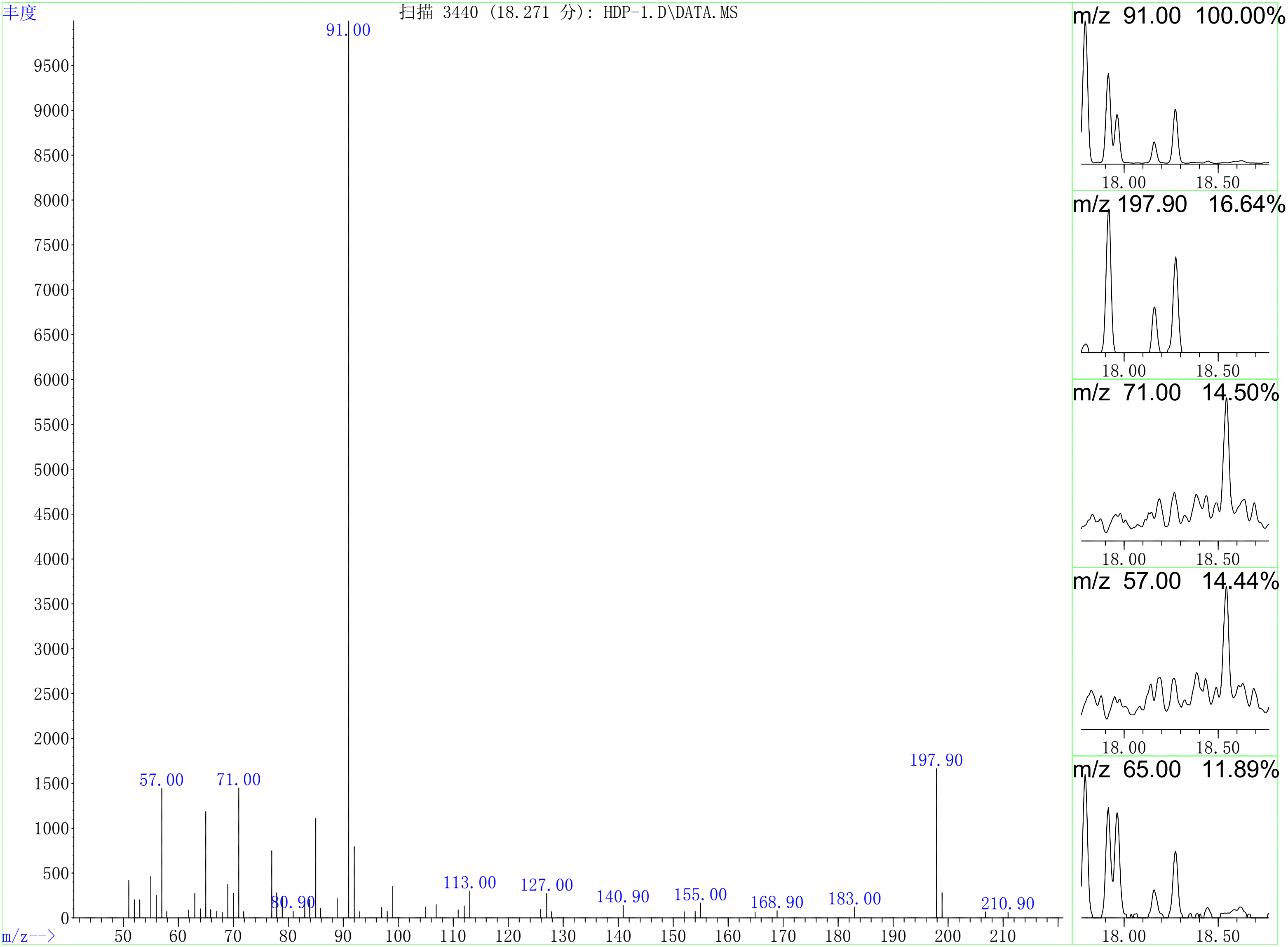

Data File: D:\GYM\DATA\2025\20251105\HDP-1.D
样品：HDP-1

峰编号： 26 18.271 分钟处 面积： 7396945 面积 % 0.22

每个谱库中 3 个最匹配的记录。 Ref\# CAS\# 匹配度

C:\database\DEMO.L 未检索到匹配。

未知谱图基于顶点

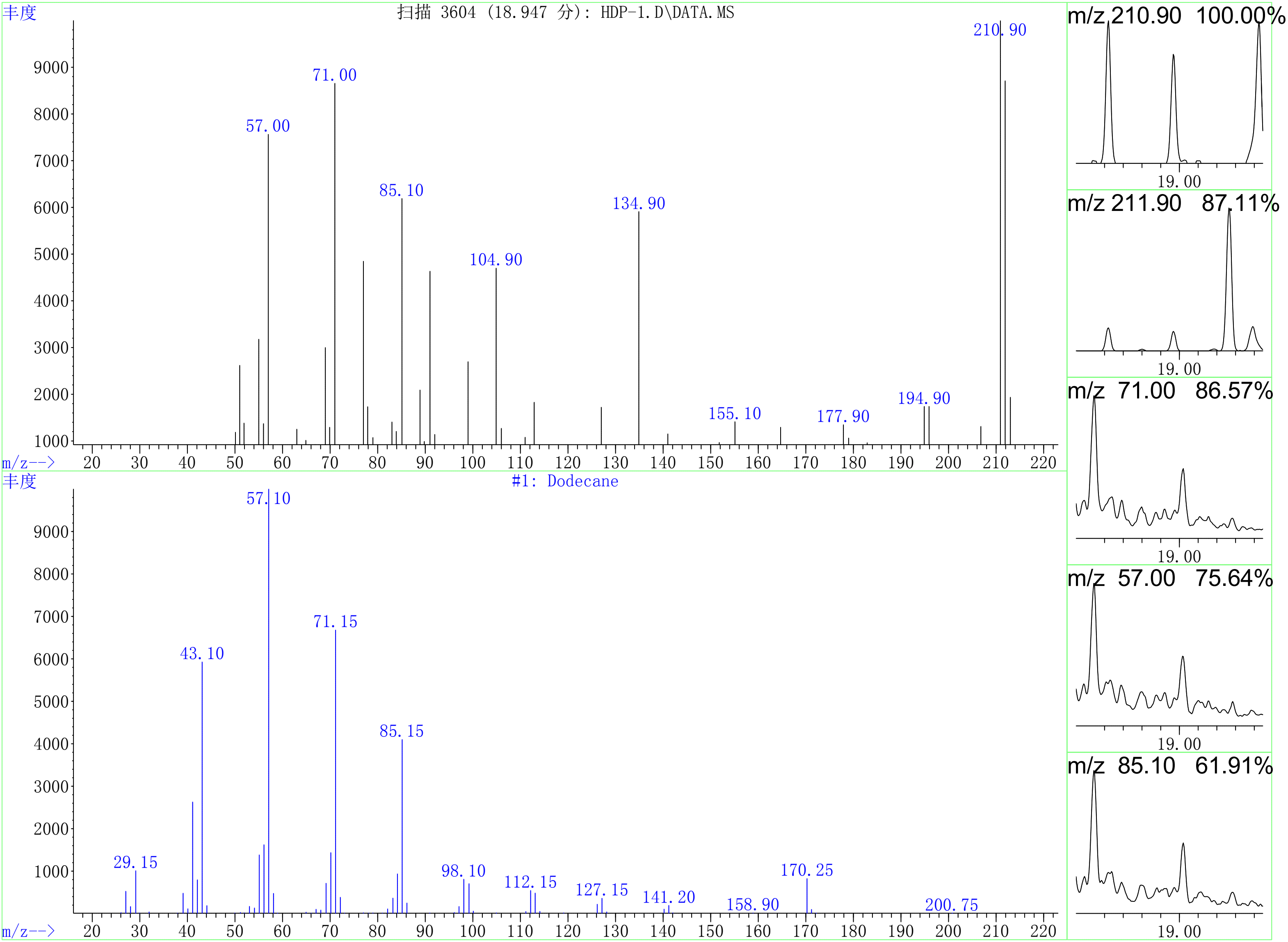

Data File: D:\GYM\DATA\2025\20251105\HDP-1.D
样品：HDP-1

峰编号： 27　　18.947 分钟处　面积：　5074941　面积 % 0.15

每个谱库中 3 个最匹配的记录。	Ref\#	CAS\#	匹配度
C:\database\DEMO.L			
1 Dodecane	1	000112-40-3	4

未知谱图基于顶点

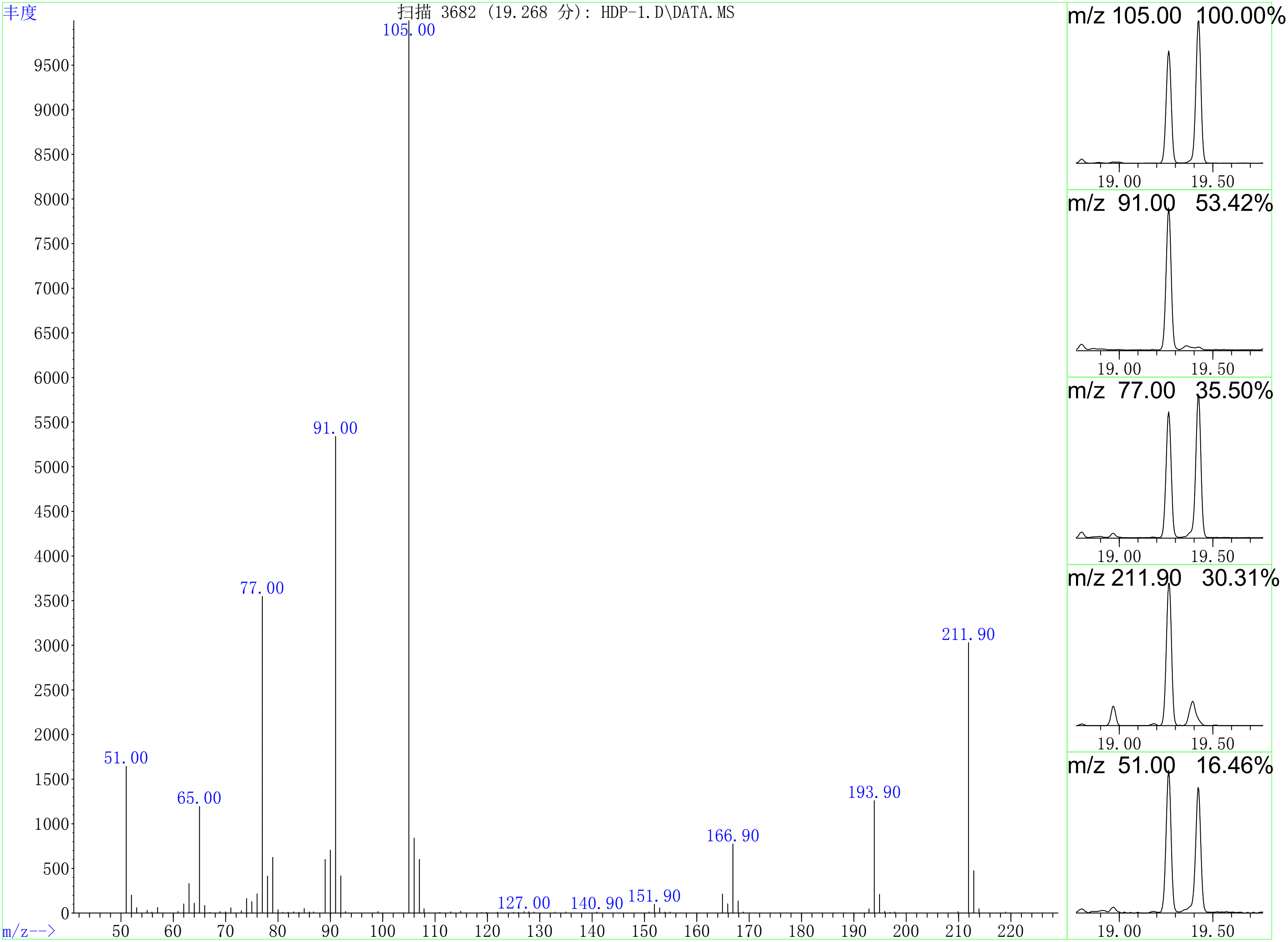

Data File: D:\GYM\DATA\2025\20251105\HDP-1.D
样品：HDP-1

峰编号： 28 19.268 分钟处 面积： 29033888 面积 % 0.84

每个谱库中 3 个最匹配的记录。 Ref\# CAS\# 匹配度

C:\database\DEMO.L 未检索到匹配。

未知谱图基于顶点

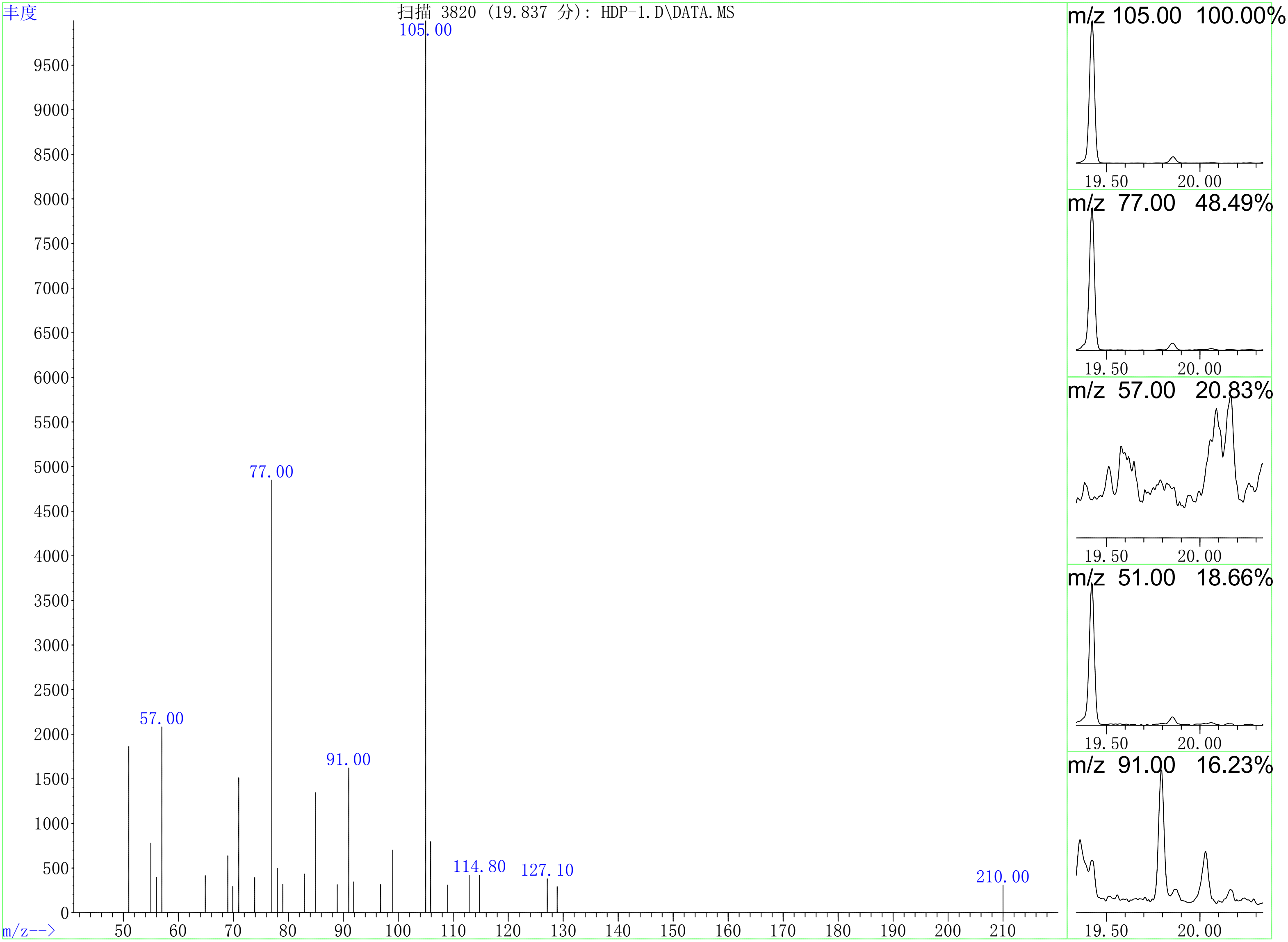

Data File: D:\GYM\DATA\2025\20251105\HDP-1.D
样品：HDP-1

峰编号： 29 19.837 分钟处 面积： 4319640 面积 % 0.13

每个谱库中 3 个最匹配的记录。 Ref\# CAS\# 匹配度

C:\database\DEMO.L 未检索到匹配。

未知谱图基于顶点

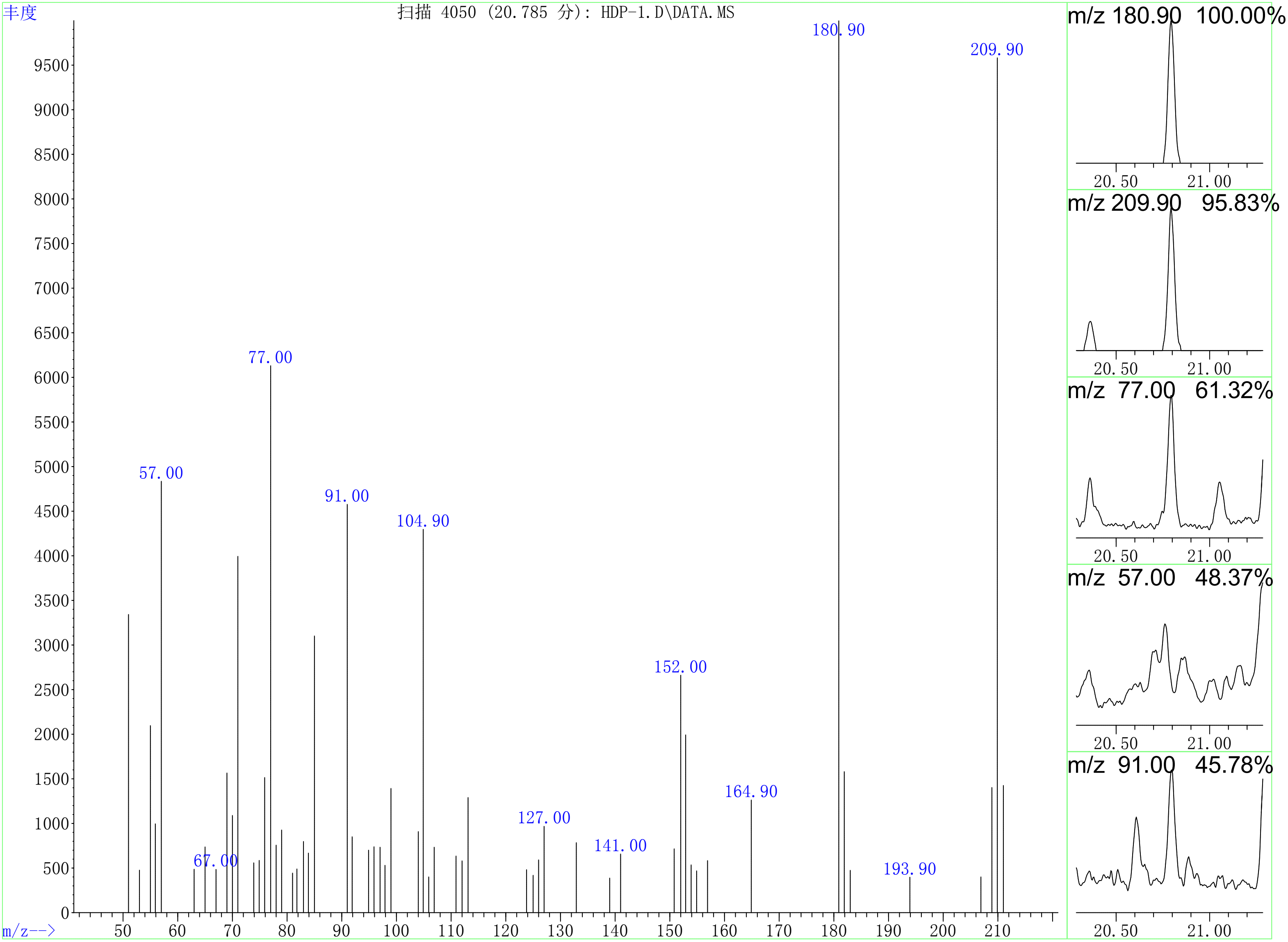

Data File: D:\GYM\DATA\2025\20251105\HDP-1.D
样品：HDP-1

峰编号： 30   20.785 分钟处 面积： 3920269 面积 % 0.11

每个谱库中 3 个最匹配的记录。   Ref\# CAS\# 匹配度

C:\database\DEMO.L 未检索到匹配。

谱库检索报告 - 化学工作站积分器

未知谱图基于顶点

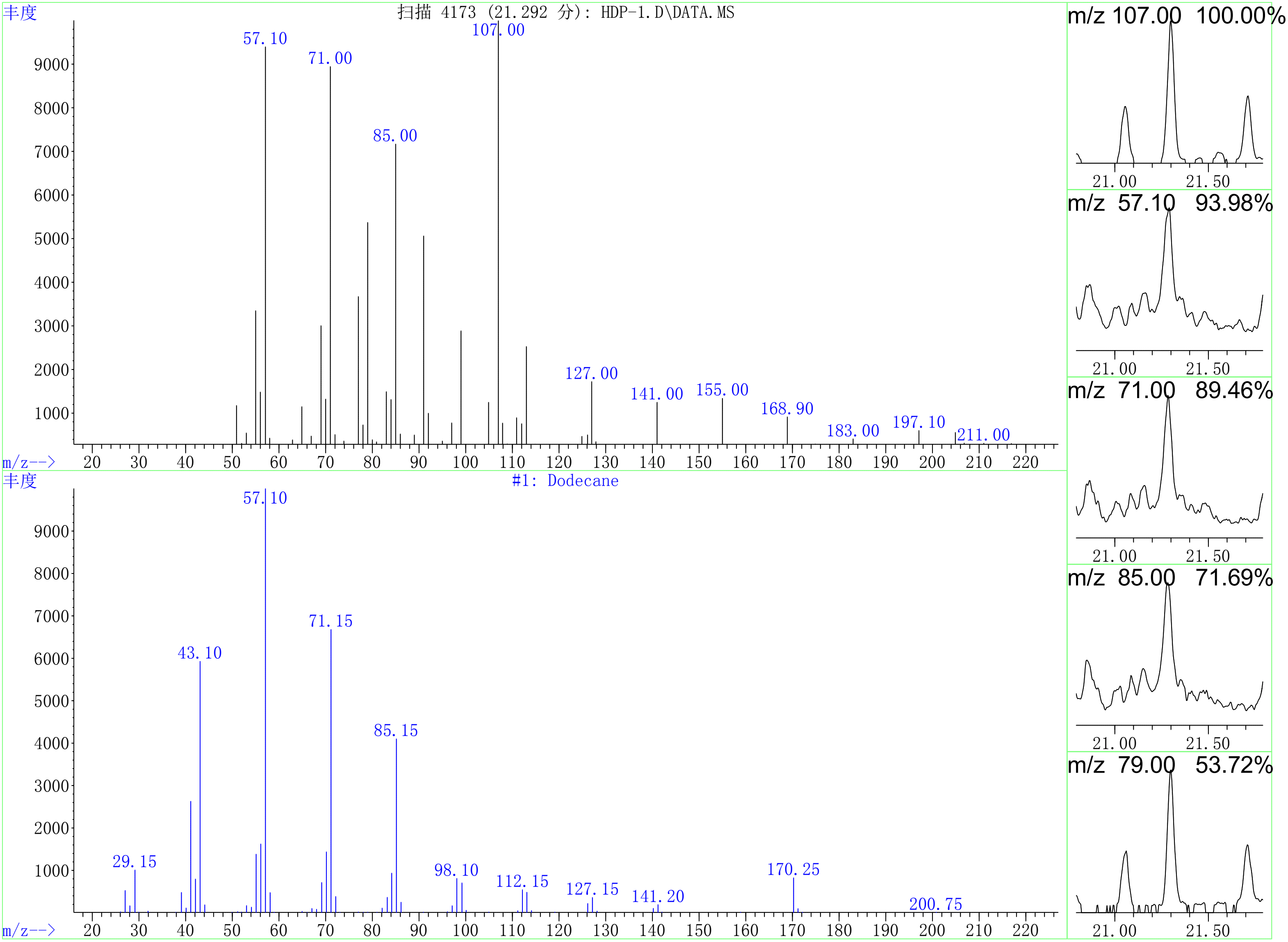

Data File: D:\GYM\DATA\2025\20251105\HDP-1.D
样品：HDP-1

峰编号： 31 21.292 分钟处 面积： 2470352 面积 % 0.07

每个谱库中 3 个最匹配的记录。 Ref\# CAS\# 匹配度

C:\database\DEMO.L

1 Dodecane	1	000112-40-3	22

未知谱图基于顶点

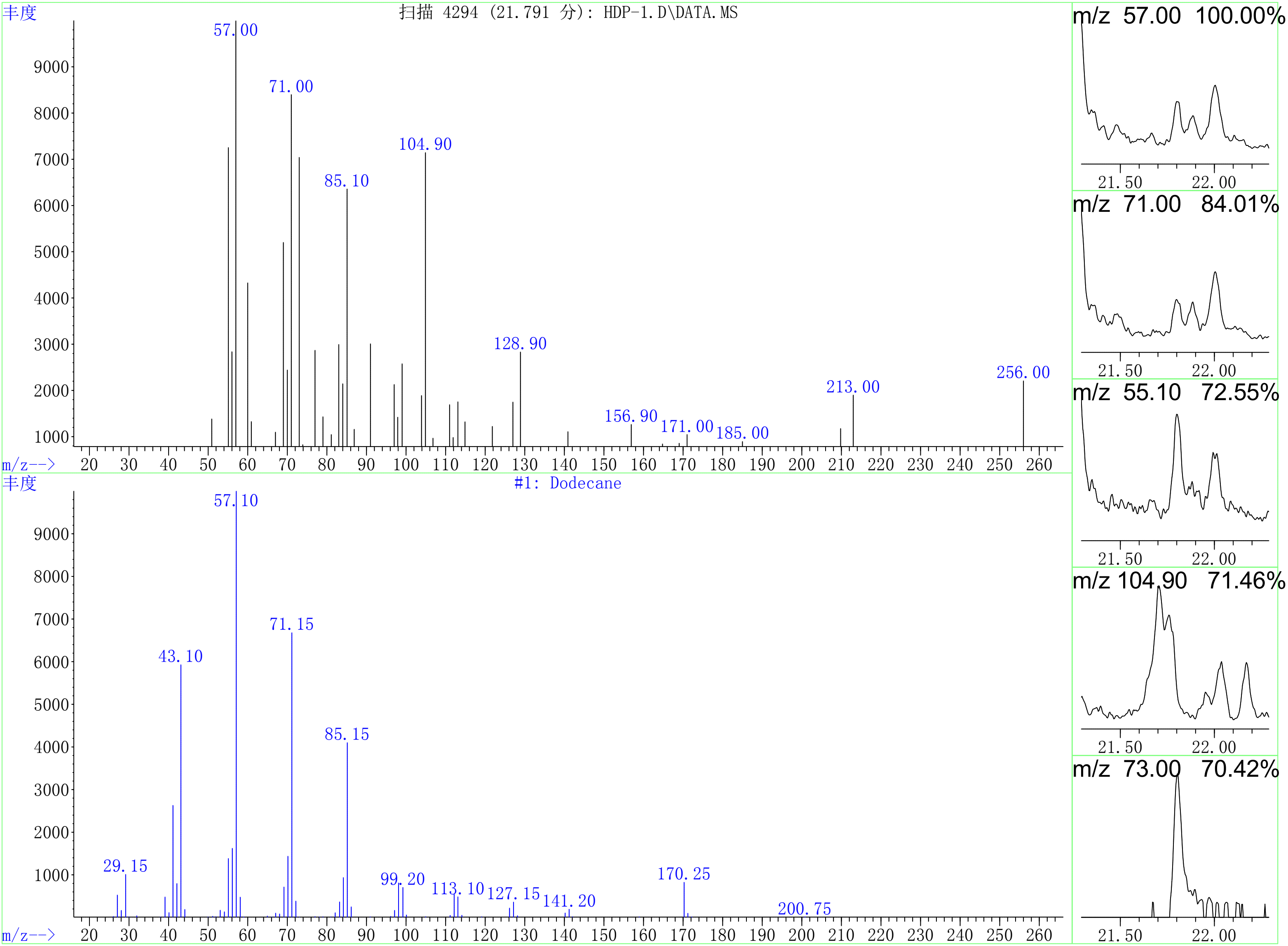

Data File: D:\GYM\DATA\2025\20251105\HDP-1.D
样品：HDP-1

峰编号： 32 21.791 分钟处 面积： 2976259 面积 % 0.09

每个谱库中 3 个最匹配的记录。	Ref\#	CAS\#	匹配度
C:\database\DEMO.L			
1 Dodecane	1	000112-40-3	25
