# Supplementary material for: High-throughput chiral copper foils by curved-surface confinement recrystallization
Source: Nat Commun. 2026 Feb 20;17:2796. doi: 10.1038/s41467-026-69862-7 (PMC13022495; doi:10.1038/s41467-026-69862-7)
Supplement: Supplementary file 3 — Supplementary Dataset 1 [file 41467_2026_69862_MOESM3_ESM.zip › Supplementary Data 1/Supplementary Fig.14-16/GC-MS Starting materials.pdf]

谱库检索报告

数据路径 : D:\GYM\DATA\2025\20251105\
数据文件 : HDP-0.D
采集 : 05 Nov 2025 16:11
操作者 : zky-HP\zky
样品 : HDP-0
其他 :
ALS 样品瓶: 2 样品乘积因子: 1

检索库: C:\database\DEMO.L 最小匹配度: 0

未知谱图: 顶点
积分事件: 化学工作站积分器 - events14.e

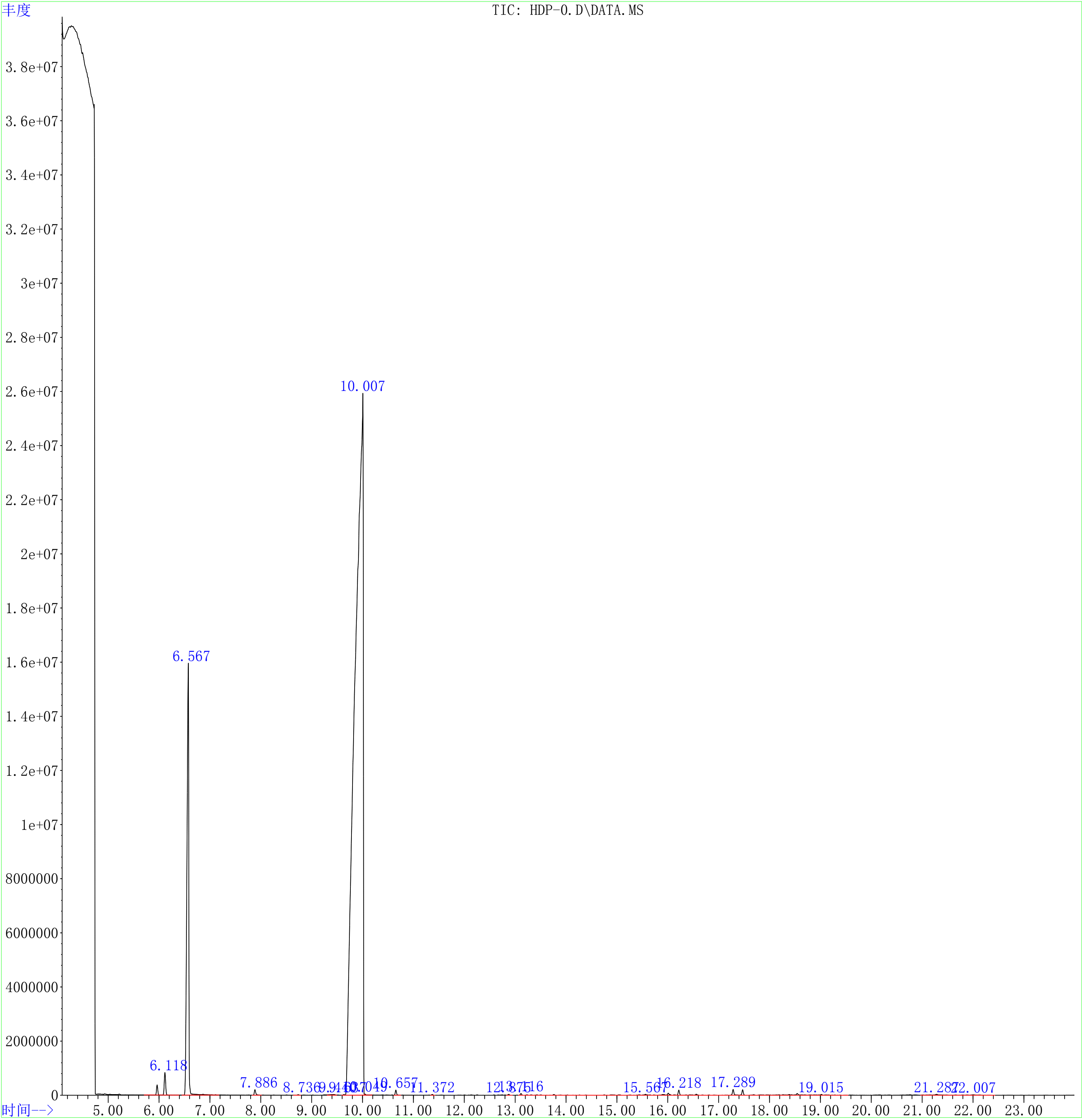

未知谱图基于顶点

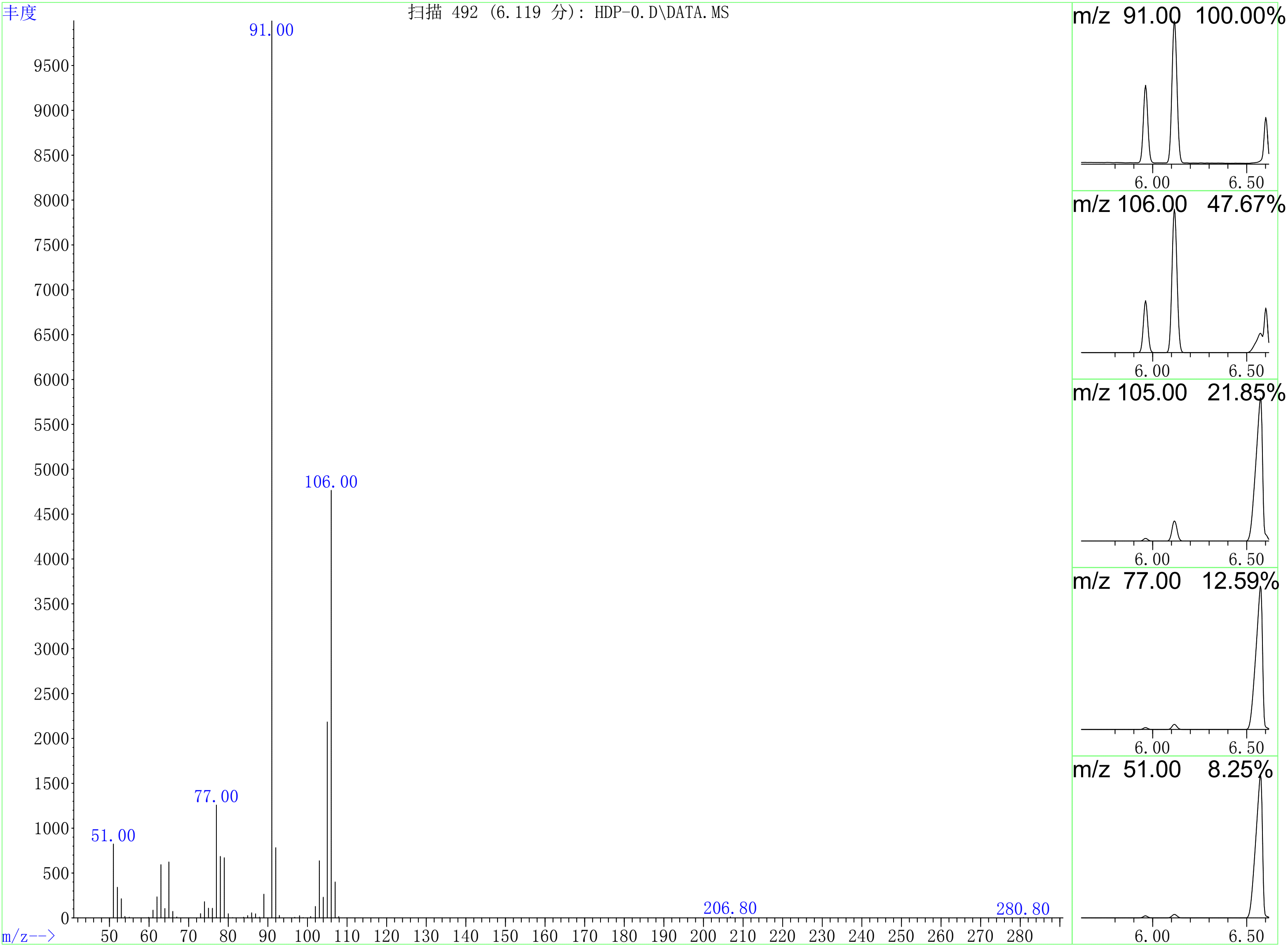

Data File: D:\GYM\DATA\2025\20251105\HDP-0.D
样品：HDP-0

峰编号： 1 6.119 分钟处 面积： 21903153 面积 % 0.65

每个谱库中 3 个最匹配的记录。 Ref\# CAS\# 匹配度

C:\database\DEMO.L 未检索到匹配。

未知谱图基于顶点

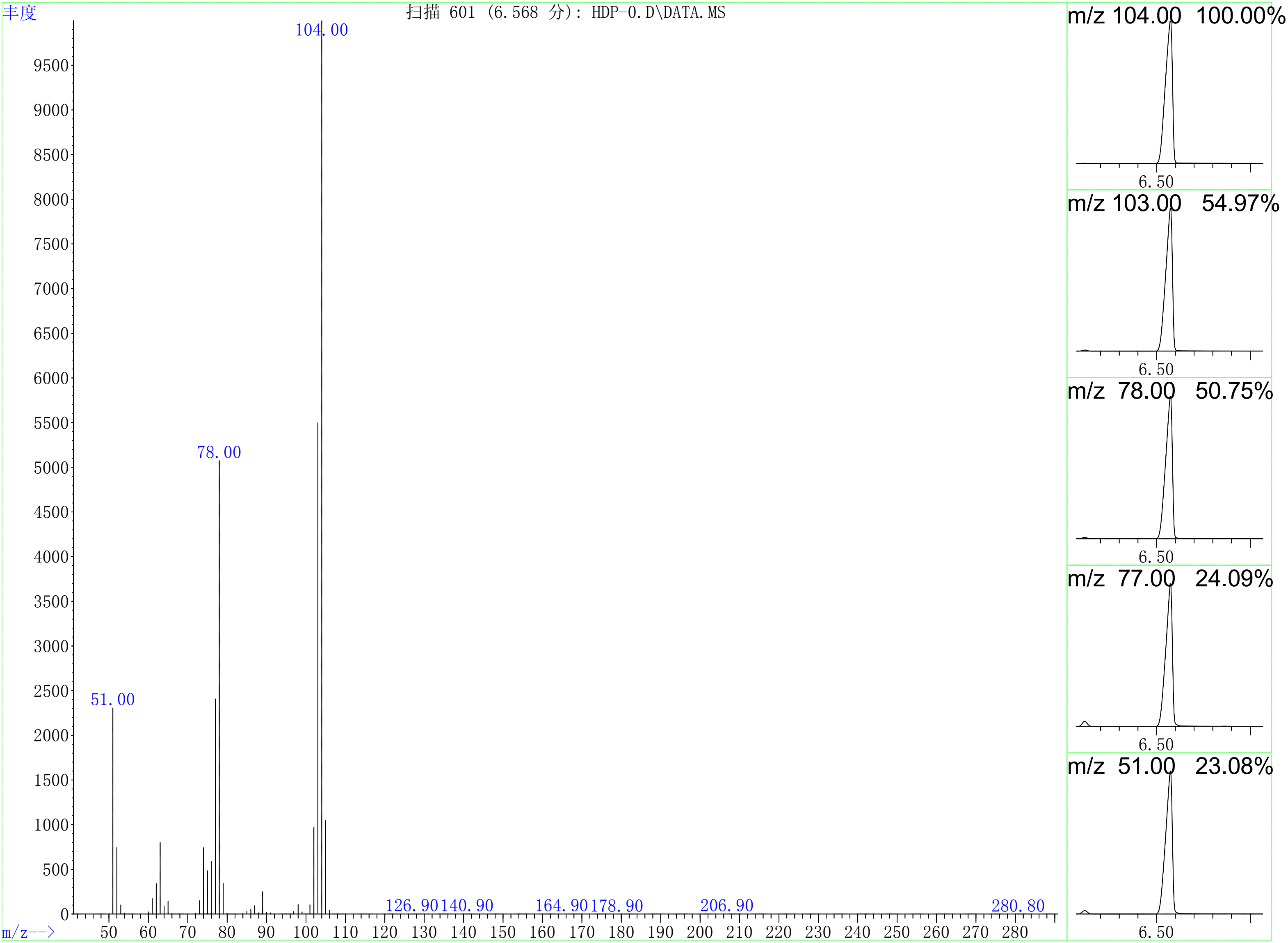

Data File: D:\GYM\DATA\2025\20251105\HDP-0.D
样品：HDP-0

峰编号： 2　　6.568 分钟处　面积：404289771　面积 % 11.93

每个谱库中 3 个最匹配的记录。　　Ref\#　CAS\#　匹配度

C:\database\DEMO.L　未检索到匹配。

未知谱图基于顶点

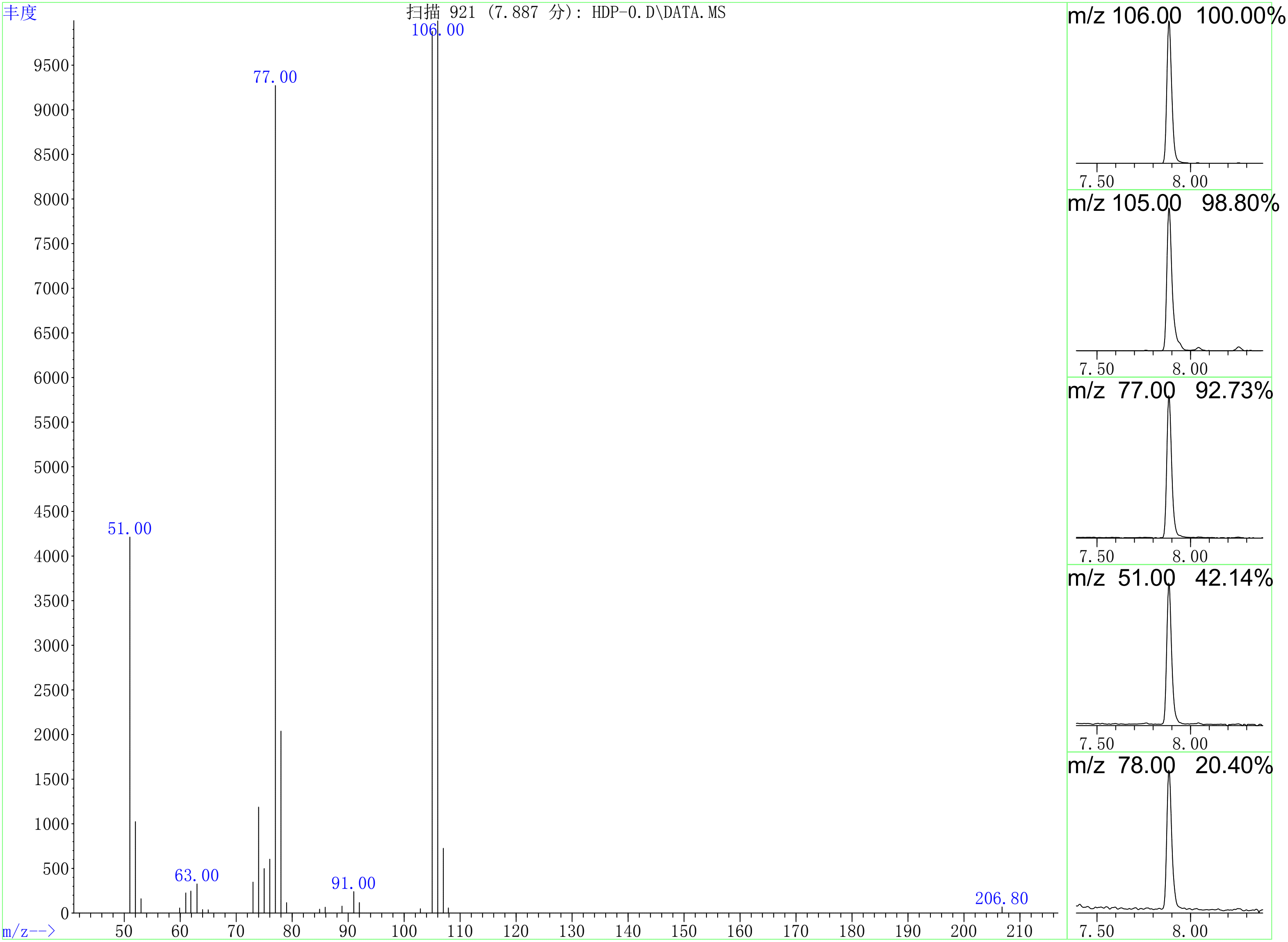

Data File: D:\GYM\DATA\2025\20251105\HDP-0.D
样品：HDP-0

峰编号： 3 7.887 分钟处 面积： 3913854 面积 % 0.12

每个谱库中 3 个最匹配的记录。	Ref\#	CAS\#	匹配度
C:\database\DEMO.L 未检索到匹配。			

未知谱图基于顶点

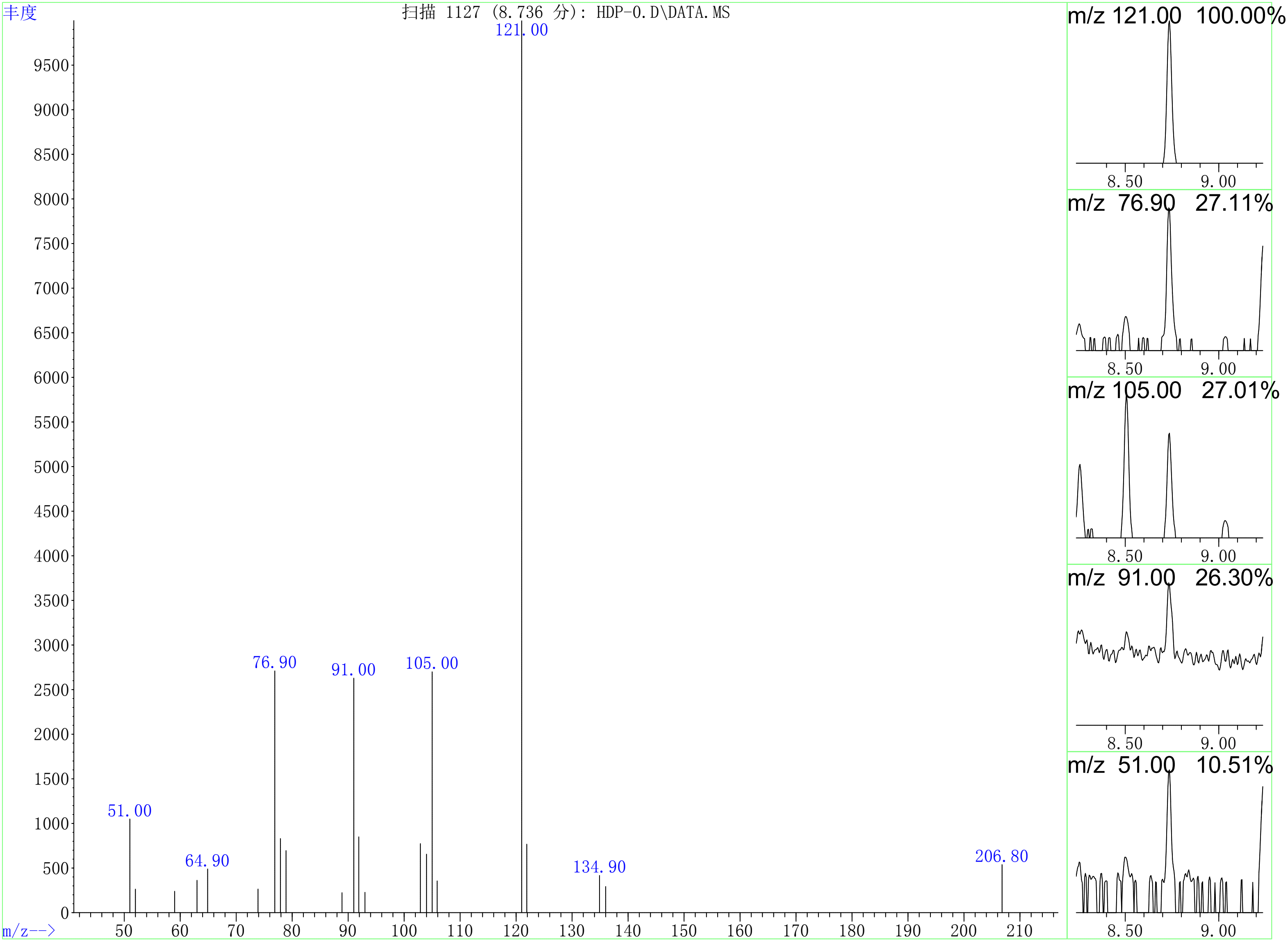

Data File: D:\GYM\DATA\2025\20251105\HDP-0.D
样品：HDP-0

峰编号： 4 8.736 分钟处 面积： 318413 面积 % 0.01

每个谱库中 3 个最匹配的记录。	Ref\#	CAS\#	匹配度
C:\database\DEMO.L 未检索到匹配。			

未知谱图基于顶点

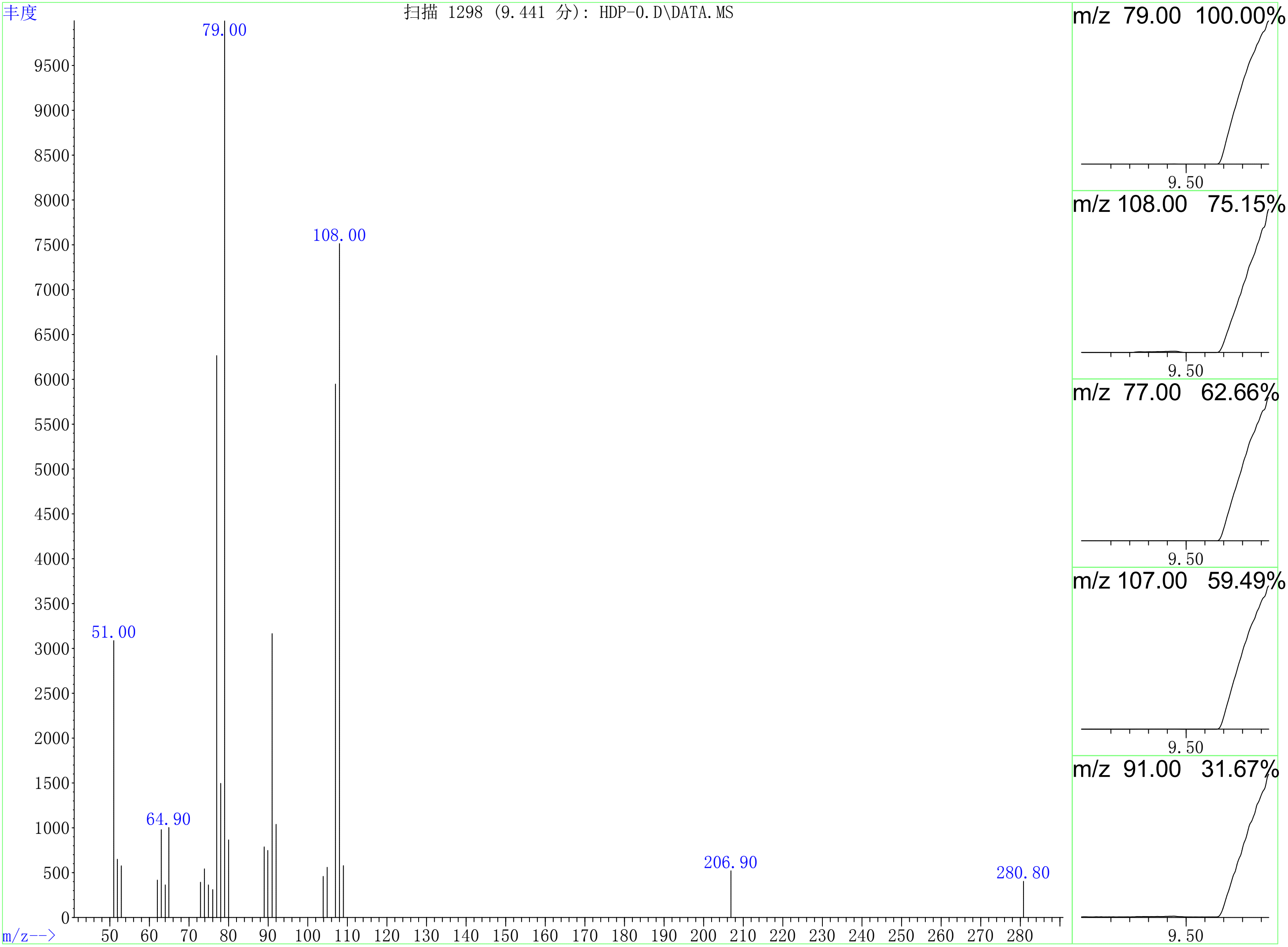

Data File: D:\GYM\DATA\2025\20251105\HDP-0.D
样品：HDP-0

峰编号： 5 9.441 分钟处 面积： 1614837 面积 % 0.05

每个谱库中 3 个最匹配的记录。 Ref\# CAS\# 匹配度

C:\database\DEMO.L 未检索到匹配。

未知谱图基于顶点

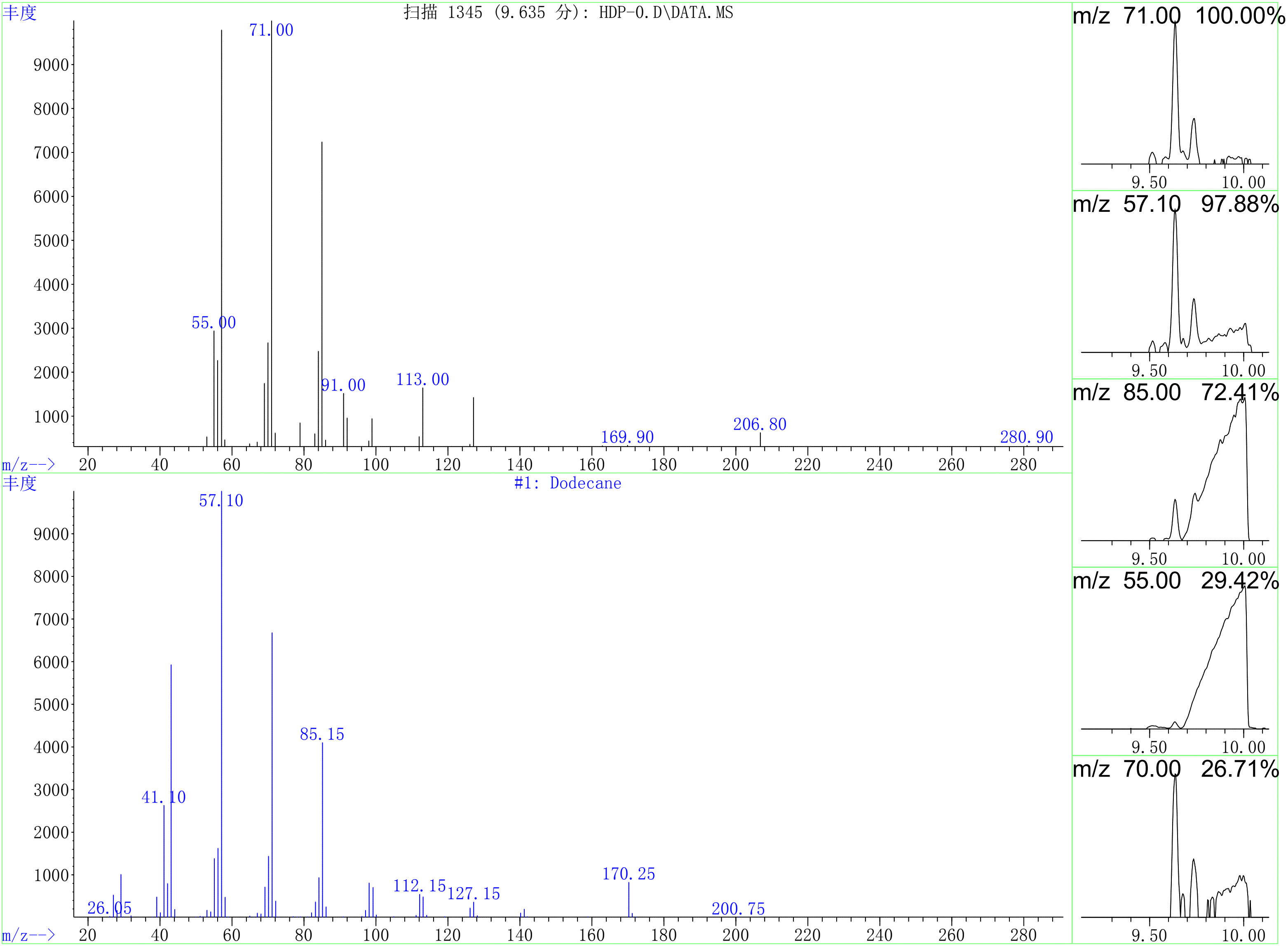

Data File: D:\GYM\DATA\2025\20251105\HDP-0.D
样品：HDP-0

峰编号： 6　　9.635 分钟处　面积：　373998　面积 % 0.01

每个谱库中 3 个最匹配的记录。	Ref\#	CAS\#	匹配度
C:\database\DEMO.L			
1 Dodecane	1	000112-40-3	50

未知谱图基于顶点

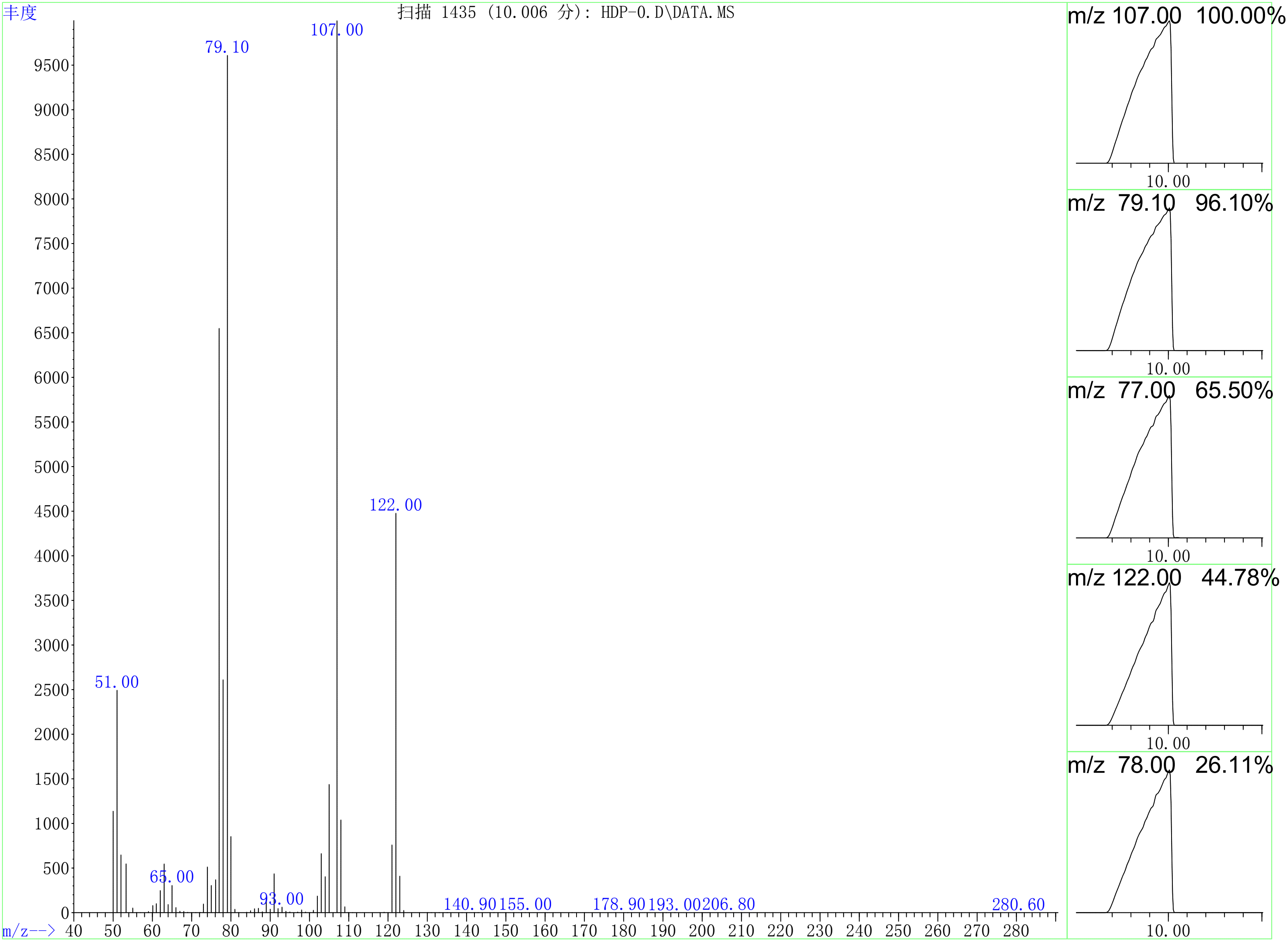

Data File: D:\GYM\DATA\2025\20251105\HDP-0.D
样品：HDP-0

峰编号： 7 10.006 分钟处 面积：2915099717 面积 % 86.04

每个谱库中 3 个最匹配的记录。 Ref\# CAS\# 匹配度

C:\database\DEMO.L 未检索到匹配。

未知谱图基于顶点

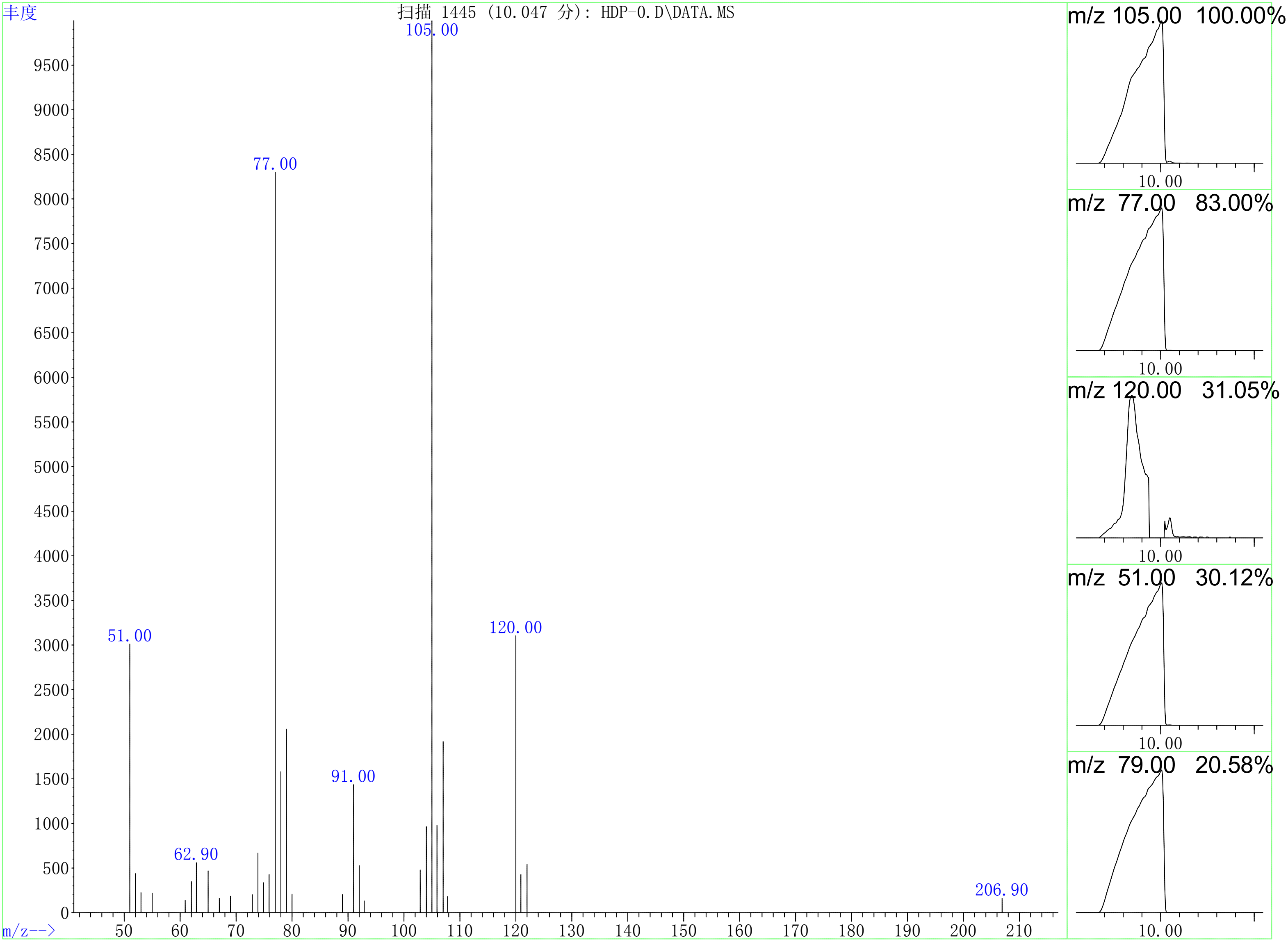

Data File: D:\GYM\DATA\2025\20251105\HDP-0.D
样品：HDP-0

峰编号： 8 10.047 分钟处 面积： 738658 面积 % 0.02

每个谱库中 3 个最匹配的记录。 Ref\# CAS\# 匹配度

C:\database\DEMO.L 未检索到匹配。

未知谱图基于顶点

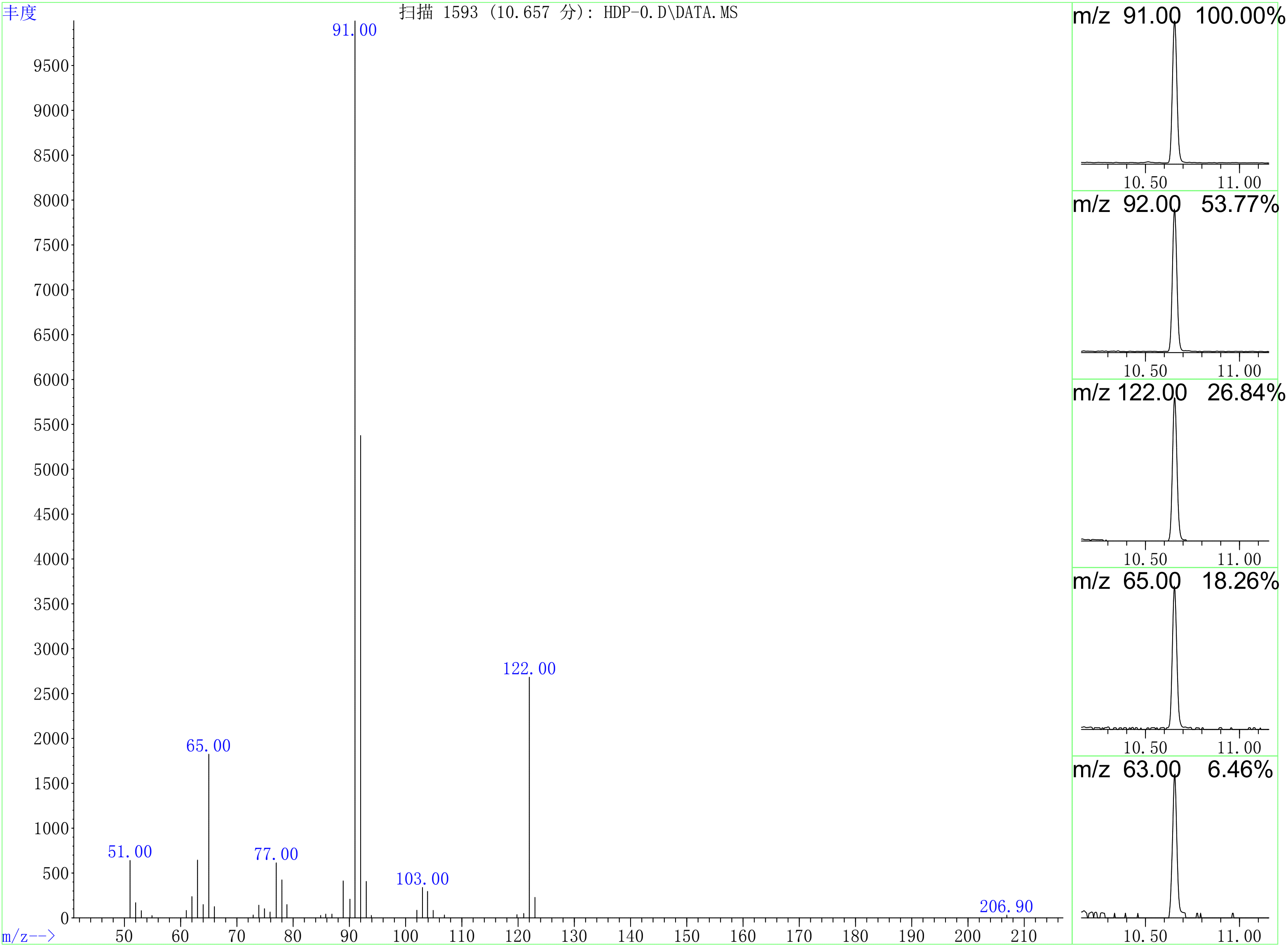

Data File: D:\GYM\DATA\2025\20251105\HDP-0.D
样品：HDP-0

峰编号： 9　　10.657 分钟处　面积：　3157911　面积 % 0.09

每个谱库中 3 个最匹配的记录。　　Ref\#　CAS\#　匹配度

C:\database\DEMO.L　未检索到匹配。

未知谱图基于顶点

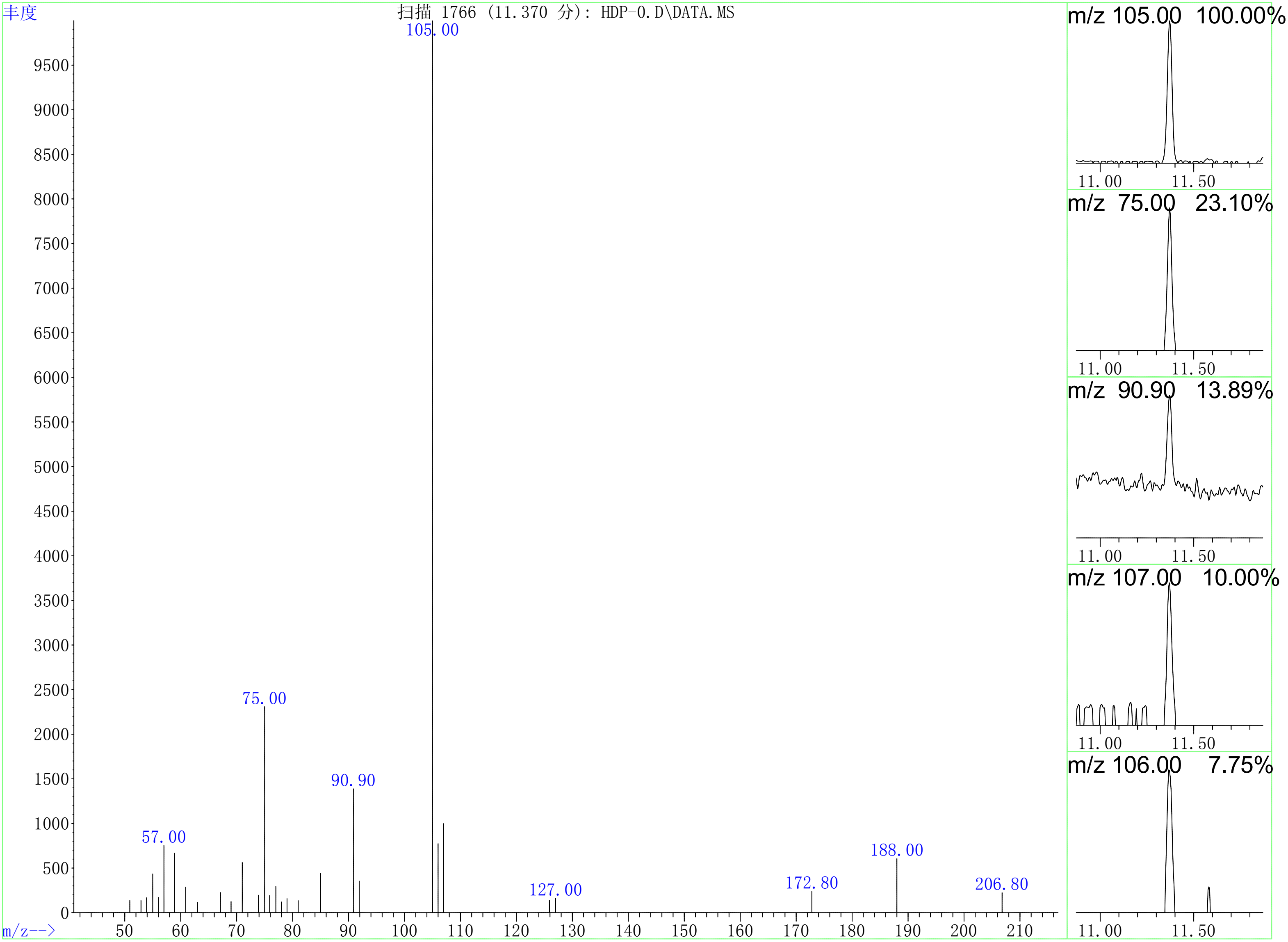

Data File: D:\GYM\DATA\2025\20251105\HDP-0.D
样品：HDP-0

峰编号： 10 11.370 分钟处 面积： 475421 面积 % 0.01

每个谱库中 3 个最匹配的记录。 Ref\# CAS\# 匹配度

C:\database\DEMO.L 未检索到匹配。

未知谱图基于顶点

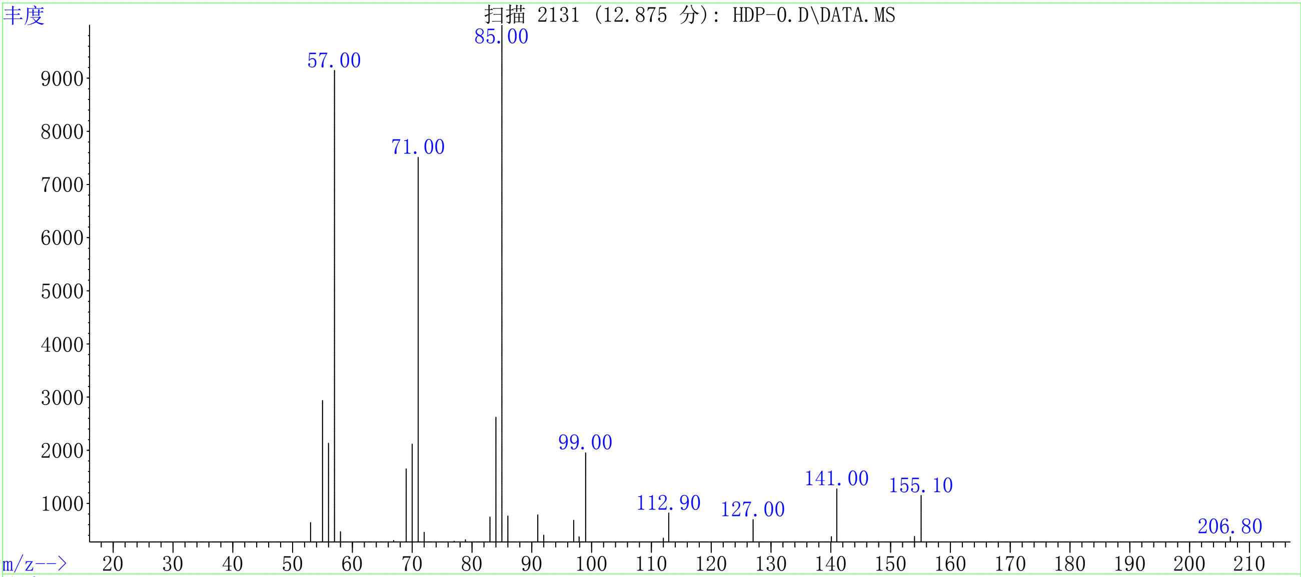

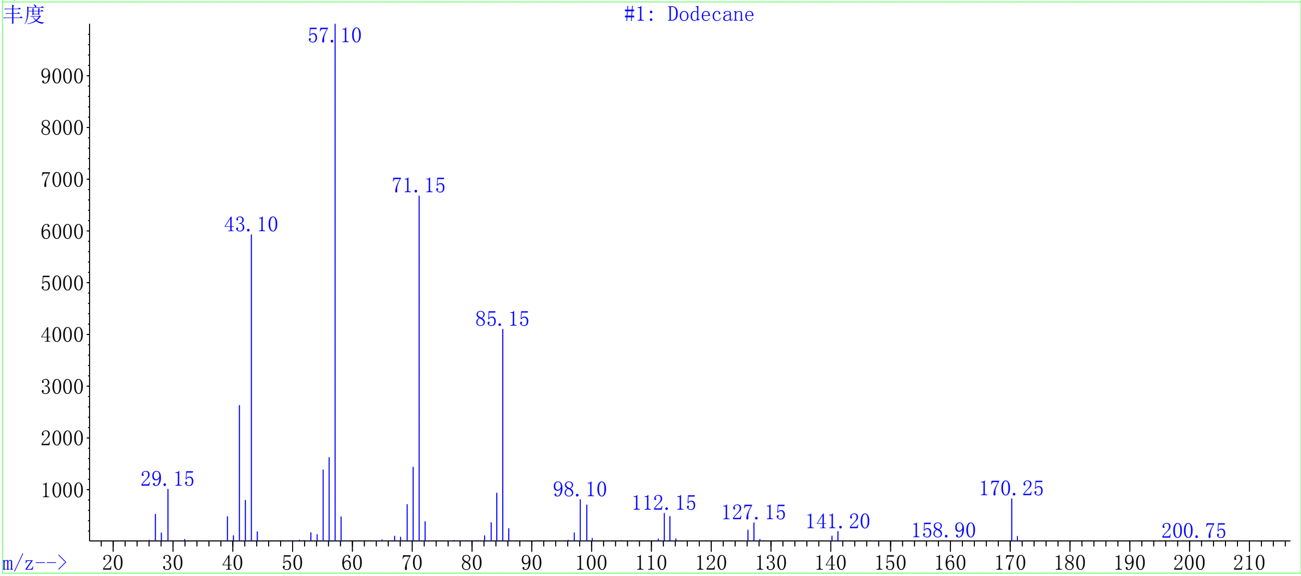

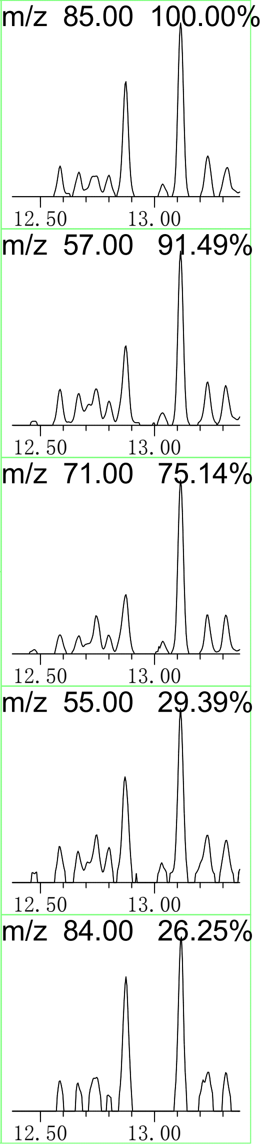

Data File: D:\GYM\DATA\2025\20251105\HDP-0.D
样品：HDP-0

峰编号： 11 12.875 分钟处 面积： 599277 面积 % 0.02

每个谱库中 3 个最匹配的记录。	Ref\#	CAS\#	匹配度
C:\database\DEMO.L			
1 Dodecane	1	000112-40-3	37

未知谱图基于顶点

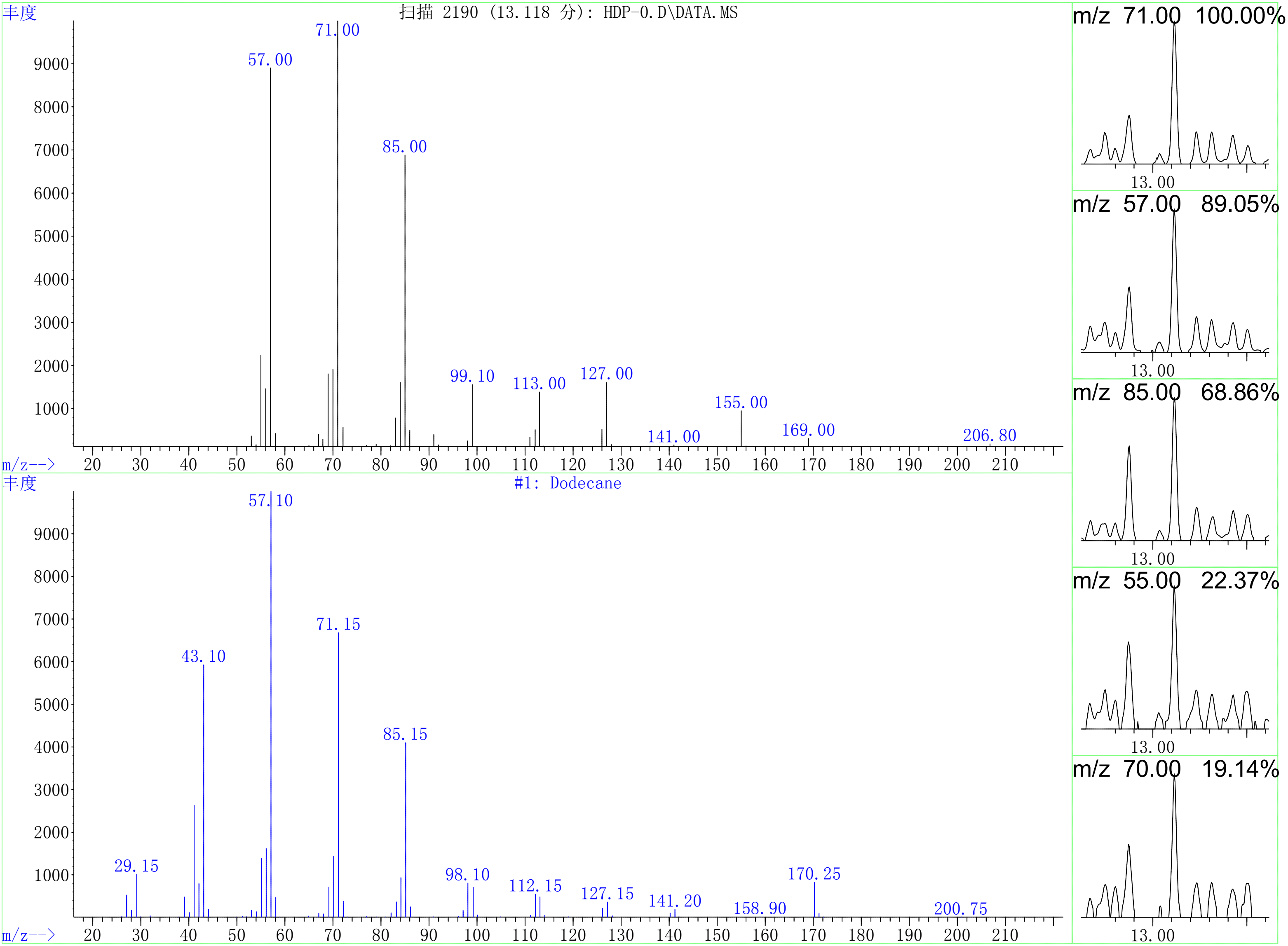

Data File: D:\GYM\DATA\2025\20251105\HDP-0.D
样品：HDP-0

峰编号： 12 13.118 分钟处 面积： 2031521 面积 % 0.06

每个谱库中 3 个最匹配的记录。 Ref\# CAS\# 匹配度

C:\database\DEMO.L

1 Dodecane	1	000112-40-3	42

未知谱图基于顶点

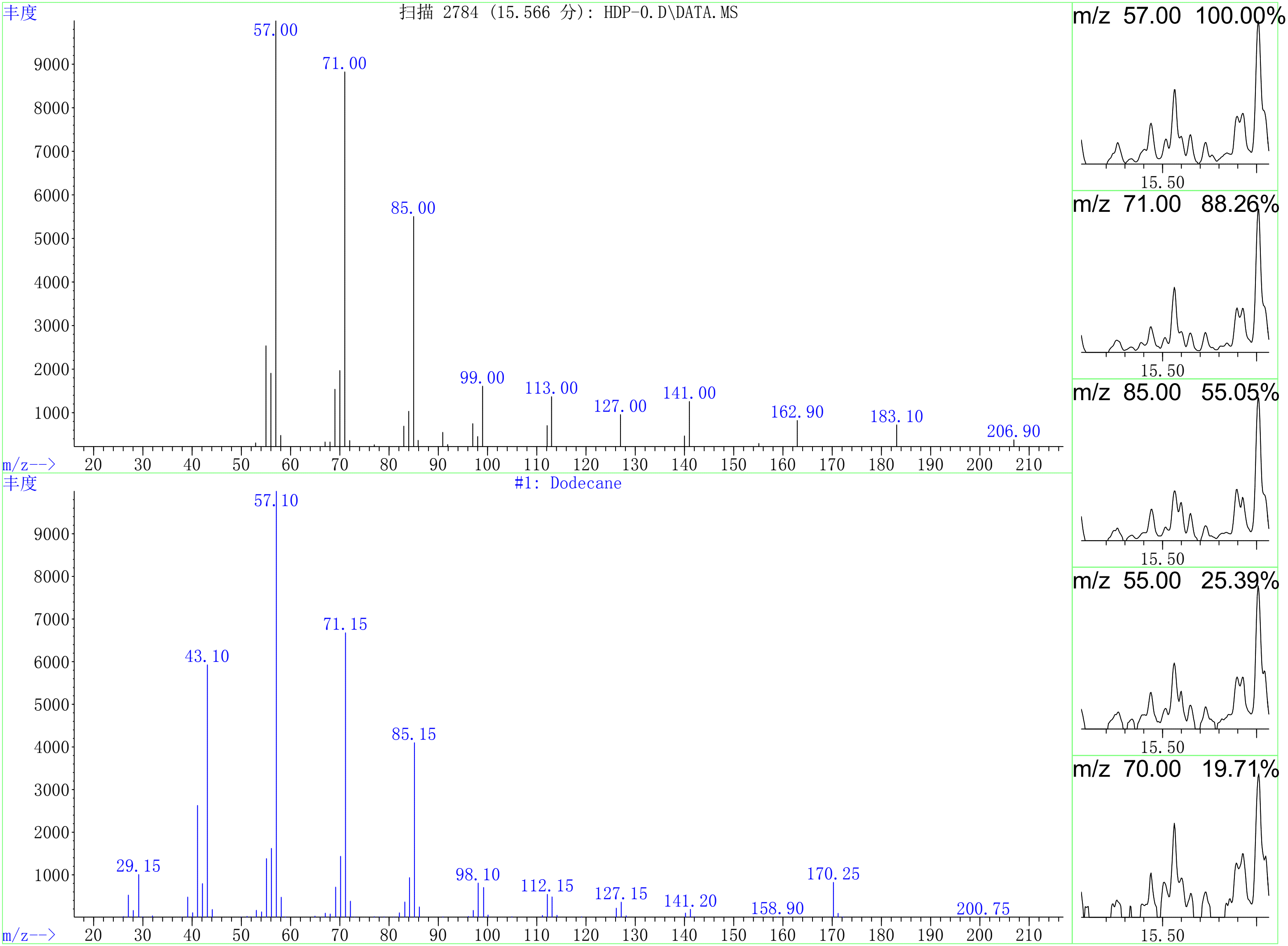

Data File: D:\GYM\DATA\2025\20251105\HDP-0.D
样品：HDP-0

峰编号： 13 15.566 分钟处 面积： 4088366 面积 % 0.12

每个谱库中 3 个最匹配的记录。	Ref\#	CAS\#	匹配度
C:\database\DEMO.L			
1 Dodecane	1	000112-40-3	64

未知谱图基于顶点

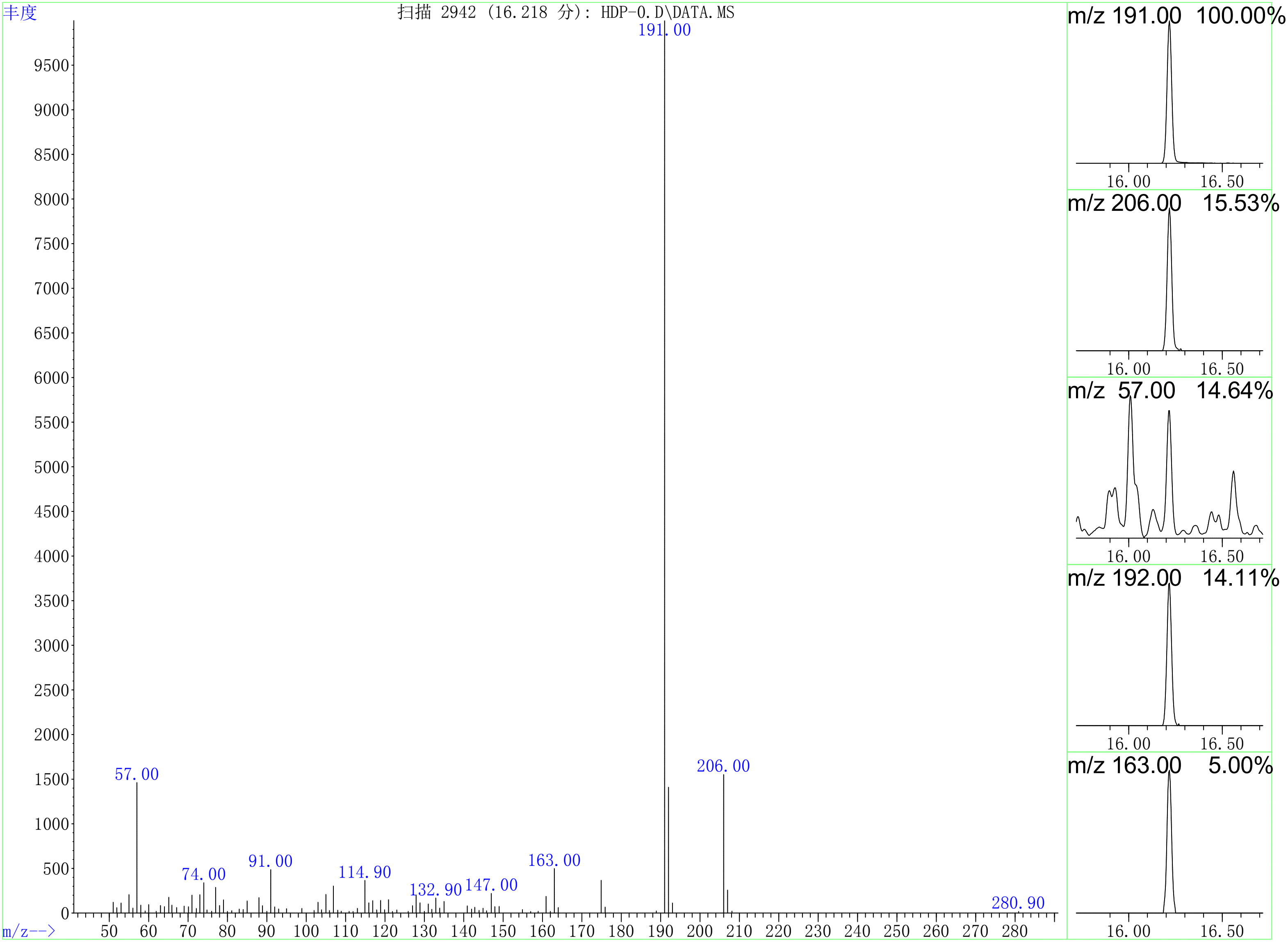

Data File: D:\GYM\DATA\2025\20251105\HDP-0.D
样品：HDP-0

峰编号： 14 16.218 分钟处 面积： 8681047 面积 % 0.26

每个谱库中 3 个最匹配的记录。 Ref\# CAS\# 匹配度

C:\database\DEMO.L 未检索到匹配。

未知谱图基于顶点

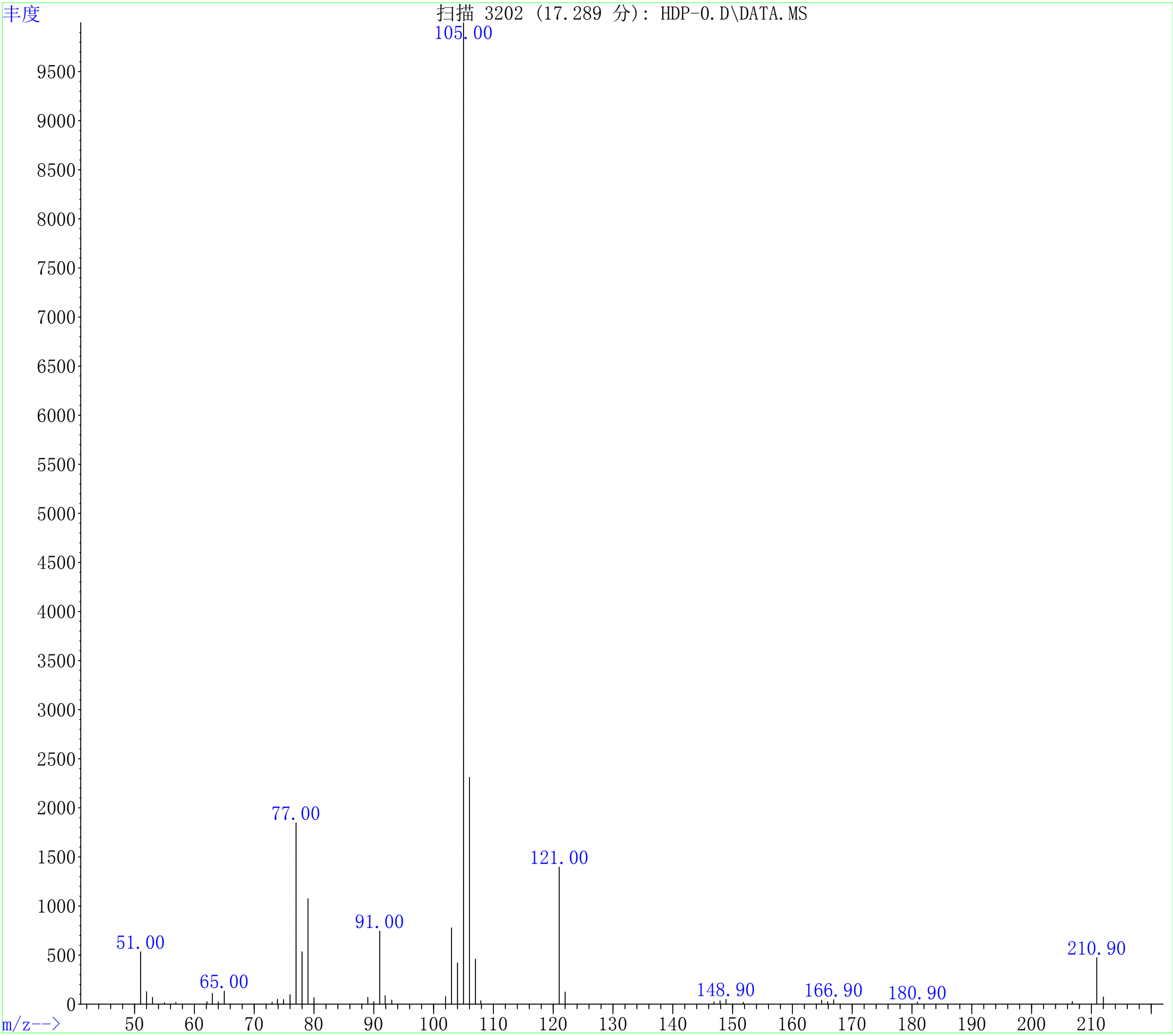

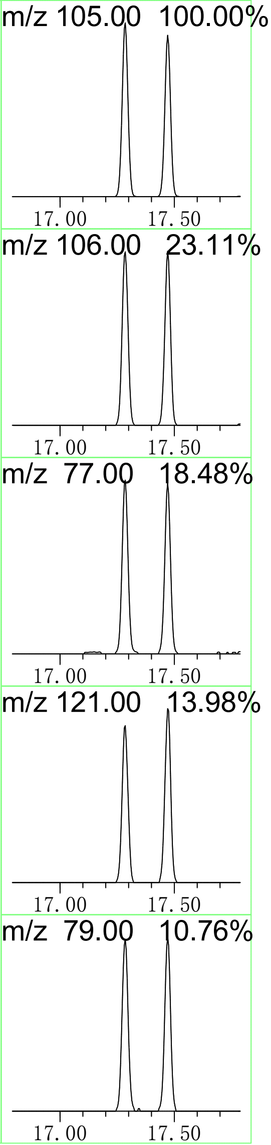

Data File: D:\GYM\DATA\2025\20251105\HDP-0.D
样品：HDP-0

峰编号： 15 17.289 分钟处 面积： 14981165 面积 % 0.44

每个谱库中 3 个最匹配的记录。 Ref\# CAS\# 匹配度

C:\database\DEMO.L 未检索到匹配。

未知谱图基于顶点

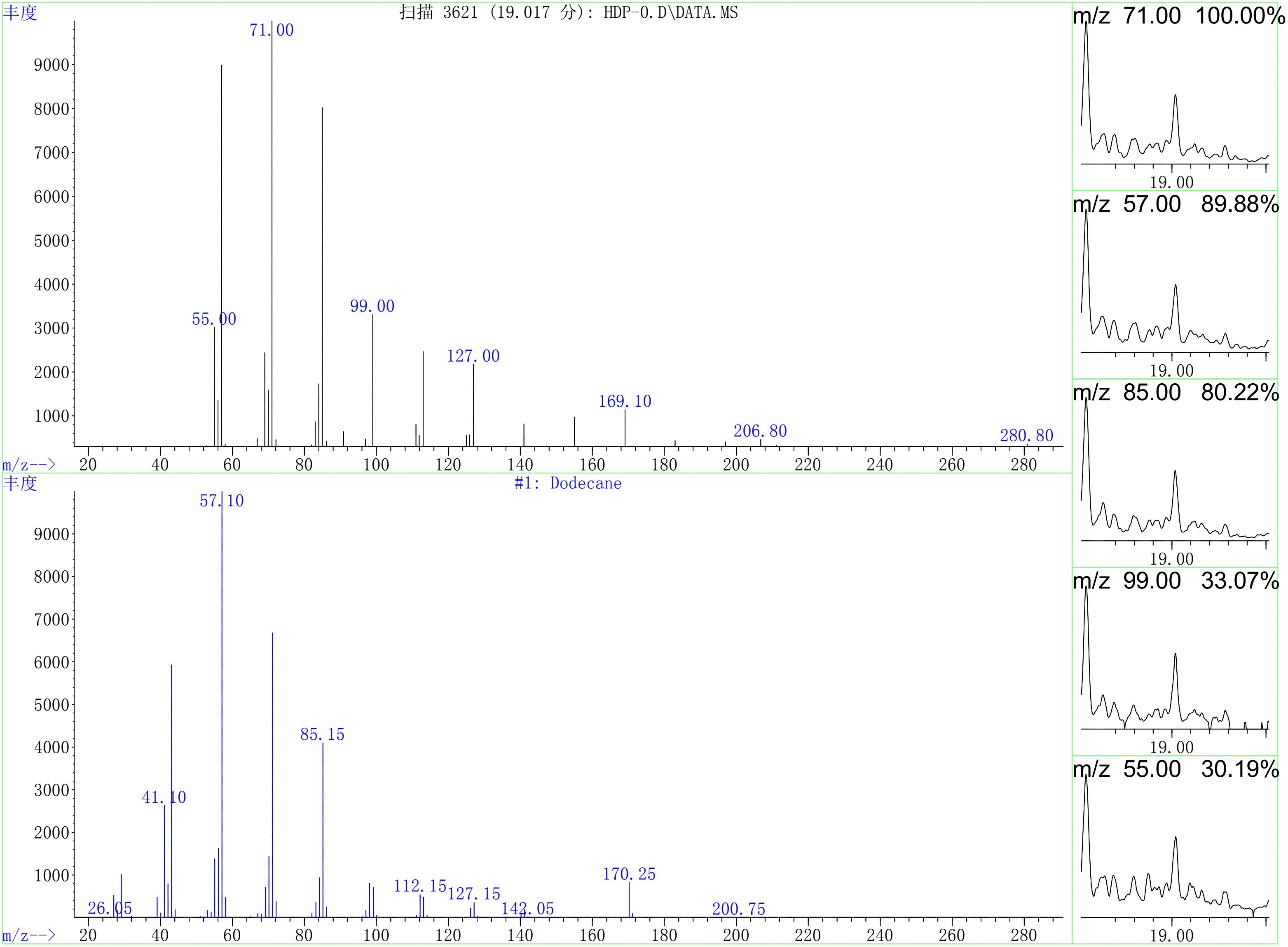

Data File: D:\GYM\DATA\2025\20251105\HDP-0.D
样品：HDP-0

峰编号： 16 19.017 分钟处 面积： 1878936 面积 % 0.06

每个谱库中 3 个最匹配的记录。	Ref\#	CAS\#	匹配度
C:\database\DEMO.L			
1 Dodecane	1	000112-40-3	9

未知谱图基于顶点

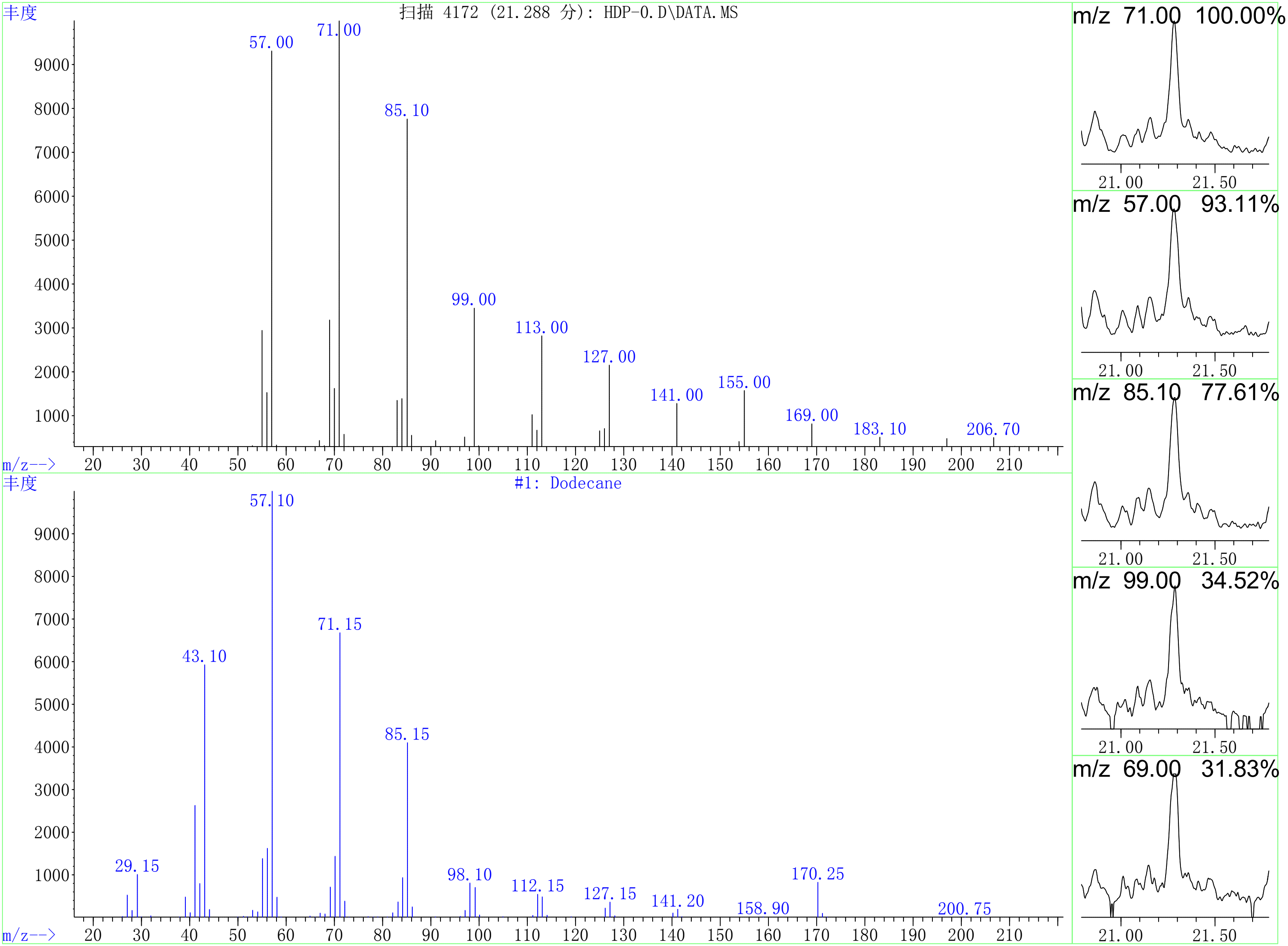

Data File: D:\GYM\DATA\2025\20251105\HDP-0.D
样品：HDP-0

峰编号： 17　21.288 分钟处　面积： 2493306　面积 % 0.07

每个谱库中 3 个最匹配的记录。	Ref\#	CAS\#	匹配度
C:\database\DEMO.L			
1 Dodecane	1	000112-40-3	25

未知谱图基于顶点

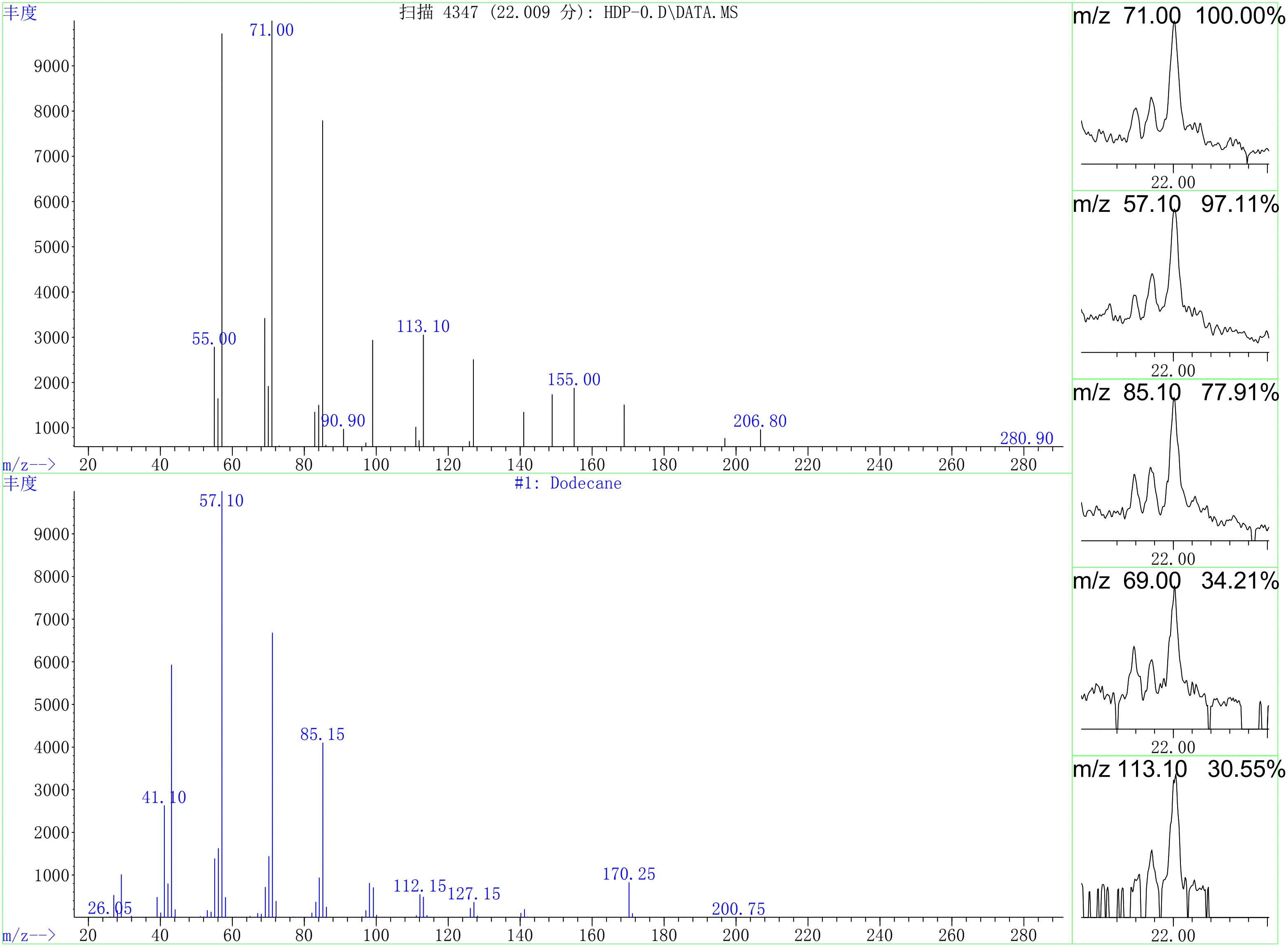

Data File: D:\GYM\DATA\2025\20251105\HDP-0.D
样品：HDP-0

峰编号： 18 22.009 分钟处 面积： 1397518 面积 % 0.04

每个谱库中 3 个最匹配的记录。 Ref\# CAS\# 匹配度

C:\database\DEMO.L
1 Dodecane 1 000112-40-3 40
