# Supplementary figures and images for: High-throughput chiral copper foils by curved-surface confinement recrystallization
Source: Nat Commun. 2026 Feb 20;17:2796. doi: 10.1038/s41467-026-69862-7 (PMC13022495; doi:10.1038/s41467-026-69862-7)

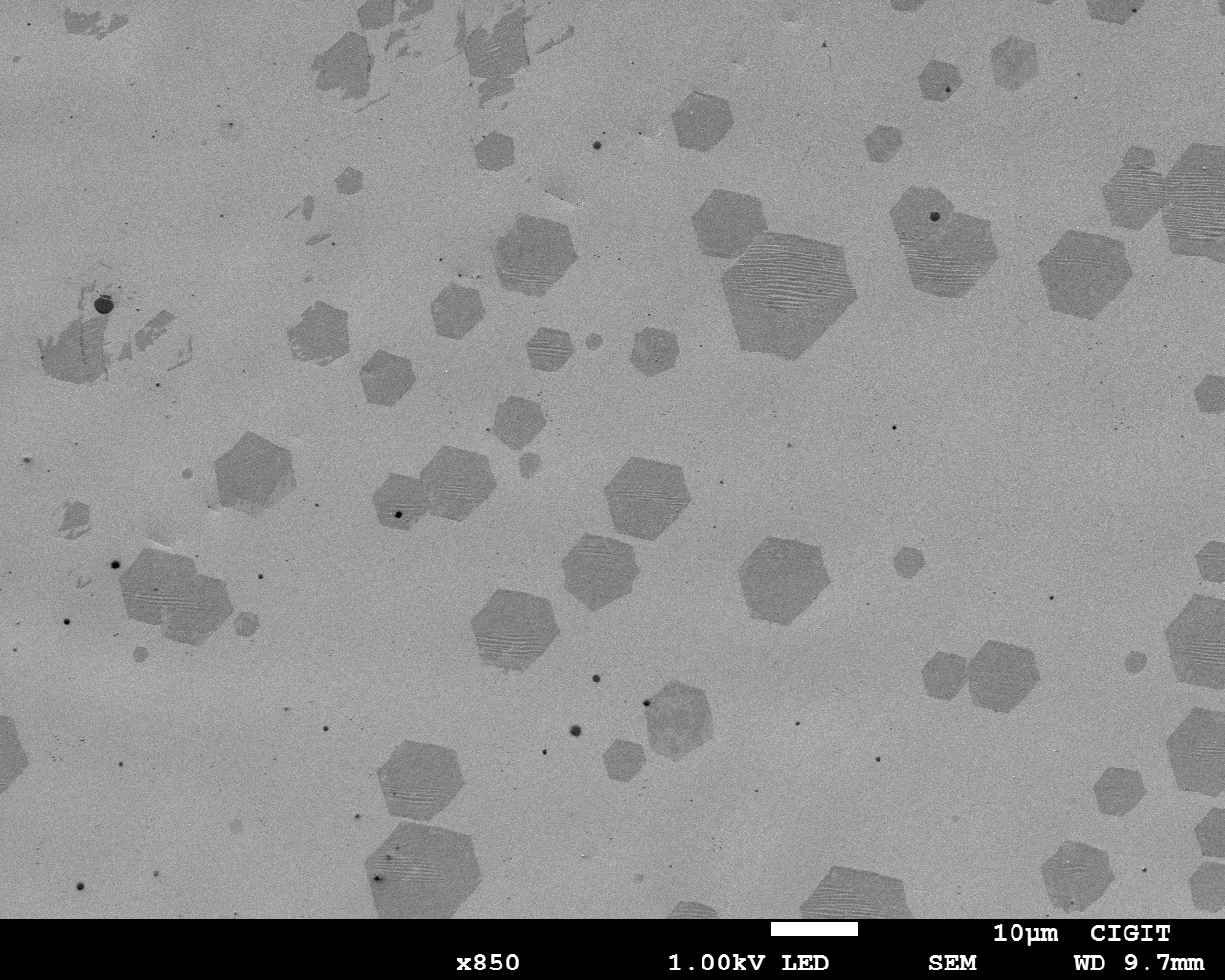

Supplement: Supplementary file 3 — Supplementary Dataset 1 [file 41467_2026_69862_MOESM3_ESM.zip › Supplementary Data 1/Figure 4/Figure 4b/1.bmp]

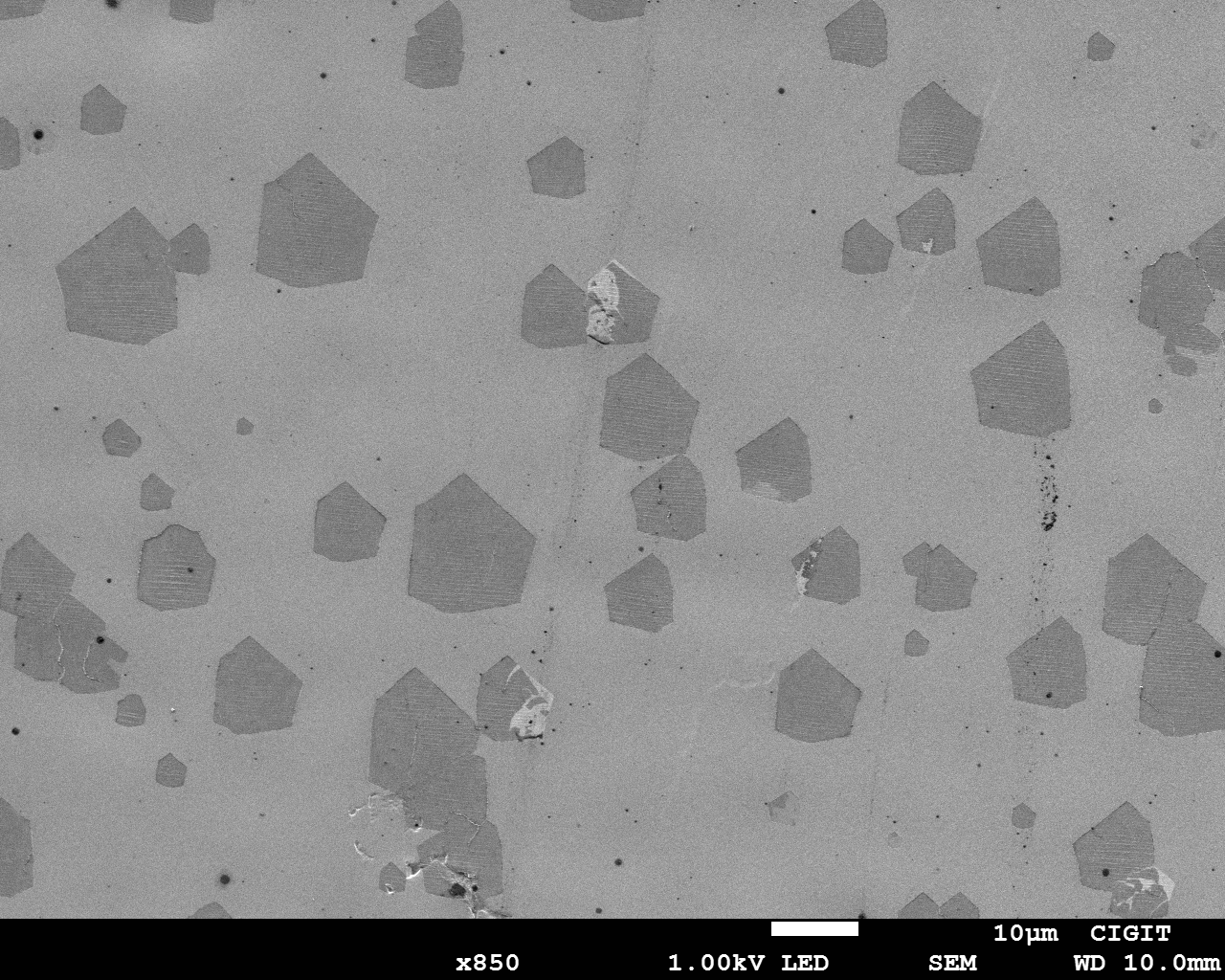

Supplement: Supplementary file 3 — Supplementary Dataset 1 [file 41467_2026_69862_MOESM3_ESM.zip › Supplementary Data 1/Figure 4/Figure 4b/2.bmp]

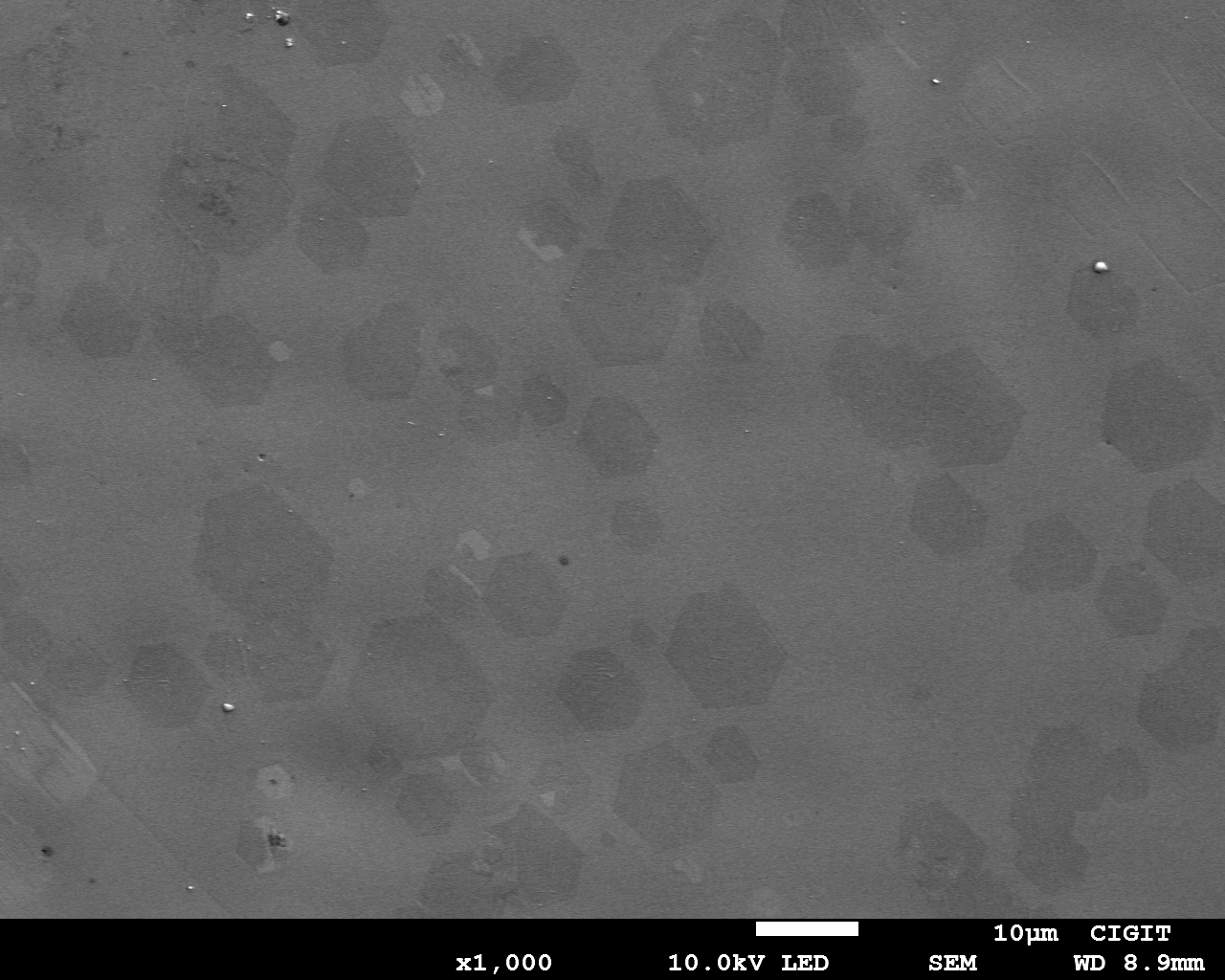

Supplement: Supplementary file 3 — Supplementary Dataset 1 [file 41467_2026_69862_MOESM3_ESM.zip › Supplementary Data 1/Figure 4/Figure 4b/3.bmp]

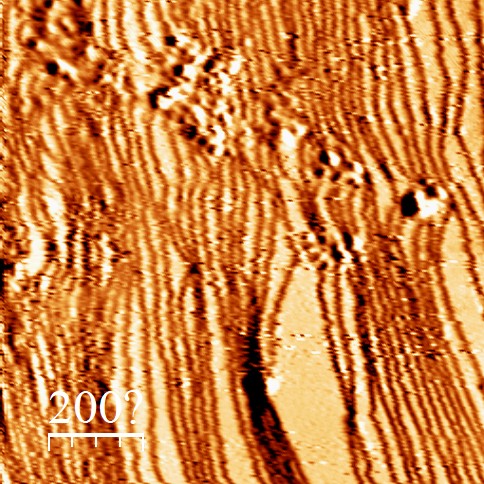

Supplement: Supplementary file 3 — Supplementary Dataset 1 [file 41467_2026_69862_MOESM3_ESM.zip › Supplementary Data 1/Supplementary Fig.7/STM4.jpg]

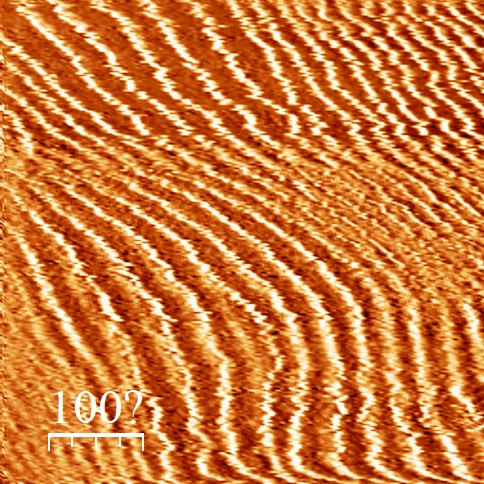

Supplement: Supplementary file 3 — Supplementary Dataset 1 [file 41467_2026_69862_MOESM3_ESM.zip › Supplementary Data 1/Supplementary Fig.7/STM3.jpg]

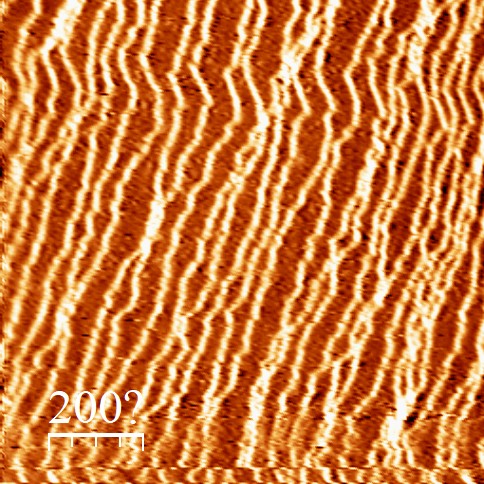

Supplement: Supplementary file 3 — Supplementary Dataset 1 [file 41467_2026_69862_MOESM3_ESM.zip › Supplementary Data 1/Supplementary Fig.7/STM2.jpg]

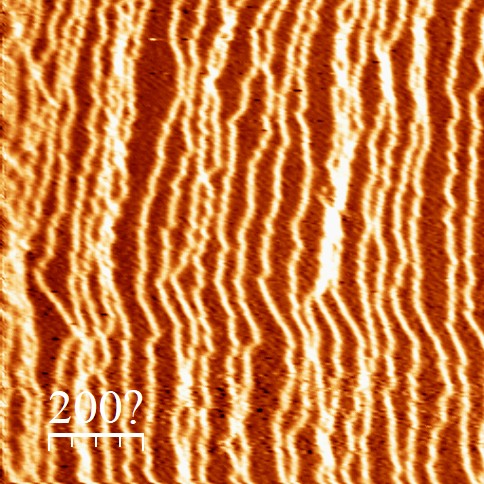

Supplement: Supplementary file 3 — Supplementary Dataset 1 [file 41467_2026_69862_MOESM3_ESM.zip › Supplementary Data 1/Supplementary Fig.7/STM1.jpg]

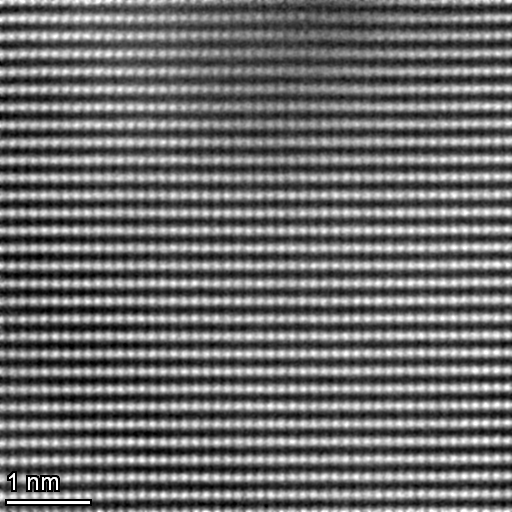

Supplement: Supplementary file 3 — Supplementary Dataset 1 [file 41467_2026_69862_MOESM3_ESM.zip › Supplementary Data 1/Supplementary Fig.1/1715 16.1 Mx HAADF STEM.tif]

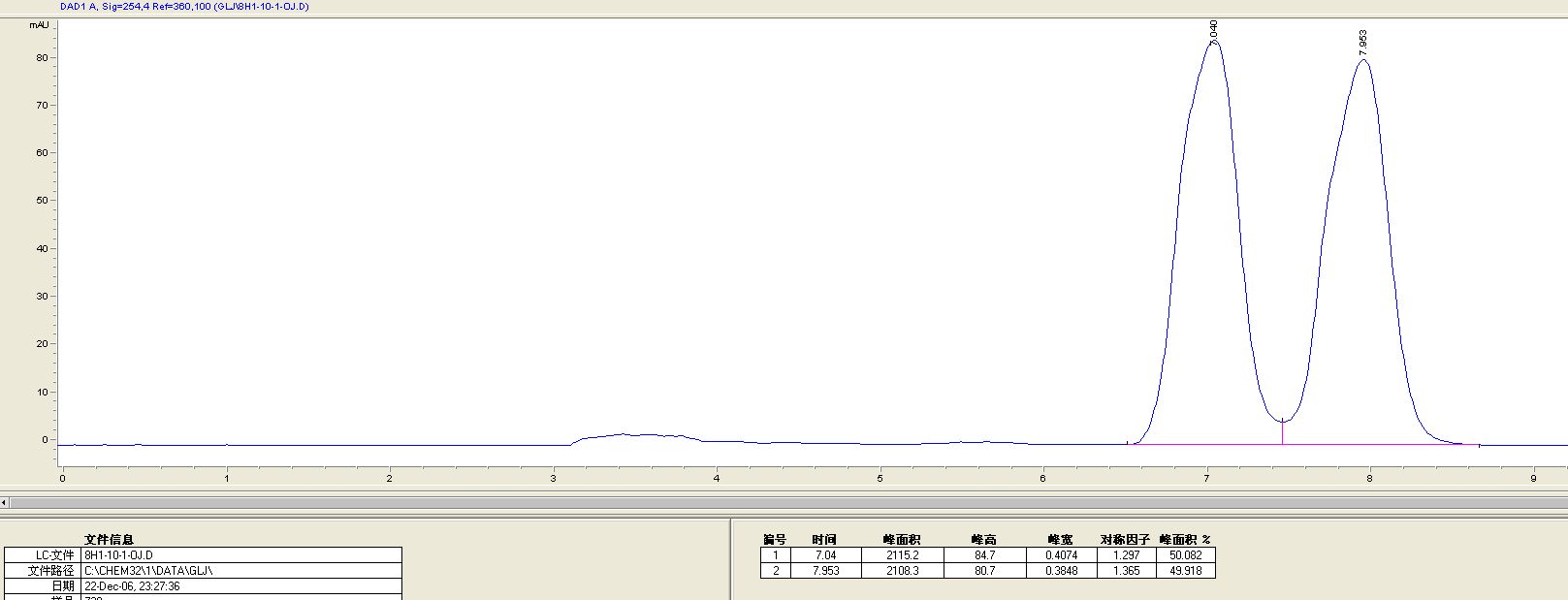

Supplement: Supplementary file 3 — Supplementary Dataset 1 [file 41467_2026_69862_MOESM3_ESM.zip › Supplementary Data 1/Supplementary Fig.14-16/HPLC Cu( 5 6 6).bmp.bmp]

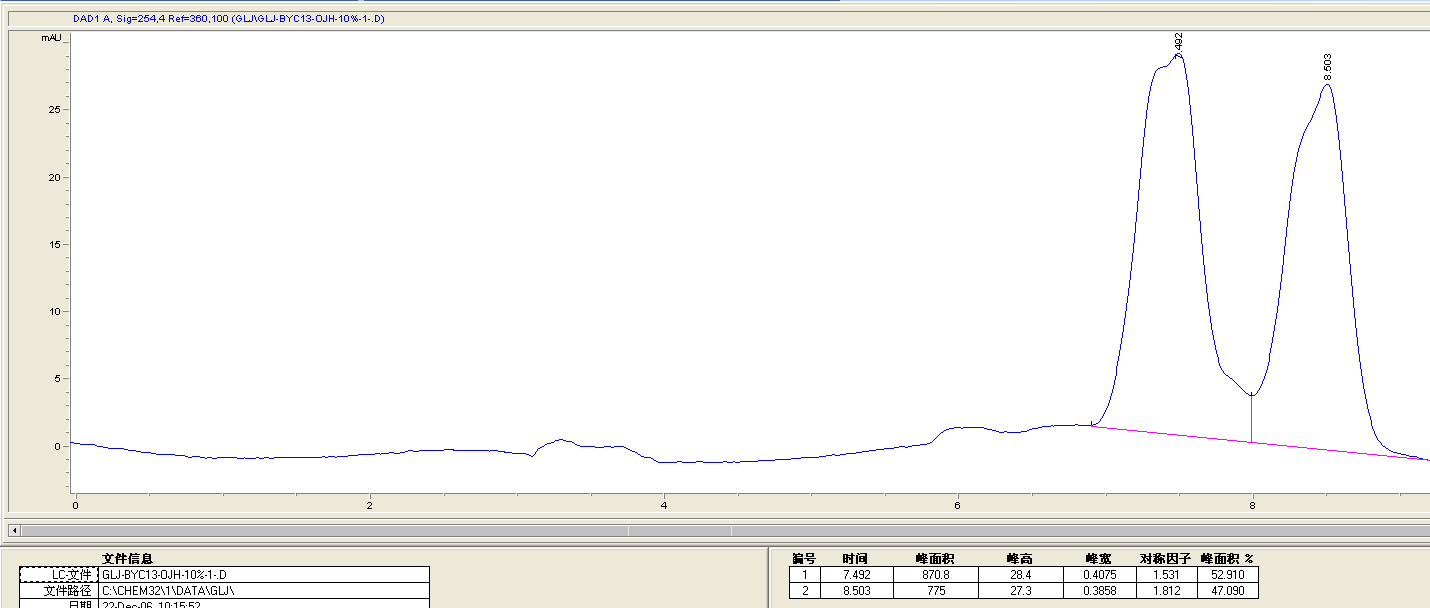

Supplement: Supplementary file 3 — Supplementary Dataset 1 [file 41467_2026_69862_MOESM3_ESM.zip › Supplementary Data 1/Supplementary Fig.14-16/HPLC Cu(06 69 72).bmp]

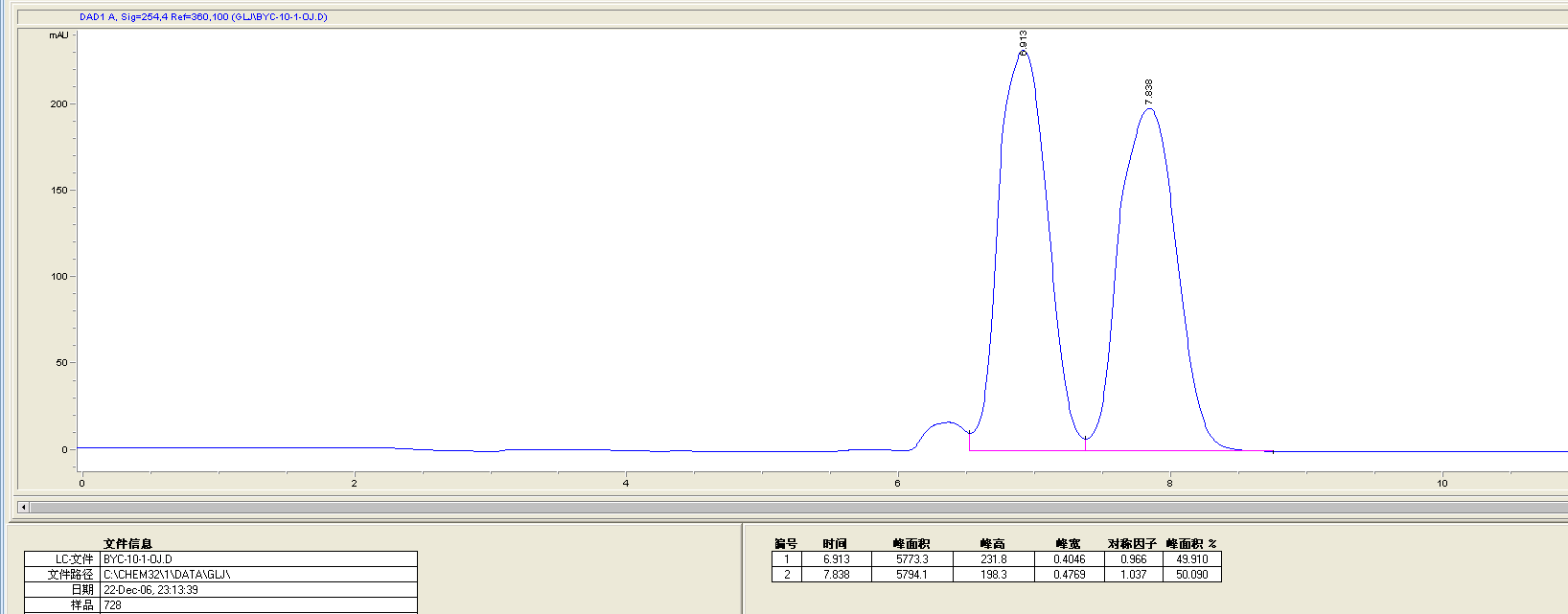

Supplement: Supplementary file 3 — Supplementary Dataset 1 [file 41467_2026_69862_MOESM3_ESM.zip › Supplementary Data 1/Supplementary Fig.14-16/HPLC Starting maaterials.bmp]

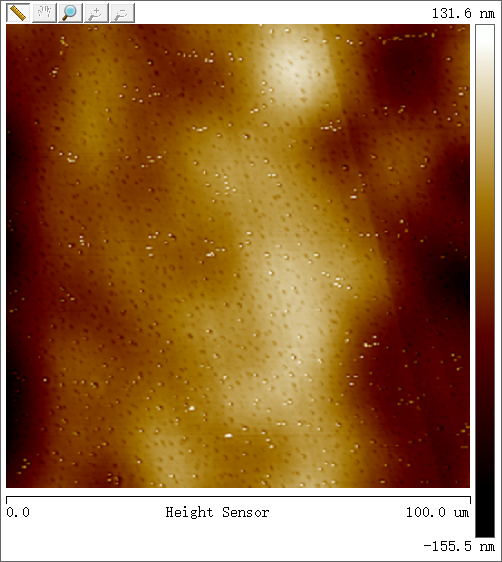

Supplement: Supplementary file 3 — Supplementary Dataset 1 [file 41467_2026_69862_MOESM3_ESM.zip › Supplementary Data 1/Supplementary Fig.6/After annealing (2).bmp]

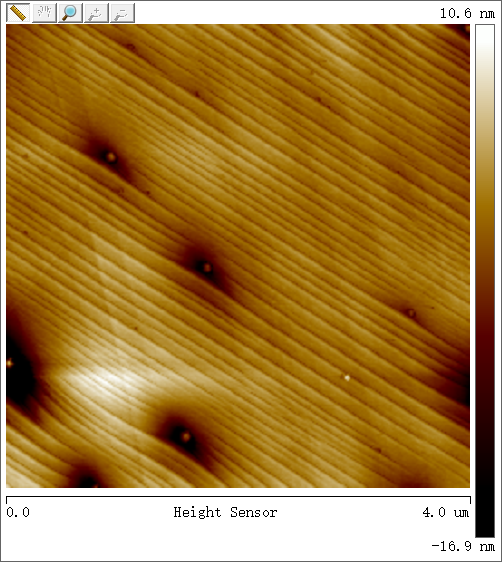

Supplement: Supplementary file 3 — Supplementary Dataset 1 [file 41467_2026_69862_MOESM3_ESM.zip › Supplementary Data 1/Supplementary Fig.6/After annealing (3).bmp]

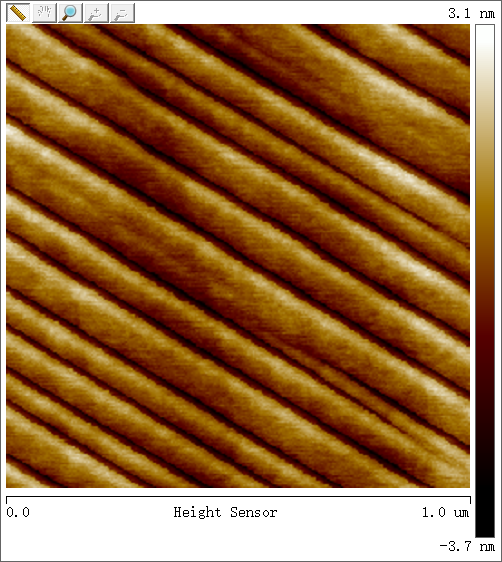

Supplement: Supplementary file 3 — Supplementary Dataset 1 [file 41467_2026_69862_MOESM3_ESM.zip › Supplementary Data 1/Supplementary Fig.6/After annealing (4).bmp]

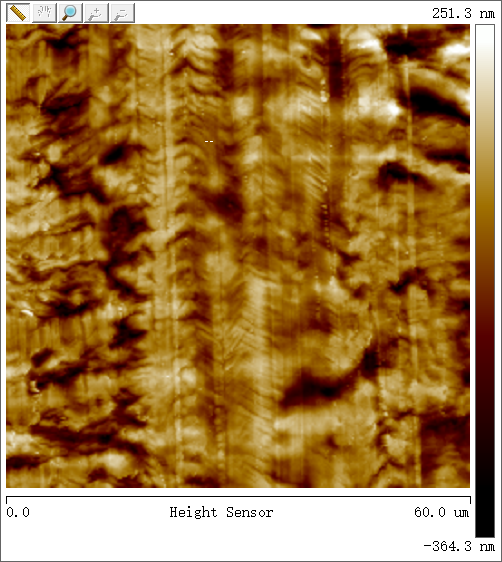

Supplement: Supplementary file 3 — Supplementary Dataset 1 [file 41467_2026_69862_MOESM3_ESM.zip › Supplementary Data 1/Supplementary Fig.6/Before annealing (2).bmp]

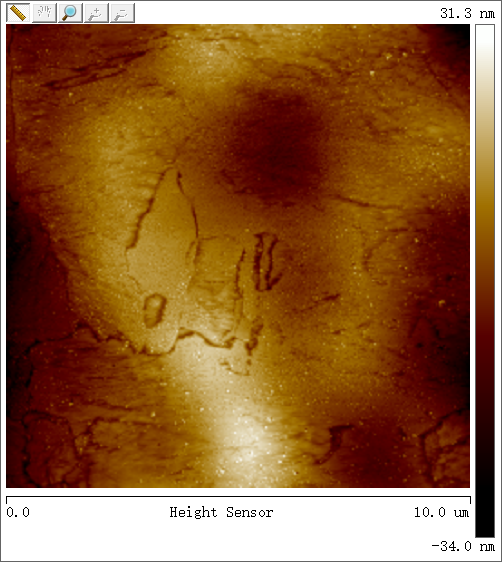

Supplement: Supplementary file 3 — Supplementary Dataset 1 [file 41467_2026_69862_MOESM3_ESM.zip › Supplementary Data 1/Supplementary Fig.6/Before annealing (3).bmp]

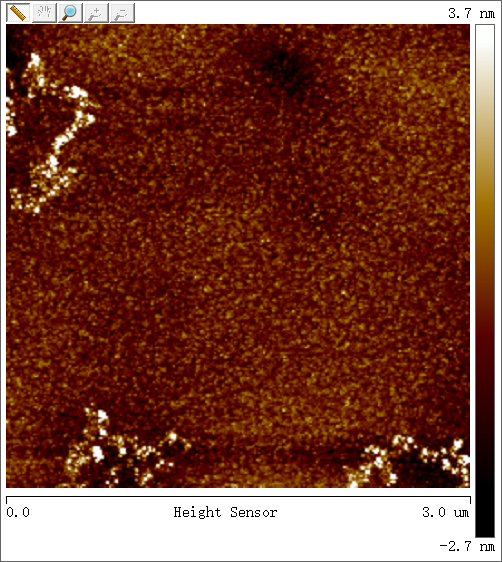

Supplement: Supplementary file 3 — Supplementary Dataset 1 [file 41467_2026_69862_MOESM3_ESM.zip › Supplementary Data 1/Supplementary Fig.6/Before annealing (4).bmp]
