# Supplementary material for: High-throughput chiral copper foils by curved-surface confinement recrystallization
Source: Nat Commun. 2026 Feb 20;17:2796. doi: 10.1038/s41467-026-69862-7 (PMC13022495; doi:10.1038/s41467-026-69862-7)
Supplement: Supplementary file 4 — Reporting Summary [file 41467_2026_69862_MOESM4_ESM.pdf]

nature portfolio

Corresponding author(s): Leining Zhang, Feng Ding, Haofei Shi

Last updated by author(s): Feb 1, 2026

Reporting Summary

Nature Portfolio wishes to improve the reproducibility of the work that we publish. This form provides structure for consistency and transparency in reporting. For further information on Nature Portfolio policies, see our Editorial Policies and the Editorial Policy Checklist.

Statistics

For all statistical analyses, confirm that the following items are present in the figure legend, table legend, main text, or Methods section.

n/a	Confirmed	
☐	☒	The exact sample size (*n*) for each experimental group/condition, given as a discrete number and unit of measurement
☐	☒	A statement on whether measurements were taken from distinct samples or whether the same sample was measured repeatedly
☐	☒	The statistical test(s) used AND whether they are one- or two-sided<br>*Only common tests should be described solely by name; describe more complex techniques in the Methods section.*
☒	☐	A description of all covariates tested
☒	☐	A description of any assumptions or corrections, such as tests of normality and adjustment for multiple comparisons
☐	☒	A full description of the statistical parameters including central tendency (e.g. means) or other basic estimates (e.g. regression coefficient) AND variation (e.g. standard deviation) or associated estimates of uncertainty (e.g. confidence intervals)
☒	☐	For null hypothesis testing, the test statistic (e.g. *F*, *t*, *r*) with confidence intervals, effect sizes, degrees of freedom and *P* value noted<br>*Give P values as exact values whenever suitable.*
☒	☐	For Bayesian analysis, information on the choice of priors and Markov chain Monte Carlo settings
☒	☐	For hierarchical and complex designs, identification of the appropriate level for tests and full reporting of outcomes
☒	☐	Estimates of effect sizes (e.g. Cohen's *d*, Pearson's *r*), indicating how they were calculated

Our web collection on statistics for biologists contains articles on many of the points above.

Software and code

Policy information about availability of computer code

Data collection	*Provide a description of all commercial, open source and custom code used to collect the data in this study, specifying the version used OR state that no software was used.*
Data analysis	Data fitting and initial plotting were performed using OriginPro (version, 2021). All final figures were compiled, arranged, and aesthetically refined using Adobe Illustrator (CC2019).

For manuscripts utilizing custom algorithms or software that are central to the research but not yet described in published literature, software must be made available to editors and reviewers. We strongly encourage code deposition in a community repository (e.g. GitHub). See the Nature Portfolio guidelines for submitting code & software for further information.

Data

Policy information about availability of data

All manuscripts must include a data availability statement. This statement should provide the following information, where applicable:

- Accession codes, unique identifiers, or web links for publicly available datasets
- A description of any restrictions on data availability
- For clinical datasets or third party data, please ensure that the statement adheres to our policy

The data that support the findings of this study are available from the corresponding authors upon request. Unprocessed raw data is provided as Supplementary Data 1.

Research involving human participants, their data, or biological material

Policy information about studies with human participants or human data. See also policy information about sex, gender (identity/presentation), and sexual orientation and race, ethnicity and racism.

Reporting on sex and gender	This study does not involve human participants, human data, or human tissue. Therefore, reporting on sex and gender is not applicable.
Reporting on race, ethnicity, or other socially relevant groupings	This study does not involve human participants, human data, or human tissue. Therefore, reporting on race, ethnicity, or other socially relevant groupings is not applicable.
Population characteristics	Not applicable. This is a materials science study utilizing copper foils and quartz tube setups. No human or animal population was involved.
Recruitment	Not applicable. This study did not recruit any human participants.
Ethics oversight	The experimental work in this study (materials annealing, characterization, and analysis) did not require ethics committee approval, as it did not involve human participants, human data, vertebrate animals, or protected specimens.

Note that full information on the approval of the study protocol must also be provided in the manuscript.

Field-specific reporting

Please select the one below that is the best fit for your research. If you are not sure, read the appropriate sections before making your selection.

- [x] Life sciences
- [ ] Behavioural & social sciences
- [ ] Ecological, evolutionary & environmental sciences

For a reference copy of the document with all sections, see nature.com/documents/nr-reporting-summary-flat.pdf

Life sciences study design

All studies must disclose on these points even when the disclosure is negative.

Sample size	Three independent samples (n=3) were chosen in accordance with standard practice in materials science, balancing experimental cost and statistical reliability. Robust data were ensured by taking a high number of technical replicates (e.g., $>$100 measurements per sample) to effectively characterize material properties and verify reproducibility.
Data exclusions	No data were excluded from the analyses; all collected data are presented in the analysis.
Replication	All experimental findings were highly reproducible. Multiple independent replicates were performed and presented, which consistently yielded the same trend, confirming the generality of the observed phenomenon.
Randomization	Randomization was not applicable to this study. The copper foil samples were not randomly assigned to experimental groups. Instead, each sample was intentionally prepared and annealed under specific, controlled curvature conditions to establish a deterministic gradient structure for systematic investigation. Sample selection for characterization was based on spatial position along the gradient, not random choice.
Blinding	Blinding was not applicable to this study. All material characterization measurements (e.g., EBSD, Raman spectroscopy) and data fitting procedures (e.g., linear regression) were performed objectively based on instrument outputs and established algorithms, eliminating the potential for subjective bias in data collection and analysis

Reporting for specific materials, systems and methods

We require information from authors about some types of materials, experimental systems and methods used in many studies. Here, indicate whether each material, system or method listed is relevant to your study. If you are not sure if a list item applies to your research, read the appropriate section before selecting a response.

Materials & experimental systems

n/a	Involved in the study	
x		Antibodies
x		Eukaryotic cell lines
x		Palaeontology and archaeology
x		Animals and other organisms
x		Clinical data
x		Dual use research of concern
x		Plants

Methods

n/a	Involved in the study	
x		ChIP-seq
x		Flow cytometry
x		MRI-based neuroimaging

Plants

Seed stocks	n/a
Novel plant genotypes	n/a
Authentication	n/a
